# TBPEH-TBPB Initiate the Radical Addition of Benzaldehyde and Allyl Esters

**DOI:** 10.3390/ijms232213704

**Published:** 2022-11-08

**Authors:** Binlong Sun, Xiaoyu Tian, Sen Yang, Yingying Wang, Yingying Shao, Xinhao Fu, Wenyuan Wang, Minting Tu, Yang Chen, Junhui Wu, Changyuan Wu, Chengxia Tan

**Affiliations:** College of Chemical Engineering, Zhejiang University of Technology, Hangzhou 310014, China

**Keywords:** radical addition, TBPEH-TBPB, olefin acylation, allyl ester, metal-free

## Abstract

Tert-butylperoxy-2-ethylhexanoate (TBPEH) and tert-butyl peroxybenzoate (TBPB) promote the radical acylation of allyl ester with benzaldehyde to synthesize new carbonyl-containing compounds under solvent-free and metal-free conditions. This reaction is compatible with electron-donating and halogen groups and has excellent atom utilization and chemical selectivity. Furthermore, the synthetic compounds can further apply to the preparation of lactone, piperidine, tetrazole and oxazole.

## 1. Introduction

The functionalization of alkenes is an important part of organic synthesis, which is widely used in various fields, such as synthetic drugs, natural products, and advanced materials [1,2,3,4,5,6,7]. Among them, the acylation of alkenes is a hotspot in chemical synthesis. For instance, the free radical addition reaction in the synthesis of glufosinate-ammonium by Hoechst company [8,9], which involved the alkene radical phosphorylation of the key intermediate 1-cyanoallyl acetate (ACA), was found to have the advantages of high rate, simple conditions and high atom economy. Moreover, the cyanohydrin [10,11,12,13,14,15,16,17] and carbon-carbon double bond [18,19,20,21,22,23] structure possessed by this compound have a wide range of applications in the chemical field and pharmaceutical synthesis, which determines its excellent practicability and ductility. However, few studies were conducted on 1-cyanoallyl acetate-related responses. 

What’s worth mentioning, though, is that the addition of benzaldehyde and olefins has attracted much attention because many asymmetric ketones or complex molecular skeletons that are difficult to synthesize by traditional methods can be constructed by the addition reaction of benzaldehydes with alkenes [24,25,26,27]. 

Hence, inspired by those mentioned above, according to the process conditions of glufosinate-ammonium and without changing the structure of cyanohydrin, the method development and structural modification of 1-cyanoallyl acetate were studied by taking benzaldehyde as the acylation reagent and TBPEH-TBPB as the initiators. Furthermore, the addition reactions of benzaldehyde with different kinds of allyl esters, like allyl hexanoate and allyl acetoacetate, were developed.

Herein, we report the radical addition reaction of 1-cyanoallyl acetate with benzaldehyde under metal-free conditions. In addition, we extensively studied the reactions of benzaldehyde with different kinds of allyl esters, like allyl hexanoate and allyl acetoacetate. Finally, compounds **3a**–**3y**, **4a**–**4u** and **5a**–**5o** were synthesized in one step, which can further apply to the preparation of lactone, piperidine, tetrazole and oxazole [28,29,30,31,32].

## 2. Results and Discussion

### 2.1. Synthesis of Target Compounds

**Scheme 1:**
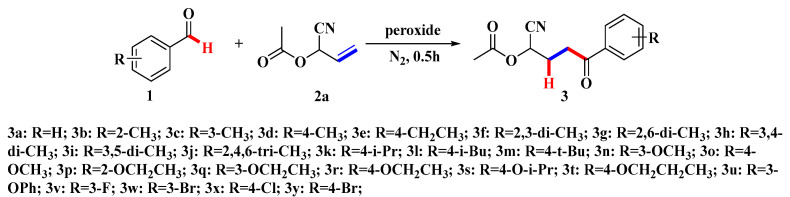
Synthetic route of compounds **3**.

The synthetic pathway to target compound **3** is shown in Scheme 1.

Initially, we screened our approach by optimizing the reaction conditions of benzaldehyde **1a** and 1-cyanallyl acetate **2a**, and the influence of different reaction conditions on the yields is shown in Table 1. We found that peroxide, solvent and temperature affected the reaction critically. In the beginning, the radical addition initiated by TBPEH showed a higher yield than that by TBHP, TBPB and other initiators (entries 1–3). The temperature was further screened, and it was found that lowering the temperature had a significant effect on the yield (entry 4). Subsequently, it was surprising that when we mixed TBPEH and TBPB under the same condition, the desired compound **3a** was isolated with a yield of 43% (entry 5). It was supposed that TBPEH plays the role of medium-temperature initiator and TBPB plays the role of high-temperature initiator, which means TBPEH decomposes and releases heat after being heated so that TBPB decomposes rapidly at a lower temperature [33,34]. Meanwhile, TBPB is decomposed to generate benzoyl radical, which is conducive to the reaction. Moreover, the corresponding product **3a** was afforded in 57% yield when the amount of TBPEH and TBPB was increased to 2 equiv. (entry 6). In addition, DMF and DMSO were selected as the solvents, but no target compounds were detected (entries 7–8). When the amount of benzaldehyde **1a** was reduced to 1.5 equiv., the yield of the desired product decreased to 26% (entry 9). From the subsequent reaction mechanism, it can be seen that benzaldehyde **1a** is the main source of H^+^, so 2 equiv. of benzaldehyde is needed, at least. According to Scheme 1, entry 6 was selected as the optimal condition.

**Figure 1:**
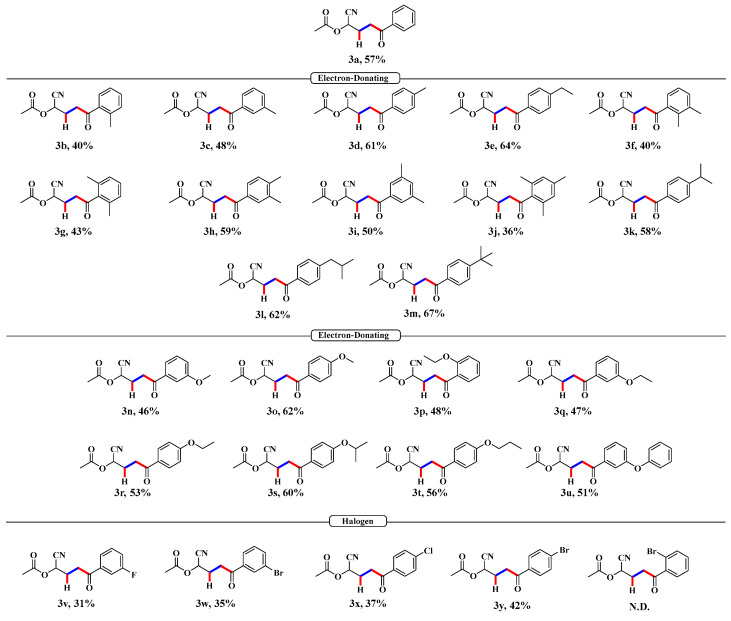
The substrate scope of benzaldehyde.

Next, we further explored the substrate scope of benzaldehydes under optimal conditions (Figure 1). We found that benzaldehydes substituted with electron-donating groups (R, OR) and halogens (F, Cl, Br) were suitable for this reaction, while the electron-donating groups contributed to the yields. This can be explained by the electron-donating effect, as the electron-donating groups made acyl radicals easier to generate, and the yield was higher than the benzaldehyde substituted with the electron-withdrawing group. For example, the order of yields was **3o** (4-OCH_3_) > **3a** (H) > **3y** (4-Br). As the results progressed, it was found that the strong electron-withdrawing groups (CN, NO_2_) were completely incompatible with this system. Besides the electronic effect of various functional groups, the position of the substitutes on the benzene ring also has a great influence on the yields. Due to the steric effect, the yield of ortho-position groups is lower than that of meta-position and para-position. In case as follow, compounds **3b** (ortho-, 40%), **3c** (meta-, 48%) and **3d** (para-, 61%). Additionally, 1-cyanoallyl acetate can hardly react with those benzaldehydes substituted with larger groups on the ortho position, such as 2-Br. In short, this reaction had high atom utilization and good chemical selectivity. Acyl radicals were always added at the 1-position of the alkenyl group. This special selectivity can be attributed to the anti-Markovnikov addition initiated by peroxides [35].

**Figure 2:**
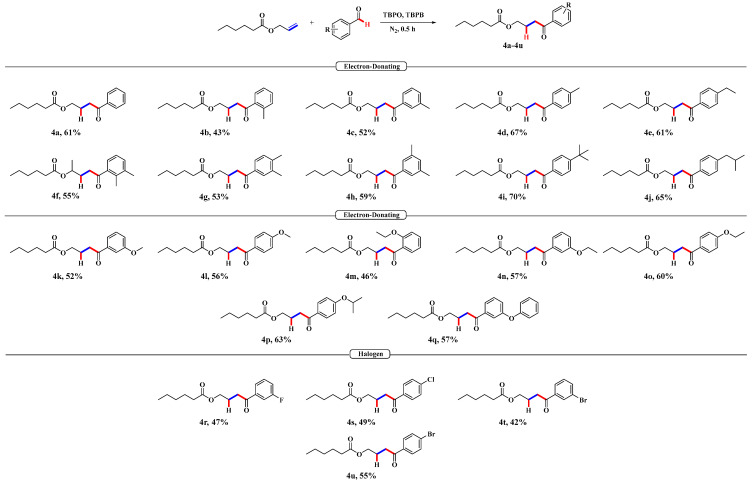
The radical additions of allyl hexanoate with substituted benzaldehyde.

**Figure 3:**
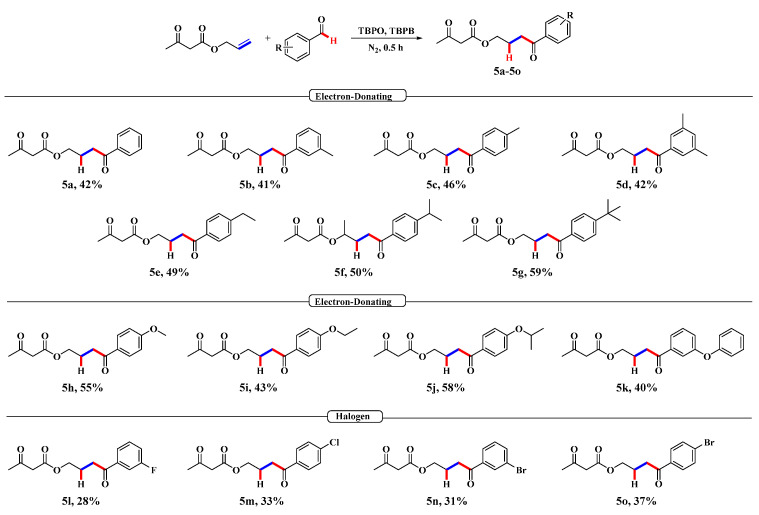
The radical additions of allyl acetoacetate with substituted benzaldehyde.

Subsequently, taking steric effect and application into consideration, the radical additions of substituted benzaldehyde with allyl hexanoate and allyl acetoacetate were performed under optimal conditions. The results are shown in Figure 2 and Figure 3, respectively. Compared to 1-cyanoallyl acetate, there was a slight yield increase in the reactions of allyl hexanoate (longer chain) with various substituted benzaldehyde, which may be due to the absence of the electron-withdrawing group (-CN). As for allyl acetoacetate, by-products formed, and the yields decreased. In the meantime, the steric effect was more obvious, and allyl acetoacetate could hardly react with ortho-substituted benzaldehyde. Overall, the electronic effect and steric effect of various substituents on the benzene ring still showed the same corresponding effects.

### 2.2. Mechanism

When benzaldehyde **1a** and 1-cyanoallyl acetate **2a** were reacted with 2,2,6,6-tetramethyl-1-piperidinyloxy (TEMPO) under the standard conditions [36,37], the TEMPO-trapped product **6a** (detected by GC-Mass analysis) was obtained, and the formation of **3a** was completely inhibited. It clearly supports that this reaction was a free radical reaction.

**Scheme 2:**
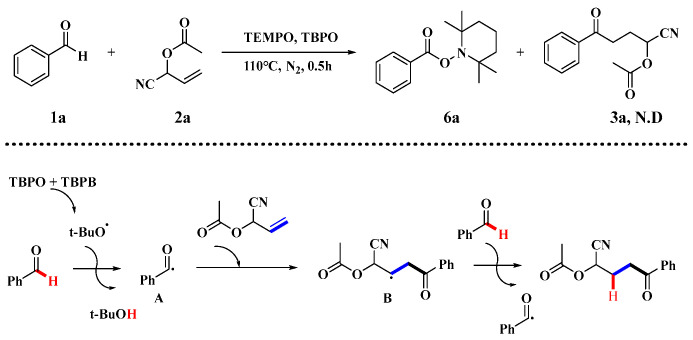
Plausible mechanism.

On the basis of these results and previous reports [20,38], we, therefore, proposed a possible mechanism, as shown in Scheme 2. The first step involves TBPEH and TBPB cleavage initiated by thermal conditions to give tert-butoxy radical, which then abstracts aldehydic hydrogen of **1a** to obtain benzoyl radical **A**. The addition of **A** to an alkene forms intermediate **B**, which in turn abstracts a hydrogen radical from another aldehyde to give compound **3a**.

## 3. Experimental Section

### 3.1. General Information

^1^H and ^13^C NMR spectra were recorded on a Bruker 500 MHz spectrometer in CDCl_3_ with TMS as the internal standard. GC-Mass spectra were determined on an Agilent 7890B spectrometer. High-resolution electrospray mass spectra (HR–ESI–MS) were determined using a UPLC H-CLASS/QTOF G2-XS mass spectrometer (Waters, Milford, MA, USA). All products were identified by ^1^H and ^13^C NMR and HRMS. The starting materials were purchased from Macklin, Rhawm, Adamas or TCI and used without further purification. The characterization data for all synthesized compounds are provided in the Supporting Information file (Figures S1–S184).

### 3.2. Synthesis

#### 3.2.1. General Synthesis of Compounds **3a**–**3y**

Substituted benzaldehydes (0.4 mmol), 1-cyanoallyl acetate (0.2 mmol), tert-butyl peroxybenzoate (0.4 mmol) and tert-butylperoxy-2-ethylhexanoate (0.4 mmol) were added to a flask. The mixture was reacted at 110 °C for 30 min. TLC was used to track the reaction progress. After the reaction was completed, it was separated by column chromatography to give the corresponding compound **3a**–**3y**.

1-cyano-4-oxo-4-phenylbutyl acetate (**3a**): Yellow liquid, yield 57%. ^1^H NMR (500 MHz, Chloroform-*d*) *δ* 8.06–7.90 (m, 2H), 7.66–7.59 (m, 1H), 7.54–7.47 (m, 2H), 5.53 (t, *J* = 6.5 Hz, 1H), 3.35–3.16 (m, 2H), 2.50–2.34 (m, 2H), 2.15 (s, 3H); ^13^C NMR (126 MHz, Chloroform-*d*) *δ* 197.28, 168.99, 136.22, 133.63, 128.80, 128.03, 116.62, 60.46, 33.08, 26.65, 20.36. HRMS calculated for C_13_H_13_NO_3_ (M + Na)^+^ 254.0788, found 254.0793.

1-cyano-4-oxo-4-(o-tolyl) butyl acetate (**3b**): Yellow liquid, yield 40%. ^1^H NMR (500 MHz, Chloroform-*d*) *δ* 7.70 (d, *J* = 7.5 Hz, 1H), 7.42 (td, *J* = 7.5, 1.0 Hz, 1H), 7.34–7.25 (m, 2H), 5.52 (t, *J* = 6.5 Hz, 1H), 3.21–3.14 (m, 2H), 2.53 (s, 3H), 2.42–2.33 (m, 2H), 2.15 (s, 3H); ^13^C NMR (126 MHz, Chloroform-*d*) *δ* 200.74, 168.99, 138.64, 136.70, 132.24, 131.91, 128.63, 125.86, 116.59, 60.44, 35.63, 26.78, 21.51, 20.34. HRMS calculated for C_14_H_15_NO_3_ (M + Na)^+^ 268.0944, found 268.0950.

1-cyano-4-oxo-4-(m-tolyl) butyl acetate (**3c**): Yellow liquid, yield 48%. ^1^H NMR (500 MHz, Chloroform-*d*) *δ* 7.87–7.70 (m, 2H), 7.44–7.36 (m, 2H), 5.52 (t, *J* = 6.5 Hz, 1H), 3.28–3.19 (m, 2H), 2.44 (s, 3H), 2.42–2.36 (m, 2H), 2.15 (s, 3H); ^13^C NMR (126 MHz, Chloroform-*d*) *δ* 197.45, 168.95, 138.61, 136.26, 134.34, 128.63, 128.51, 125.22, 116.61, 60.47, 33.11, 26.68, 21.33, 20.32. HRMS calculated for C_14_H_15_NO_3_ (M + Na)^+^ 268.0944, found 268.0945.

1-cyano-4-oxo-4-(p-tolyl) butyl acetate (**3d**): Yellow liquid, yield 61%. ^1^H NMR (500 MHz, Chloroform-*d*) *δ* 7.96–7.78 (m, 2H), 7.35–7.21 (m, 2H), 5.51 (t, *J* = 6.5 Hz, 1H), 3.32–3.12 (m, 2H), 2.44 (s, 3H), 2.42–2.36 (m, 2H), 2.14 (s, 3H); ^13^C NMR (126 MHz, Chloroform-*d*) *δ* 196.87, 168.97, 144.49, 133.77, 129.43, 128.12, 116.65, 60.50, 32.92, 26.70, 21.68, 20.34. HRMS calculated for C_14_H_15_NO_3_ (M + Na)^+^ 268.0944, found 268.0950.

1-cyano-4-(4-ethylphenyl)-4-oxobutyl acetate (**3e**): Yellow liquid, yield 64%. ^1^H NMR (500 MHz, Chloroform-*d*) *δ* 7.90 (d, *J* = 8.5 Hz, 2H), 7.37–7.27 (m, 2H), 5.51 (t, *J* = 6.5 Hz, 1H), 3.31–3.14 (m, 2H), 2.73 (q, *J* = 7.5 Hz, 2H), 2.47–2.32 (m, 2H), 2.14 (s, 3H), 1.28–1.25 (m, 3H); ^13^C NMR (126 MHz, Chloroform-*d*) *δ* 196.92, 168.97, 150.65, 133.98, 128.25, 128.23, 116.64, 60.50, 32.94, 28.96, 26.70, 20.32, 15.15. HRMS calculated for C_15_H_17_NO_3_ (M + Na)^+^ 282.1101 found 282.1107.

1-cyano-4-(2,3-dimethylphenyl)-4-oxobutyl acetate (**3f**): Yellow liquid, yield 40%. ^1^H NMR (500 MHz, Chloroform-*d*) *δ* 7.38 (d, *J* = 7.5 Hz, 1H), 7.30 (d, *J* = 7.0 Hz, 1H), 7.18 (t, *J* = 7.5 Hz, 1H), 5.52 (t, *J* = 6.5 Hz, 1H), 3.19–3.05 (m, 2H), 2.42–2.35 (m, 2H), 2.33 (s, 6H), 2.16 (s, 3H); ^13^C NMR (126 MHz, Chloroform-*d*) *δ* 202.91, 168.93, 138.91, 138.54, 135.36, 132.82, 125.35, 125.17, 116.53, 60.39, 36.79, 26.79, 20.41, 20.31, 16.43. HRMS calculated for C_15_H_17_NO_3_ (M + Na)^+^ 282.1101 found 282.1110.

1-cyano-4-(2,6-dimethylphenyl)-4-oxobutyl acetate (**3g**): Yellow liquid, yield 43%. ^1^H NMR (500 MHz, Chloroform-*d*) *δ* 7.20 (t, *J* = 7.5 Hz, 1H), 7.05 (d, *J* = 7.5 Hz, 2H), 5.54 (t, *J* = 6.5 Hz, 1H), 3.00–2.91 (m, 2H), 2.46–2.32 (m, 2H), 2.24 (s, 6H), 2.17 (s, 3H); ^13^C NMR (126 MHz, Chloroform-*d*) *δ* 207.56, 168.87, 141.34, 132.34, 128.96, 127.89, 116.42, 60.28, 39.05, 26.07, 20.29, 19.07. HRMS calculated for C_15_H_17_NO_3_ (M + Na)^+^ 282.1101 found 282.1109.

1-cyano-4-(3,4-dimethylphenyl)-4-oxobutyl acetate (**3h**): Yellow liquid, yield 59%. ^1^H NMR (500 MHz, Chloroform-*d*) *δ* 7.75 (s, 1H), 7.71 (dd, *J* = 8.0, 2.0 Hz, 1H), 7.25 (d, *J* = 8.0 Hz, 1H), 5.52 (t, *J* = 6.5 Hz, 1H), 3.31–3.10 (m, 2H), 2.44–2.36 (m, 2H), 2.34 (s, 6H), 2.15 (s, 3H); ^13^C NMR (126 MHz, Chloroform-*d*) *δ* 197.11, 168.95, 143.19, 137.13, 134.18, 129.95, 129.11, 125.72, 116.64, 60.52, 32.92, 26.75, 20.31, 20.02, 19.75. HRMS calculated for C_15_H_17_NO_3_ (M + Na)^+^ 282.1101 found 282.1105.

1-cyano-4-(3,5-dimethylphenyl)-4-oxobutyl acetate (**3i**): Yellow liquid, yield 50%. ^1^H NMR (500 MHz, Chloroform-*d*) *δ* 7.58 (d, *J* = 1.5 Hz, 2H), 7.25 (s, 1H), 5.52 (t, *J* = 6.5 Hz, 1H), 3.28–3.16 (m, 2H), 2.40 (s, 6H), 2.38–2.36 (m, 2H), 2.15 (s, 3H); ^13^C NMR (126 MHz, Chloroform-*d*) *δ* 197.60, 168.94, 138.43, 136.35, 135.20, 125.78, 116.63, 60.50, 33.15, 26.71, 21.20, 20.32. HRMS calculated for C_15_H_17_NO_3_ (M + Na)^+^ 282.1101 found 282.1108.

1-cyano-4-mesityl-4-oxobutyl acetate (**3j**): Yellow liquid, yield 36%. ^1^H NMR (500 MHz, Chloroform-*d*) *δ* 6.87 (d, *J* = 1.0 Hz, 2H), 5.53 (t, *J* = 6.5 Hz, 1H), 2.98–2.89 (m, 2H), 2.42–2.32 (m, 2H), 2.30 (s, 3H), 2.20 (s, 6H), 2.16 (s, 3H); ^13^C NMR (126 MHz, Chloroform-*d*) *δ* 207.76, 168.88, 138.86, 138.65, 132.42, 128.62, 116.43, 60.30, 39.17, 26.16, 21.01, 20.30, 19.06. HRMS calculated for C_16_H_19_NO_3_ (M + Na)^+^ 296.1257, found: 296.1266.

1-cyano-4-(4-isopropylphenyl)-4-oxobutyl acetate (**3k**): Yellow liquid, yield 58%. ^1^H NMR (500 MHz, Chloroform-*d*) *δ* 7.92 (d, *J* = 8.0 Hz, 2H), 7.35 (d, *J* = 8.5 Hz, 2H), 5.52 (t, *J* = 6.5 Hz, 1H), 3.30–3.12 (m, 2H), 3.07–2.88 (m, 1H), 2.50–2.31 (m, 2H), 2.15 (s, 3H), 1.29 (d, *J* = 7.0 Hz, 6H); ^13^C NMR (126 MHz, Chloroform-*d*) *δ* 196.87, 168.93, 155.21, 134.13, 128.27, 126.84, 116.61, 60.50, 34.27, 32.93, 26.73, 23.61, 20.30. HRMS calculated for C_16_H_19_NO_3_ (M + Na)^+^ 296.1257, found: 296.1263.

1-cyano-4-(4-isobutylphenyl)-4-oxobutyl acetate (**3l**): Yellow liquid, yield 62%. ^1^H NMR (500 MHz, Chloroform-*d*) *δ* 7.90 (d, *J* = 8.3 Hz, 2H), 7.27 (d, *J* = 8.5 Hz, 2H), 5.52 (t, *J* = 6.5 Hz, 1H), 3.30–3.11 (m, 2H), 2.56 (d, *J* = 7.2 Hz, 2H), 2.47–2.33 (m, 2H), 2.15 (s, 3H), 1.96–1.85 (m, 1H), 0.93 (d, *J* = 6.6 Hz, 6H); ^13^C NMR (126 MHz, Chloroform-*d*) *δ* 196.92, 168.93, 148.21, 134.03, 129.46, 127.99, 116.61, 60.51, 45.39, 32.93, 30.08, 26.73, 22.29, 20.30. HRMS calculated for C_17_H_21_NO_3_ (M + Na)^+^ 310.1414, found 310.1412.

4-(4-(tert-butyl) phenyl)-1-cyano-4-oxobutyl acetate (**3m**): Yellow liquid, yield 67%. ^1^H NMR (500 MHz, Chloroform-*d*) *δ* 7.92 (d, *J* = 9.0 Hz, 2H), 7.51 (d, *J* = 8.5 Hz, 2H), 5.52 (t, *J* = 6.5 Hz, 1H), 3.31–3.14 (m, 2H), 2.47–2.35 (m, 2H), 2.15 (s, 3H), 1.36 (s, 9H); ^13^C NMR (126 MHz, Chloroform-*d*) *δ* 196.90, 168.96, 157.44, 133.67, 127.99, 125.71, 116.64, 60.50, 35.18, 32.94, 31.05, 26.70, 20.33. HRMS calculated for C_17_H_21_NO_3_ (M + Na)^+^ 310.1414, found 310.1423.

1-cyano-4-(3-methoxyphenyl)-4-oxobutyl acetate (**3n**): Yellow liquid, yield 46%. ^1^H NMR (500 MHz, Chloroform-*d*) *δ* 7.56 (dt, *J* = 7.5, 1.0 Hz, 1H), 7.52–7.49 (m, 1H), 7.41 (t, *J* = 8.0 Hz, 1H), 7.16 (m, 1H), 5.52 (t, *J* = 6.5 Hz, 1H), 3.88 (s, 3H), 3.32–3.15 (m, 2H), 2.47–2.32 (m, 2H), 2.15 (s, 3H); ^13^C NMR (126 MHz, Chloroform-*d*) *δ* 197.08, 168.92, 159.96, 130.17, 129.75, 126.17, 120.59, 119.97, 112.37, 60.42, 55.48, 33.19, 26.73, 20.31. HRMS calculated for C_14_H_15_NO_4_ (M + Na)^+^ 284.0893, found 284.0901.

1-cyano-4-(4-methoxyphenyl)-4-oxobutyl acetate (**3o**): Yellow liquid, yield 62%. ^1^H NMR (500 MHz, Chloroform-*d*) *δ* 7.96 (d, *J* = 9.0 Hz, 2H), 6.96 (d, *J* = 9.0 Hz, 2H), 5.51 (t, *J* = 6.5 Hz, 1H), 3.89 (s, 3H), 3.28–3.07 (m, 2H), 2.42–2.34 (m, 2H), 2.14 (s, 3H); ^13^C NMR (126 MHz, Chloroform-*d*) *δ* 194.73, 167.95, 162.87, 129.31, 128.34, 115.64, 112.91, 59.55, 54.51, 31.65, 25.80, 19.31. HRMS calculated for C_14_H_15_NO_4_ (M + Na)^+^ 284.0893, found 284.0900.

1-cyano-4-(2-ethoxyphenyl)-4-oxobutyl acetate (**3p**): Yellow liquid, yield 48%. ^1^H NMR (500 MHz, Chloroform-*d*) *δ* 7.76 (dd, *J* = 8.0, 2.0 Hz, 1H), 7.55–7.43 (m, 1H), 7.01 (t, *J* = 7.5 Hz, 1H), 6.97 (d, *J* = 8.5 Hz, 1H), 5.48 (t, *J* = 6.5 Hz, 1H), 4.17 (q, *J* = 7.0 Hz, 2H), 3.30 (td, *J* = 7.0, 2.0 Hz, 2H), 2.35 (q, *J* = 7.0 Hz, 2H), 2.14 (s, 3H); ^13^C NMR (126 MHz, Chloroform-*d*) *δ* 198.22, 167.99, 157.34, 133.09, 129.52, 126.14, 119.60, 115.72, 111.36, 63.18, 59.68, 37.59, 19.32, 13.74. HRMS calculated for C_15_H_17_NO_4_ (M +Na)^+^ 298.1050, found 298.1057.

1-cyano-4-(3-ethoxyphenyl)-4-oxobutyl acetate (**3q**): Yellow liquid, yield 47%. ^1^H NMR (500 MHz, Chloroform-*d*) *δ* 7.56–7.53 (m, 1H), 7.50–7.48 (m, 1H), 7.40 (t, *J* = 8.0 Hz, 1H), 7.16–7.12 (m, 1H), 5.52 (t, *J* = 6.5 Hz, 1H), 4.17–4.06 (m, 2H), 3.26–3.19 (m, 2H), 2.43–2.36 (m, 2H), 2.15 (s, 3H), 1.46 (t, *J* = 7.0 Hz, 3H); ^13^C NMR (126 MHz, Chloroform-*d*) *δ* 197.13, 168.95, 159.29, 137.52, 129.74, 120.42, 120.34, 116.58, 113.08, 63.77, 60.43, 33.18, 26.70, 20.33, 14.72. HRMS calculated for C_15_H_17_NO_4_ (M + Na)^+^ 298.1050, found 298.1059.

1-cyano-4-(4-ethoxyphenyl)-4-oxobutyl acetate (**3r**): Yellow liquid, yield 53%. ^1^H NMR (500 MHz, Chloroform-*d*) *δ* 7.95 (d, *J* = 9.0 Hz, 2H), 6.95 (d, *J* = 9.0 Hz, 2H), 5.52 (t, *J* = 6.5 Hz, 1H), 4.26–4.02 (m, 2H), 3.32–3.03 (m, 2H), 2.46–2.29 (m, 2H), 2.15 (s, 3H), 1.46 (t, *J* = 7.0 Hz, 3H); ^13^C NMR (126 MHz, Chloroform-*d*) *δ* 195.72, 168.97, 163.29, 130.31, 129.12, 114.32, 63.83, 60.55, 32.62, 26.79, 20.33, 14.63. HRMS calculated for C_15_H_17_NO_4_ (M + Na)^+^ 298.1050, found 298.1058.

1-cyano-4-(4-isopropoxyphenyl)-4-oxobutyl acetate (**3s**): Yellow liquid, yield 60%. ^1^H NMR (500 MHz, Chloroform-*d*) *δ* 7.94 (d, *J* = 9.0 Hz, 2H), 6.93 (d, *J* = 9.0 Hz, 2H), 5.51 (t, *J* = 6.5 Hz, 1H), 4.67 (p, *J* = 6.0 Hz, 1H), 3.26–3.12 (m, 2H), 2.44–2.33 (m, 2H), 2.15 (s, 3H), 1.39 (d, *J* = 6.0 Hz, 6H); ^13^C NMR (126 MHz, Chloroform-*d*) *δ* 195.70, 168.98, 162.39, 130.34, 128.86, 116.67, 115.23, 70.21, 60.56, 32.59, 26.79, 21.87, 20.33. HRMS calculated for C_16_H_19_NO_4_ (M + Na)^+^ 312.1206, found 312.1210.

1-cyano-4-oxo-4-(4-propoxyphenyl) butyl acetate (**3t**): Yellow liquid, yield 56%. ^1^H NMR (500 MHz, Chloroform-*d*) *δ* 7.95 (d, *J* = 9.0 Hz, 2H), 6.95 (d, *J* = 9.0 Hz, 2H), 5.51 (s, 1H), 4.00 (t, *J* = 7.0 Hz, 2H), 3.20–3.17 (m, 2H), 2.45–2.32 (m, 2H), 2.14 (s, 3H), 1.85 (q, *J* = 6.5 Hz, 2H), 1.07 (t, *J* = 7.0 Hz, 3H); ^13^C NMR (126 MHz, Chloroform-*d*) *δ* 195.74, 168.98, 163.50, 130.29, 129.07, 114.34, 69.80, 60.55, 32.62, 26.79, 22.42, 20.33, 10.43. HRMS calculated for C_16_H_19_NO_4_ (M + Na)^+^ 312.1206, found 312.1208.

1-cyano-4-oxo-4-(3-phenoxyphenyl) butyl acetate (**3u**): Yellow liquid, yield 51%. ^1^H NMR (500 MHz, Chloroform-*d*) *δ* 7.70 (dt, *J* = 7.5, 1.0 Hz, 1H), 7.60 (t, *J* = 2.0 Hz, 1H), 7.46 (t, *J* = 8.0 Hz, 1H), 7.43–7.35 (m, 2H), 7.25 (m, 1H), 7.21–7.14 (m, 1H), 7.09–7.00 (m, 2H), 5.51 (t, *J* = 6.5 Hz, 1H), 3.37–3.04 (m, 2H), 2.56–2.27 (m, 2H), 2.15 (s, 3H); ^13^C NMR (126 MHz, Chloroform-*d*) *δ* 196.63, 168.90, 157.98, 137.95, 130.13, 129.99, 123.99, 123.67, 122.64, 119.19, 117.75, 60.37, 33.24, 26.63, 20.30. HRMS calculated for C_19_H_17_NO_4_ (M + Na)^+^ 346.1050 found 346.1057.

1-cyano-4-(3-fluorophenyl)-4-oxobutyl acetate (**3v**): Yellow liquid, yield 31%. ^1^H NMR (500 MHz, Chloroform-*d*) *δ* 7.79–7.74 (m, 1H), 7.69–7.64 (m, 1H), 7.53–7.46 (m, 1H), 7.35–7.29 (m, 1H), 5.53 (t, *J* = 6.5 Hz, 1H), 3.33–3.14 (m, 2H), 2.47–2.35 (m, 2H), 2.16 (s, 3H); ^13^C NMR (126 MHz, Chloroform-*d*) *δ* 194.97, 167.89, 162.90, 137.25 (d, *J* = 6.4 Hz), 129.48 (d, *J* = 7.6 Hz), 122.74, 119.64 (d, *J* = 21.4 Hz), 115.47, 113.77 (d, *J* = 22.4 Hz), 59.32, 32.28, 25.55, 19.30. HRMS calculated for C_13_H_12_FNO_3_ (M + Na)^+^ 272.0693, found 272.0699.

4-(3-bromophenyl)-1-cyano-4-oxobutyl acetate (**3w**): Yellow liquid, yield 35%. ^1^H NMR (500 MHz, Chloroform-*d*) *δ* 8.11 (t, *J* = 2.0 Hz, 1H), 7.91 (m, 1H), 7.74 (m, 1H), 7.39 (t, *J* = 8.0 Hz, 1H), 5.53 (t, *J* = 6.5 Hz, 1H), 3.22 (m, 2H), 2.40 (m, 2H), 2.16 (s, 3H); ^13^C NMR (126 MHz, Chloroform-*d*) *δ* 195.88, 168.89, 137.90, 136.44, 131.08, 130.36, 126.51, 123.15, 116.47, 60.30, 33.21, 26.52, 20.31. HRMS calculated for C_13_H_12_BrNO_3_ (M + Na)^+^ 331.9893, found 331.9901.

4-(4-chlorophenyl)-1-cyano-4-oxobutyl acetate (**3x**): Yellow liquid, yield 37%. ^1^H NMR (500 MHz, Chloroform-*d*) *δ* 7.92 (d, *J* = 8.5 Hz, 2H), 7.48 (d, *J* = 8.5 Hz, 2H), 5.52 (t, *J* = 6.0 Hz, 1H), 3.31–3.11 (m, 2H), 2.48–2.31 (m, 2H), 2.15 (s, 3H); ^13^C NMR (126 MHz, Chloroform-*d*) *δ* 196.00, 168.89, 140.13, 134.53, 129.40, 129.11, 116.50, 60.36, 33.07, 26.58, 20.29. HRMS calculated for C_13_H_12_ClNO_3_ (M + Na)^+^ 288.0398 found 288.0405.

4-(4-bromophenyl)-1-cyano-4-oxobutyl acetate (**3y**): Yellow liquid, yield 42%. ^1^H NMR (500 MHz, Chloroform-*d*) *δ* 7.85 (d, *J* = 8.5 Hz, 2H), 7.65 (d, *J* = 8.5 Hz, 2H), 5.52 (t, *J* = 6.0 Hz, 1H), 3.29–3.11 (m, 2H), 2.46–2.34 (m, 2H), 2.15 (s, 3H); ^13^C NMR (126 MHz, Chloroform-*d*) *δ* 196.19, 168.88, 134.92, 132.11, 129.48, 128.85, 116.49, 60.34, 33.05, 26.56, 20.30. HRMS calculated for C_13_H_12_BrNO_3_ (M + Na)^+^ 331.9893, found 331.9899.

#### 3.2.2. General Synthesis of Compounds **4a**–**4u** and **5a**–**5o**

The synthesis of compounds **4a**–**4u** and **5a**–**5o** was similar to that of **3a**–**3y**.

4-oxo-4-phenylbutyl hexanoate (**4a**): Colorless liquid, yield 61%. ^1^H NMR (500 MHz, Chloroform-*d*) *δ* 7.98 (dd, *J* = 8.0, 1.0 Hz, 2H), 7.67–7.52 (m, 1H), 7.48 (t, *J* = 8.0 Hz, 2H), 4.19 (t, *J* = 6.5 Hz, 2H), 3.08 (t, *J* = 7.0 Hz, 2H), 2.30 (t, *J* = 7.5 Hz, 2H), 2.16–2.07 (m, 2H), 1.65–1.62 (m, 2H), 1.33–1.29 (m, 4H), 0.90 (t, *J* = 7.0 Hz, 3H); ^13^C NMR (126 MHz, Chloroform-*d*) *δ* 199.07, 173.81, 136.85, 133.08, 128.60, 127.97, 63.52, 34.89, 34.26, 31.31, 24.64, 23.30, 22.28, 13.86. HRMS calculated for C_16_H_22_O_3_ (M + Na)^+^ 285.1461, found 285.1469.

4-oxo-4-(o-tolyl)butyl hexanoate (**4b**): Yellow liquid, yield 43%. ^1^H NMR (500 MHz, Chloroform-*d*) *δ* 7.65 (d, *J* = 8.0 Hz, 1H), 7.43–7.35 (m, 1H), 7.27 (d, *J* = 15.5 Hz, 2H), 4.17 (t, *J* = 6.5 Hz, 2H), 3.00 (t, *J* = 7.5 Hz, 2H), 2.51 (s, 3H), 2.30 (t, *J* = 7.5 Hz, 2H), 2.14–2.03 (m, 2H), 1.68–1.57 (m, 2H), 1.32–1.30 (m, 4H), 0.90 (t, *J* = 7.0 Hz, 3H); ^13^C NMR (126 MHz, Chloroform-*d*) *δ* 203.10, 173.83, 138.06, 137.82, 131.99, 131.29, 128.33, 125.67, 63.48, 37.76, 34.27, 31.31, 24.65, 23.41, 22.29, 21.27, 13.87. HRMS calculated for C_17_H_24_O_3_ (M + Na)^+^ 299.1618. found 299.1620.

4-oxo-4-(m-tolyl)butyl hexanoate (**4c**): Yellow liquid, yield 52%. ^1^H NMR (500 MHz, Chloroform-*d*) *δ* 7.79–7.74 (m, 2H), 7.42–7.33 (m, 2H), 4.19 (t, *J* = 6.5 Hz, 2H), 3.06 (t, *J* = 7.5 Hz, 2H), 2.43 (s, 3H), 2.30 (t, *J* = 7.5 Hz, 2H), 2.11 (p, *J* = 7.0 Hz, 2H), 1.63 (p, *J* = 7.5 Hz, 2H), 1.33–1.29 (m, 4H), 0.90 (t, *J* = 7.0 Hz, 3H); ^13^C NMR (126 MHz, Chloroform-*d*) *δ* 199.34, 173.86, 138.44, 136.93, 133.87, 128.53, 128.50, 125.24, 63.58, 34.97, 34.31, 31.35, 24.68, 23.37, 22.33, 21.38, 13.91. HRMS calculated for C_17_H_24_O_3_ (M + Na)^+^ 299.1618, found 299.1621.

4-oxo-4-(p-tolyl)butyl hexanoate (**4d**): Colorless liquid, yield 67%. ^1^H NMR (500 MHz, Chloroform-*d*) *δ* 7.87 (d, *J* = 8.5 Hz, 2H), 7.29–7.27 (m, 2H), 4.18 (t, *J* = 6.0 Hz, 2H), 3.05 (t, *J* = 7.0 Hz, 2H), 2.43 (s, 3H), 2.30 (t, *J* = 7.5 Hz, 2H), 2.16–2.05 (m, 2H), 1.65–1.61 (m, 2H), 1.33–1.29 (m, 4H), 0.90 (t, *J* = 7.0 Hz, 3H); ^13^C NMR (126 MHz, Chloroform-*d*) *δ* 198.73, 173.82, 143.86, 134.39, 129.26, 128.10, 63.57, 34.76, 34.27, 31.31, 24.64, 23.38, 22.29, 21.60, 13.87. HRMS for C_17_H_24_O_3_ (M + Na)^+^ 299.1618, found 299.1623.

4-(4-ethylphenyl)-4-oxobutyl hexanoate (**4e**): Colorless liquid, yield 61%. ^1^H NMR (500 MHz, Chloroform-*d*) *δ* 7.90 (d, *J* = 8.5 Hz, 2H), 7.30 (d, *J* = 8.0 Hz, 2H), 4.18 (t, *J* = 6.0 Hz, 2H), 3.05 (t, *J* = 7.0 Hz, 2H), 2.72 (q, *J* = 7.5 Hz, 2H), 2.30 (t, *J* = 7.5 Hz, 2H), 2.16–2.05 (m, 2H), 1.66–1.61 (m, 2H), 1.33–1.29 (m, 4H), 1.29–1.26 (m, 3H), 0.90 (t, *J* = 7.0 Hz, 3H); ^13^C NMR (126 MHz, Chloroform-*d*) *δ* 198.76, 173.82, 150.05, 134.60, 128.09, 63.58, 34.77, 34.27, 31.31, 28.92, 24.64, 23.38, 22.29, 15.16, 13.87. HRMS calculated for C_18_H_26_O_3_ (M + Na)^+^ 313.1774, found 313.1777.

5-(2,3-dimethylphenyl)-5-oxopentan-2-yl hexanoate (**4f**): Yellow liquid, yield 55%. ^1^H NMR (500 MHz, Chloroform-*d*) *δ* 7.32 (d, *J* = 7.5 Hz, 1H), 7.26 (d, *J* = 7.5 Hz, 1H), 7.16 (t, *J* = 8.0 Hz, 1H), 4.17 (t, *J* = 6.5 Hz, 2H), 2.94 (t, *J* = 7.0 Hz, 2H), 2.32 (s, 3H), 2.31 (s, 3H), 2.31–2.28 (m, 2H), 2.13–2.01 (m, 2H), 1.69–1.59 (m, 2H), 1.36–1.28 (m, 4H), 0.90 (t, *J* = 7.0 Hz, 3H); ^13^C NMR (126 MHz, Chloroform-*d*) *δ* 205.32, 173.81, 140.03, 138.28, 134.82, 132.22, 125.22, 124.87, 63.43, 38.90, 34.26, 31.31, 24.64, 23.38, 22.29, 20.36, 16.38, 13.86. HRMS calculated for C_18_H_26_O_3_ (M + Na)^+^ 313.1774, found 313.1775.

4-(3,4-dimethylphenyl)-4-oxobutyl hexanoate (**4g**): Colorless liquid, yield 35%. ^1^H NMR (500 MHz, Chloroform-*d*) *δ* 7.75 (s, 1H), 7.70 (dd, *J* = 80, 2.0 Hz, 1H), 7.23 (d, *J* = 7.5 Hz, 1H), 4.18 (t, *J* = 6.5 Hz, 2H), 3.04 (t, *J* = 7.5 Hz, 2H), 2.33 (s, 6H), 2.32–2.28 (m, 2H), 2.14–2.05 (m, 2H), 1.66–1.59 (m, 2H), 1.33–1.29 (m, 4H), 0.90 (t, *J* = 7.0 Hz, 3H); ^13^C NMR (126 MHz, Chloroform-*d*) *δ* 199.02, 173.83, 142.57, 136.92, 134.80, 129.80, 129.12, 125.72, 63.60, 34.76, 34.28, 31.32, 24.64, 23.42, 22.29, 19.97, 19.75, 13.87. HRMS calculated for C_18_H_26_O_3_ (M + Na)^+^ 313.1774, found: 313.1778.

4-(3,5-dimethylphenyl)-4-oxobutyl hexanoate (**4h**): Colorless liquid, yield 59%. ^1^H NMR (500 MHz, Chloroform-*d*) *δ* 7.58 (s, 2H), 7.22 (s, 1H), 4.18 (t, *J* = 6.0 Hz, 2H), 3.05 (t, *J* = 7.5 Hz, 2H), 2.39 (s, 6H), 2.30 (t, *J* = 7.5 Hz, 2H), 2.16–2.03 (m, 2H), 1.68–1.58 (m, 2H), 1.36–1.28 (m, 4H), 0.90 (t, *J* = 7.0 Hz, 3H); ^13^C NMR (126 MHz, Chloroform-*d*) *δ* 199.52, 173.84, 138.22, 136.98, 134.70, 125.78, 63.58, 34.97, 34.28, 31.31, 24.64, 23.37, 22.29, 21.21, 13.87. HRMS calculated for C_18_H_26_O_3_ (M + Na)^+^ 313.1774, found 313.1780.

4-(4-(tert-butyl)phenyl)-4-oxobutyl hexanoate (**4i**): Colorless liquid, yield 70%. ^1^H NMR (500 MHz, Chloroform-*d*) *δ* 7.92 (d, *J* = 8.5 Hz, 2H), 7.49 (d, *J* = 8.5 Hz, 2H), 4.18 (t, *J* = 6.5 Hz, 2H), 3.05 (t, *J* = 7.5 Hz, 2H), 2.30 (t, *J* = 7.5 Hz, 2H), 2.10 (p, *J* = 7.0 Hz, 2H), 1.69–1.57 (m, 2H), 1.36 (s, 9H), 1.31 (m, 4H), 0.90 (t, *J* = 6.5 Hz, 3H); ^13^C NMR (126 MHz, Chloroform-*d*) *δ* 198.77, 173.83, 156.84, 134.28, 127.95, 125.53, 63.58, 35.10, 34.76, 34.27, 31.31, 31.07, 24.64, 23.38, 22.29, 13.87. HRMS calculated for C_19_H_28_O_3_ (M + Na)^+^ 341.2087, found 341.2088.

4-(4-isobutylphenyl)-4-oxobutyl hexanoate (**4j**): Colorless liquid, yield 65%. ^1^H NMR (500 MHz, Chloroform-*d*) *δ* 7.89 (d, *J* = 8.0 Hz, 2H), 7.24 (d, *J* = 8.5 Hz, 2H), 4.18 (t, *J* = 6.5 Hz, 2H), 3.05 (t, *J* = 7.0 Hz, 2H), 2.54 (d, *J* = 7.0 Hz, 2H), 2.30 (t, *J* = 7.5 Hz, 2H), 2.17–2.04 (m, 2H), 2.00–1.81 (m, 1H), 1.67–1.55 (m, 2H), 1.34–1.29 (m, 4H), 0.92 (d, *J* = 7.0 Hz, 6H), 0.91–0.87 (m, 3H); ^13^C NMR (126 MHz, Chloroform-*d*) *δ* 198.76, 173.79, 147.57, 134.65, 129.30, 127.96, 63.57, 45.37, 34.76, 34.26, 31.31, 30.08, 24.64, 23.38, 22.30, 13.86. HRMS calculated for C_19_H_28_O_3_ (M + Na)^+^ 341.2087, found 341.2093.

4-(3-methoxyphenyl)-4-oxobutyl hexanoate (**4k**): Yellow liquid, yield 52%. ^1^H NMR (500 MHz, Chloroform-*d*) *δ* 7.55 (m, 1H), 7.52–7.49 (m, 1H), 7.39 (t, *J* = 8.0 Hz, 1H), 7.15–7.10 (m, 1H), 4.19 (t, *J* = 6.0 Hz, 2H), 3.87 (s, 3H), 3.06 (t, *J* = 7.5 Hz, 2H), 2.30 (t, *J* = 7.5 Hz, 2H), 2.16–2.04 (m, 2H), 1.69–1.56 (m, 2H), 1.34–1.29 (m, 4H), 0.90 (t, *J* = 7.0 Hz, 3H); ^13^C NMR (126 MHz, Chloroform-*d*) *δ* 198.92, 173.83, 159.87, 138.21, 129.58, 120.60, 119.50, 112.35, 63.50, 55.43, 35.01, 34.26, 31.31, 24.64, 23.35, 22.29. HRMS calculated for C_17_H_24_O_4_ (M + Na)^+^ 315.1567, found 315.1576.

4-(4-methoxyphenyl)-4-oxobutyl hexanoate (**4l**): Colorless liquid, yield 56%. ^1^H NMR (500 MHz, Chloroform-*d*) *δ* 7.96 (d, *J* = 9.0 Hz, 2H), 6.95 (d, *J* = 8.5 Hz, 2H), 4.18 (t, *J* = 6.0 Hz, 2H), 3.89 (s, 3H), 3.02 (t, *J* = 7.0 Hz, 2H), 2.30 (t, *J* = 7.5 Hz, 2H), 2.17–2.04 (m, 2H), 1.66–1.57 (m, 2H), 1.34–1.28 (m, 4H), 0.90 (t, *J* = 6.5 Hz, 3H); ^13^C NMR (126 MHz, Chloroform-*d*) *δ* 197.64, 173.83, 163.49, 130.23, 129.96, 113.73, 63.62, 55.45, 34.51, 34.28, 31.32, 24.65, 23.47, 22.30, 13.88. HRMS calculated for C_17_H_24_O_4_ (M + Na)^+^ 315.1567, found 315.1577.

4-(2-ethoxyphenyl)-4-oxobutyl hexanoate (**4m**): Yellow liquid, yield 46%. ^1^H NMR (500 MHz, Chloroform-*d*) *δ* 7.71 (dd, *J* = 7.5, 2.0 Hz, 1H), 7.49–7.38 (m, 1H), 7.00 (t, *J* = 7.5 Hz, 1H), 6.95 (d, *J* = 8.5 Hz, 1H), 4.19–4.12 (m, 4H), 3.10 (t, *J* = 7.5 Hz, 2H), 2.29 (t, *J* = 7.5 Hz, 2H), 2.06 (p, *J* = 6.5 Hz, 2H), 1.67–1.60 (m, 2H), 1.49 (t, *J* = 7.0 Hz, 3H), 1.34–1.29 (m, 4H), 0.90 (t, *J* = 7.0 Hz, 3H); ^13^C NMR (126 MHz, Chloroform-*d*) *δ* 201.63, 173.86, 157.95, 133.39, 130.33, 128.32, 120.55, 112.29, 64.07, 63.86, 40.30, 34.29, 31.31, 24.65, 23.58, 22.28, 14.75, 13.86. HRMS calculated for C_18_H_26_O_4_ (M + Na)^+^ 329.1723, found 329.1730.

4-(3-ethoxyphenyl)-4-oxobutyl hexanoate (**4n**): Yellow liquid, yield 57%. ^1^H NMR (500 MHz, Chloroform-*d*) *δ* 7.56–7.51 (m, 1H), 7.50–7.48 (m, 1H), 7.37 (t, *J* = 8.0 Hz, 1H), 7.14–7.08 (m, 1H), 4.18 (t, *J* = 6.0 Hz, 2H), 4.10 (q, *J* = 7.0 Hz, 2H), 3.05 (t, *J* = 7.0 Hz, 2H), 2.30 (t, *J* = 7.5 Hz, 2H), 2.16–2.05 (m, 2H), 1.66–1.58 (m, 2H), 1.45 (t, *J* = 7.0 Hz, 3H), 1.35–1.28 (m, 4H), 0.90 (t, *J* = 7.0 Hz, 3H); ^13^C NMR (126 MHz, Chloroform-*d*) *δ* 197.94, 172.82, 158.21, 137.17, 128.55, 119.43, 118.88, 112.09, 62.70, 62.51, 34.00, 33.26, 30.31, 23.64, 22.35, 21.29, 13.73, 12.87. HRMS calculated for C_18_H_26_O_4_ (M + Na)^+^ 329.1723, found 329.1726.

4-(4-ethoxyphenyl)-4-oxobutyl hexanoate (**4o**): Colorless liquid, yield 60%. ^1^H NMR (500 MHz, Chloroform-*d*) *δ* 7.94 (d, *J* = 9.0 Hz, 2H), 6.93 (d, *J* = 9.0 Hz, 2H), 4.18 (t, *J* = 6.5 Hz, 2H), 4.11 (q, *J* = 7.0 Hz, 2H), 3.02 (t, *J* = 7.5 Hz, 2H), 2.30 (t, *J* = 7.5 Hz, 2H), 2.16–2.01 (m, 2H), 1.68–1.55 (m, 2H), 1.46 (t, *J* = 7.0 Hz, 3H), 1.34–1.28 (m, 4H), 0.90 (t, *J* = 7.0 Hz, 3H); ^13^C NMR (126 MHz, Chloroform-*d*) *δ* 197.66, 173.83, 162.92, 130.23, 129.77, 114.17, 63.74, 63.63, 34.48, 34.27, 31.31, 24.64, 23.48, 22.29, 14.64, 13.87. HRMS calculated for C_18_H_26_O_4_ (M + Na)^+^ 329.1723, found 329.1729.

4-(4-isopropoxyphenyl)-4-oxobutyl hexanoate (**4p**): Colorless liquid, yield 63%. ^1^H NMR (500 MHz, Chloroform-*d*) *δ* 7.93 (d, *J* = 8.5 Hz, 2H), 6.91 (d, *J* = 9.0 Hz, 2H), 4.66 (p, *J* = 6.0 Hz, 1H), 4.18 (t, *J* = 6.5 Hz, 2H), 3.01 (t, *J* = 7.5 Hz, 2H), 2.29 (t, *J* = 7.5 Hz, 2H), 2.17–1.99 (m, 2H), 1.70–1.54 (m, 2H), 1.38 (d, *J* = 6.0 Hz, 6H), 1.33–1.29 (m, 4H), 1.26 (s, 3H); ^13^C NMR (126 MHz, Chloroform-*d*) *δ* 197.64, 173.84, 162.01, 130.26, 129.51, 115.11, 70.11, 63.64, 34.45, 34.27, 31.31, 24.64, 23.49, 22.29, 21.89, 13.87. HRMS calculated for C_19_H_28_O_4_ (M + Na)^+^ 343.1880, found 343.1883.

4-oxo-4-(3-phenoxyphenyl)butyl hexanoate (**4q**): Yellow liquid, yield 57%. ^1^H NMR (500 MHz, Chloroform-*d*) *δ* 7.72–7.67 (m, 1H), 7.62–7.58 (m, 1H), 7.44 (t, *J* = 8.0 Hz, 1H), 7.41–7.34 (m, 2H), 7.25–7.19 (m, 1H), 7.19–7.14 (m, 1H), 7.05–7.01 (m, 2H), 4.17 (t, *J* = 6.0 Hz, 2H), 3.04 (t, *J* = 7.5 Hz, 2H), 2.29 (t, *J* = 7.5 Hz, 2H), 2.17–2.01 (m, 2H), 1.67–1.55 (m, 2H), 1.34–1.29 (m, 4H), 0.90 (t, *J* = 7.0 Hz, 3H); ^13^C NMR (126 MHz, Chloroform-*d*) *δ* 198.42, 173.80, 157.81, 156.59, 138.61, 129.94, 123.84, 123.27, 122.68, 119.10, 117.88, 63.43, 35.04, 34.24, 31.31, 24.63, 23.25, 22.28, 13.87. HRMS calculated for C_22_H_26_O_4_ (M + Na)^+^ 377.1723 found 377.1732.

4-(3-fluorophenyl)-4-oxobutyl hexanoate (**4r**): Yellow liquid, yield 47%. ^1^H NMR (500 MHz, Chloroform-*d*) *δ* 7.77–7.74 (m, 1H), 7.68–7.63 (m, 1H), 7.51–7.43 (m, 1H), 7.31–7.28 (m, 1H), 4.19 (t, *J* = 6.0 Hz, 2H), 3.06 (t, *J* = 7.0 Hz, 2H), 2.30 (t, *J* = 7.5 Hz, 2H), 2.17–2.03 (m, 2H), 1.66–1.57 (m, 2H), 1.33–1.30 (m, 4H), 0.90 (t, *J* = 7.0 Hz, 3H); ^13^C NMR (126 MHz, Chloroform-*d*) *δ* 196.77, 172.80, 161.89 (d, *J* = 248.1 Hz), 137.91 (d, *J* = 6.0 Hz), 129.28 (d, *J* = 7.6 Hz), 122.70 (d, *J* = 2.8 Hz), 119.10 (d, *J* = 21.5 Hz), 113.75 (d, *J* = 22.3 Hz), 62.36, 34.08, 33.24, 30.31, 23.63, 22.19, 21.28, 12.86. HRMS calculated for C_16_H_21_FO_3_ (M + Na)^+^ 303.1367 found 303.1375.

4-(4-chlorophenyl)-4-oxobutyl hexanoate (**4s**): Colorless liquid, yield 49%. ^1^H NMR (500 MHz, Chloroform-*d*) *δ* 7.91 (d, *J* = 8.5 Hz, 2H), 7.45 (d, *J* = 8.5 Hz, 2H), 4.18 (t, *J* = 6.5 Hz, 2H), 3.04 (t, *J* = 7.0 Hz, 2H), 2.29 (t, *J* = 7.5 Hz, 2H), 2.17–2.03 (m, 2H), 1.68–1.55 (m, 2H), 1.34–1.28 (m, 4H), 0.90 (t, *J* = 7.0 Hz, 3H); ^13^C NMR (126 MHz, Chloroform-*d*) *δ* 197.81, 173.80, 139.57, 135.13, 129.38, 128.93, 63.40, 34.89, 34.24, 31.30, 24.63, 23.21, 22.28, 13.87. HRMS calculated for C_16_H_21_ClO_3_ (M + Na)^+^ 319.1071, found 319.1078.

4-(3-bromophenyl)-4-oxobutyl hexanoate (**4t**): Yellow liquid, yield 42%. ^1^H NMR (500 MHz, DMSO-*d*_6_) *δ* 8.10 (t, *J* = 1.5 Hz, 1H), 7.92–7.87 (m, 1H), 7.75–7.68 (m, 1H), 7.37 (t, *J* = 8.0 Hz, 1H), 4.18 (t, *J* = 6.5 Hz, 2H), 3.05 (t, *J* = 7.0 Hz, 2H), 2.30 (t, *J* = 7.5 Hz, 2H), 2.11 (p, *J* = 6.5 Hz, 2H), 1.67–1.59 (m, 2H), 1.33–1.30 (m, 4H), 0.90 (t, *J* = 7.0 Hz, 3H); ^13^C NMR (126 MHz, Chloroform-*d*) *δ* 197.67, 173.80, 138.55, 135.95, 131.10, 130.21, 126.48, 123.01, 63.34, 35.01, 34.25, 31.31, 24.63, 23.17, 22.29, 13.88. HRMS calculated for C_16_H_21_BrO_3_ (M + Na)^+^ 363.0566, found 363.0571.

4-(4-bromophenyl)-4-oxobutyl hexanoate (**4u**): Colorless liquid, yield 55%. ^1^H NMR (500 MHz, Chloroform-*d*) *δ* 7.84 (d, *J* = 8.5 Hz, 2H), 7.62 (d, *J* = 8.5 Hz, 2H), 4.18 (t, *J* = 6.5 Hz, 2H), 3.04 (t, *J* = 7.5 Hz, 2H), 2.29 (t, *J* = 7.5 Hz, 2H), 2.17–2.02 (m, 2H), 1.67–1.55 (m, 2H), 1.33–1.29 (m, 4H), 0.90 (t, *J* = 7.0 Hz, 3H); ^13^C NMR (126 MHz, Chloroform-*d*) *δ* 197.99, 173.79, 135.53, 131.93, 129.49, 128.27, 63.38, 34.87, 34.24, 31.31, 24.63, 23.20, 22.29, 13.87. HRMS calculated for C_16_H_21_BrO_3_ (M + Na)^+^ 363.0566, found 363.0570.

4-oxo-4-phenylbutyl 3-oxobutanoate (**5a**): Colorless liquid, yield 42%. ^1^H NMR (500 MHz, Chloroform-*d*) *δ* 8.06–7.90 (m, 2H), 7.62–7.55 (m, 1H), 7.52–7.45 (m, 2H), 4.28 (t, *J* = 6.0 Hz, 2H), 3.47 (s, 2H), 3.11 (t, *J* = 7.0 Hz, 2H), 2.28 (s, 3H), 2.19–2.08 (m, 2H); ^13^C NMR (126 MHz, Chloroform-*d*) *δ* 200.43, 198.96, 167.04, 136.77, 133.14, 128.63, 127.99, 64.66, 50.02, 34.66, 30.17, 23.08. HRMS calculated for C_14_H_16_O_4_ (M + Na)^+^ 271.0941 found 271.0948.

4-oxo-4-(m-tolyl)butyl 3-oxobutanoate (**5b**): Colorless liquid, yield 41%. ^1^H NMR (500 MHz, Chloroform-*d*) *δ* 7.87–7.68 (m, 2H), 7.48–7.32 (m, 2H), 4.27 (t, *J* = 6.5 Hz, 2H), 3.47 (s, 2H), 3.08 (t, *J* = 7.0 Hz, 2H), 2.43 (s, 3H), 2.28 (s, 3H), 2.13 (p, *J* = 6.5 Hz, 2H); ^13^C NMR (126 MHz, Chloroform-*d*) *δ* 200.49, 199.21, 167.07, 138.43, 136.80, 133.91, 128.51, 128.50, 125.22, 64.70, 50.02, 34.71, 30.17, 23.12, 21.33. HRMS calculated for C_15_H_18_O_4_ (M + Na)^+^ 285.1097 found 285.1104.

4-oxo-4-(p-tolyl)butyl 3-oxobutanoate (**5c**): Colorless liquid, yield 46%. ^1^H NMR (500 MHz, Chloroform-*d*) *δ* 7.88 (d, *J* = 8.0 Hz, 2H), 7.28 (d, *J* = 3.0 Hz, 2H), 4.27 (t, *J* = 6.5 Hz, 2H), 3.47 (s, 2H), 3.07 (t, *J* = 7.0 Hz, 2H), 2.42 (s, 3H), 2.27 (s, 3H), 2.18–2.06 (m, 2H); ^13^C NMR (126 MHz, Chloroform-*d*) *δ* 200.48, 198.64, 167.06, 143.94, 134.30, 129.30, 128.12, 64.73, 50.02, 34.53, 30.16, 23.16, 21.61. HRMS for C_15_H_18_O_4_ (M + Na)^+^ 285.1097, found 285.1104.

4-(3,5-dimethylphenyl)-4-oxobutyl 3-oxobutanoate (**5d**): Colorless liquid, yield 42%. ^1^H NMR (500 MHz, Chloroform-*d*) *δ* 7.58 (s, 2H), 7.22 (s, 1H), 4.27 (t, *J* = 6.5 Hz, 2H), 3.47 (s, 2H), 3.06 (t, *J* = 7.5 Hz, 2H), 2.39 (s, 6H), 2.28 (s, 3H), 2.18–2.03 (m, 2H); ^13^C NMR (126 MHz, Chloroform-*d*) *δ* 200.49, 199.42, 167.08, 138.26, 136.89, 134.78, 127.84, 125.80, 64.74, 50.02, 34.75, 30.17, 23.16, 21.22. HRMS calculated for C_16_H_20_O_4_ (M + Na)^+^ 299.1254, found 299.1259.

4-(4-ethylphenyl)-4-oxobutyl 3-oxobutanoate (**5e**): Colorless liquid, yield 49%. ^1^H NMR (500 MHz, Chloroform-*d*) *δ* 7.91 (d, *J* = 8.0 Hz, 2H), 7.30 (d, *J* = 8.5 Hz, 2H), 4.27 (t, *J* = 6.5 Hz, 2H), 3.47 (s, 2H), 3.07 (t, *J* = 7.0 Hz, 2H), 2.72 (q, *J* = 7.5 Hz, 2H), 2.28 (s, 3H), 2.13 (p, *J* = 7.0 Hz, 2H), 1.27 (t, *J* = 7.5 Hz, 3H); ^13^C NMR (126 MHz, Chloroform-*d*) *δ* 200.50, 198.68, 167.07, 150.13, 134.51, 130.29, 128.23, 128.12, 64.74, 50.02, 34.55, 30.16, 28.92, 23.16, 15.16. HRMS calculated for C_16_H_20_O_4_ (M + Na)^+^ 299.1254, found 299.1261.

5-(4-isopropylphenyl)-5-oxopentan-2-yl 3-oxobutanoate (**5f**): Colorless liquid, yield 50%. ^1^H NMR (500 MHz, Chloroform-*d*) *δ* 7.92 (d, *J* = 8.5 Hz, 2H), 7.33 (d, *J* = 8.5 Hz, 2H), 4.27 (t, *J* = 7.0 Hz, 2H), 3.47 (s, 2H), 3.07 (t, *J* = 7.0 Hz, 2H), 3.02–2.89 (m, 1H), 2.28 (s, 3H), 2.15–2.07 (m, 2H), 1.28 (d, *J* = 7.0 Hz, 6H); ^13^C NMR (126 MHz, Chloroform-*d*) *δ* 200.50, 198.66, 167.07, 154.69, 134.65, 130.33, 128.26, 126.71, 126.57, 64.74, 50.02, 34.54, 34.24, 30.17, 23.64, 23.16. HRMS calculated for C_17_H_22_O_4_ (M + Na)^+^ 313.1410 found 313.1418.

4-(4-(tert-butyl)phenyl)-4-oxobutyl 3-oxobutanoate (**5g**): Colorless liquid, yield 59%. ^1^H NMR (500 MHz, Chloroform-*d*) *δ* 7.92 (d, *J* = 8.5 Hz, 2H), 7.50 (d, *J* = 8.5 Hz, 2H), 4.27 (t, *J* = 6.0 Hz, 2H), 3.47 (s, 2H), 3.08 (t, *J* = 7.0 Hz, 2H), 2.28 (s, 3H), 2.13 (p, *J* = 6.5 Hz, 2H), 1.36 (s, 9H); ^13^C NMR (126 MHz, Chloroform-*d*) *δ* 200.50, 198.68, 167.07, 156.92, 134.20, 127.98, 125.57, 64.74, 50.02, 35.10, 34.54, 31.07, 30.17, 23.16. HRMS calculated for C_18_H_24_O_4_ (M + Na)^+^ 327.1567 found 327.1569.

4-(4-methoxyphenyl)-4-oxobutyl 3-oxobutanoate (**5h**): Colorless liquid, yield 55%. ^1^H NMR (500 MHz, Chloroform-*d*) *δ* 7.96 (d, *J* = 8.5 Hz, 2H), 6.94 (d, *J* = 9.0 Hz, 2H), 4.26 (t, *J* = 6.5 Hz, 2H), 3.88 (s, 3H), 3.46 (s, 2H), 3.04 (t, *J* = 7.0 Hz, 2H), 2.27 (s, 3H), 2.11 (p, *J* = 7.0 Hz, 2H); ^13^C NMR (126 MHz, Chloroform-*d*) *δ* 200.54, 197.57, 167.07, 163.53, 132.24, 130.27, 113.76, 64.77, 55.46, 50.02, 34.27, 30.17, 23.24. HRMS calculated for C_15_H_18_O_5_ (M + Na)^+^ 301.1046 found 301.1053.

4-(4-ethoxyphenyl)-4-oxobutyl 3-oxobutanoate (**5i**): Colorless liquid, yield 43%. ^1^H NMR (500 MHz, Chloroform-*d*) *δ* 7.95 (d, *J* = 9.0 Hz, 2H), 6.93 (d, *J* = 9.0 Hz, 2H), 4.27 (t, *J* = 6.0 Hz, 2H), 4.11 (q, *J* = 7.0 Hz, 2H), 3.47 (s, 2H), 3.04 (t, *J* = 7.0 Hz, 2H), 2.28 (s, 3H), 2.19–2.06 (m, 2H), 1.46 (t, *J* = 7.0 Hz, 3H); ^13^C NMR (126 MHz, Chloroform-*d*) *δ* 200.50, 197.54, 167.06, 162.96, 130.26, 129.68, 114.20, 64.79, 63.75, 50.03, 34.25, 30.17, 23.25, 14.65. HRMS calculated for C_16_H_20_O_5_ (M + Na)^+^ 315.1203 found 315.1208.

4-(4-isopropoxyphenyl)-4-oxobutyl 3-oxobutanoate (**5j**): Colorless liquid, yield 58%. ^1^H NMR (500 MHz, Chloroform-*d*) *δ* 7.94 (d, *J* = 9.0 Hz, 2H), 6.92 (d, *J* = 9.0 Hz, 2H), 4.71–4.58 (m, 1H), 4.26 (t, *J* = 6.5 Hz, 2H), 3.47 (s, 2H), 3.03 (t, *J* = 7.5 Hz, 2H), 2.28 (s, 3H), 2.11 (p, 2H), 1.38 (d, *J* = 6.5 Hz, 6H); ^13^C NMR (126 MHz, Chloroform-*d*) *δ* 200.51, 197.52, 167.08, 162.09, 130.32, 129.47, 115.17, 70.15, 64.83, 50.06, 34.25, 30.19, 23.30, 21.92. HRMS calculated for C_17_H_22_O_5_ (M + Na)^+^ 329.1359, found 329.1365.

4-oxo-4-(3-phenoxyphenyl)butyl 3-oxobutanoate (**5k**): Colorless liquid, yield 40%. ^1^H NMR (500 MHz, Chloroform-*d*) *δ* 7.71 (d, *J* = 7.5 Hz, 1H), 7.60 (t, *J* = 2.0 Hz, 1H), 7.45 (t, *J* = 8.0 Hz, 1H), 7.40–7.34 (m, 2H), 7.22 (dd, *J* = 8.0, 2.5 Hz, 1H), 7.16 (t, *J* = 7.5 Hz, 1H), 7.03 (d, *J* = 7.5 Hz, 2H), 4.26 (t, *J* = 6.5 Hz, 2H), 3.46 (s, 2H), 3.06 (t, *J* = 7.0 Hz, 2H), 2.27 (s, 3H), 2.15–2.08 (m, 2H); ^13^C NMR (126 MHz, Chloroform-*d*) *δ* 200.38, 198.31, 167.02, 157.82, 156.59, 138.54, 129.94, 123.83, 123.34, 122.73, 119.12, 117.85, 64.57, 49.99, 34.82, 30.15, 23.04. HRMS calculated for C_20_H_20_O_5_ (M + Na)^+^ 363.1203, found 363.1209.

4-(3-fluorophenyl)-4-oxobutyl 3-oxobutanoate (**5l**): Colorless liquid, yield 28%. ^1^H NMR (500 MHz, Chloroform-*d*) *δ* 7.80–7.75 (m, 1H), 7.70–7.63 (m, 1H), 7.51–7.44 (m, 1H), 7.31–7.28 (m, 1H), 4.28 (t, *J* = 6.5 Hz, 2H), 3.48 (s, 2H), 3.09 (t, *J* = 7.0 Hz, 2H), 2.28 (s, 3H), 2.14 (p, *J* = 7.0 Hz, 2H); ^13^C NMR (126 MHz, Chloroform-*d*) *δ* 200.43, 197.71, 167.02, 138.83 (d, *J* = 6.2 Hz), 130.31 (d, *J* = 7.6 Hz), 123.76 (d, *J* = 2.8 Hz), 120.16 (d, *J* = 21.4 Hz), 114.75 (d, *J* = 22.2 Hz), 99.64 (d, *J* = 87.8 Hz), 64.48, 50.02, 34.85, 30.20, 22.98. HRMS calculated for C_14_H_15_FO_4_ (M + Na)^+^ 289.0847, found 289.0854.

4-(4-chlorophenyl)-4-oxobutyl 3-oxobutanoate (**5m**): Colorless liquid, yield 33%. ^1^H NMR (500 MHz, Chloroform-*d*) *δ* 7.93 (d, *J* = 8.5 Hz, 2H), 7.46 (d, *J* = 9.0 Hz, 2H), 4.28 (t, *J* = 6.5 Hz, 2H), 3.48 (s, 2H), 3.08 (t, *J* = 7.5 Hz, 2H), 2.28 (s, 3H), 2.13 (p, *J* = 6.5 Hz, 2H); ^13^C NMR (126 MHz, Chloroform-*d*) *δ* 197.77, 167.02, 139.62, 135.06, 129.43, 128.96, 64.50, 50.03, 34.64, 30.22, 23.01. HRMS calculated for C_14_H_15_ClO_4_ (M + Na)^+^ 305.0551, found 305.0563.

4-(3-bromophenyl)-4-oxobutyl 3-oxobutanoate (**5n**): Colorless liquid, yield 31%. ^1^H NMR (500 MHz, Chloroform-*d*) *δ* 8.11 (t, *J* = 1.5 Hz, 1H), 7.94–7.88 (m, 1H), 7.75–7.66 (m, 1H), 7.37 (t, *J* = 7.5 Hz, 1H), 4.27 (t, *J* = 6.0 Hz, 2H), 3.48 (s, 2H), 3.08 (t, *J* = 7.0 Hz, 2H), 2.28 (s, 3H), 2.13 (p, *J* = 6.5 Hz, 2H); ^13^C NMR (126 MHz, Chloroform-*d*) *δ* 200.38, 197.58, 167.00, 138.49, 135.98, 131.08, 130.23, 126.54, 123.01, 64.44, 50.00, 34.77, 30.19, 22.98. HRMS calculated for C_14_H_15_BrO_4_ (M + Na)^+^ 349.0046, found 349.0056.

4-(4-bromophenyl)-4-oxobutyl 3-oxobutanoate (**5o**): Colorless liquid, yield 37%. ^1^H NMR (500 MHz, Chloroform-*d*) *δ* 7.85 (d, *J* = 8.5 Hz, 2H), 7.63 (d, *J* = 8.5 Hz, 2H), 4.28 (t, *J* = 6.0 Hz, 2H), 3.48 (s, 2H), 3.07 (t, *J* = 7.0 Hz, 2H), 2.28 (s, 3H), 2.13 (p, *J* = 6.5 Hz, 2H); ^13^C NMR (126 MHz, Chloroform-*d*) *δ* 200.43, 197.94, 167.00, 135.47, 131.95, 129.54, 128.33, 64.49, 50.02, 34.63, 30.20, 23.01. HRMS calculated for C_14_H_15_BrO_4_ (M + Na)^+^ 349.0046, found 349.0053.

## 4. Conclusions

In summary, we developed a simple method to prepare the new compound 1-cyano-4-oxo-4-phenylacetate by radical addition of benzaldehyde and 1-cyanoallyl acetate under the initiation of TBPEH and TBPB. Moreover, the addition reactions of benzaldehyde with different kinds of allyl esters, like allyl hexanoate and allyl acetoacetate, were studied, showing the same electronic effect and steric effect. Furthermore, a reasonable mechanism is proposed. This reaction is compatible with electron-donating and halogen groups, and a total of 61 new compounds (aromatic ketone esters) were synthesized in moderate yields under solvent-free and metal-free conditions, which can be further applied to the preparation of lactone, piperidine, tetrazole and oxazole. It also has excellent atomic utilization and chemical selectivity. Further studies on free radical addition to synthesizing various functionalized ketones are ongoing in our laboratory.

## Figures and Tables

**Table 1:** Effects of reaction conditions on the yields of compound **3a** ^a^.

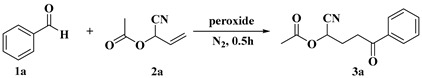				
Entry	Peroxide (equiv.)	Solvent	Temp (°C)	Yield (%) ^b^
1	TBHP (2)	-	130	<10
2	TBPB (2)	-	130	24
3	TBPEH (2)	-	120	32
4	TBPEH (2)	-	110	28
5	TBPEH (1) TBPB (1)	-	110	43
6	TBPEH (2) TBPB (2)	-	110	57
7	TBPEH (2)	DMF	120	0
8	TBPEH (2)	DMSO	120	0
9 ^c^	TBPEH (2)	-	120	26

^a^ Reaction conditions: benzaldehyde **1a** (0.4 mmol), 1-cyanoallyl acetate **2a** (0.2 mmol, 1 equiv.), N_2_, 0.5 h. ^b^ Isolated Yield. ^c^ benzaldehyde **1a** (0.3 mmol). TBHP = tert-butyl hydroperoxide; TBPB = tert-butyl peroxybenzoate; TBPEH = tert-butylperoxy-2-ethylhexanoate.

## Data Availability

Not applicable.

## Supplementary Materials

The following supporting information can be downloaded at: https://www.mdpi.com/article/10.3390/ijms232213704/s1. Figures S1–S51: ^1^H NMR spectra and ^13^C NMR spectra of **3a**–**3y**; Figures S52–S93: ^1^H NMR spectra and ^13^C NMR spectra of **4a**–**4u**; Figures S94–S123: ^1^H NMR spectra and ^13^C NMR spectra of **5a**–**5o**; Figures S124–S184: ESI-HRMS spectra of compounds **3**,**4**,**5**.

## Sample Availability

Samples of the compounds are not available from the authors.
