# Supplementary material for: TBPEH-TBPB Initiate the Radical Addition of Benzaldehyde and Allyl Esters
Source: Int J Mol Sci. 2022 Nov 8;23(22):13704. doi: 10.3390/ijms232213704 (PMC9690988; doi:10.3390/ijms232213704)
Supplement: Supplementary file 1 [file ijms-23-13704-s001.zip › ijms-2006944-supplementary.pdf]

Supporting Information

TBPO-TBPB Initiate the Radical Addition of Benzaldehyde and Allyl Esters

Bin-Long Sun, Xiao-Yu Tian, Sen Yang, Ying-Ying Wang, Ying-Ying Shao, Xin-Hao Fu, Wen-Yuan Wang, Min-Ting Tu, Yang Chen, Jun-Hui Wu, Chang-Yuan Wu, Cheng-Xia Tan *

1. ^{1}H and ^{13}C NMR spectra of compounds **3** and GC-MC of **6a** (S1–S51).
2. ^{1}H and ^{13}C NMR spectra of compounds **4** (S52-S93).
3. ^{1}H and ^{13}C NMR spectra of compounds **5** (S94-S123).
4. ESI-HRMS spectra of compounds **3** (S124-S148).
5. ESI-HRMS spectra of compounds **4** (S149-S169).
6. ESI-HRMS spectra of compounds **5** (S170-S184).

1. ^{1}H and ^{13}C NMR spectra of compounds **3** and GC-MC of **6a**.

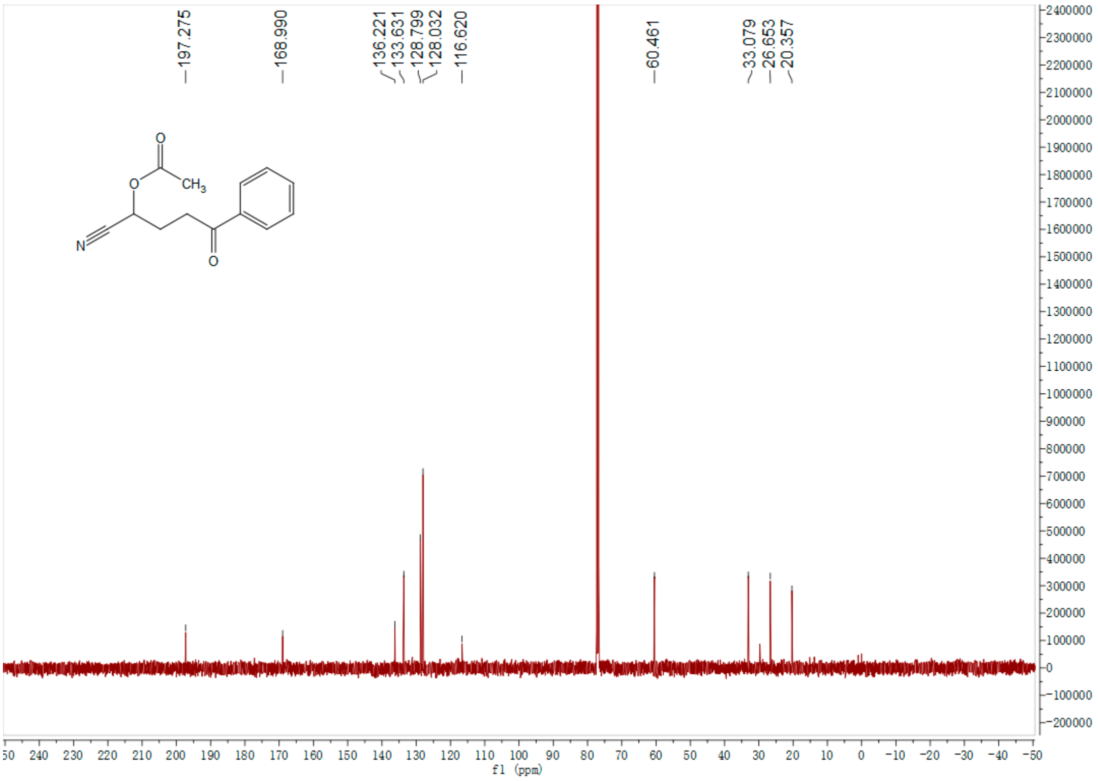

Figure S1 ^{13}C NMR spectra of **3a**

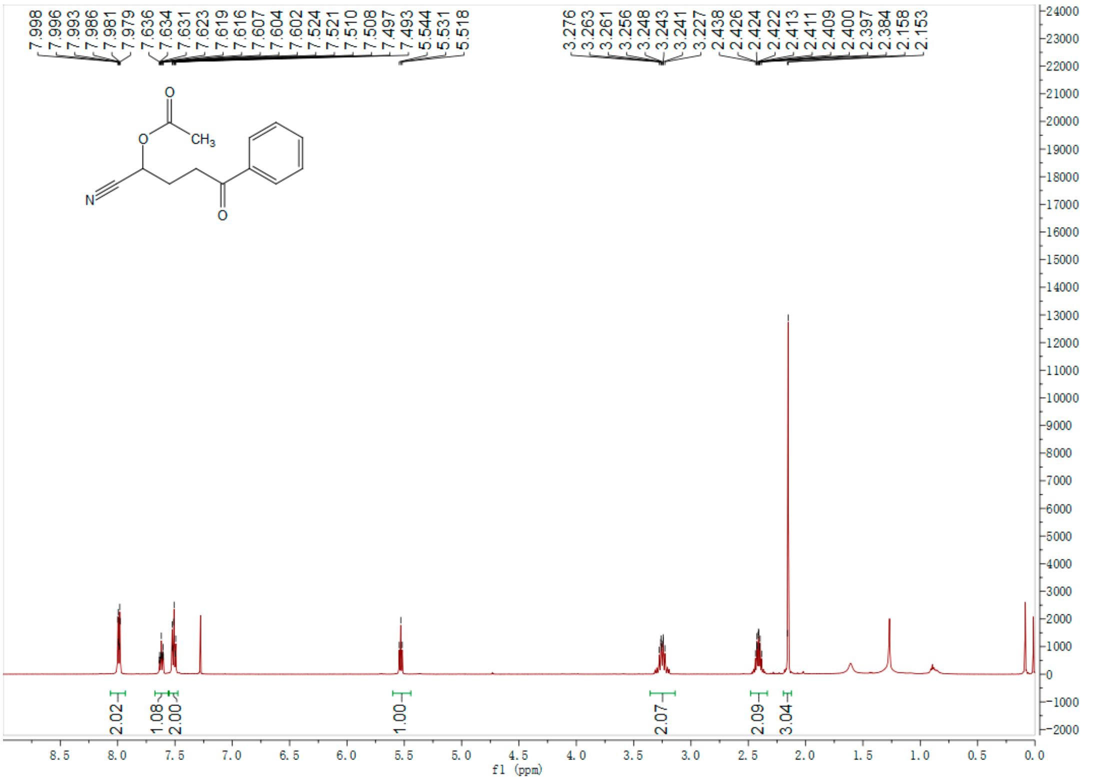

Figure S2 ^{1}H NMR spectra of **3a**

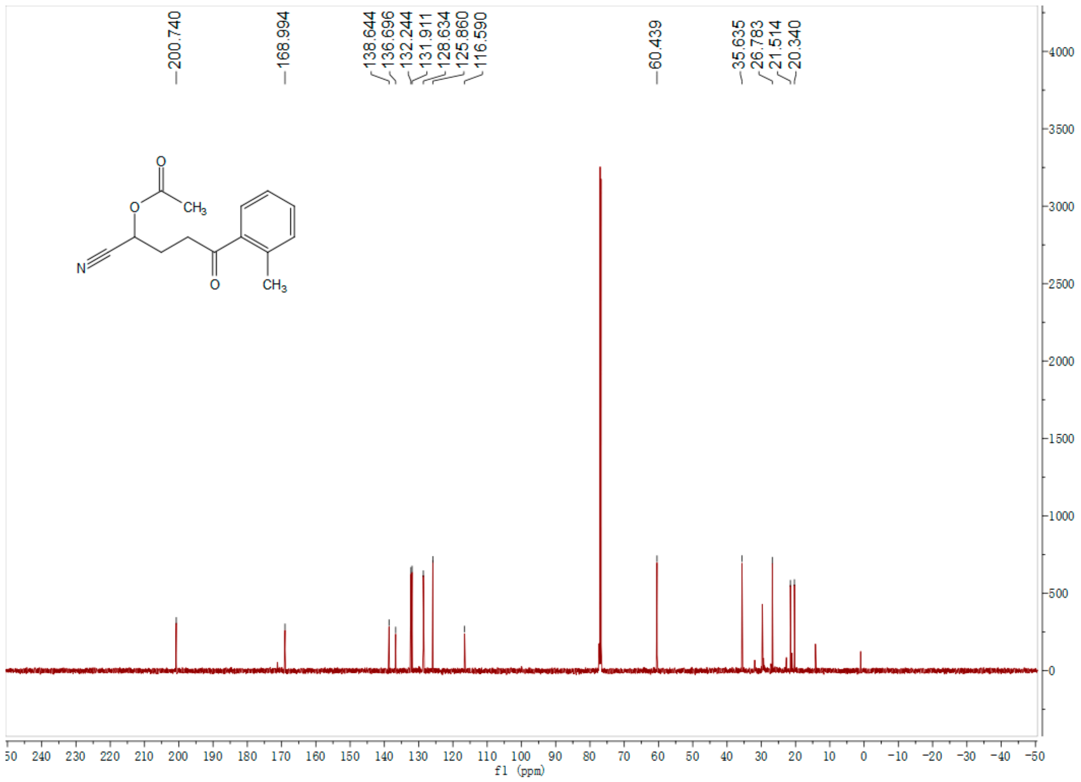

Figure S3 ^{13}C NMR spectra of **3b**

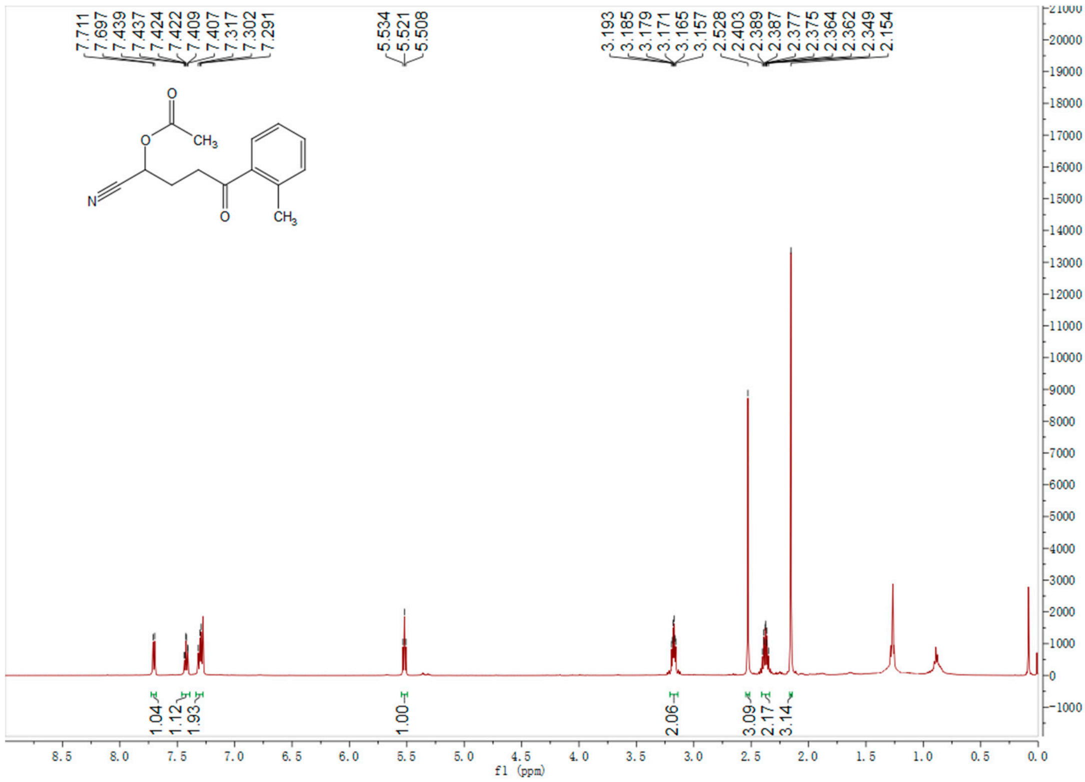

Figure S4 ^{1}H NMR spectra of **3b**

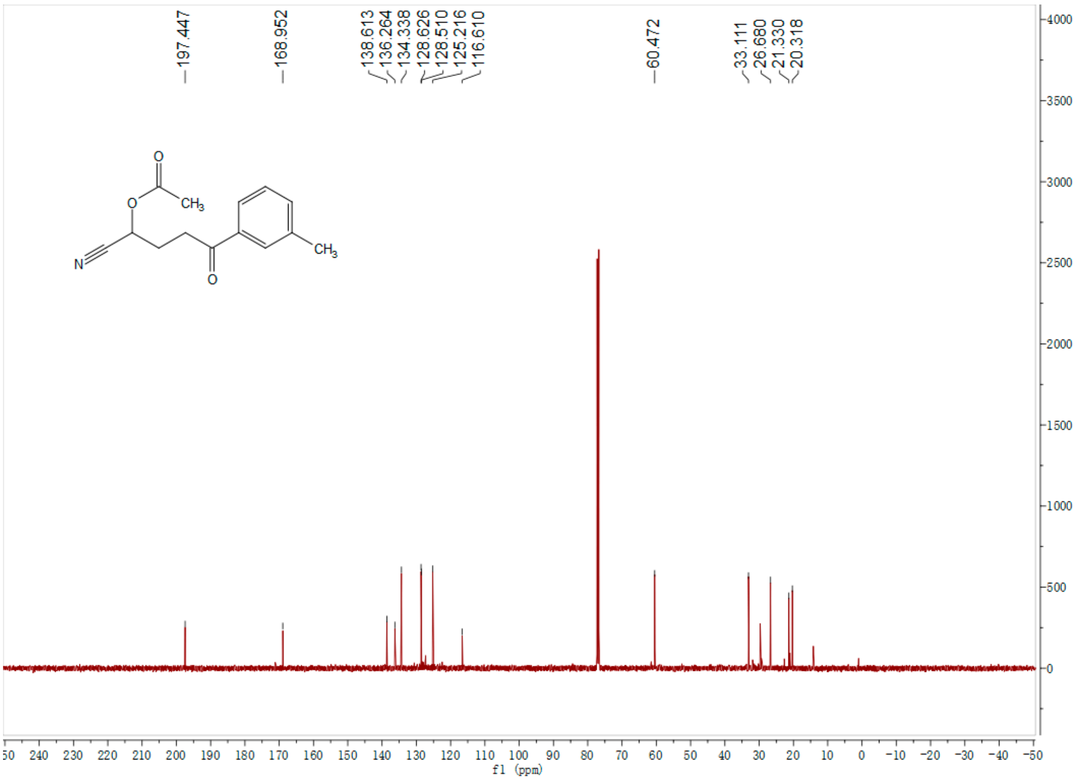

Figure S5 ^{13}C NMR spectra of **3c**

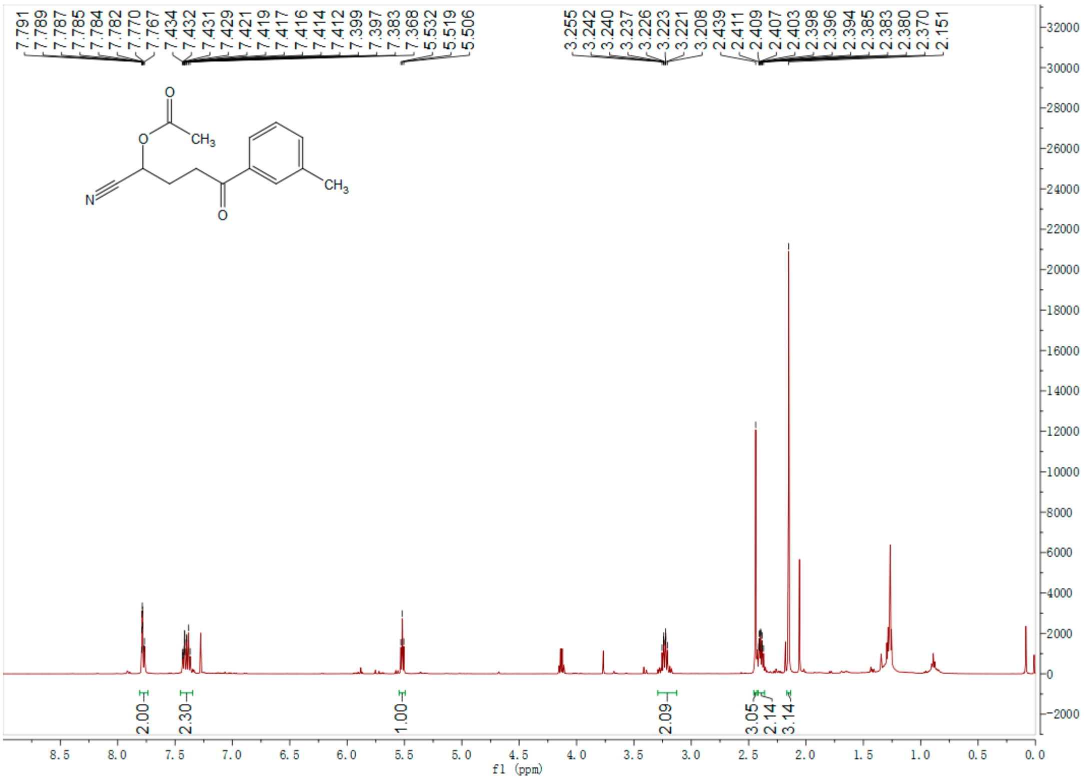

Figure S6 ^{1}H NMR spectra of **3c**

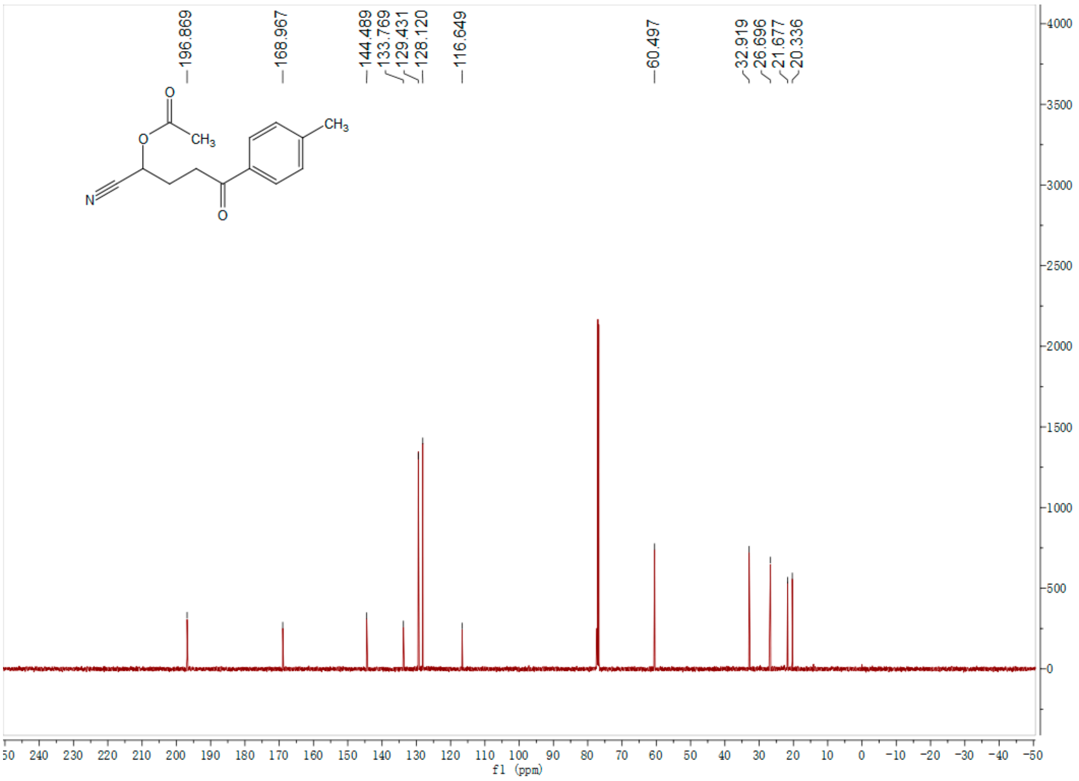

Figure S7 ^{13}C NMR spectra of **3d**

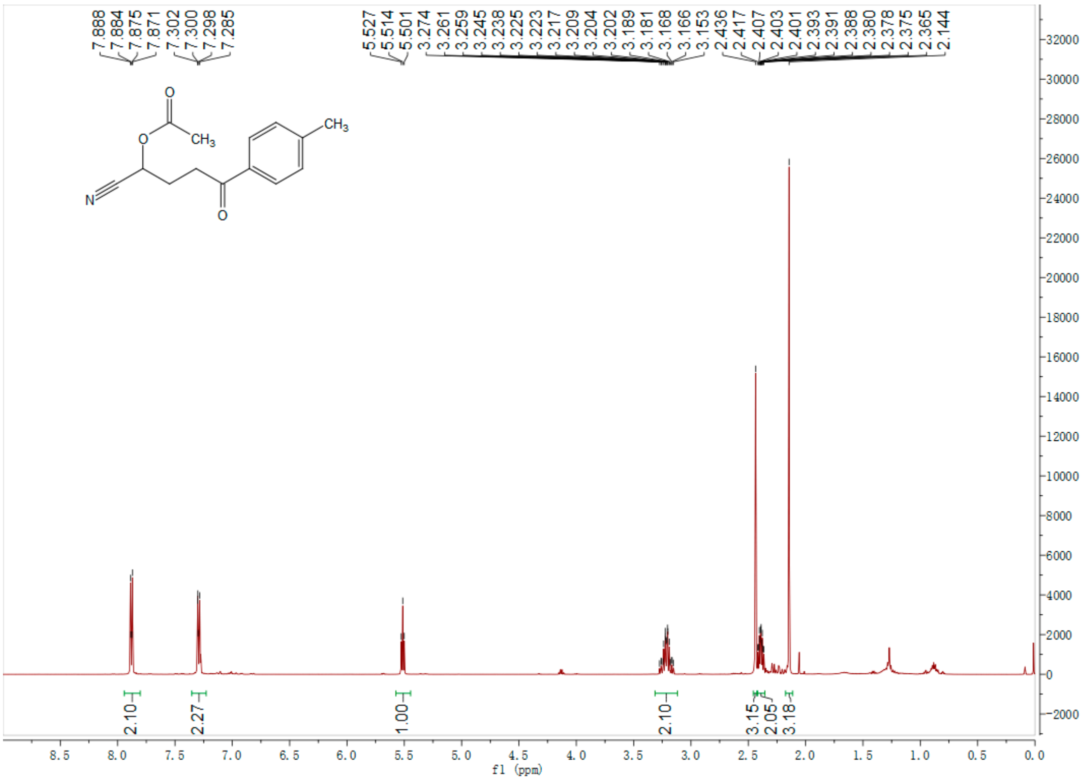

Figure S8 ^{1}H NMR spectra of **3d**

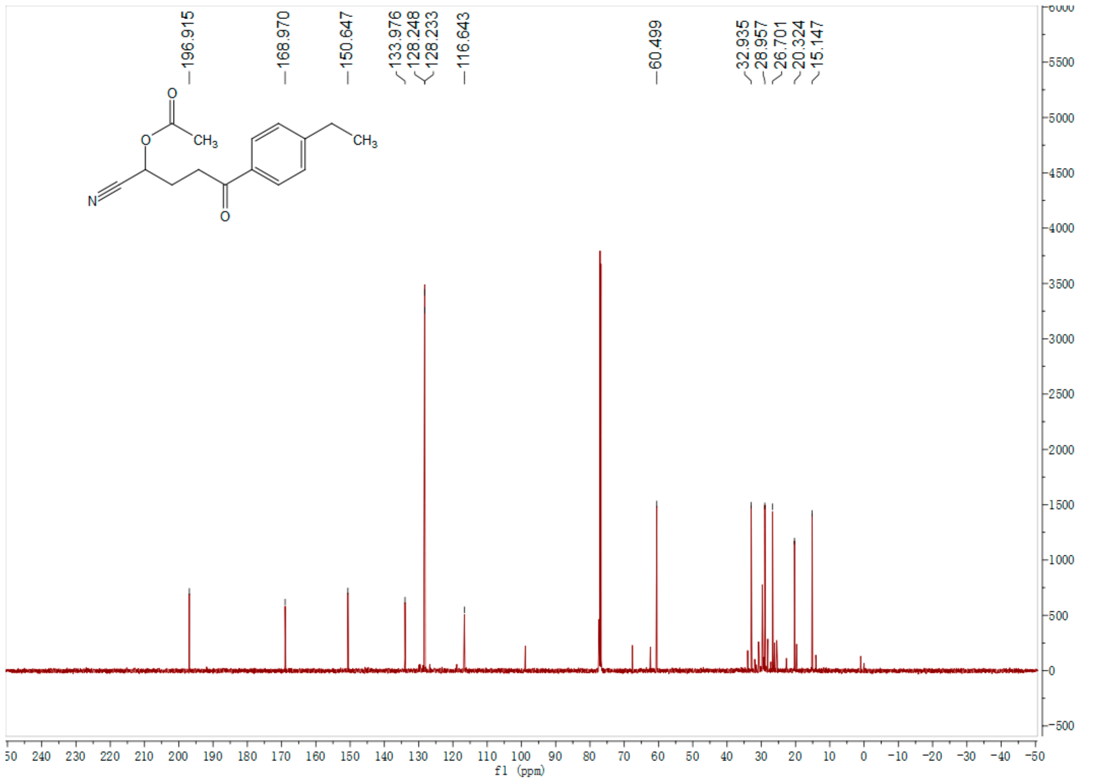

Figure S9 ^{13}C NMR spectra of **3e**

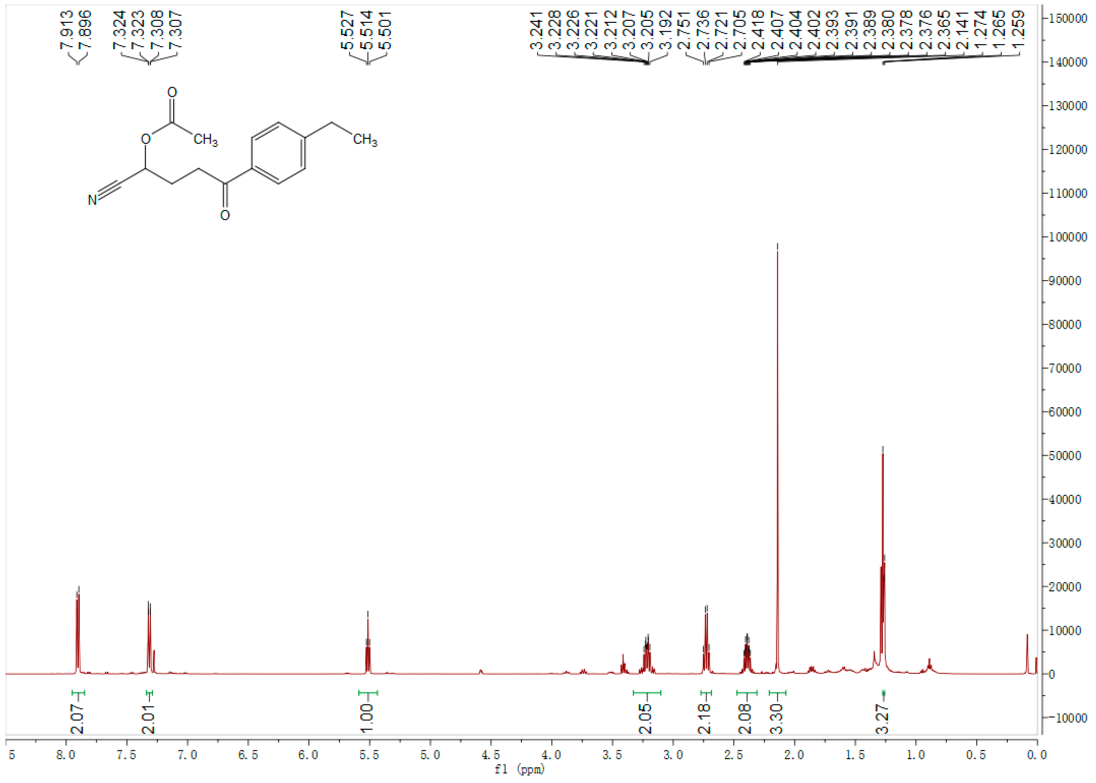

Figure S10 ^{1}H NMR spectra of **3e**

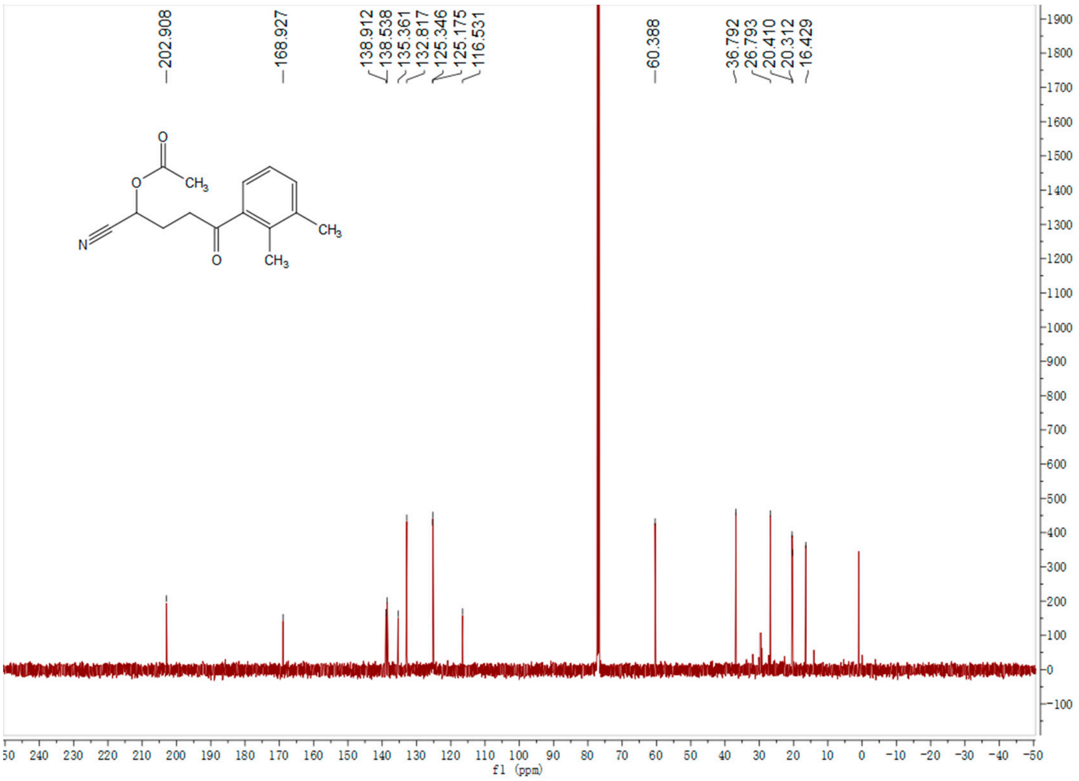

Figure S11 ^{13}C NMR spectra of **3f**

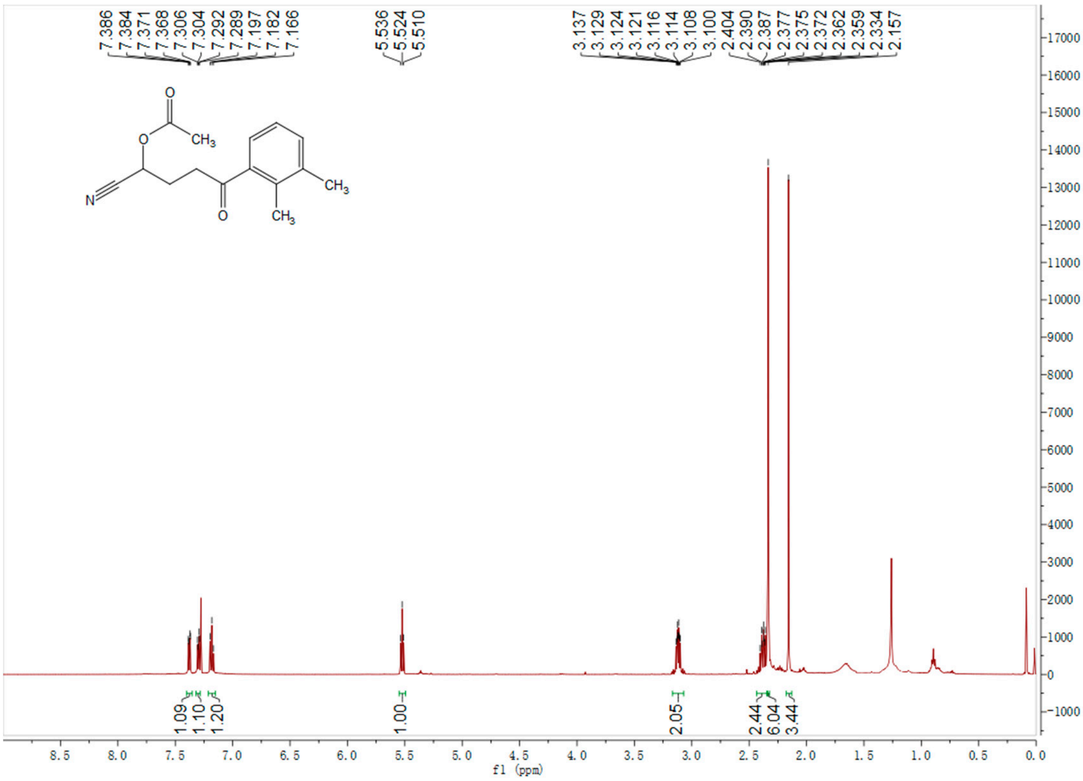

Figure S12 ^{1}H NMR spectra of **3f**

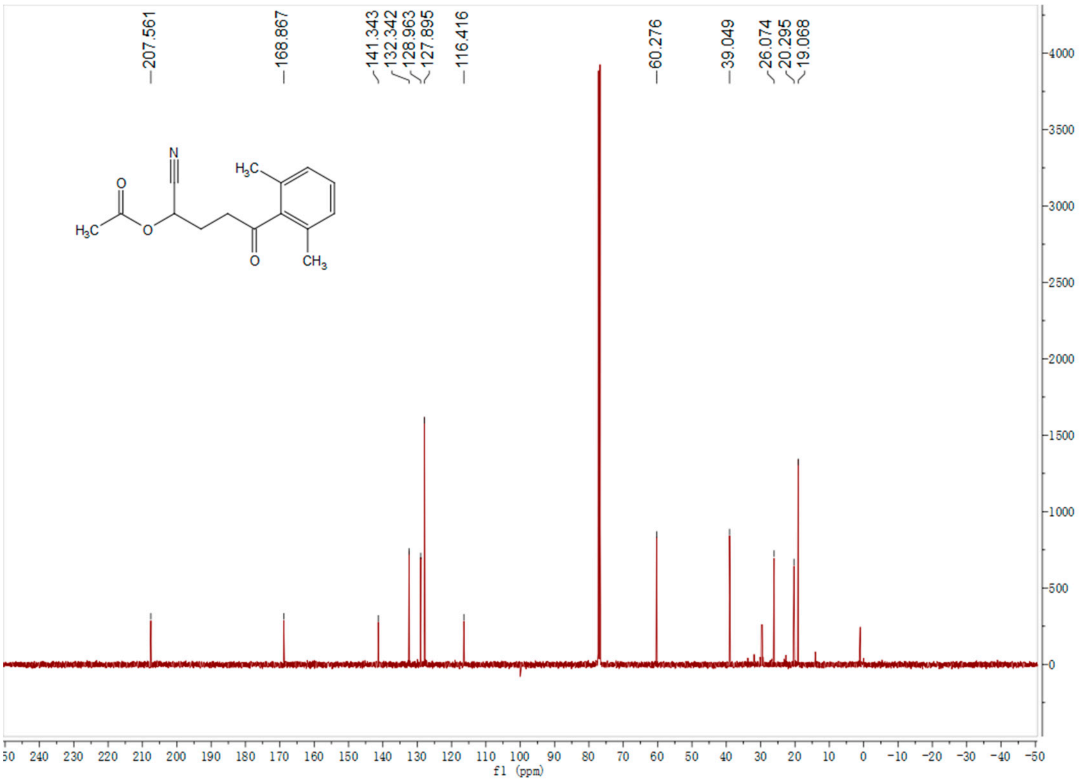

Figure S13 ^{13}C NMR spectra of **3g**

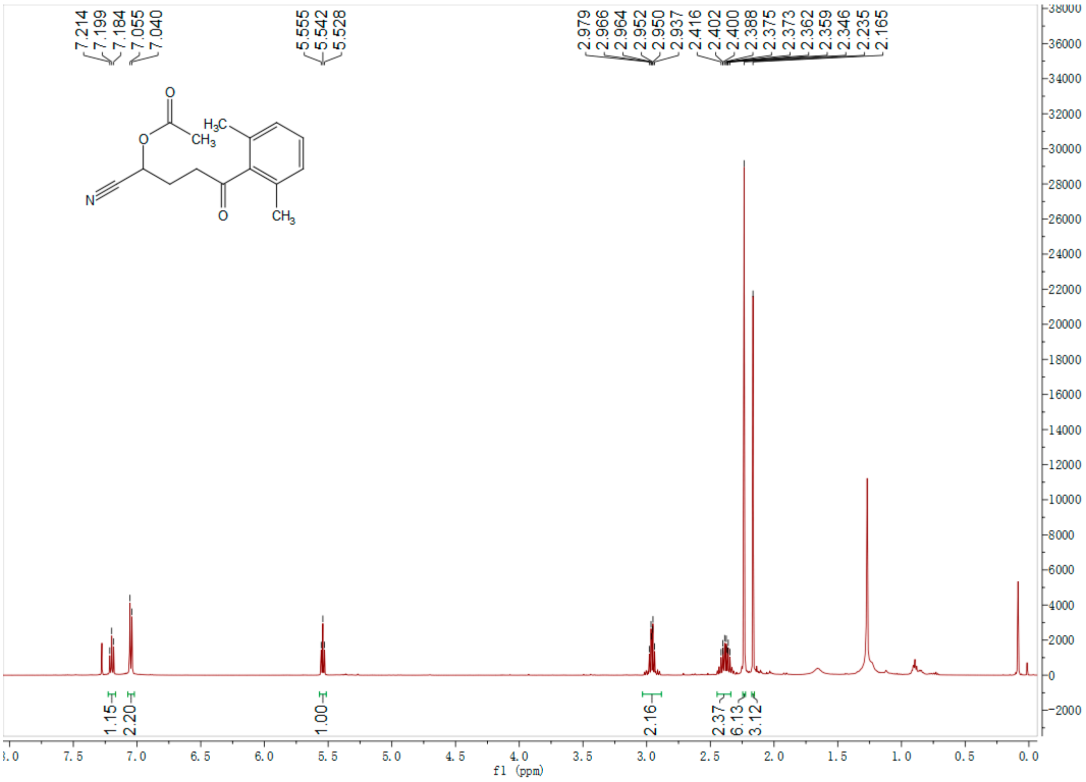

Figure S14 ^{1}H NMR spectra of **3g**

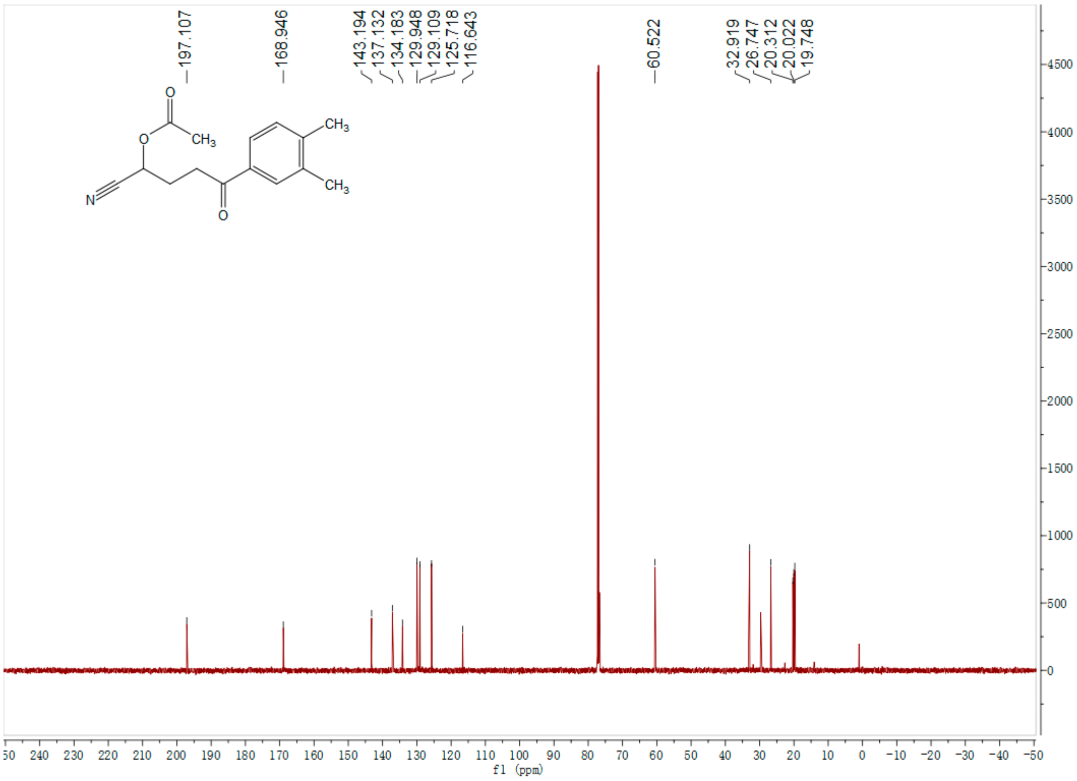

Figure S15 ^{13}C NMR spectra of **3h**

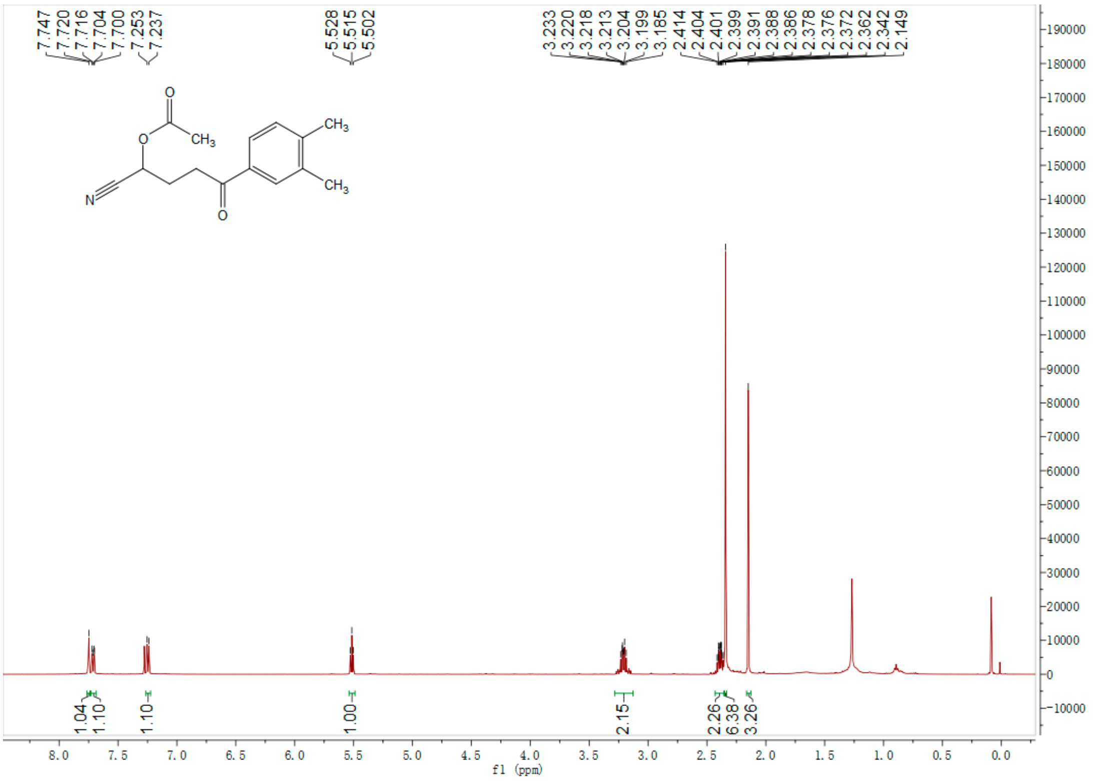

Figure S16 ^{1}H NMR spectra of **3h**

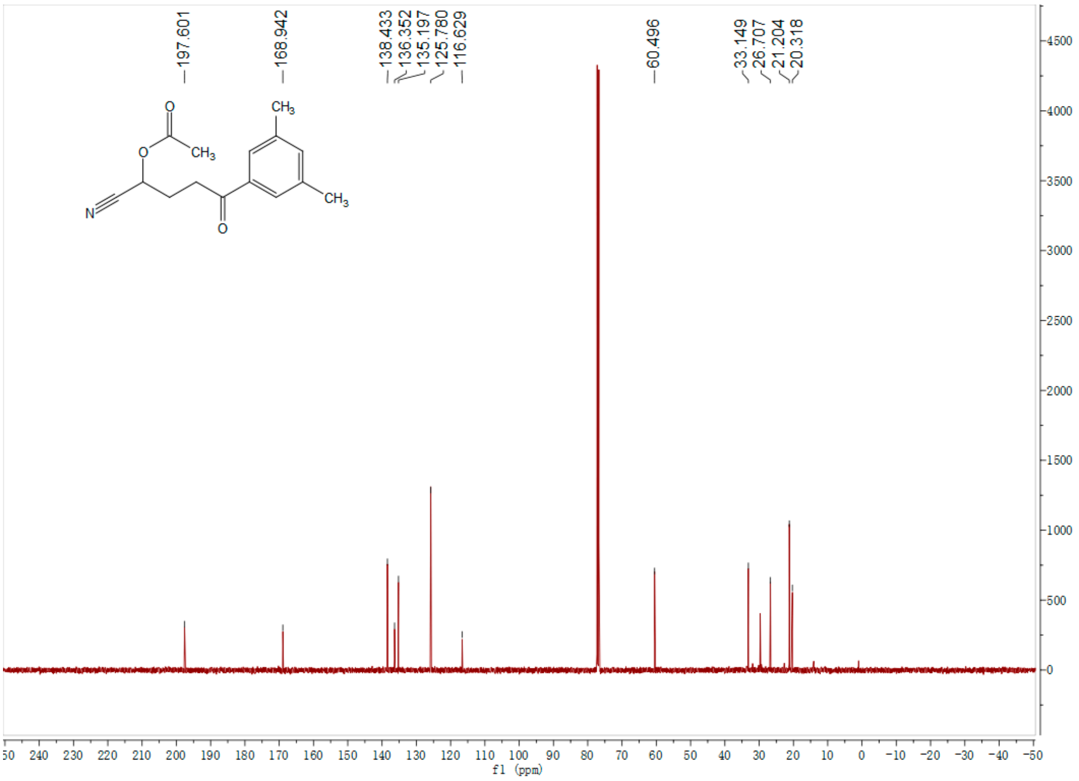

Figure S17 ^{13}C NMR spectra of **3i**

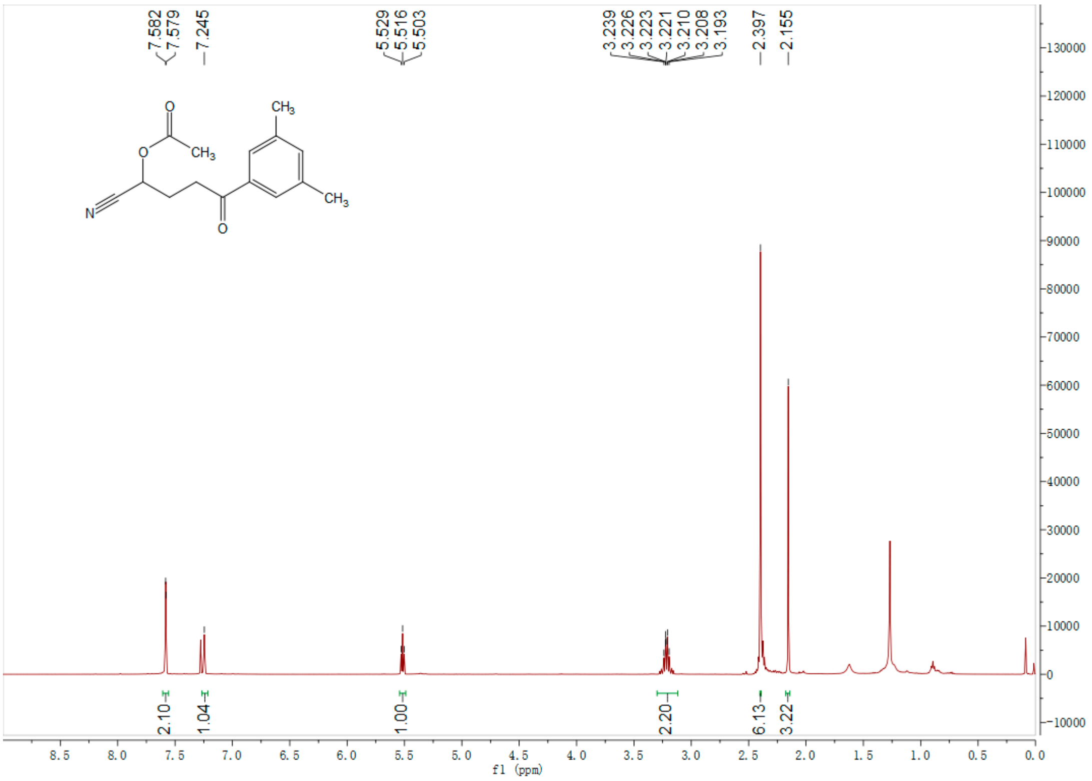

Figure S18 ^{1}H NMR spectra of **3i**

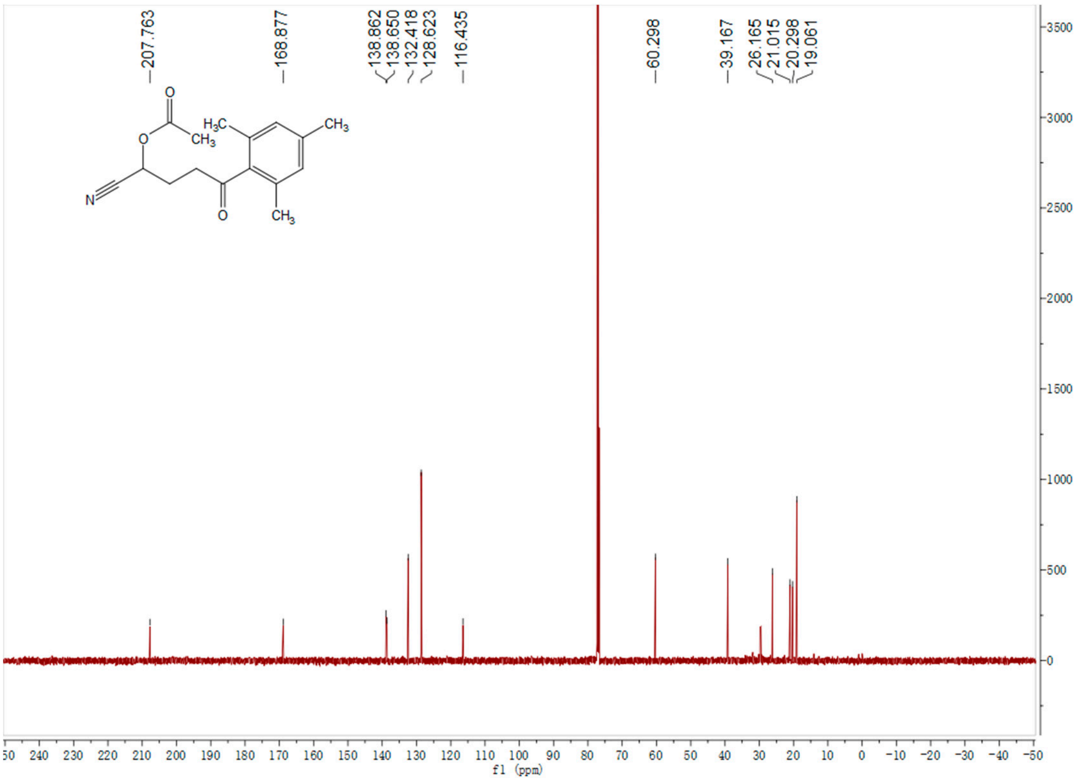

Figure S19 ^{13}C NMR spectra of **3j**

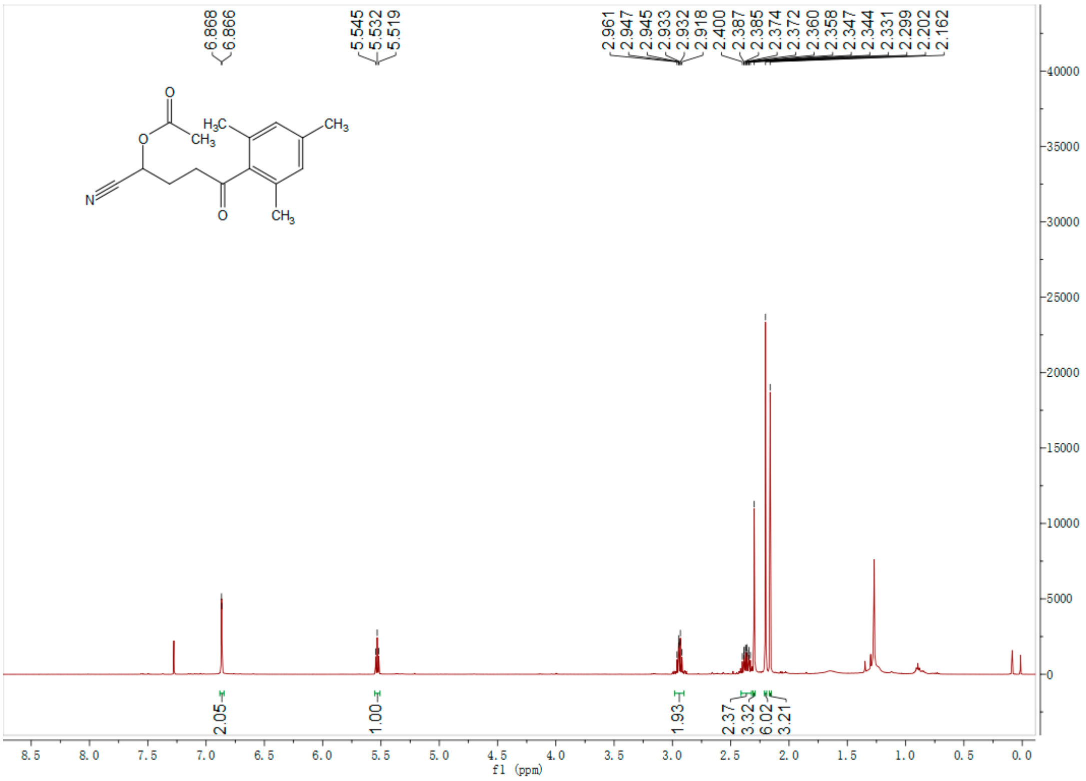

Figure S20 ^{1}H NMR spectra of **3j**

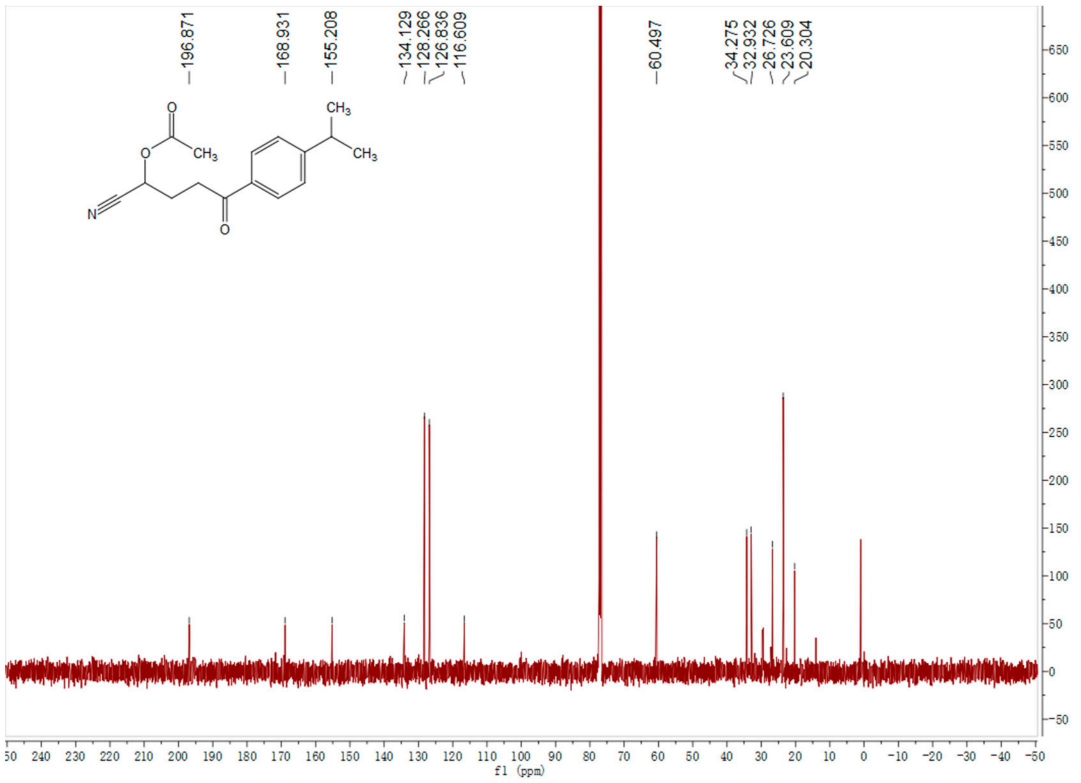

Figure S21 ^{13}C NMR spectra of **3k**

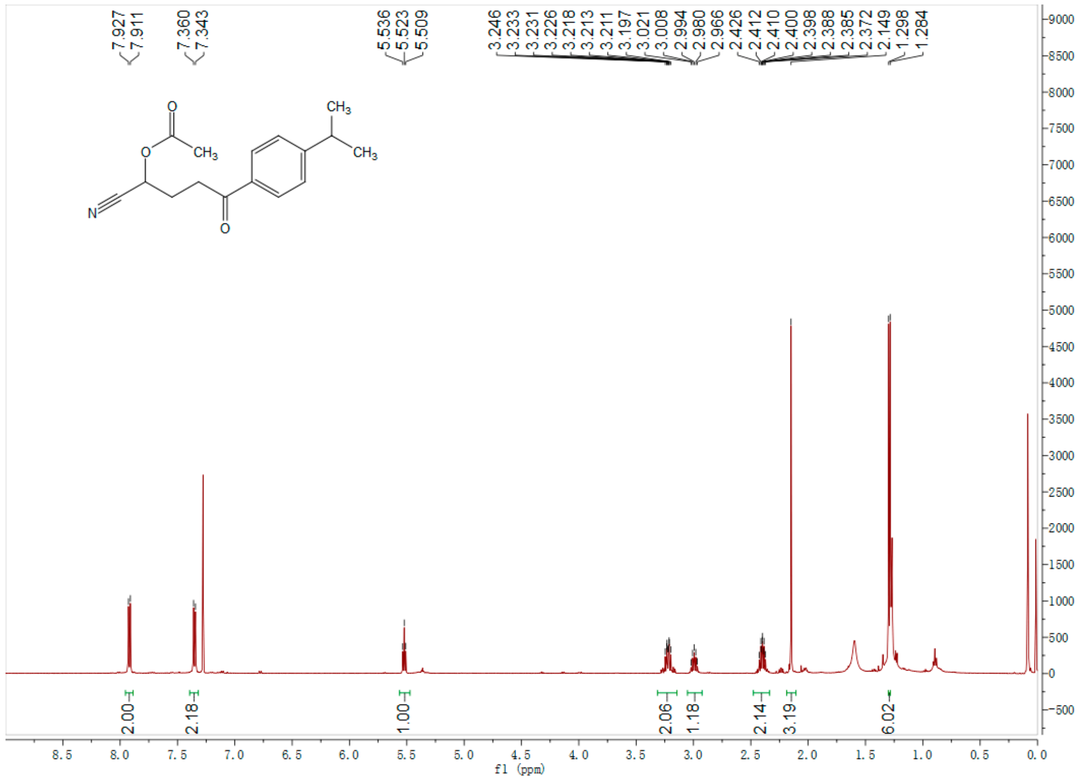

Figure S22 ^{1}H NMR spectra of **3k**

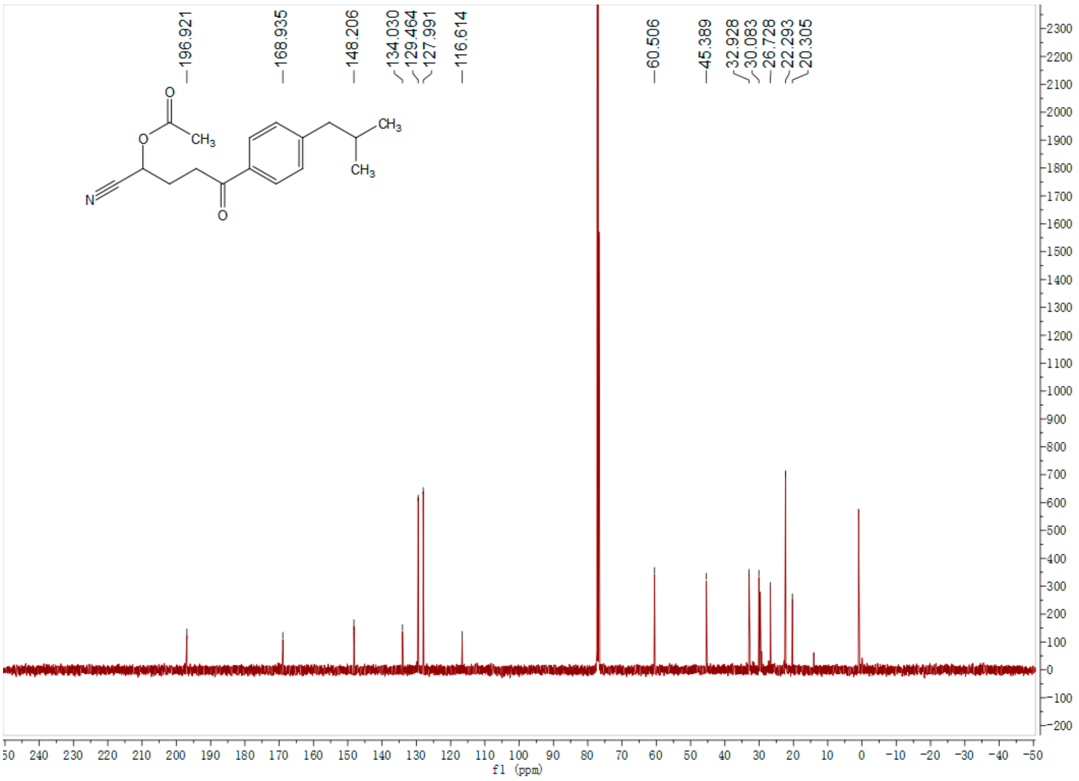

Figure S23 ^{1}H NMR spectra of **3l**

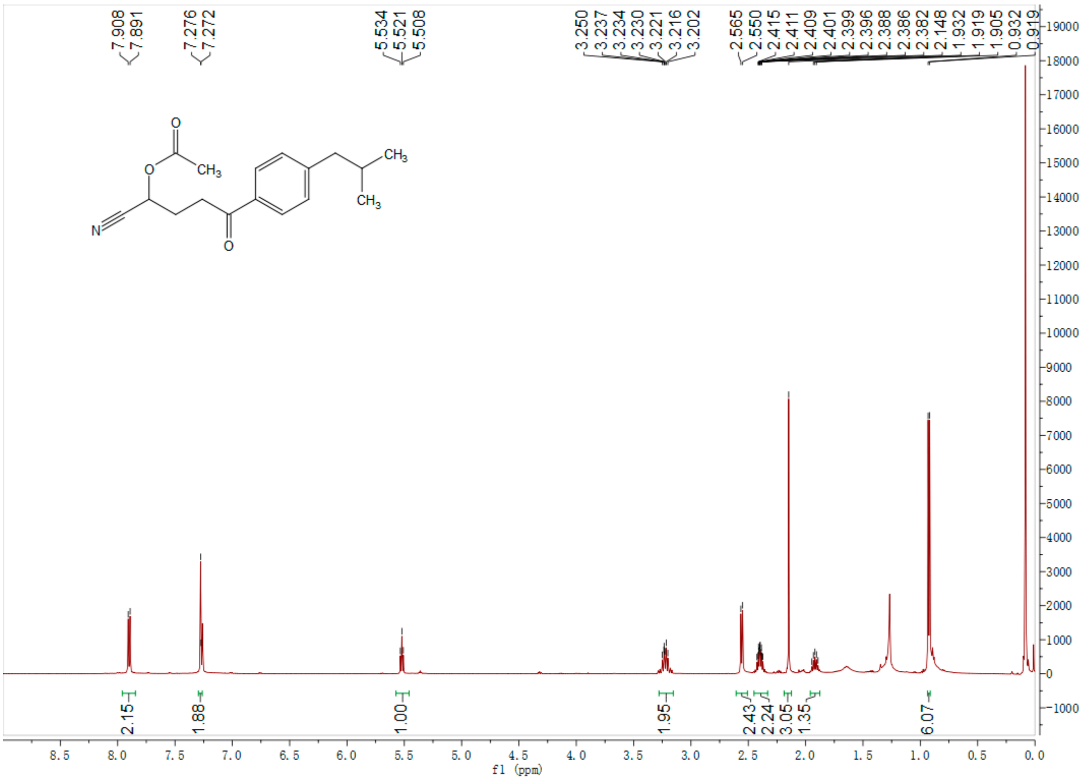

Figure S24 ^{13}C NMR spectra of **3l**

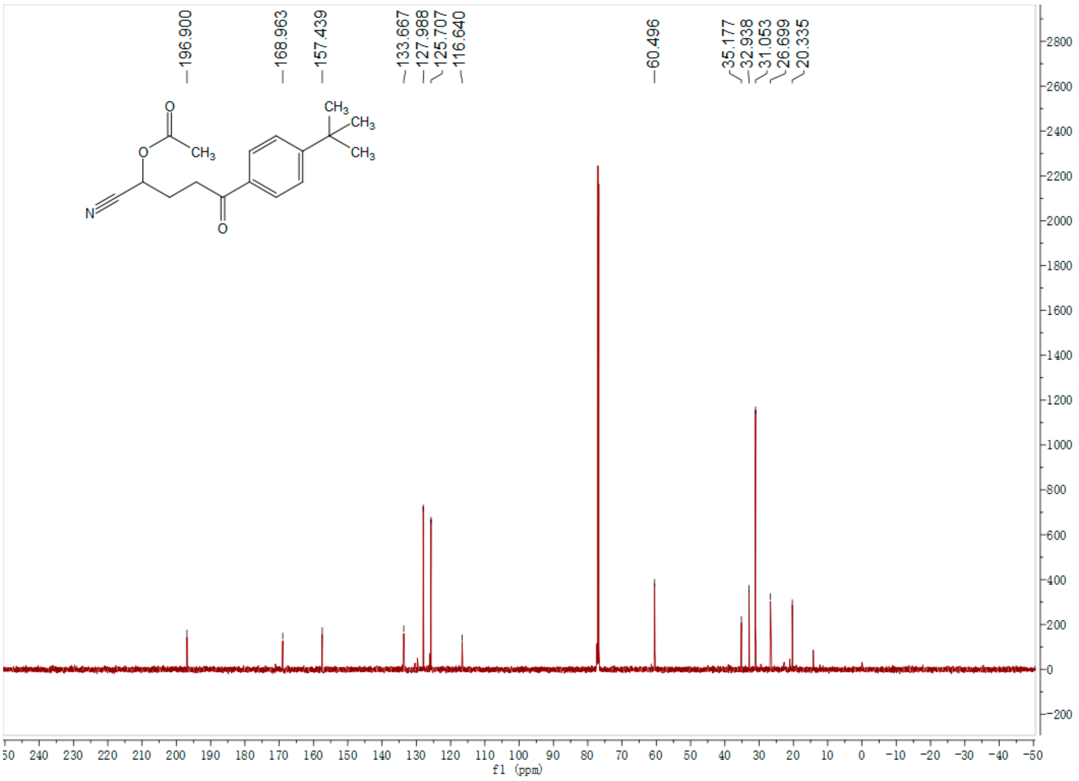

Figure S25 ^{13}C NMR spectra of **3m**

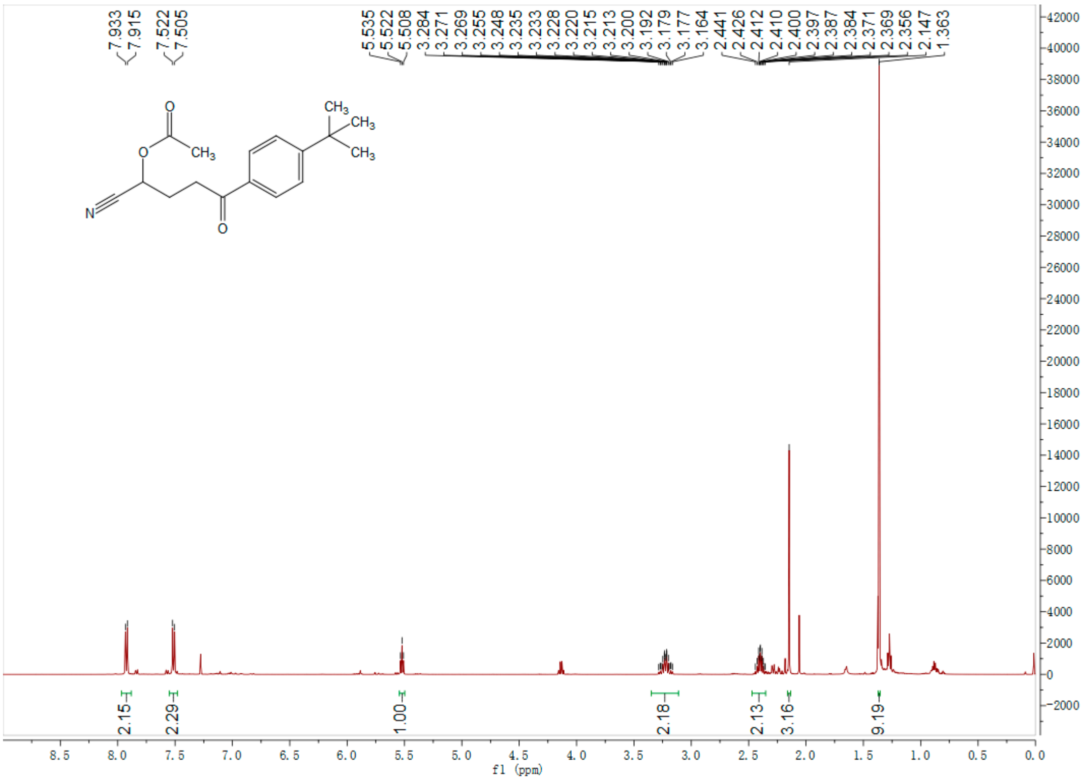

Figure S26 ^{1}H NMR spectra of **3m**

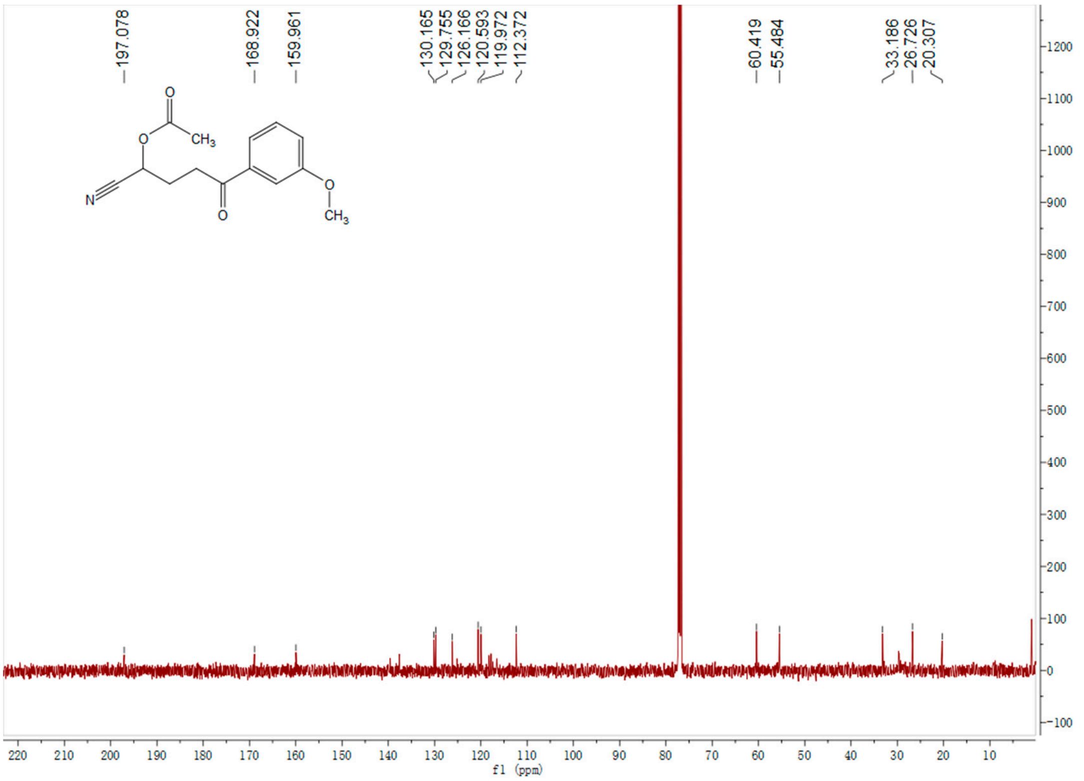

Figure S27 ^{13}C NMR spectra of **3n**

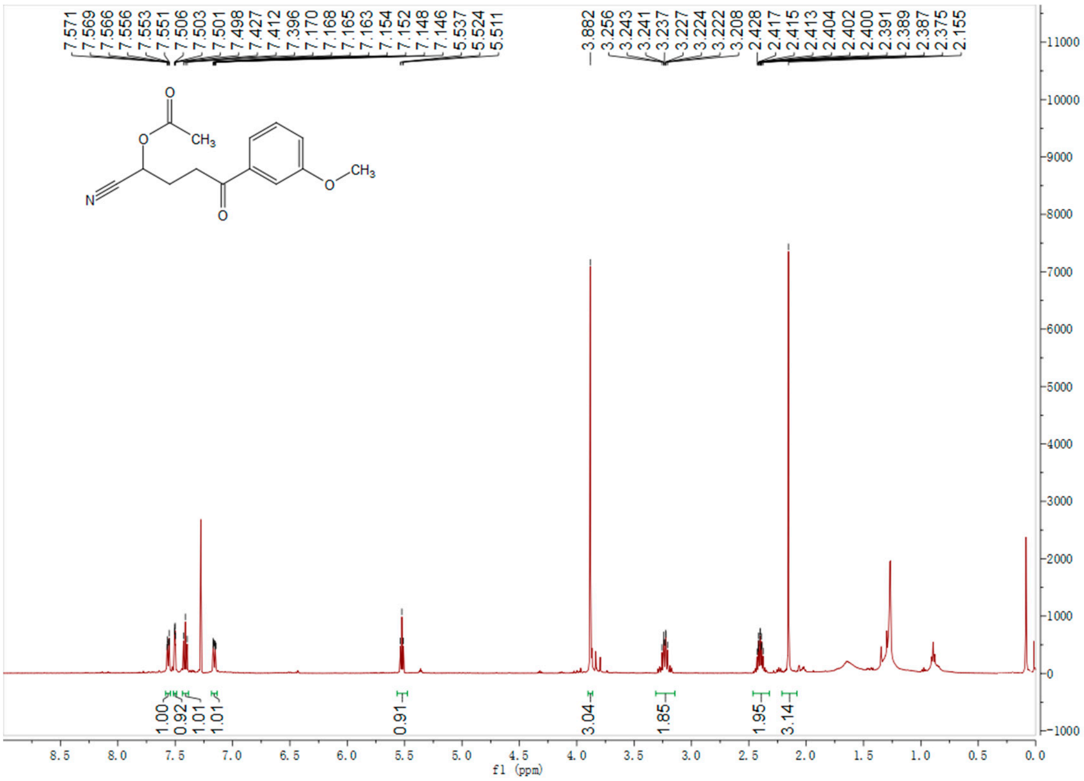

Figure S28 ^{1}H NMR spectra of **3n**

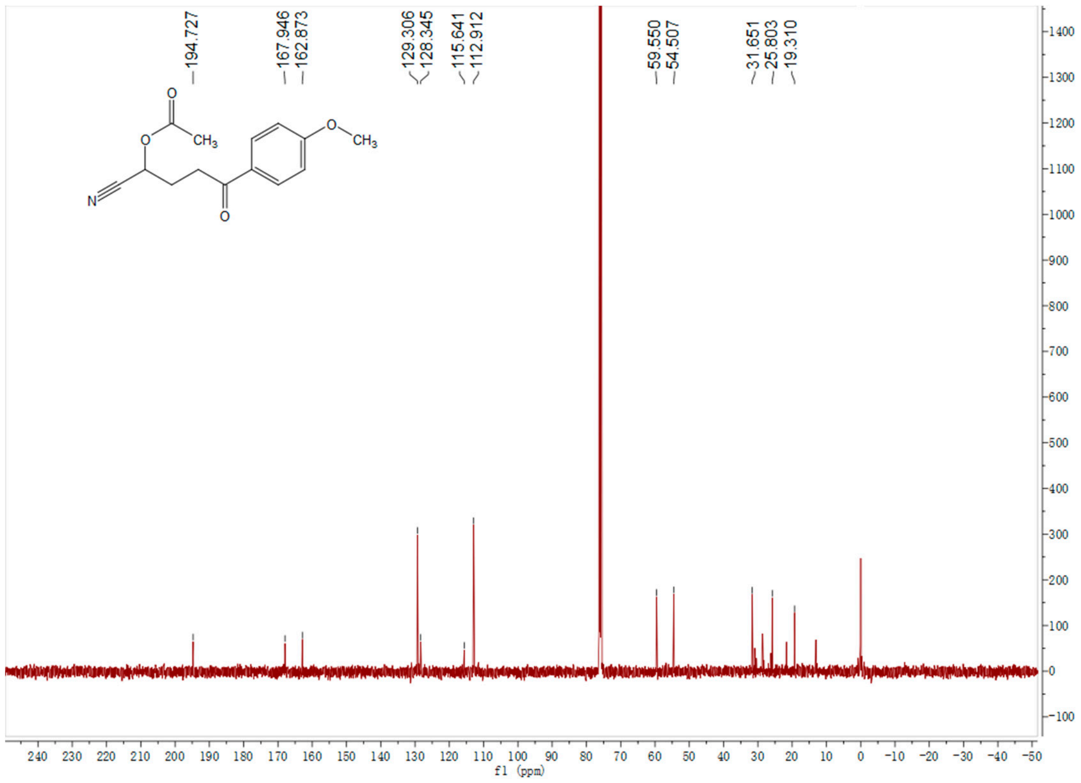

Figure S29 ^{13}C NMR spectra of **3o**

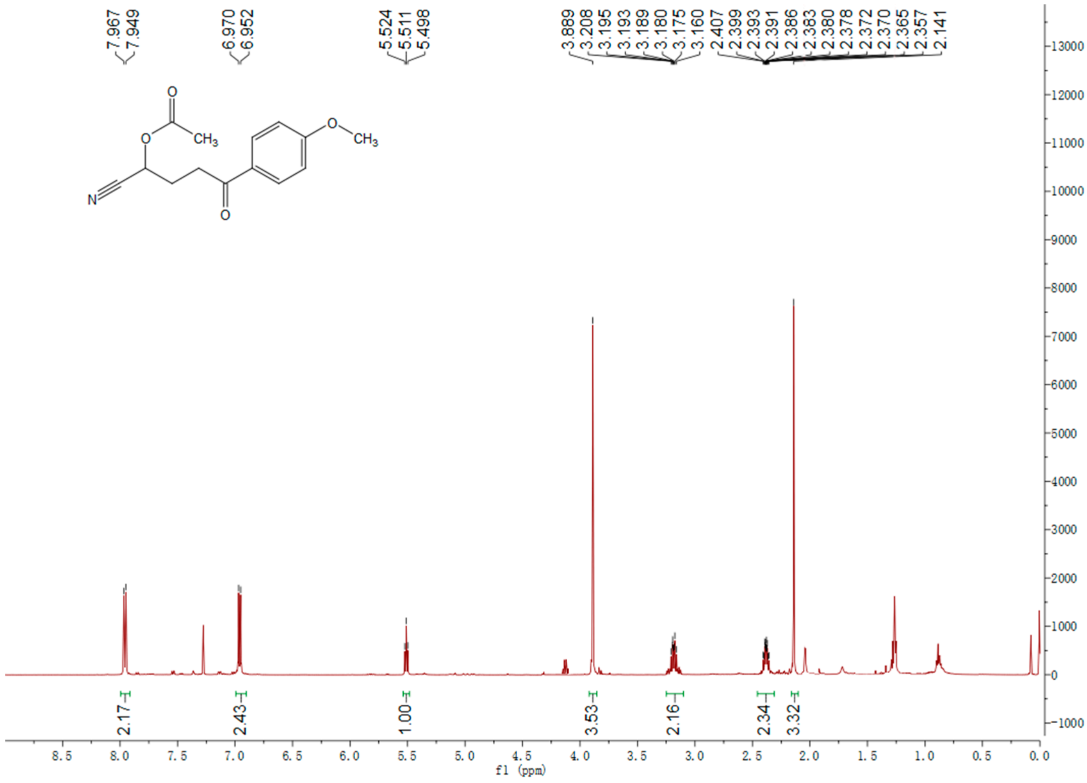

Figure S30 ^{1}H NMR spectra of **3o**

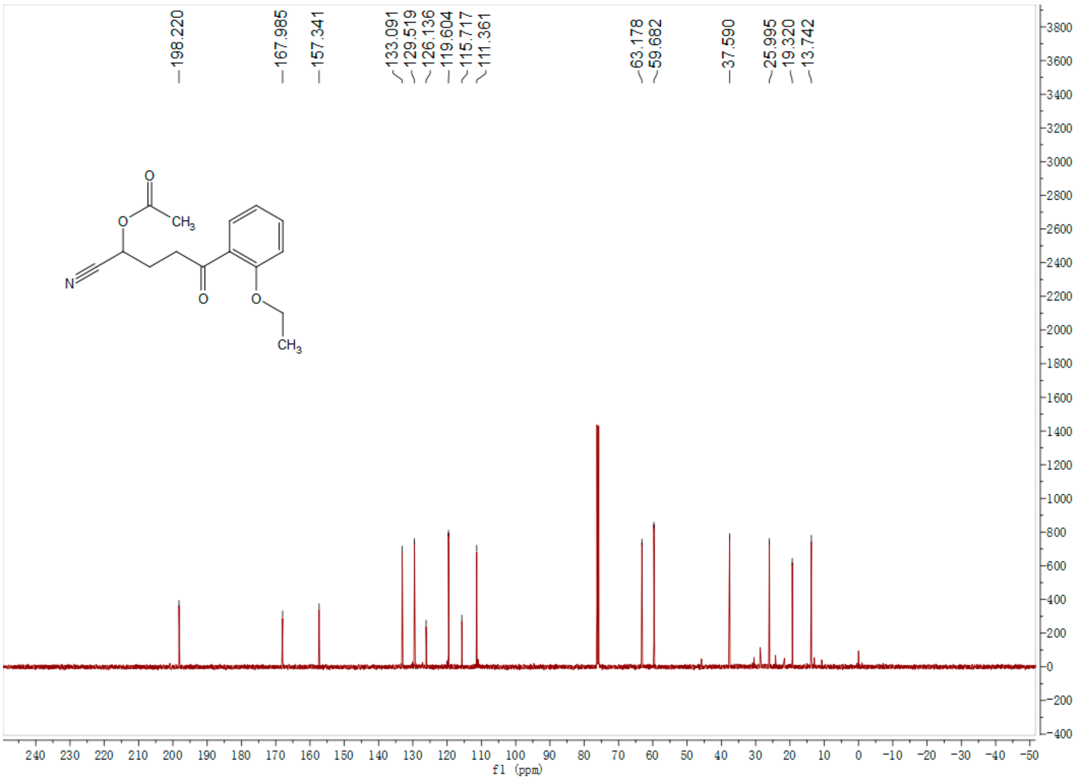

Figure S31 ^{13}C NMR spectra of **3p**

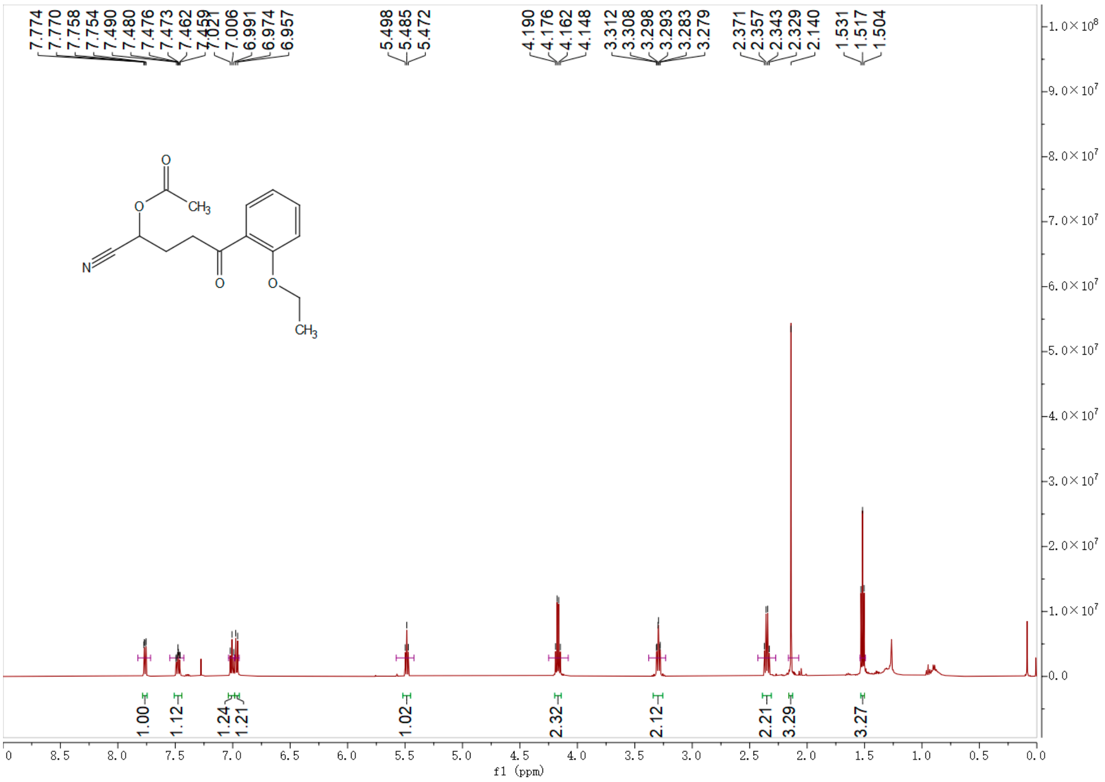

Figure S32 ^{1}H NMR spectra of **3p**

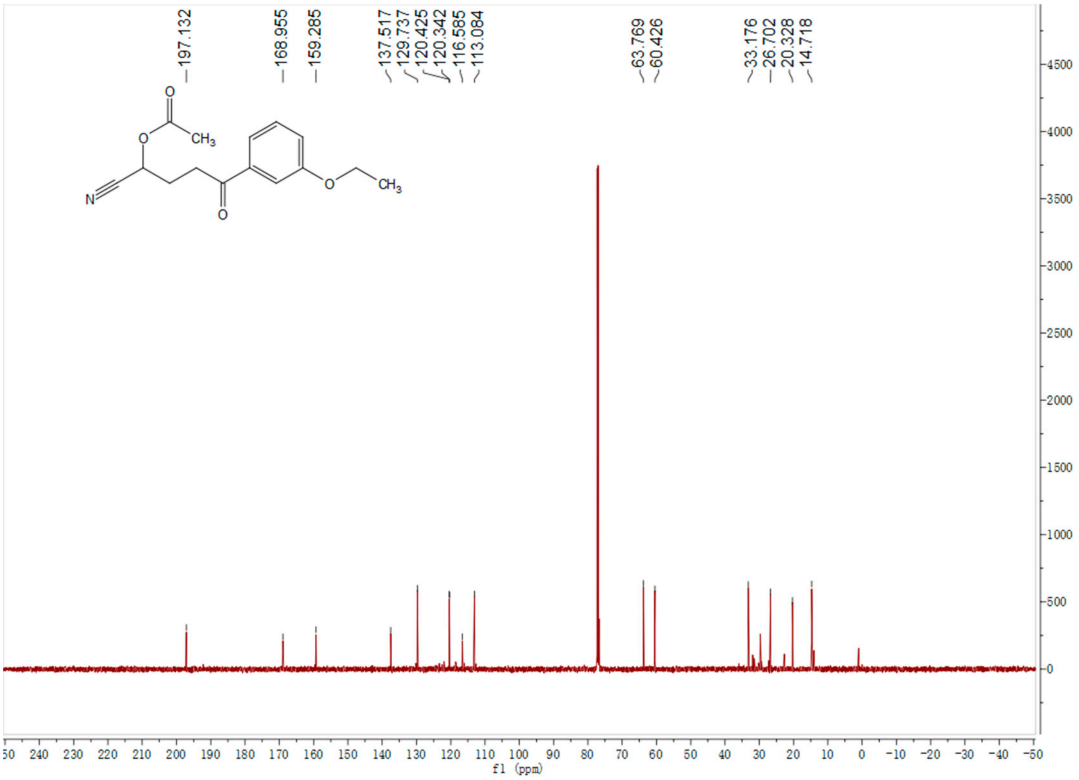

Figure S33 ^{13}C NMR spectra of **3q**

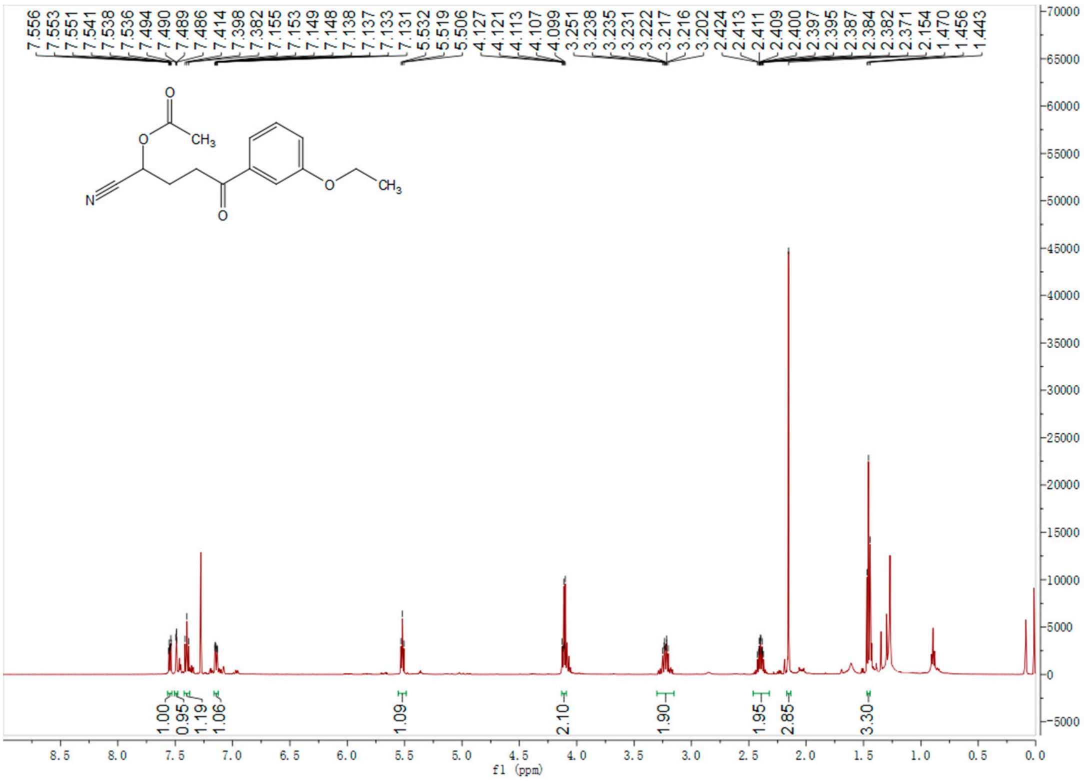

Figure S34 ^{1}H NMR spectra of **3q**

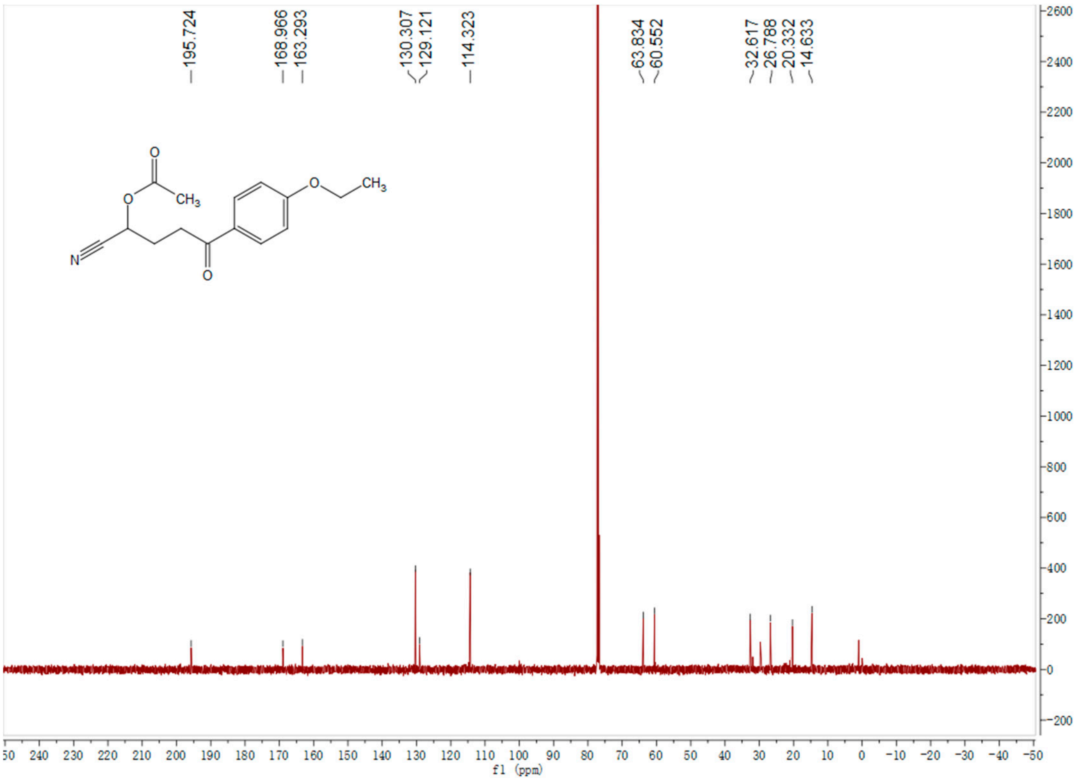

Figure S35 ^{13}C NMR spectra of **3r**

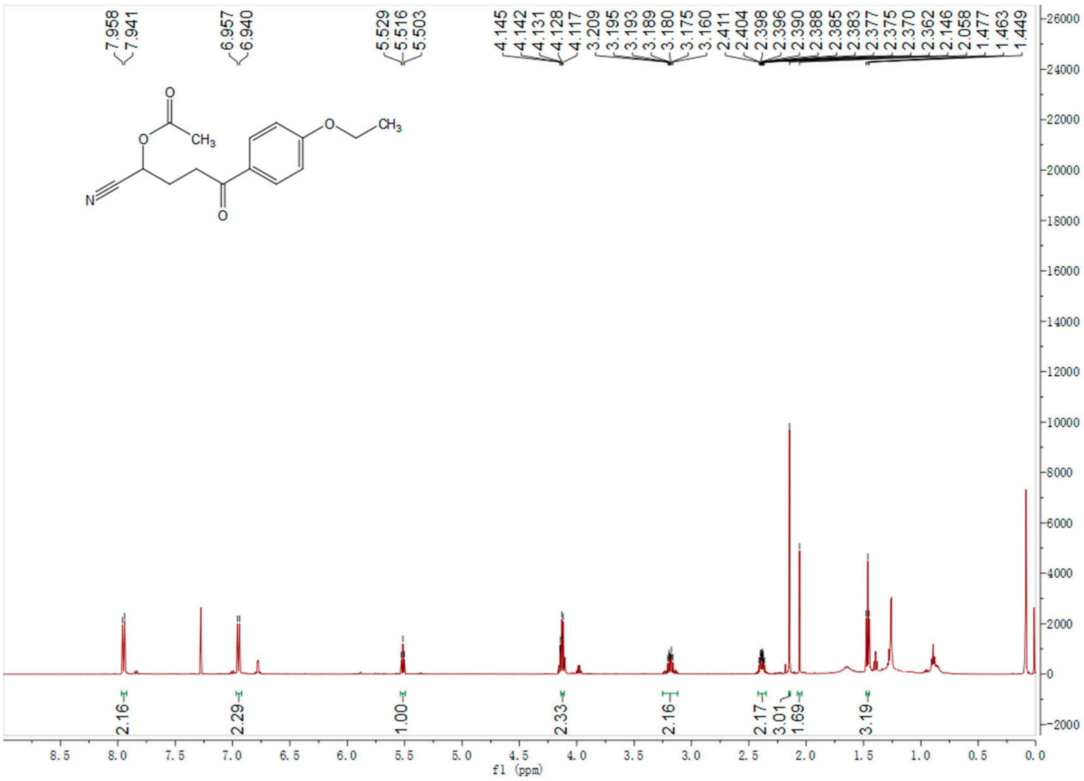

Figure S36 ^{1}H NMR spectra of **3r**

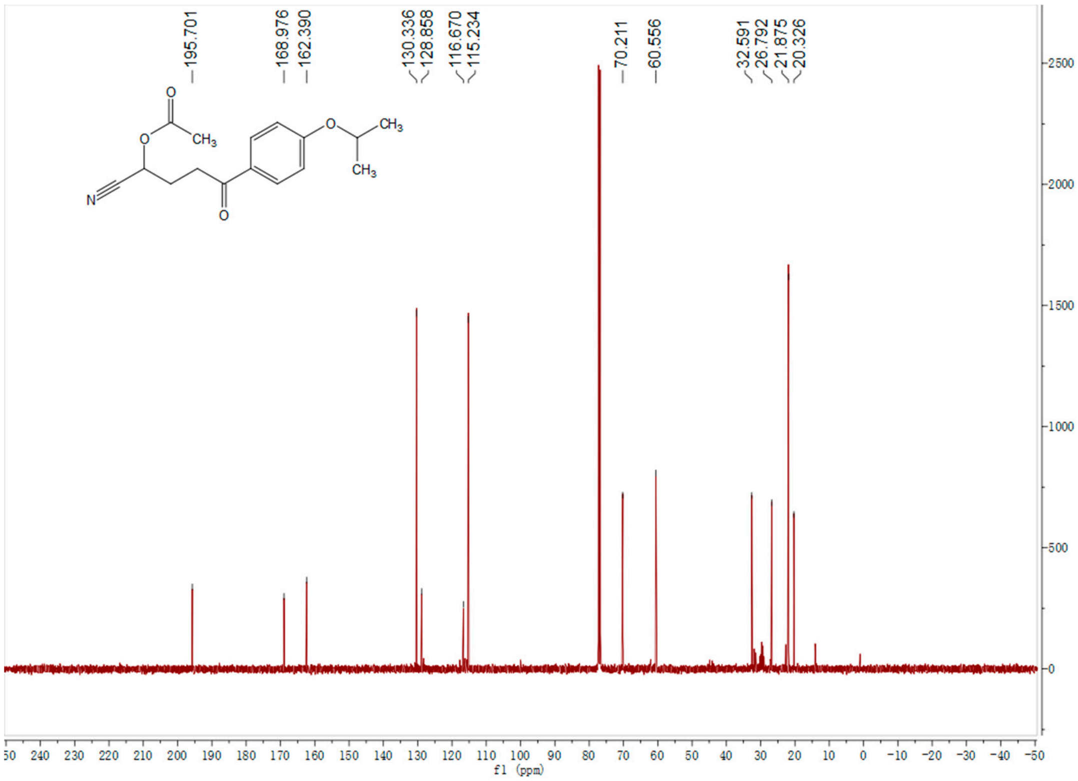

Figure S37 ^{13}C NMR spectra of **3s**

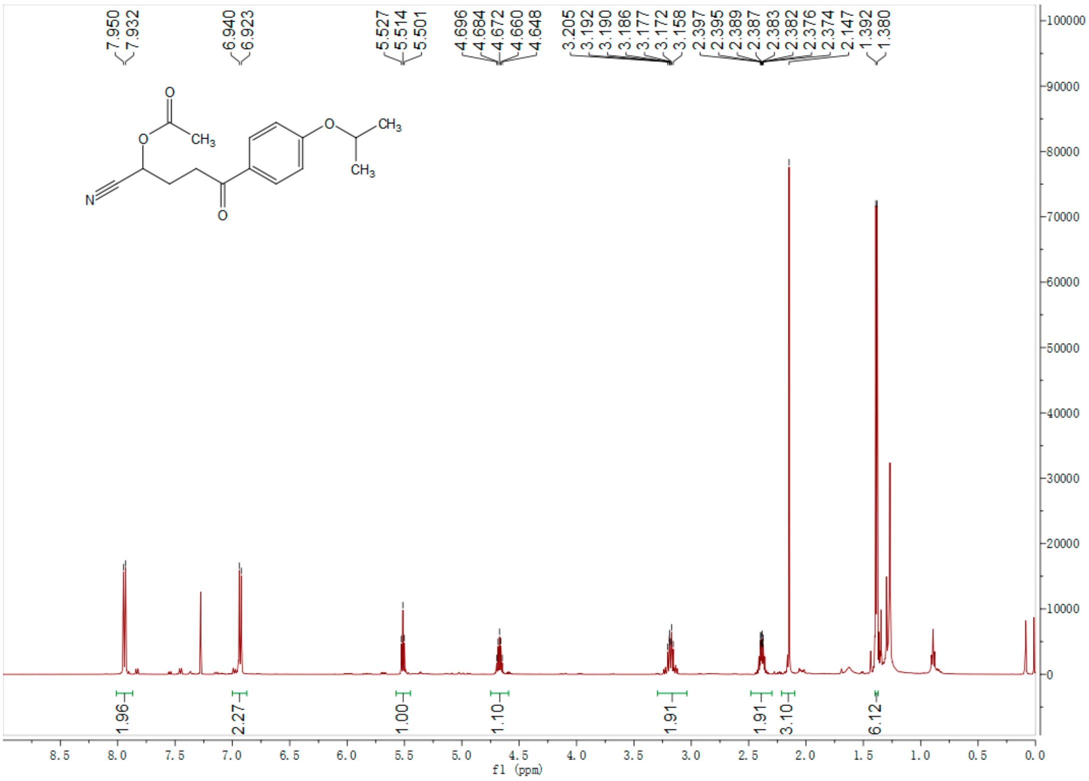

Figure S38 ^{1}H NMR spectra of **3s**

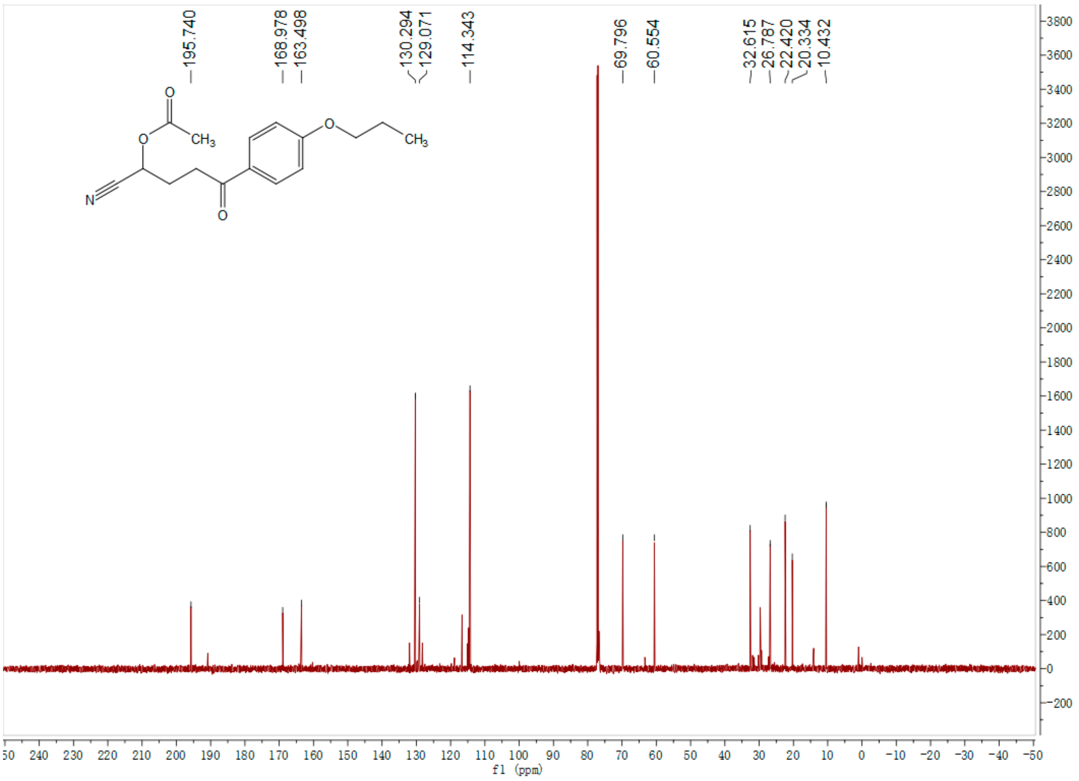

Figure S39 ^{13}C NMR spectra of **3t**

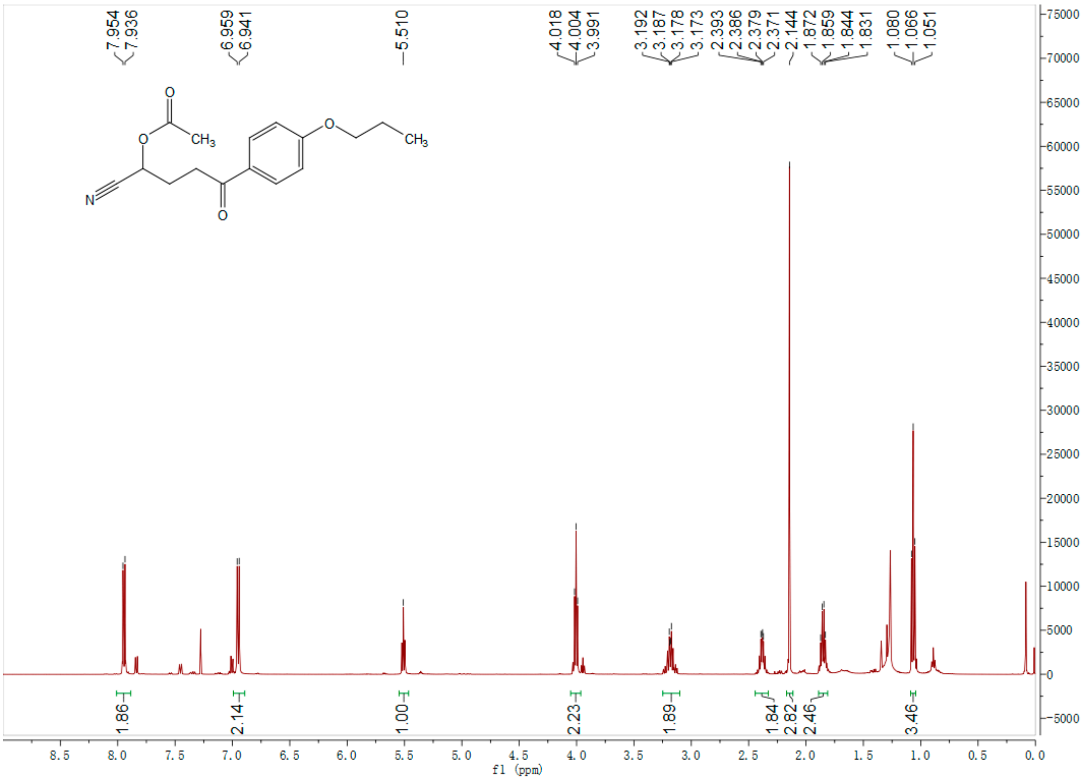

Figure S40 ^{1}H NMR spectra of **3t**

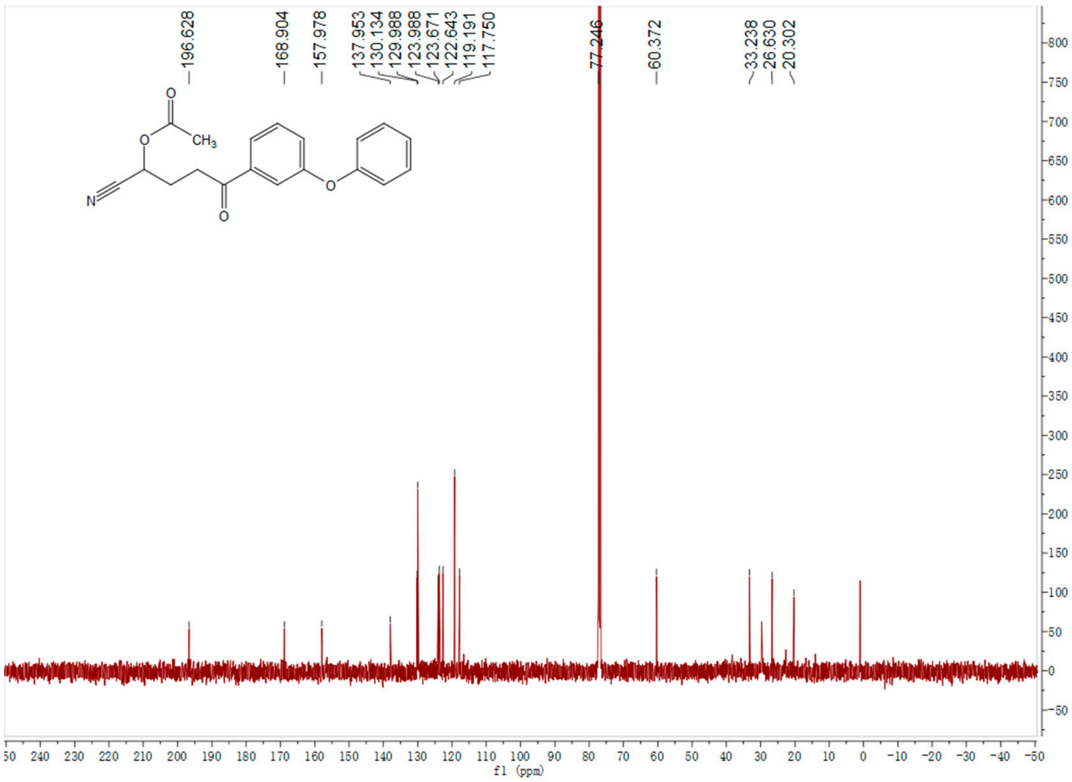

Figure S41 ^{13}C NMR spectra of **3u**

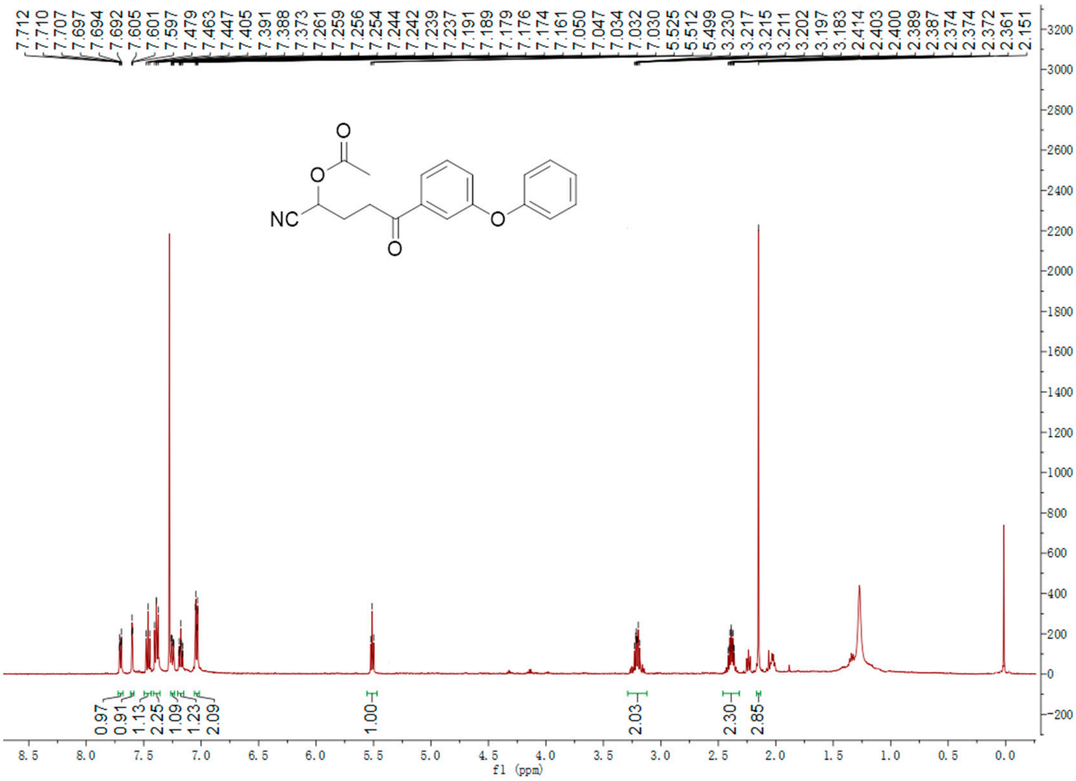

Figure S42 ^{1}H NMR spectra of **3u**

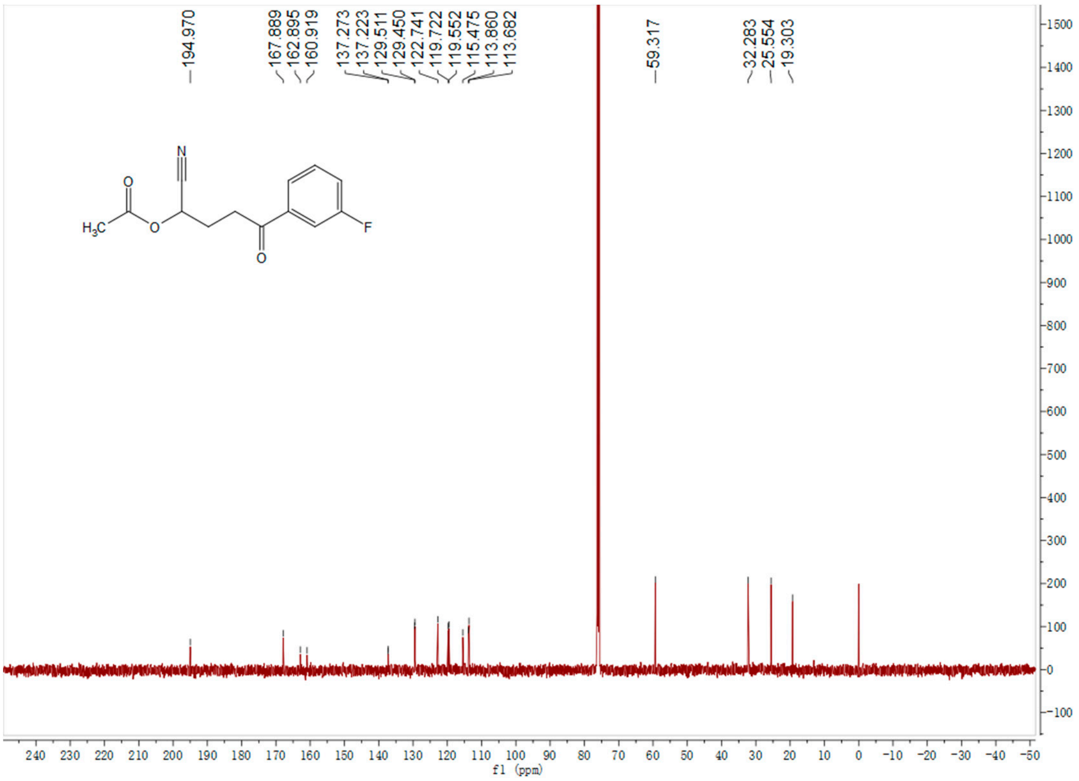

Figure S43 ^{13}C NMR spectra of **3v**

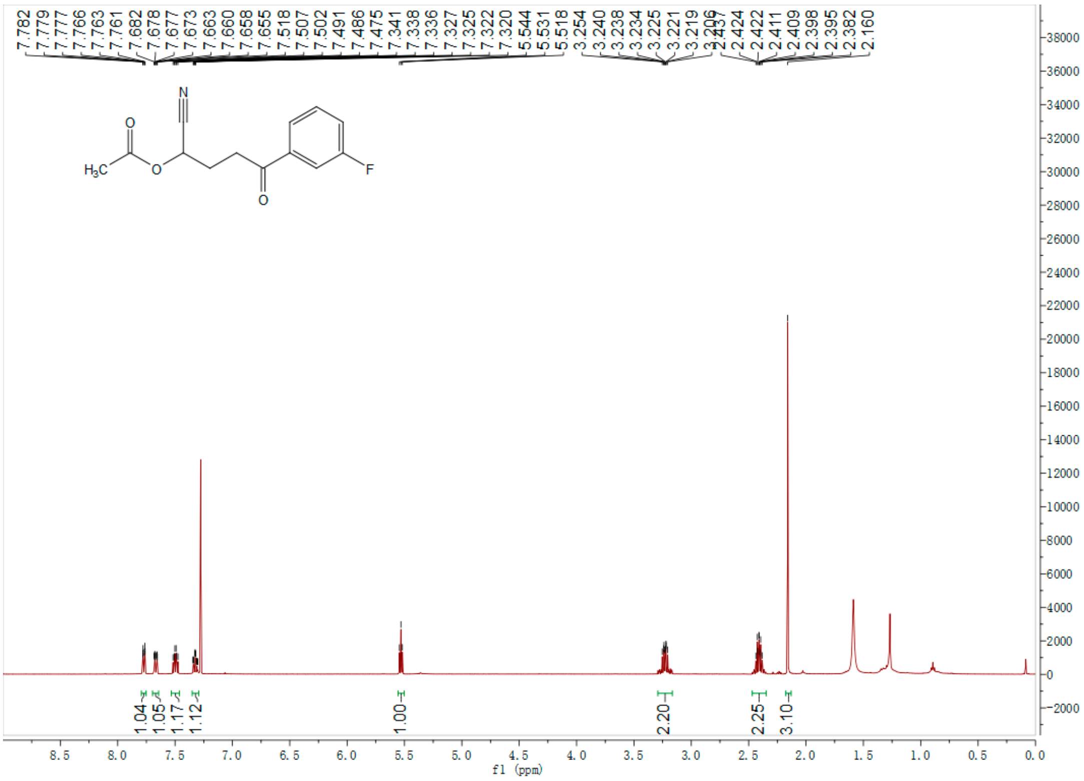

Figure S44 ^{1}H NMR spectra of **3v**

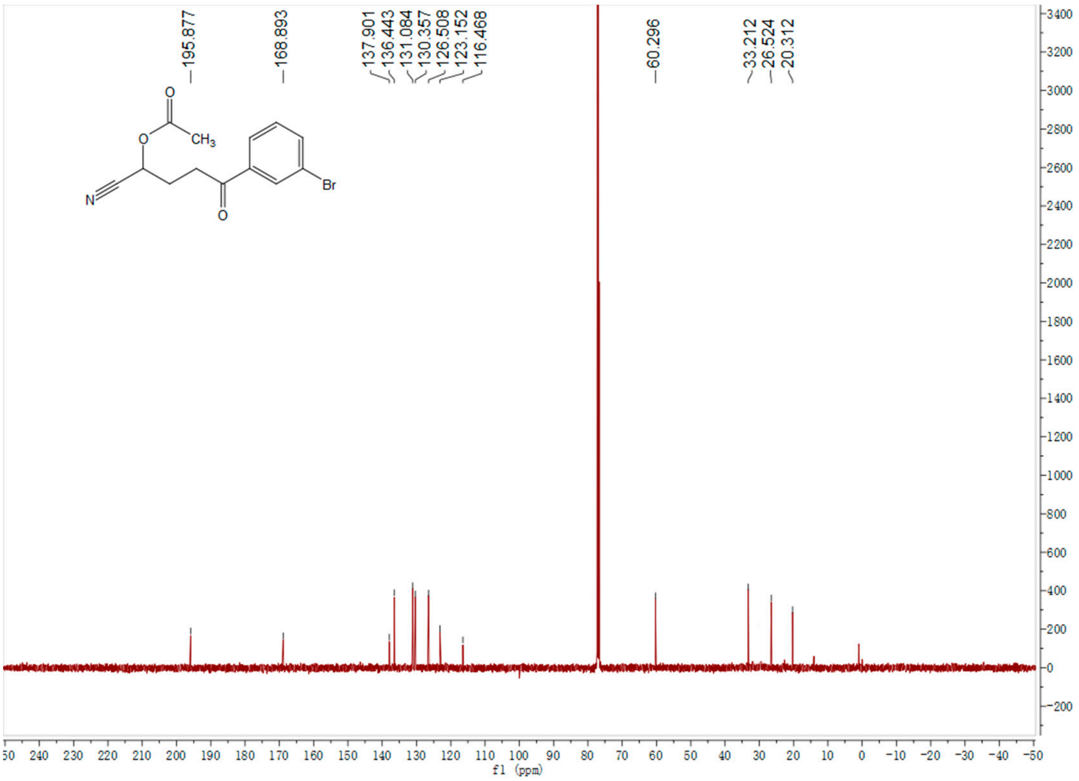

Figure S45 ^{13}C NMR spectra of **3w**

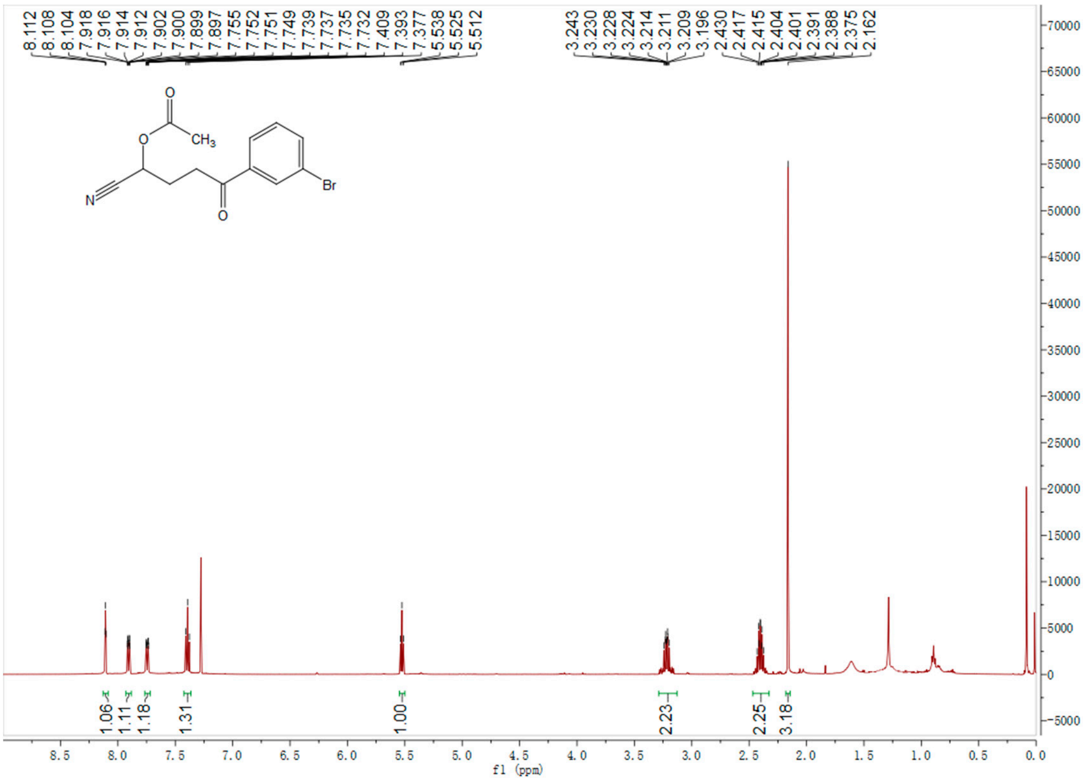

Figure S46 ^{1}H NMR spectra of **3w**

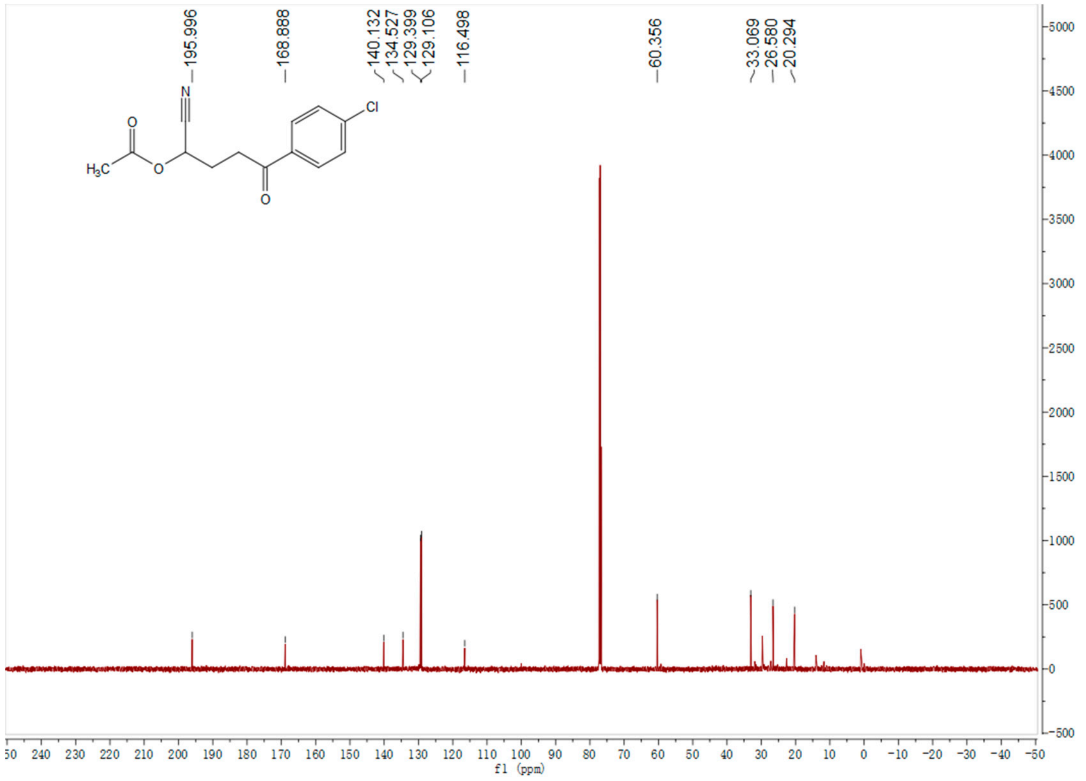

Figure S47 ^{13}C NMR spectra of **3x**

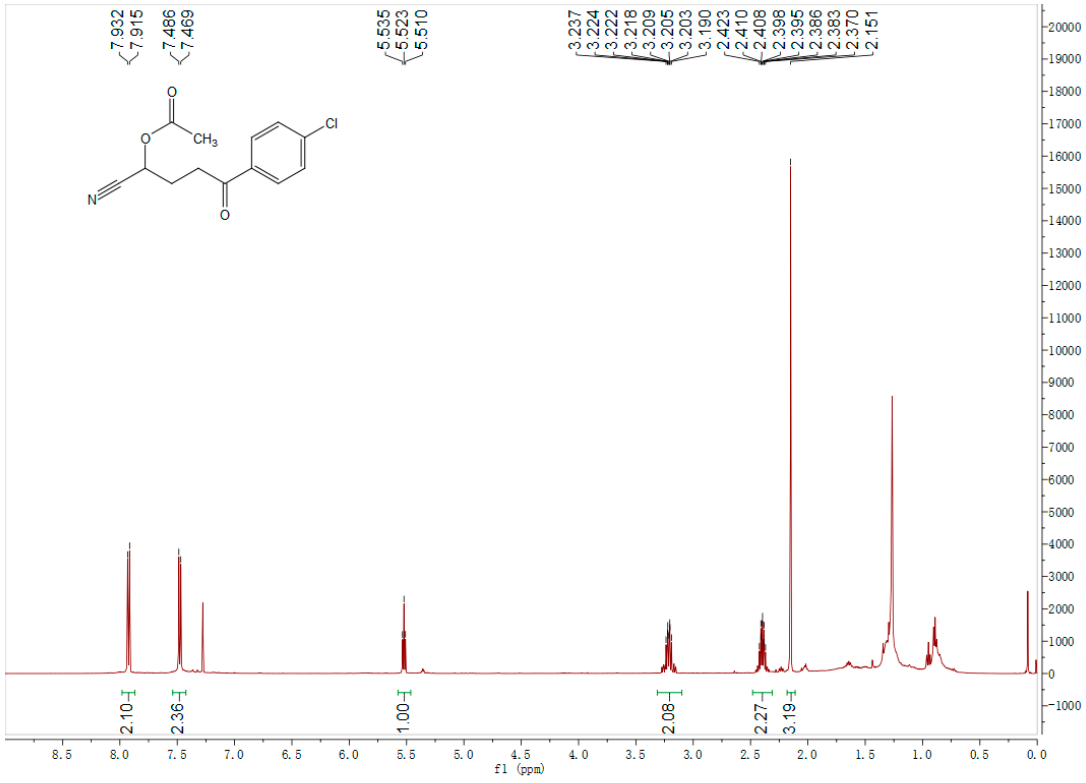

Figure S48 ^{1}H NMR spectra of **3x**

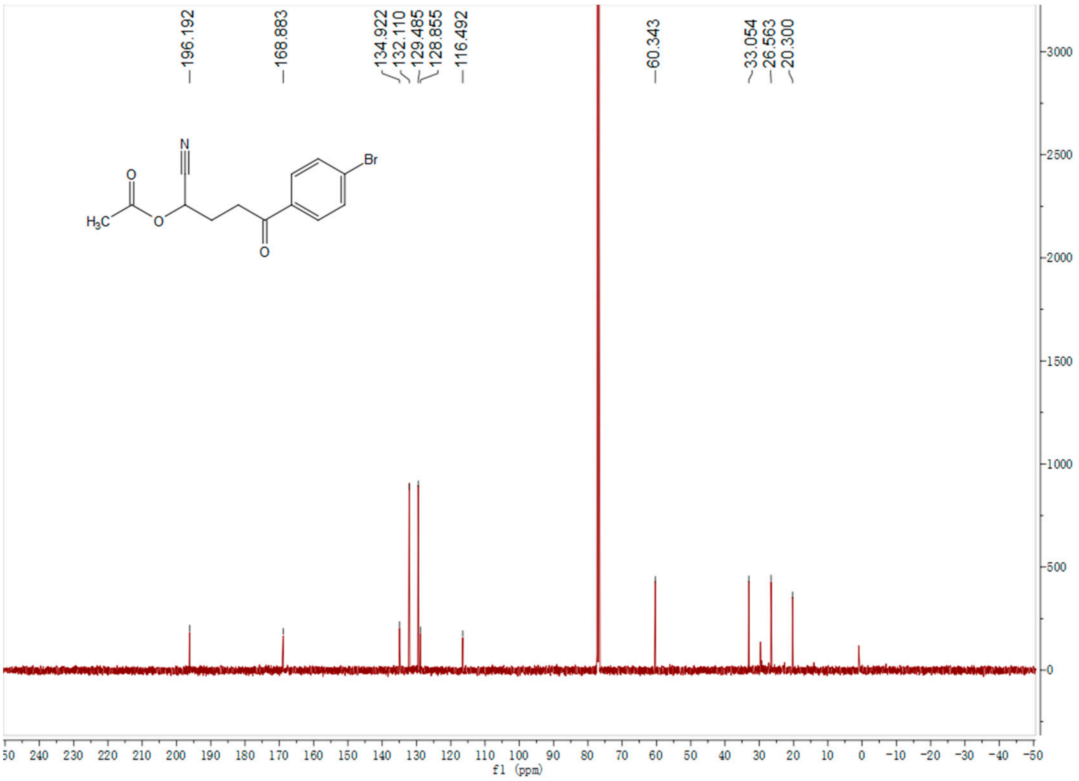

Figure S49 ^{13}C NMR spectra of **3y**

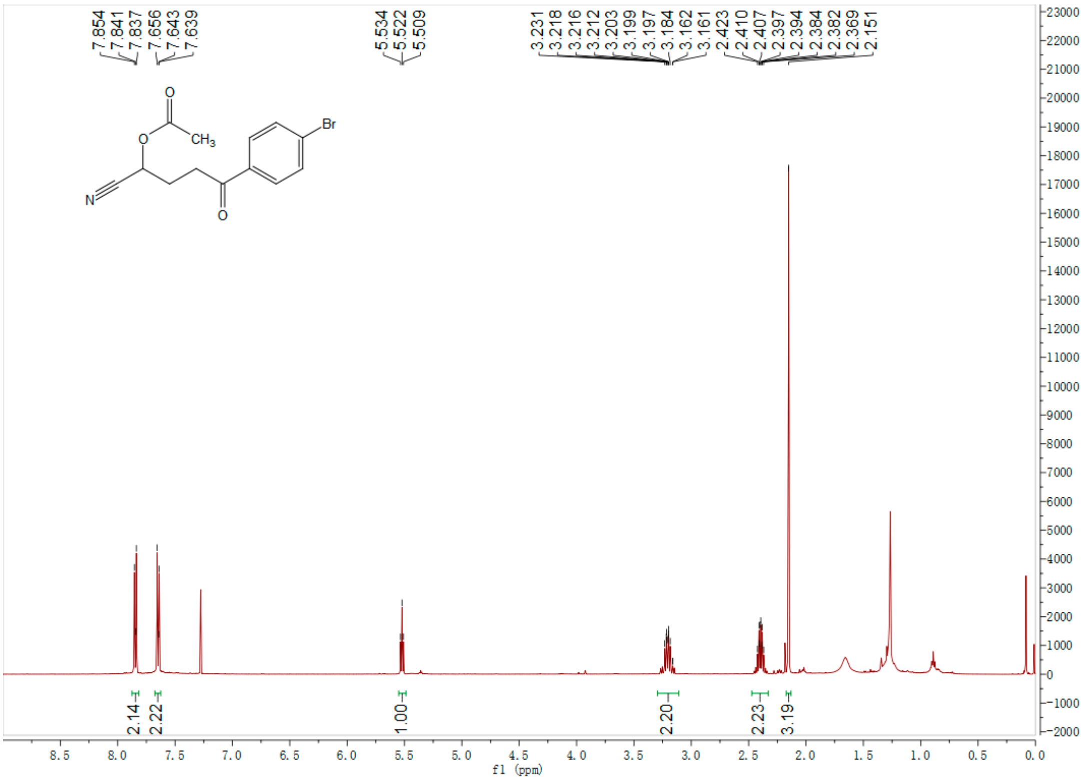

Figure S50 ^{1}H NMR spectra of **3y**

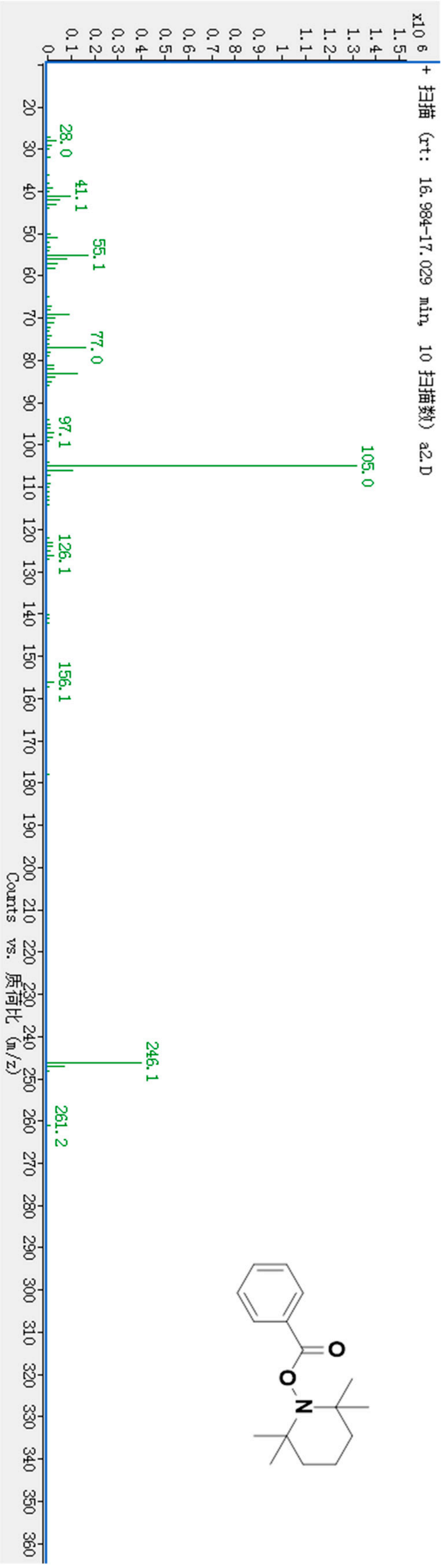

Figure S51 GC-MC of **6a**

2. ^{1}H and ^{13}C NMR spectra of compounds **4.**

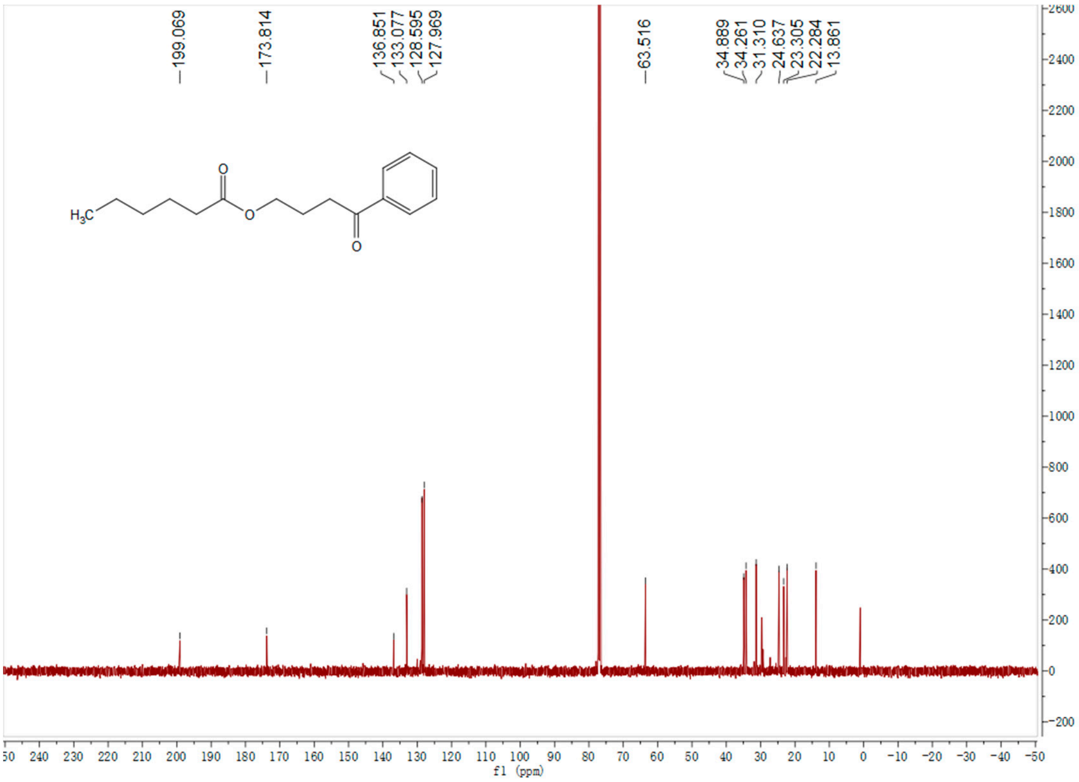

Figure S52 ^{13}C NMR spectra of **4a**

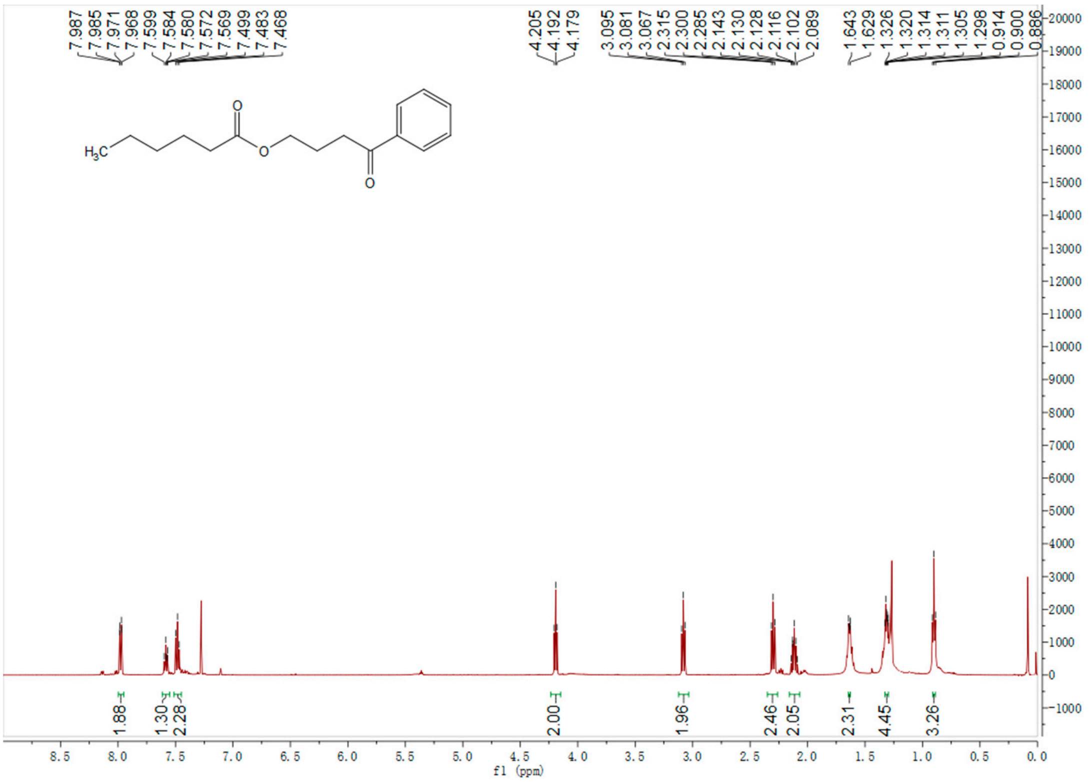

Figure S53 ^{1}H NMR spectra of **4a**

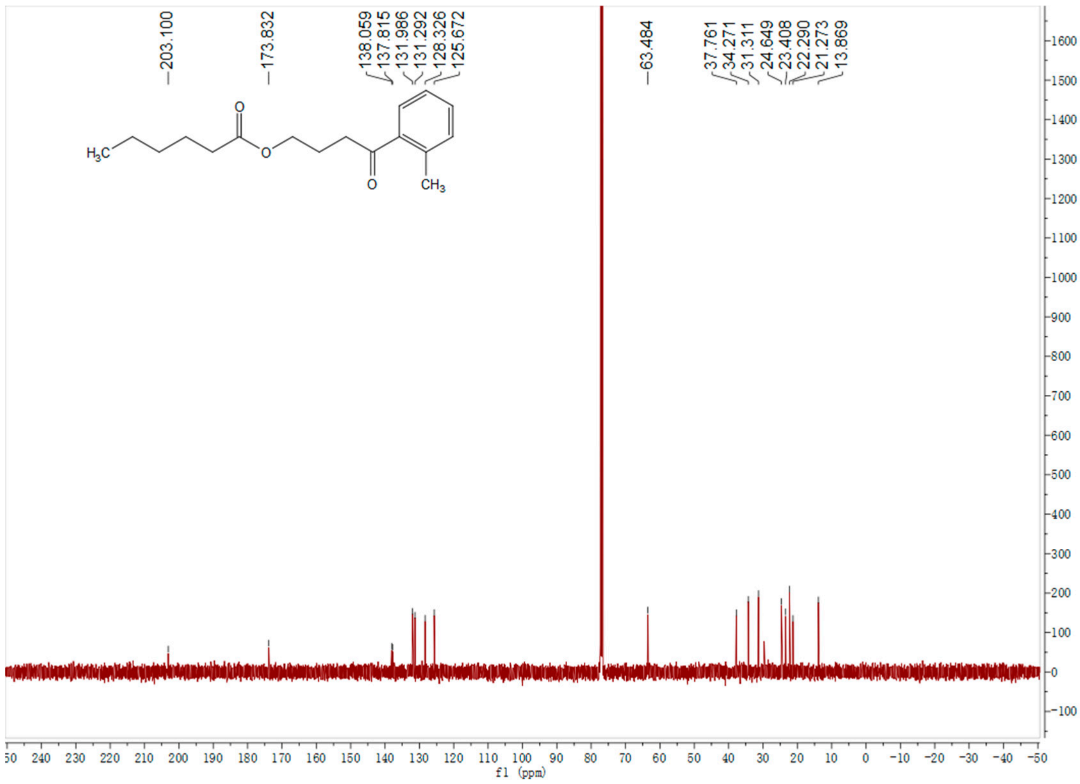

Figure S54 ^{13}C NMR spectra of **4b**

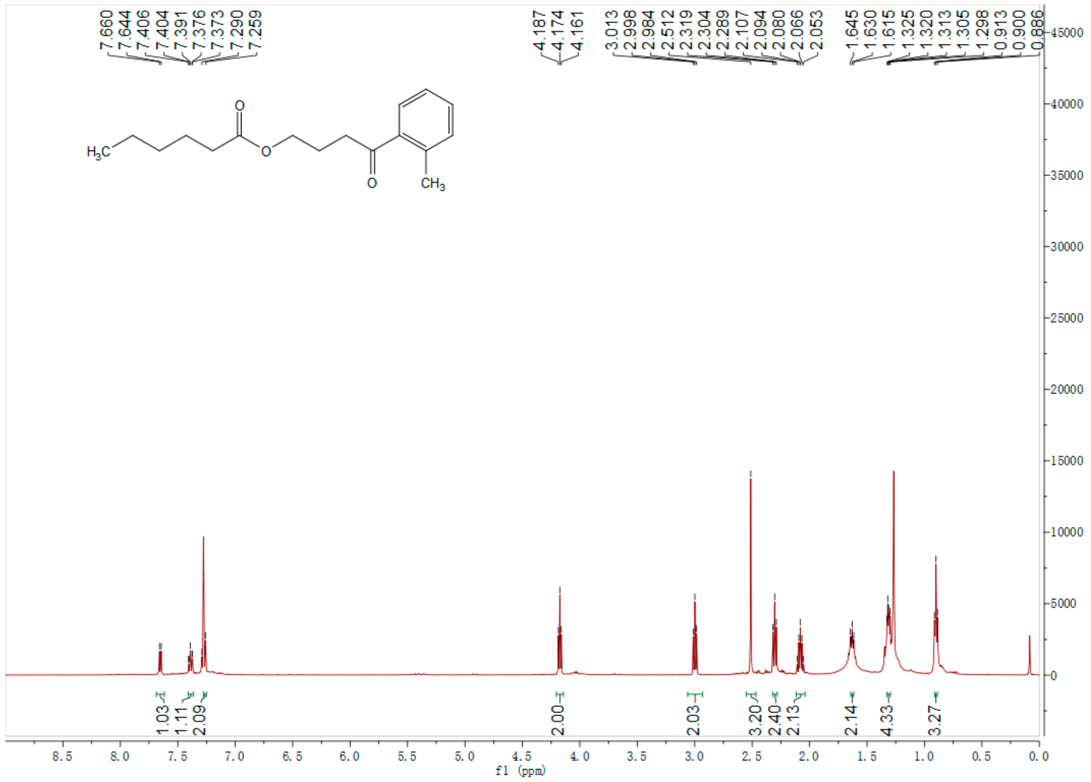

Figure S55 ^{1}H NMR spectra of **4b**

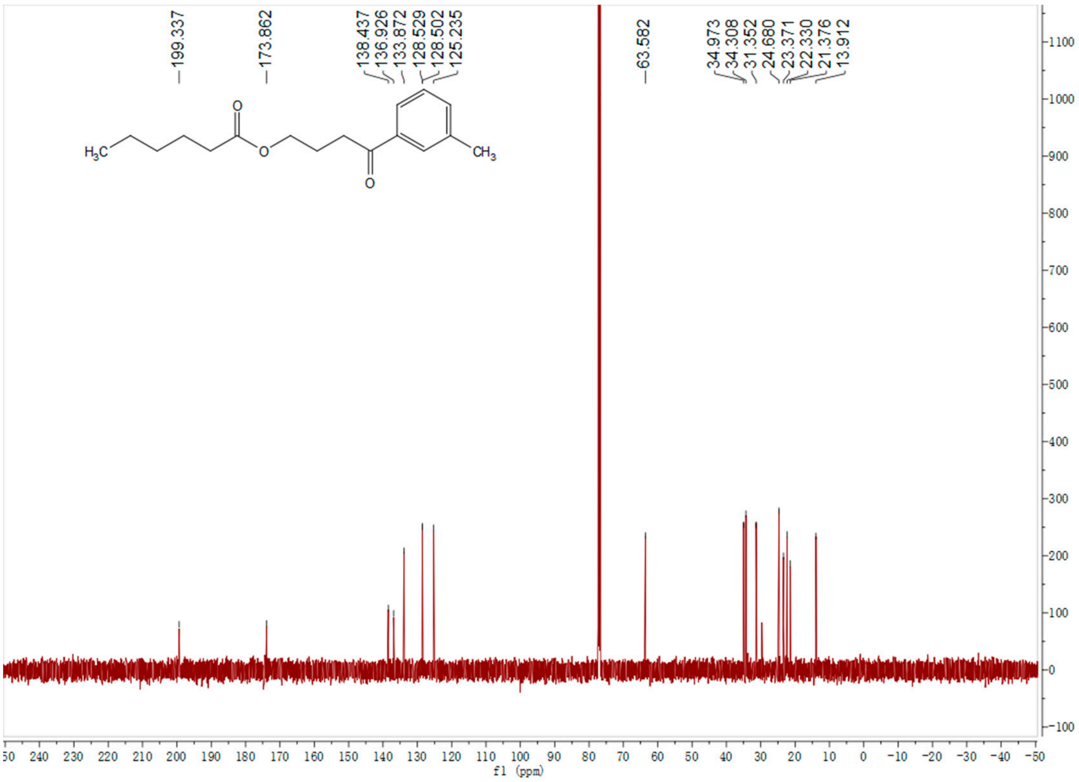

Figure S56 ^{13}C NMR spectra of **4c**

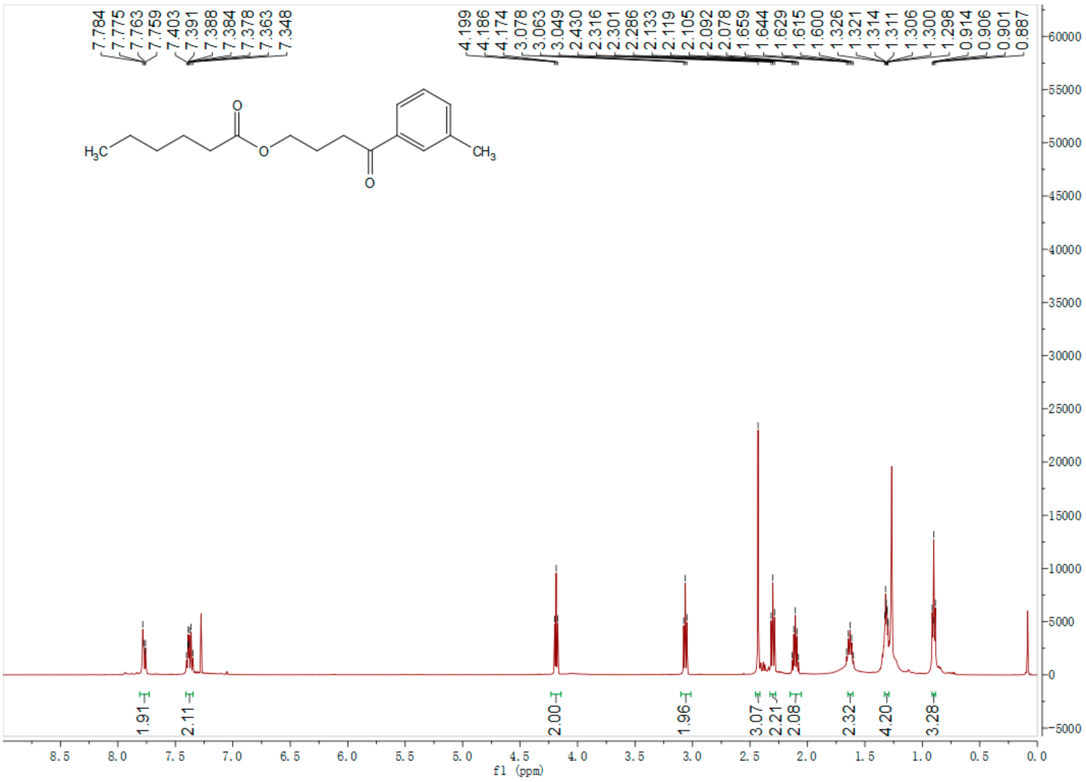

Figure S57 ^{1}H NMR spectra of **4c**

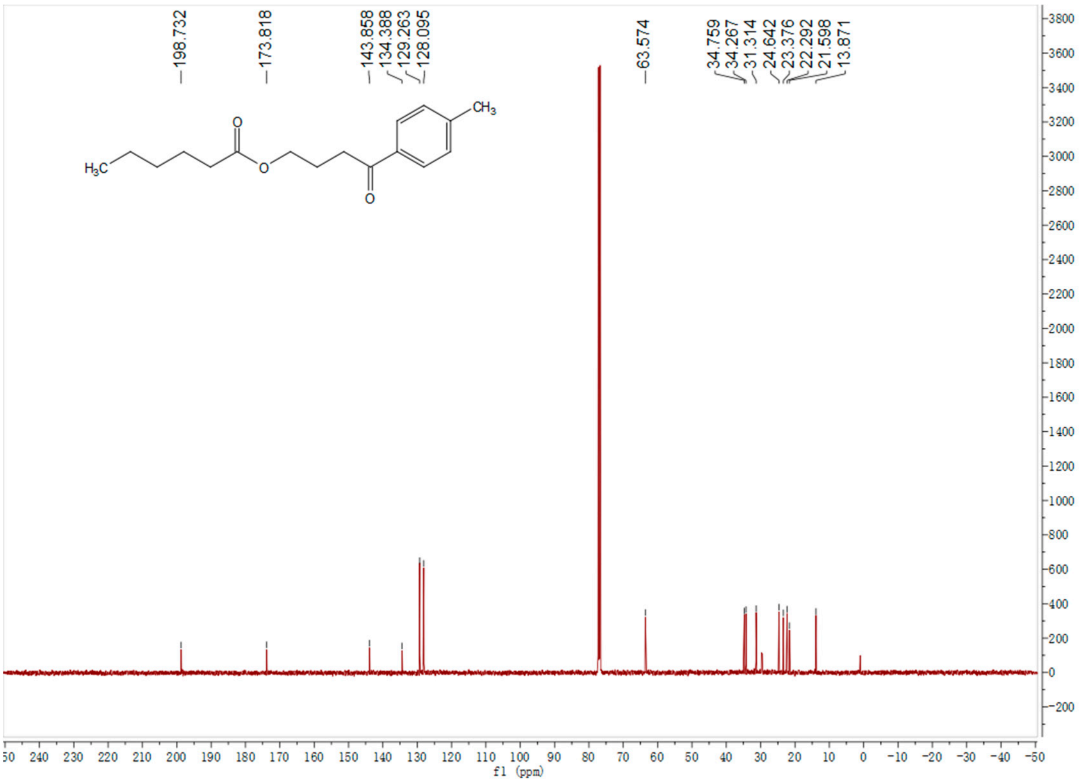

Figure S58 ^{13}C NMR spectra of **4d**

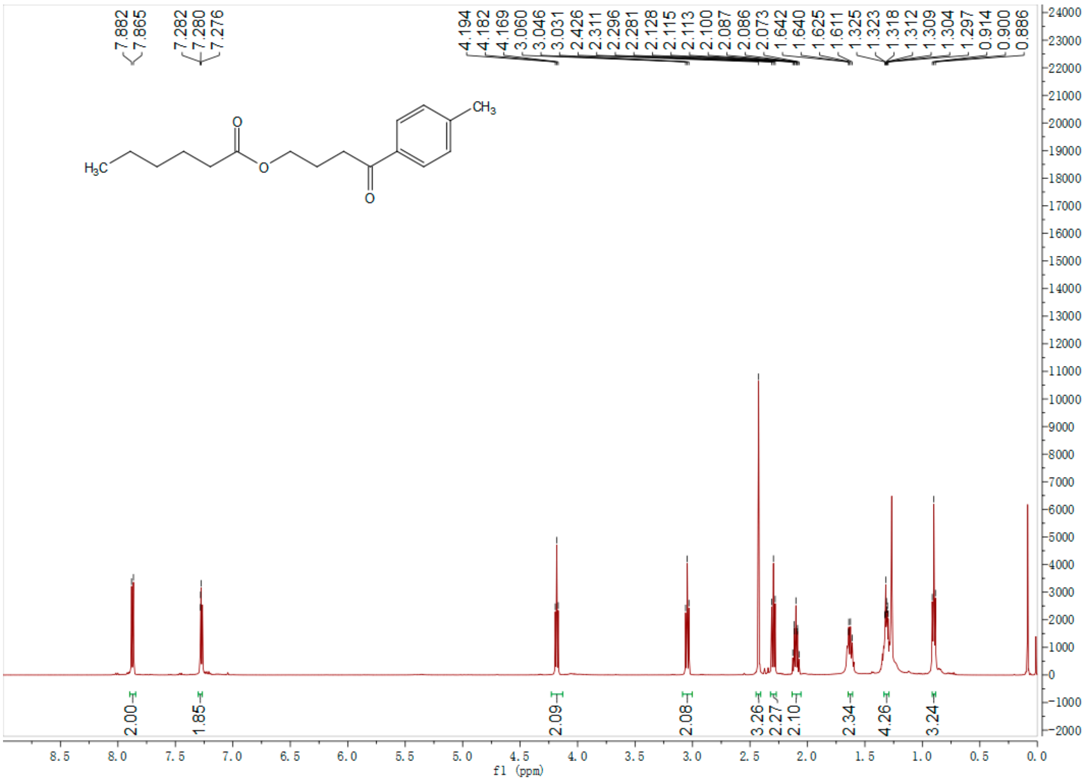

Figure S59 ^{1}H NMR spectra of **4d**

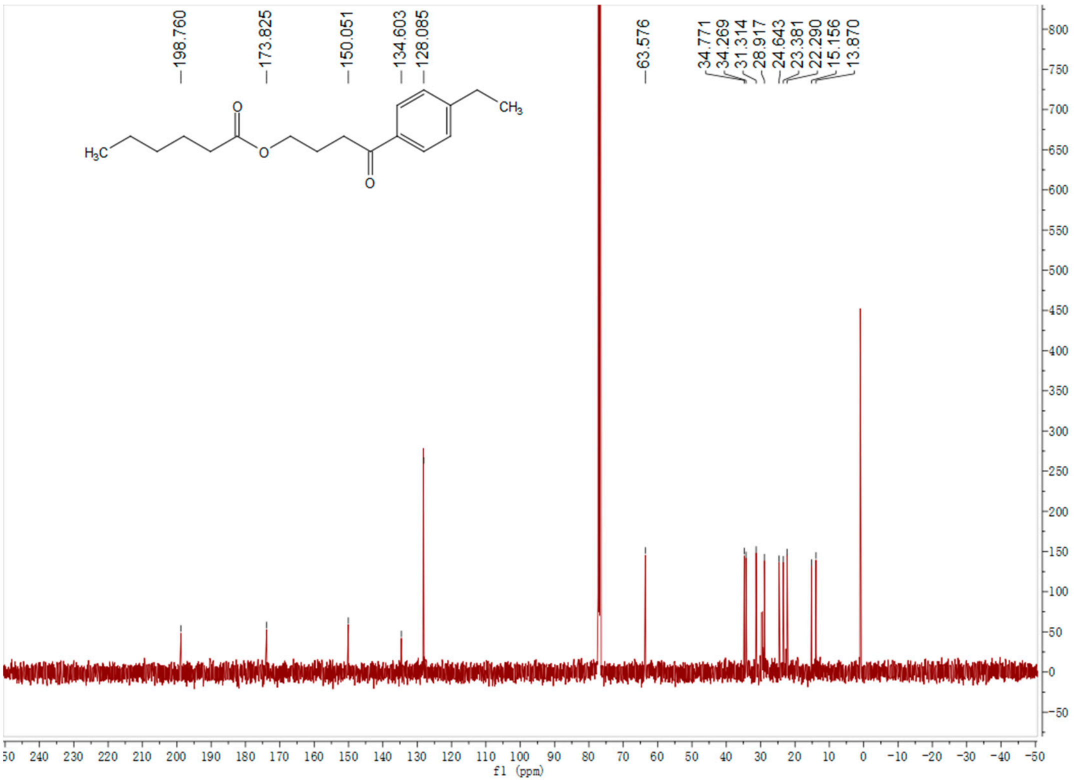

Figure S60 ^{13}C NMR spectra of **4e**

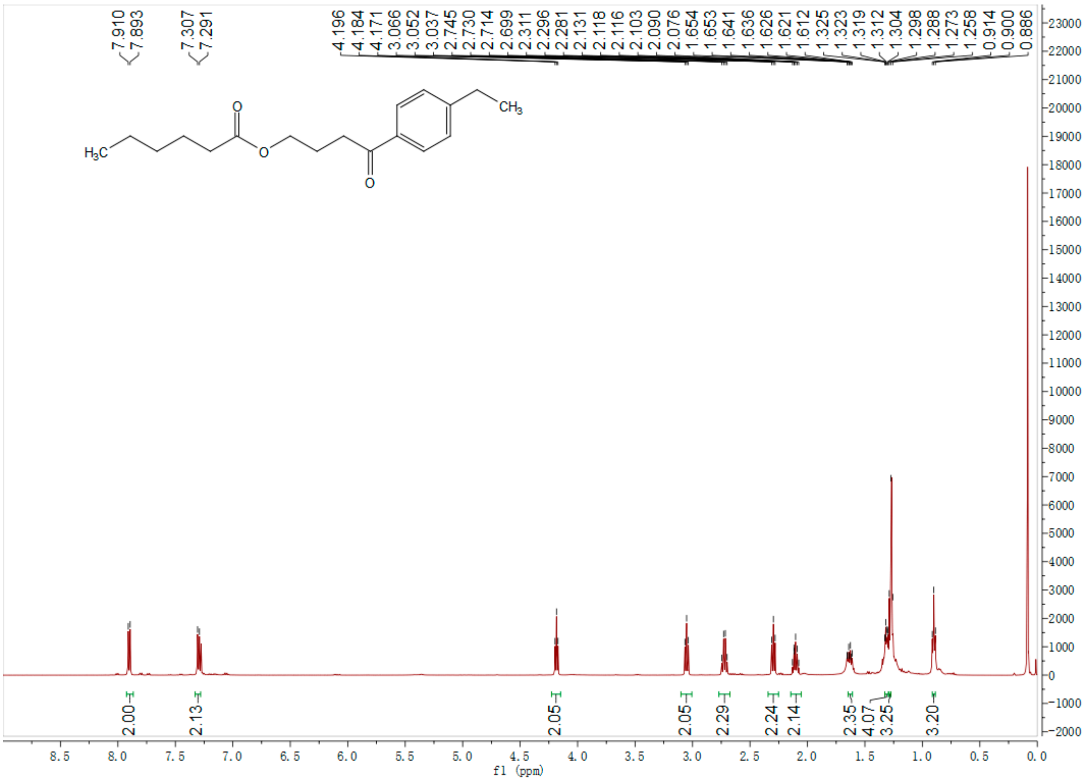

Figure S61 ^{1}H NMR spectra of **4e**

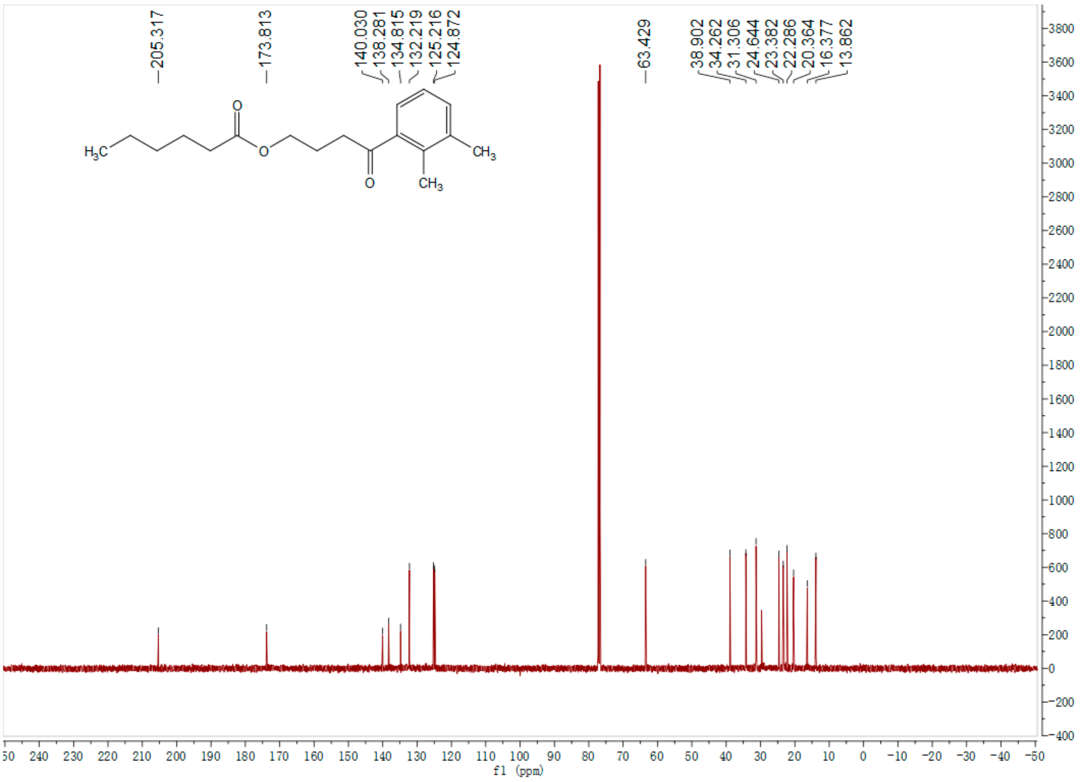

Figure S62 ^{13}C NMR spectra of **4f**

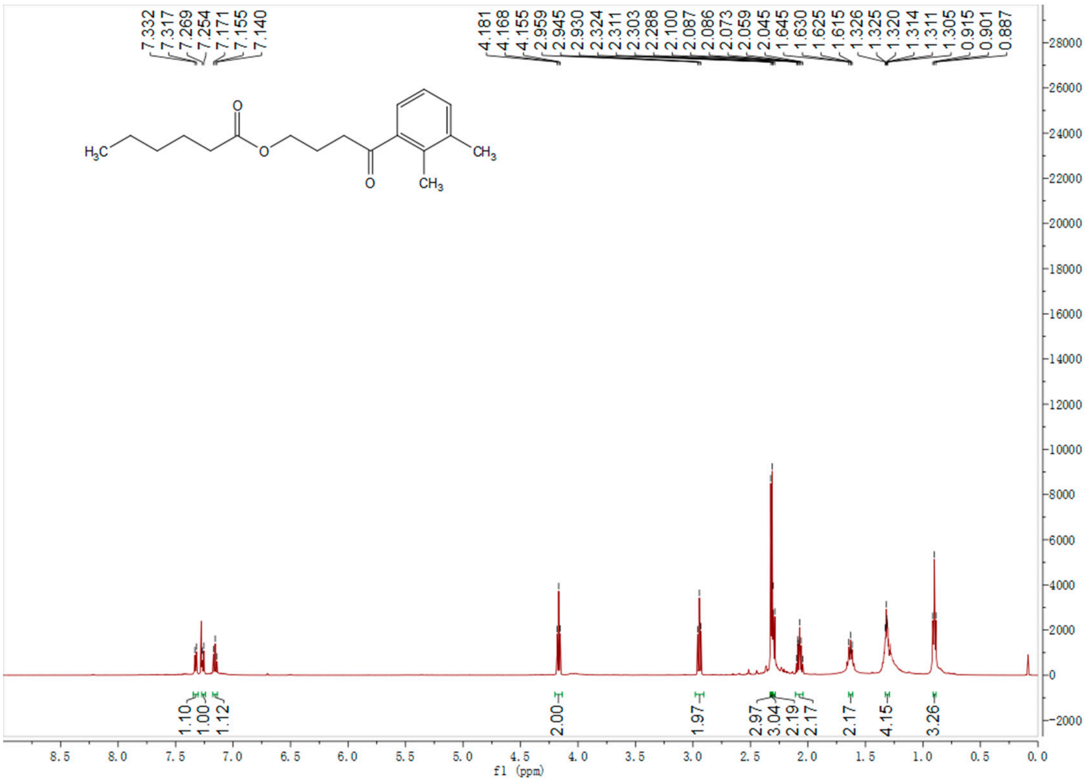

Figure S63 ^{1}H NMR spectra of **4f**

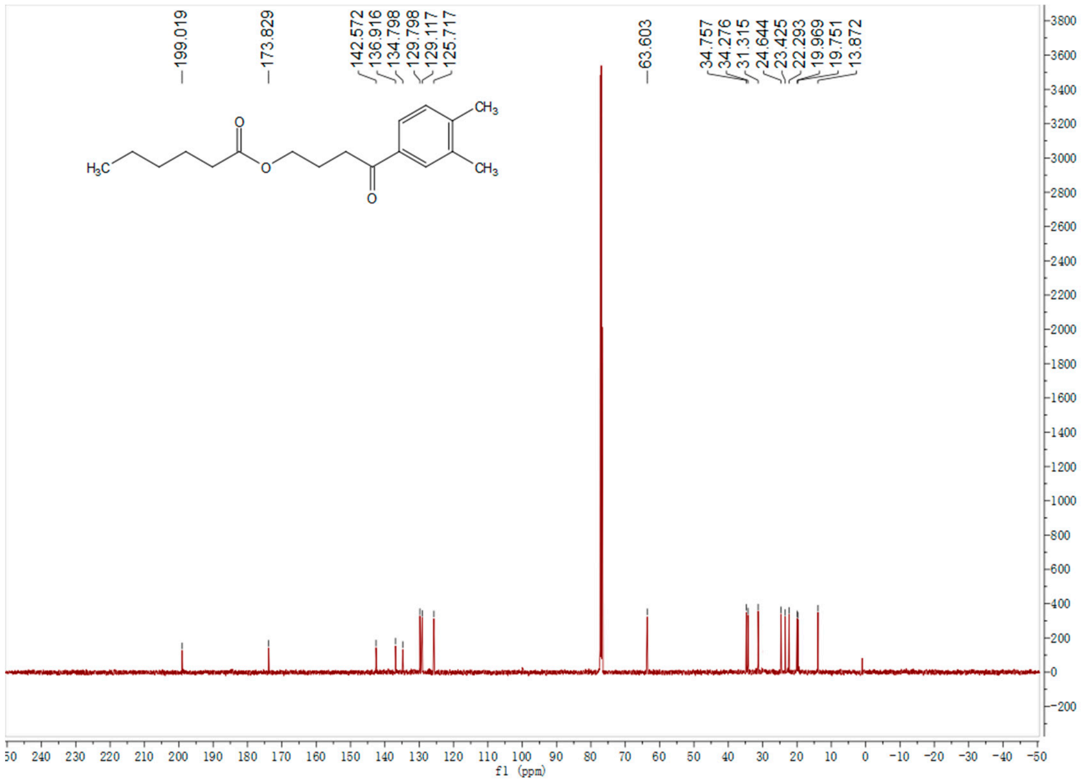

Figure S64 ^{13}C NMR spectra of **4g**

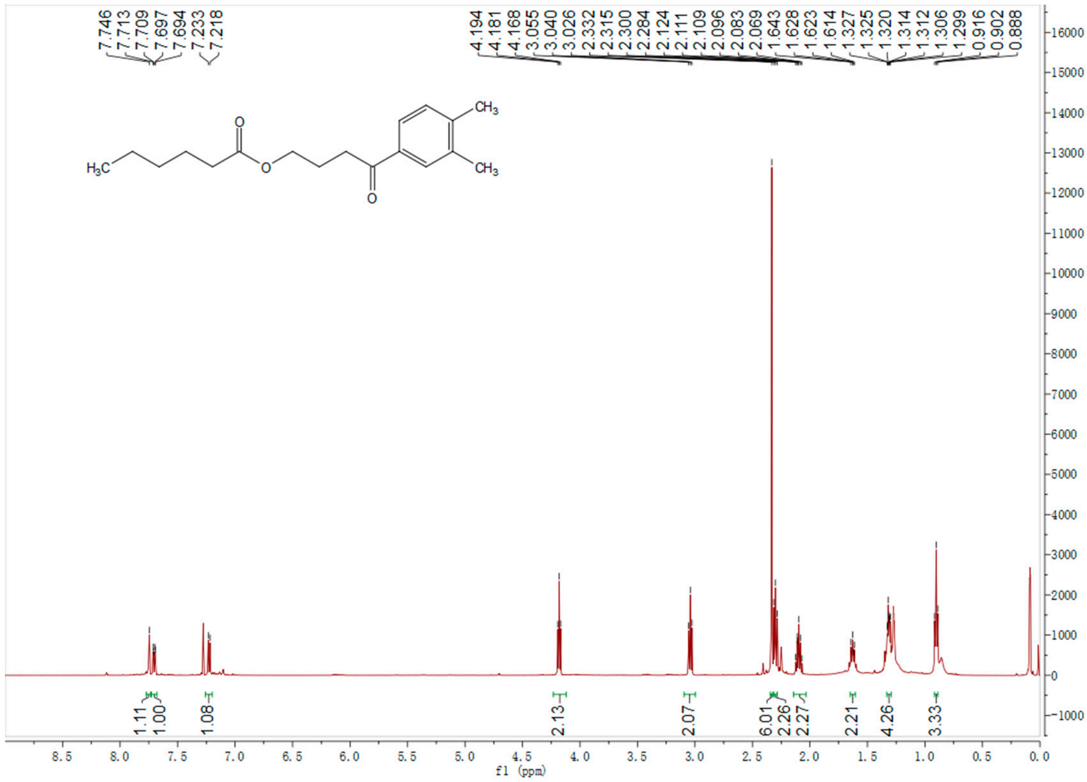

Figure S65 ^{1}H NMR spectra of **4g**

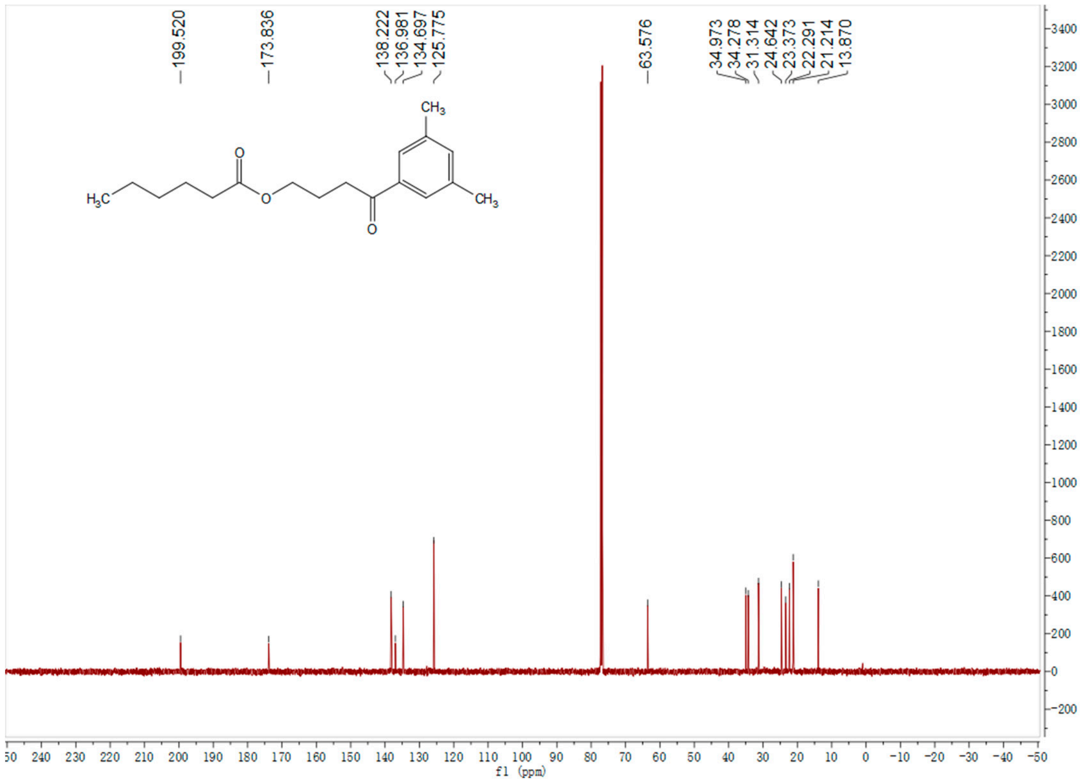

Figure S66 ^{13}C NMR spectra of **4h**

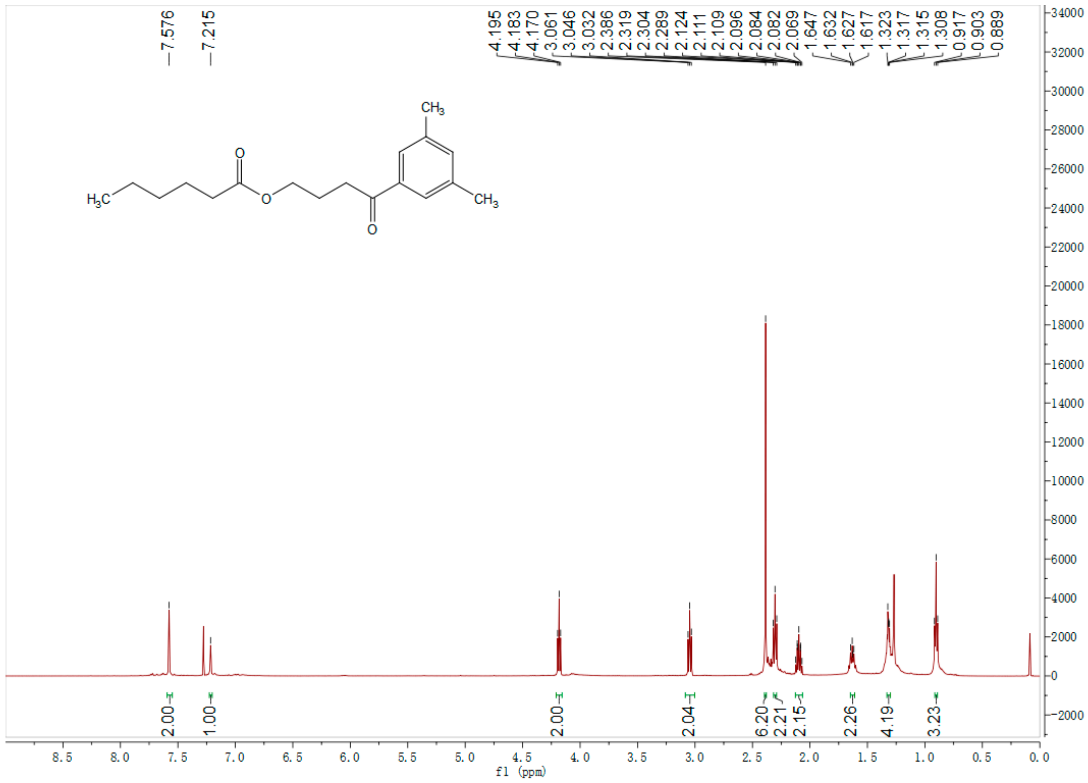

Figure S67 ^{1}H NMR spectra of **4h**

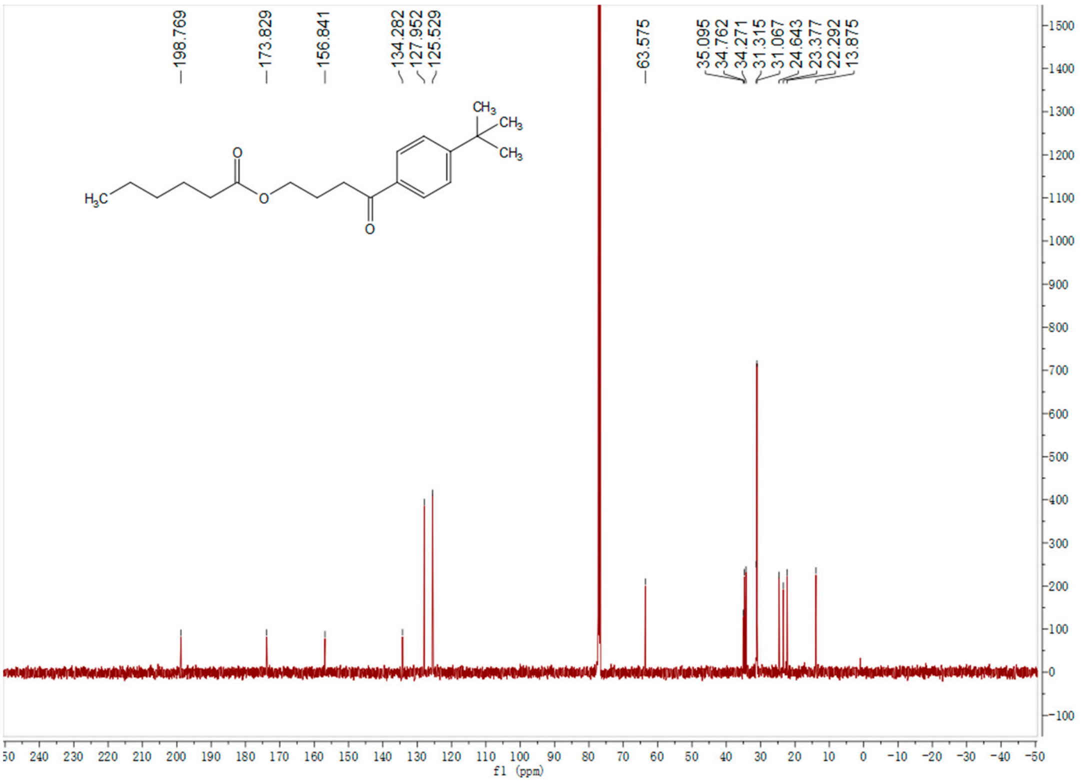

Figure S68 ^{13}C NMR spectra of **4i**

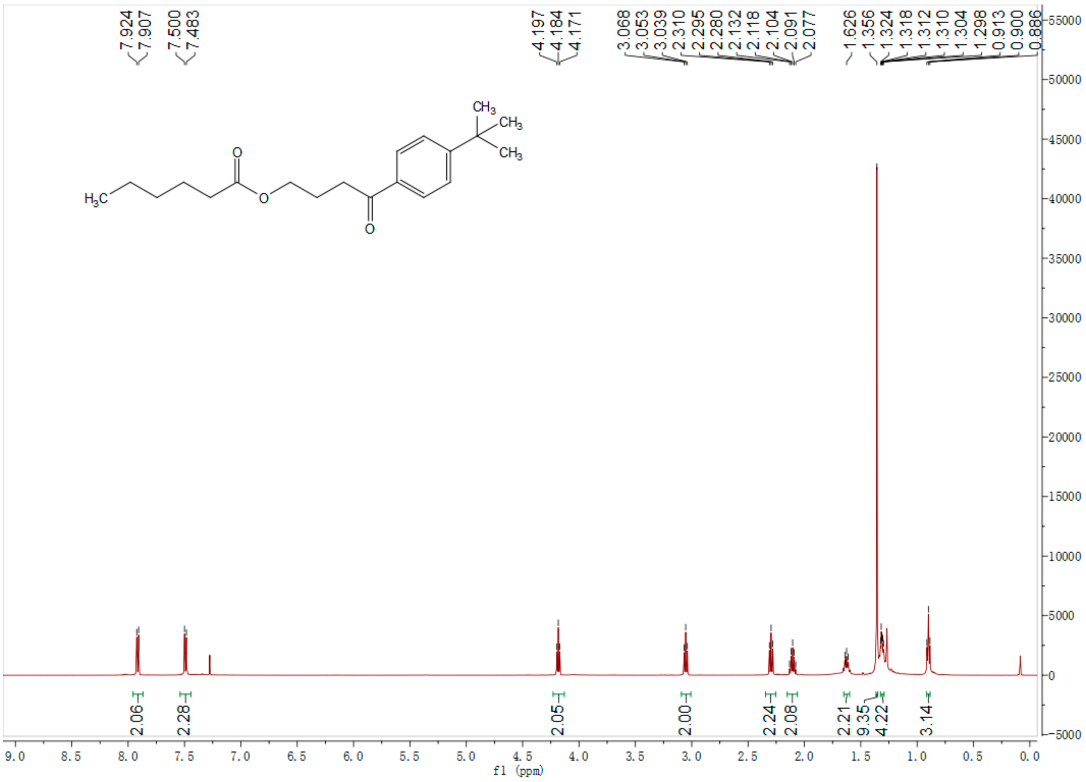

Figure S69 ^{1}H NMR spectra of **4i**

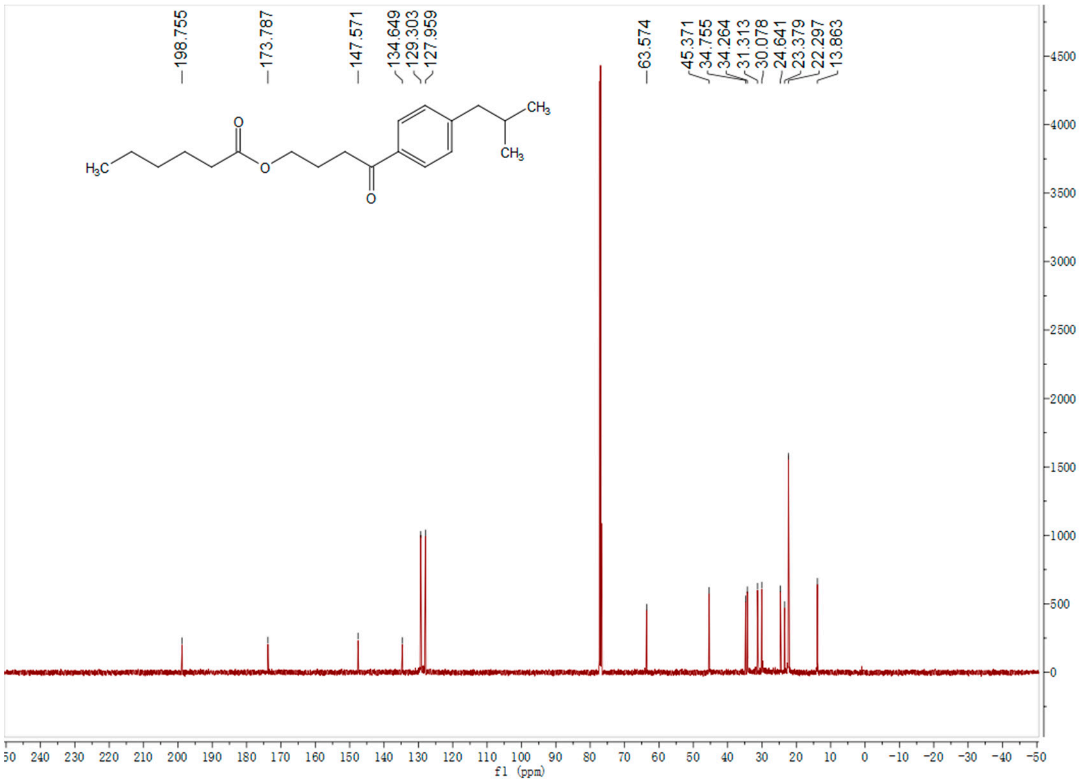

Figure S70 ^{13}C NMR spectra of **4j**

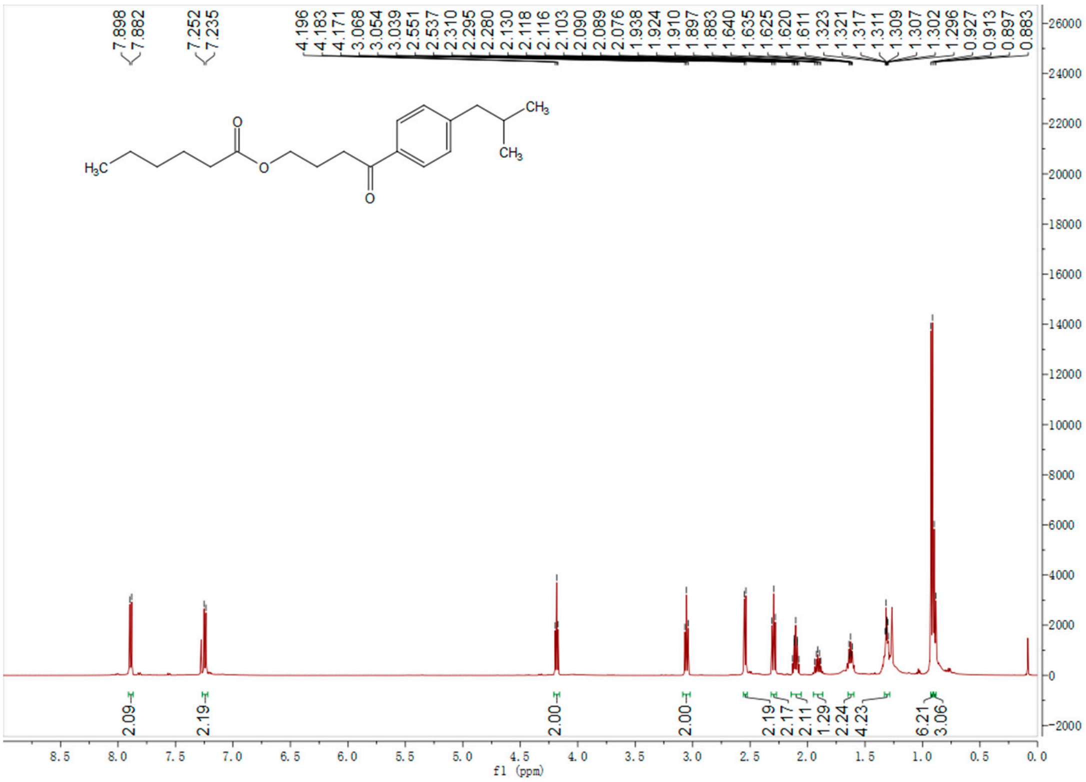

Figure S71 ^{1}H NMR spectra of **4j**

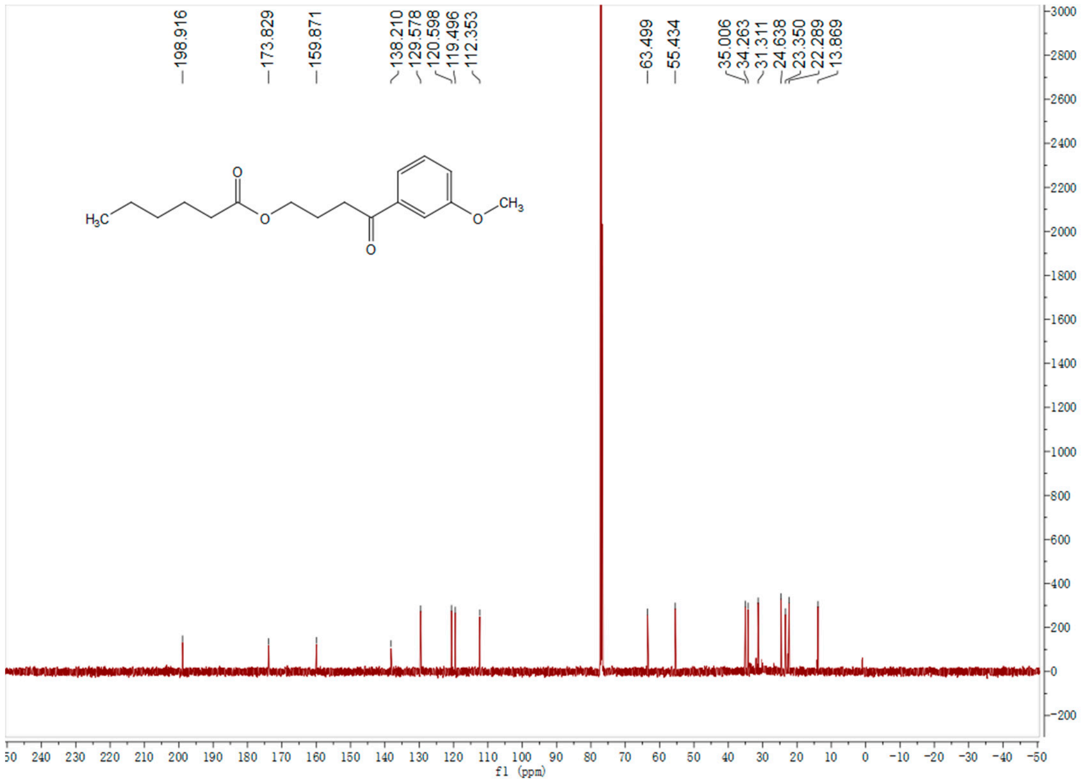

Figure S72 ^{13}C NMR spectra of **4k**

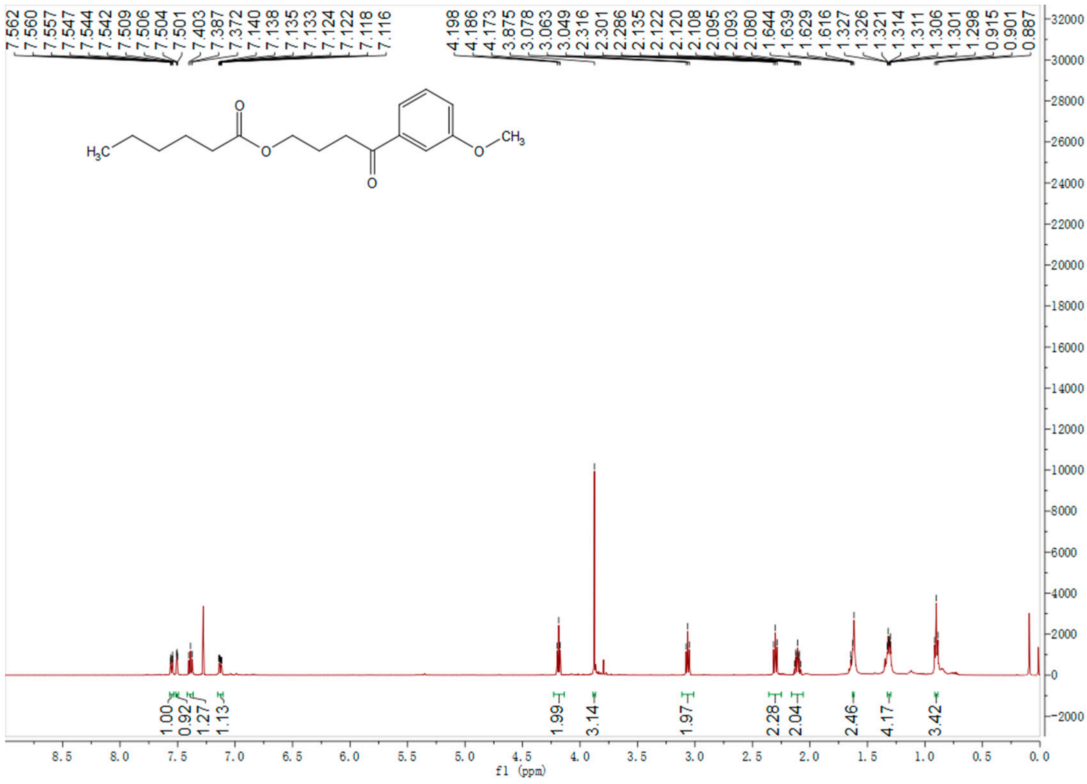

Figure S73 ^{1}H NMR spectra of **4k**

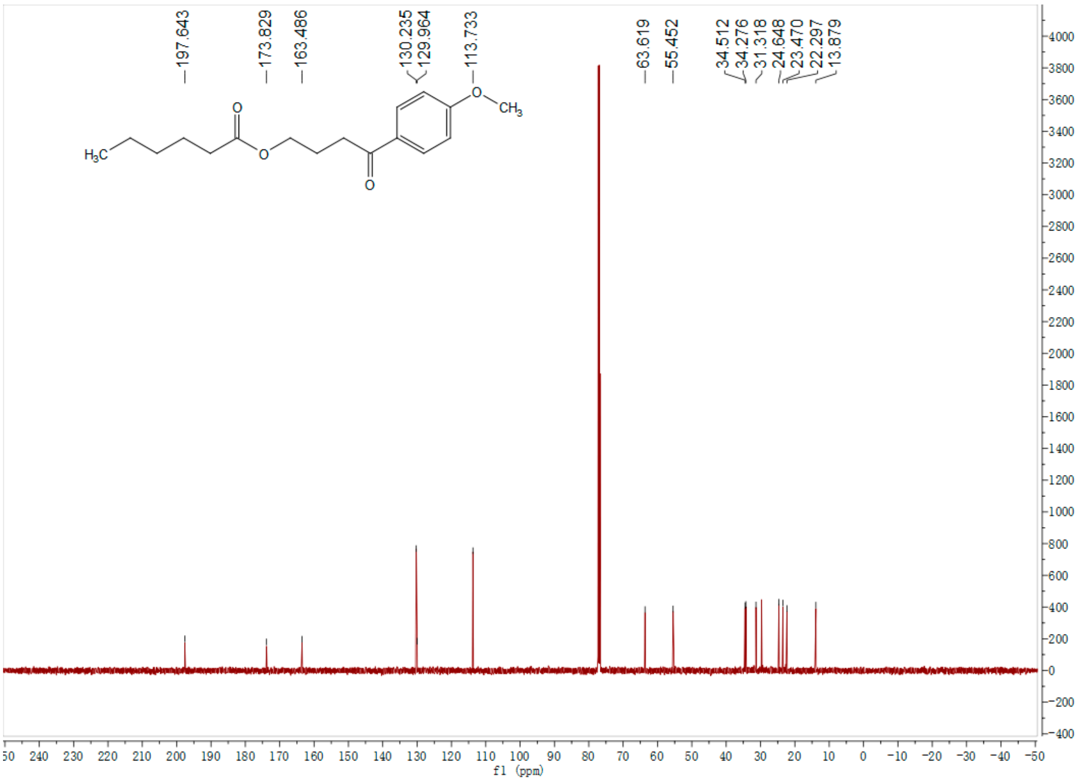

Figure S74 ^{13}C NMR spectra of **4l**

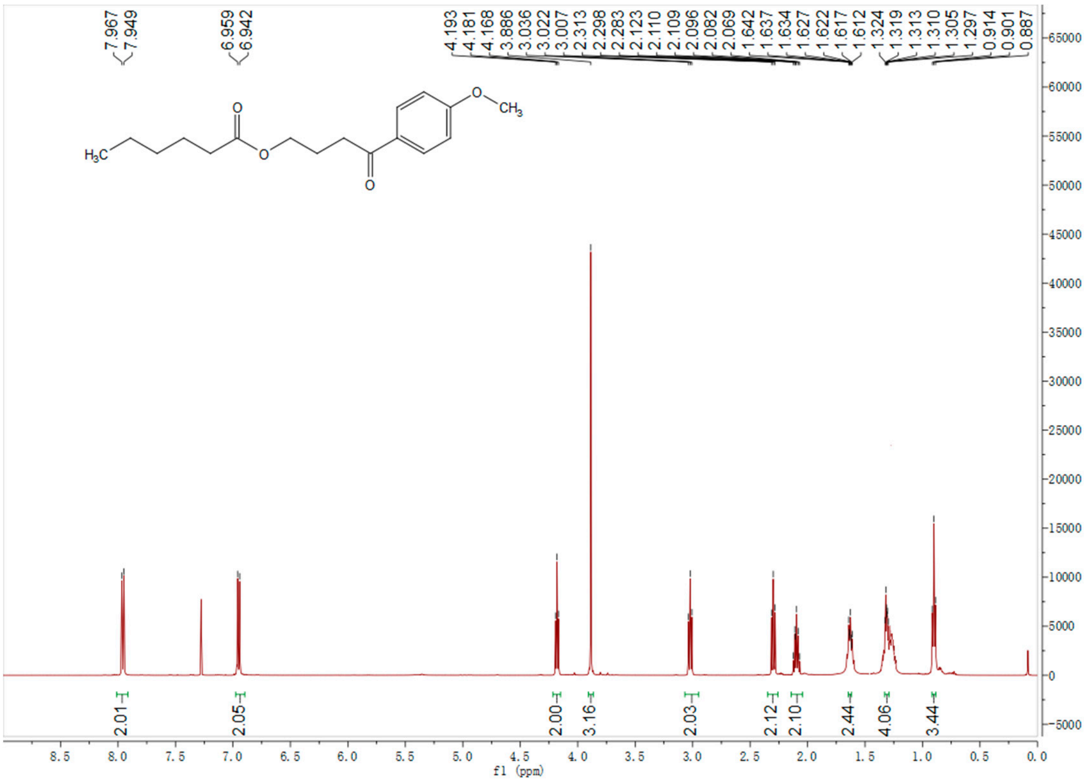

Figure S75 ^{1}H NMR spectra of **4l**

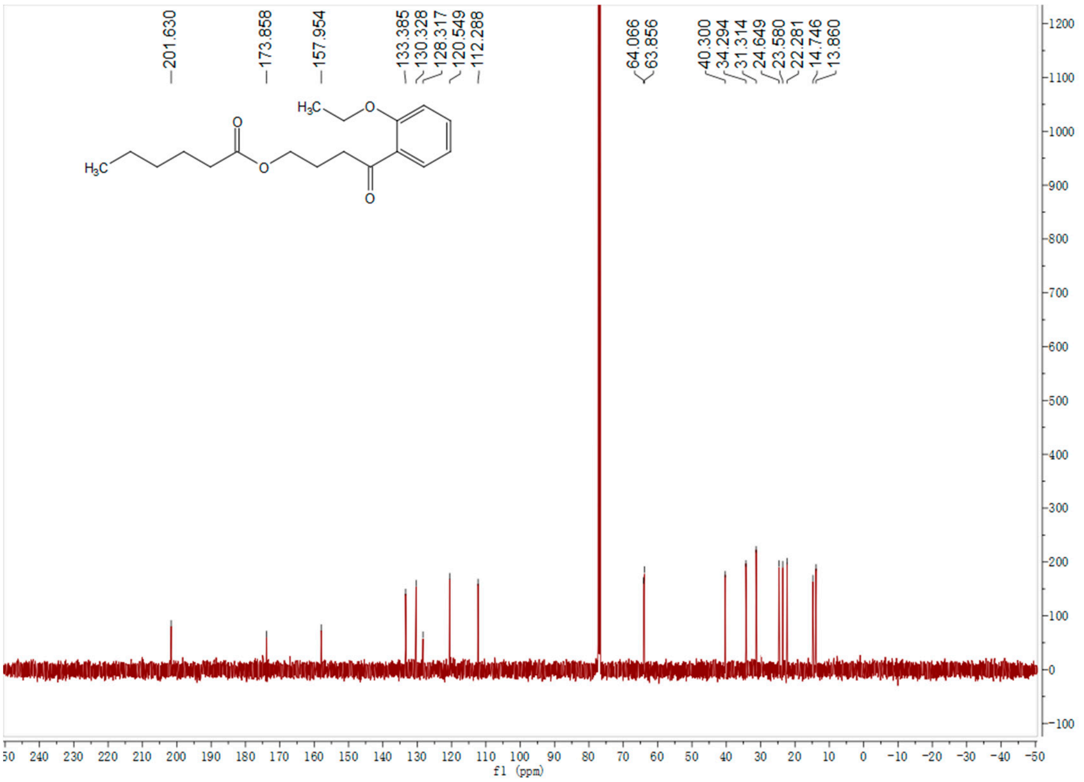

Figure S76 ^{13}C NMR spectra of **4m**

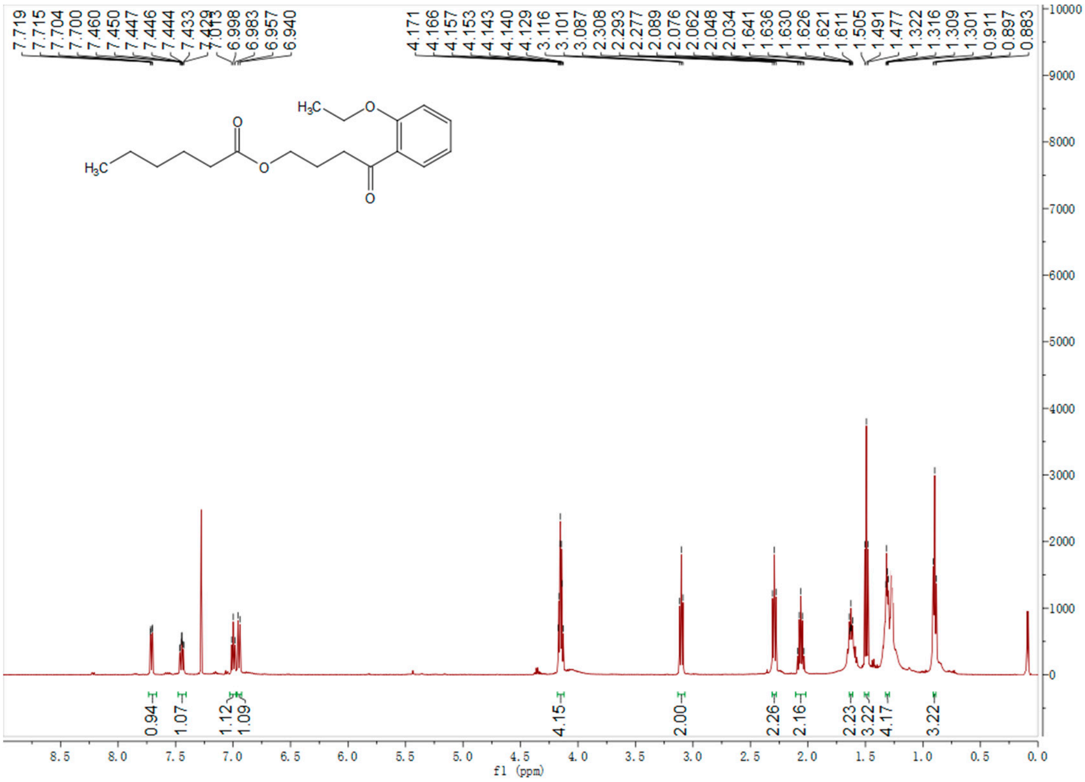

Figure S77 ^{1}H NMR spectra of **4m**

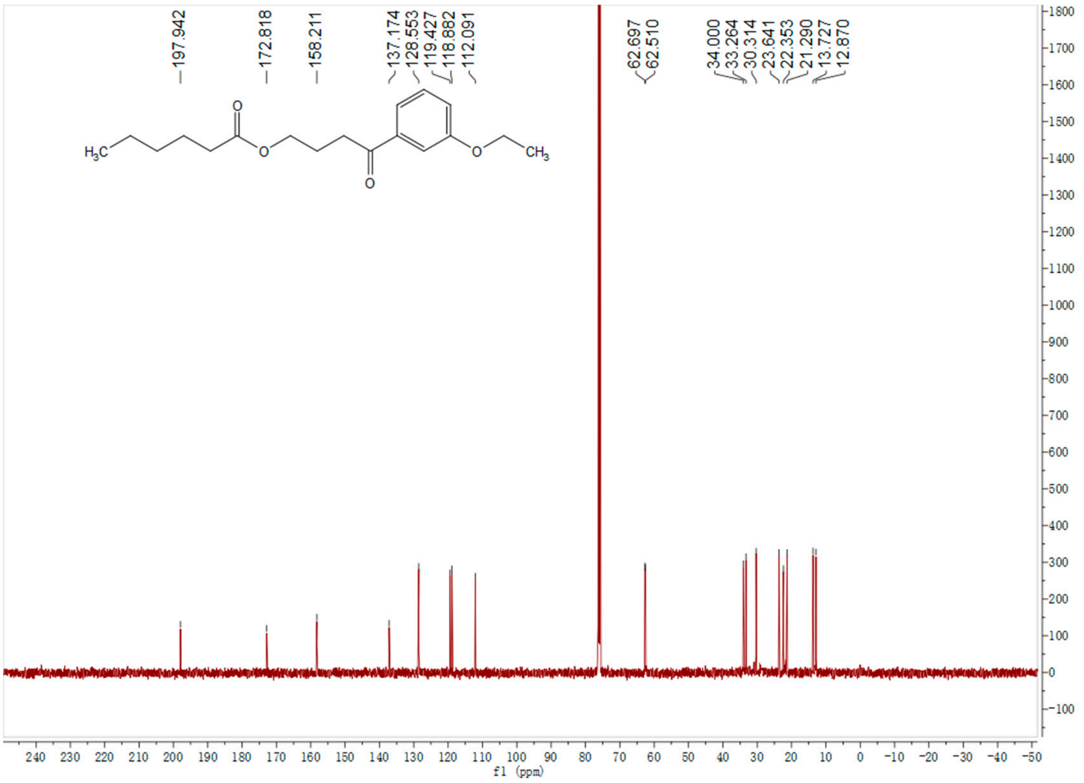

Figure S78 ^{13}C NMR spectra of **4n**

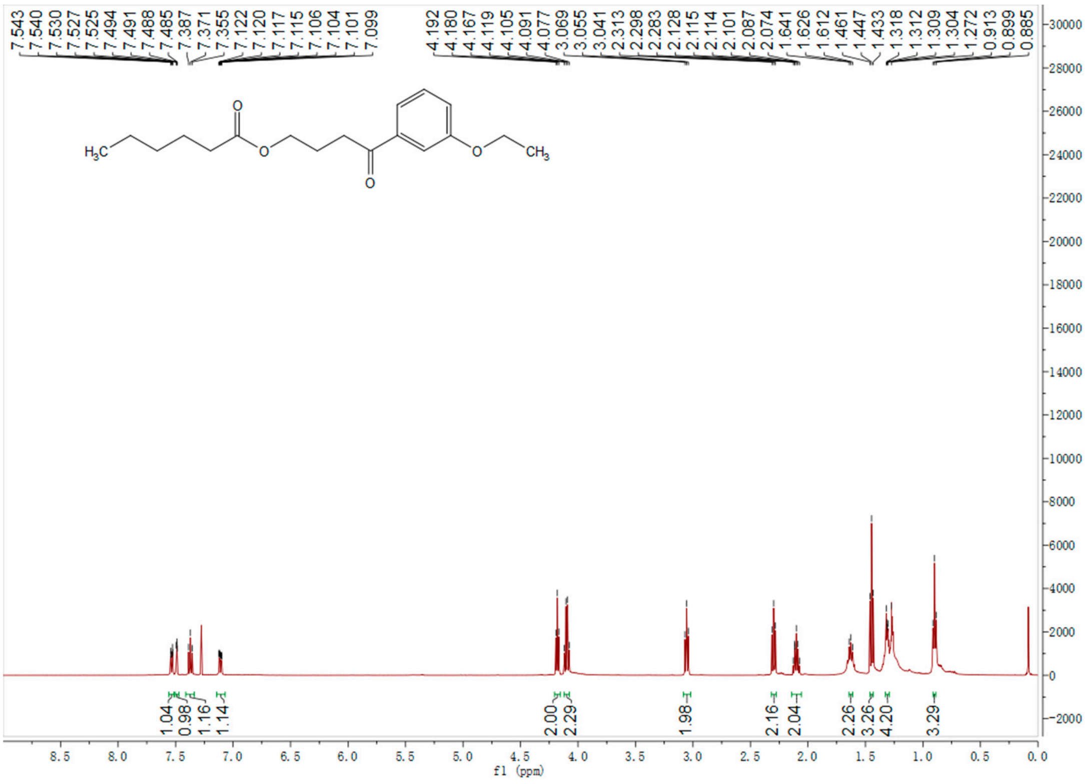

Figure S79 ^{1}H NMR spectra of **4n**

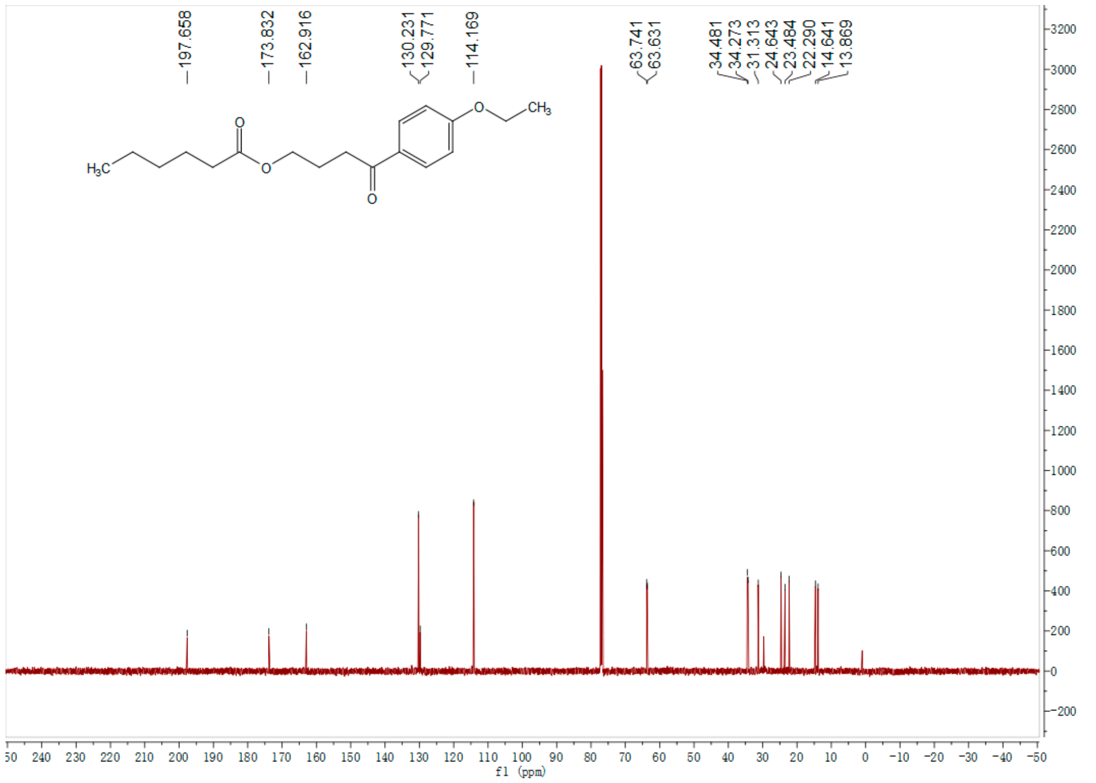

Figure S80 ^{13}C NMR spectra of **4o**

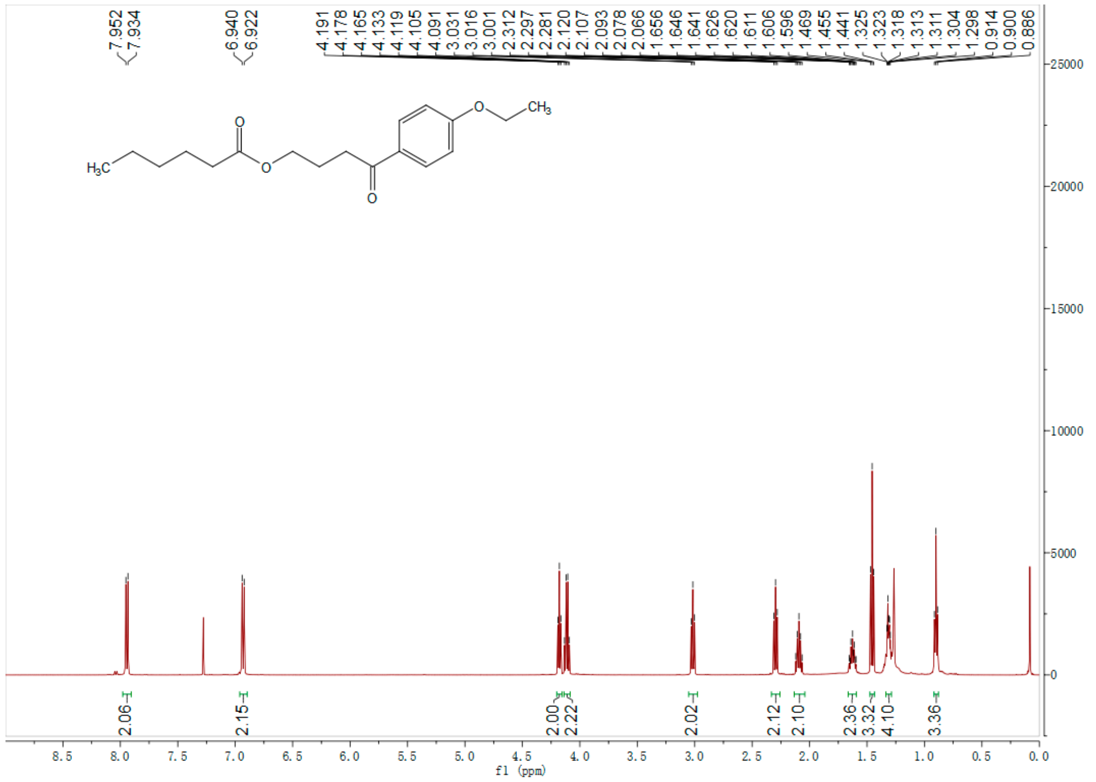

Figure S81 ^{1}H NMR spectra of **4o**

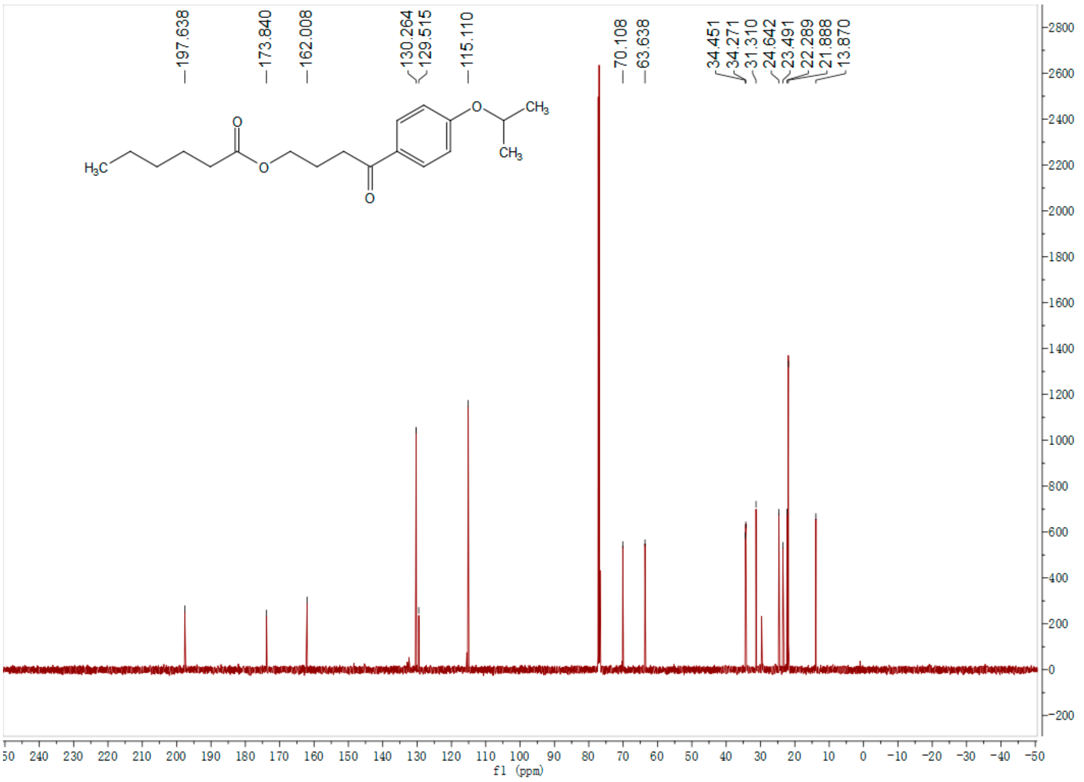

Figure S82 ^{13}C NMR spectra of **4p**

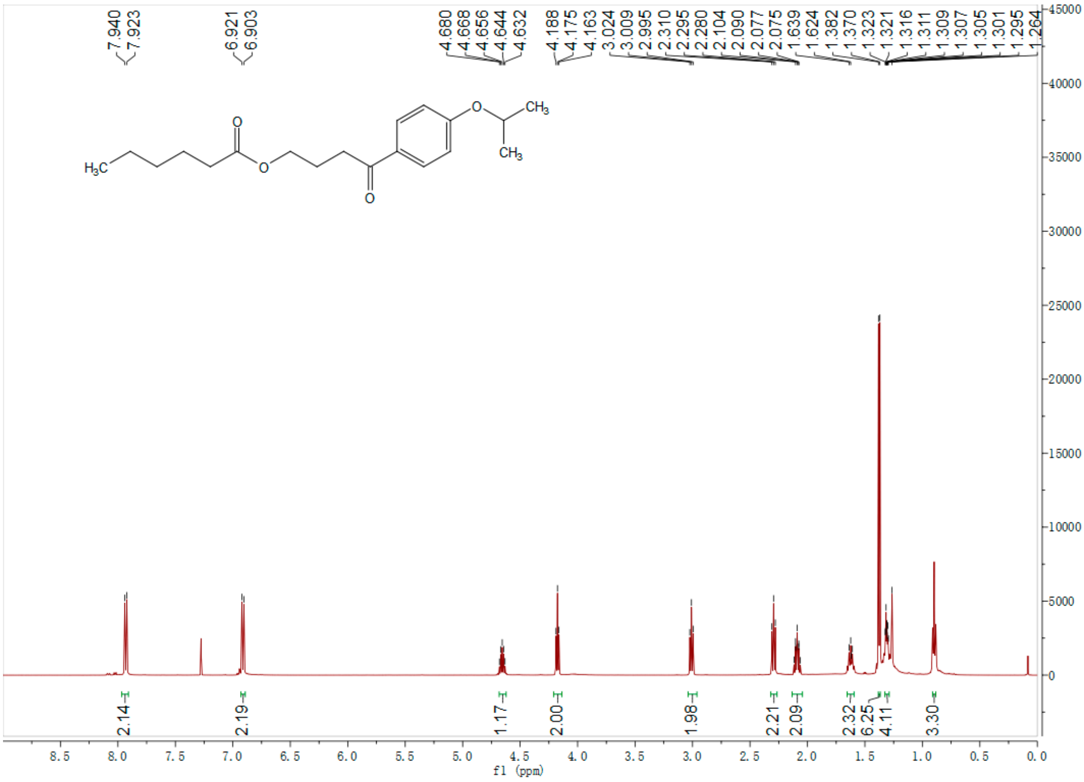

Figure S83 ^{1}H NMR spectra of **4p**

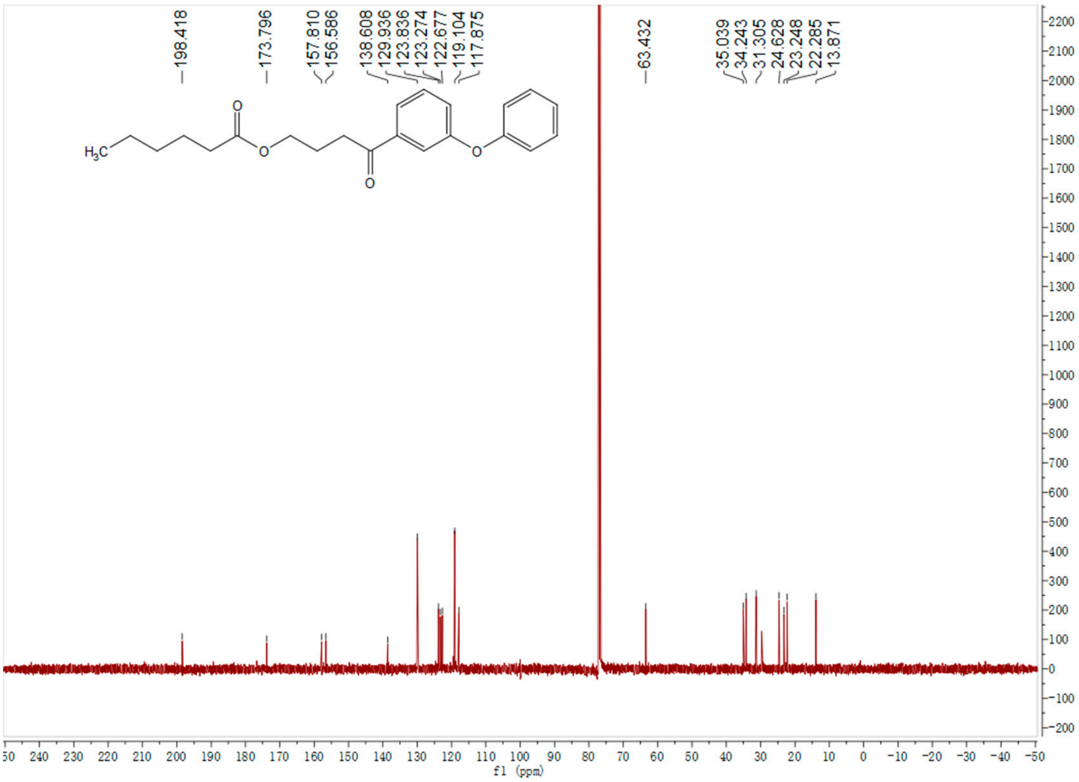

Figure S84 ^{13}C NMR spectra of **4q**

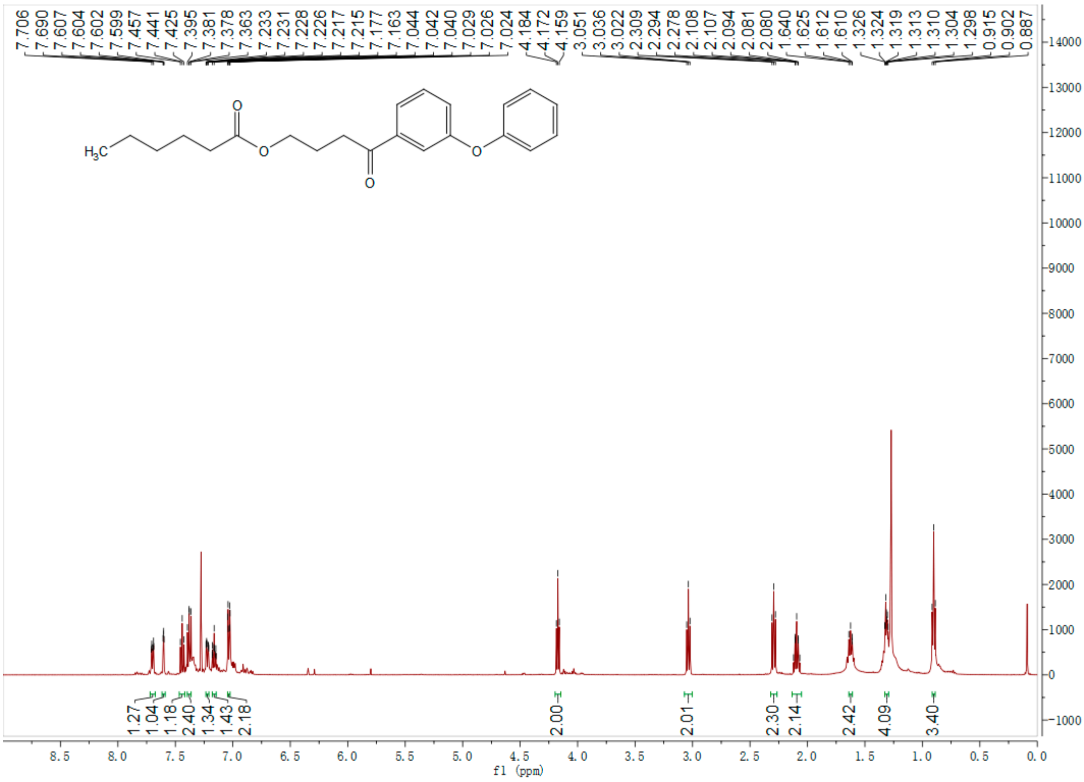

Figure S85 ^{1}H NMR spectra of **4q**

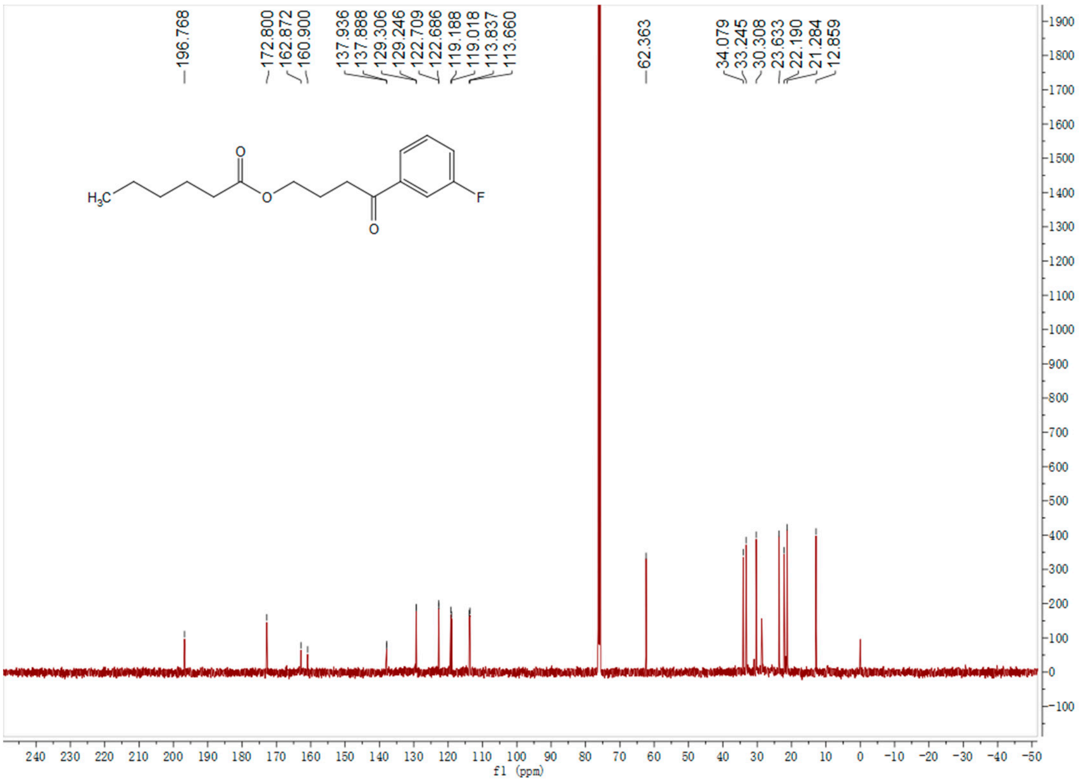

Figure S86 ^{13}C NMR spectra of **4r**

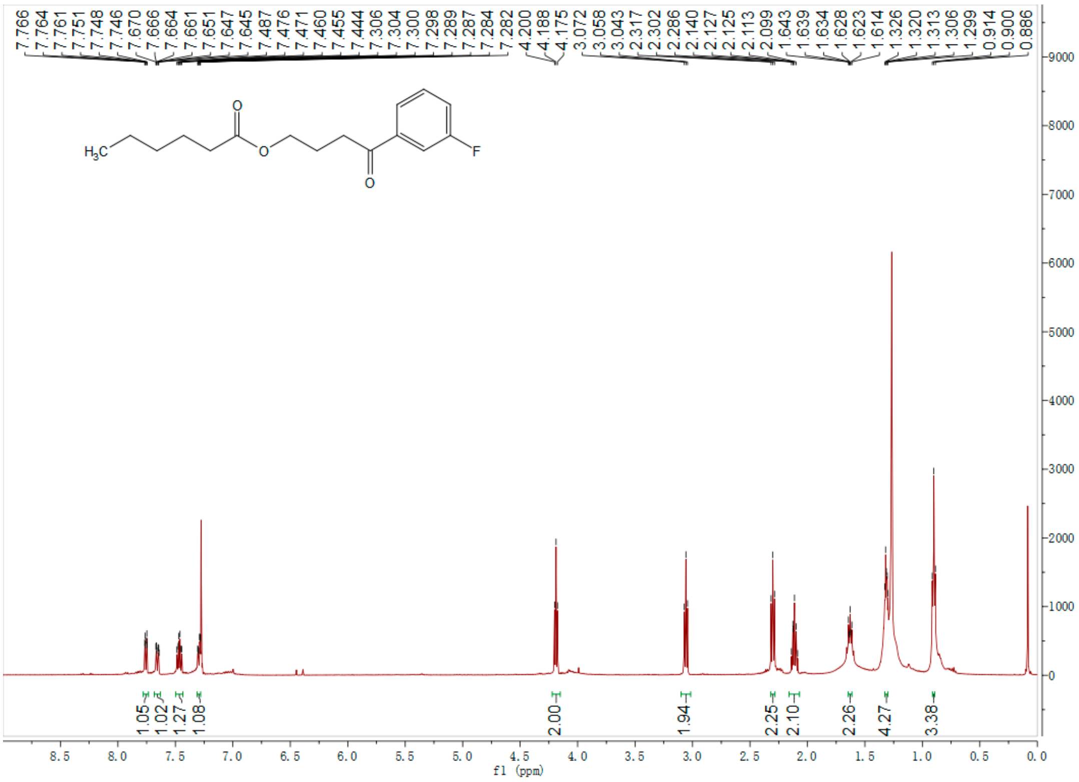

Figure S87 ^{1}H NMR spectra of **4r**

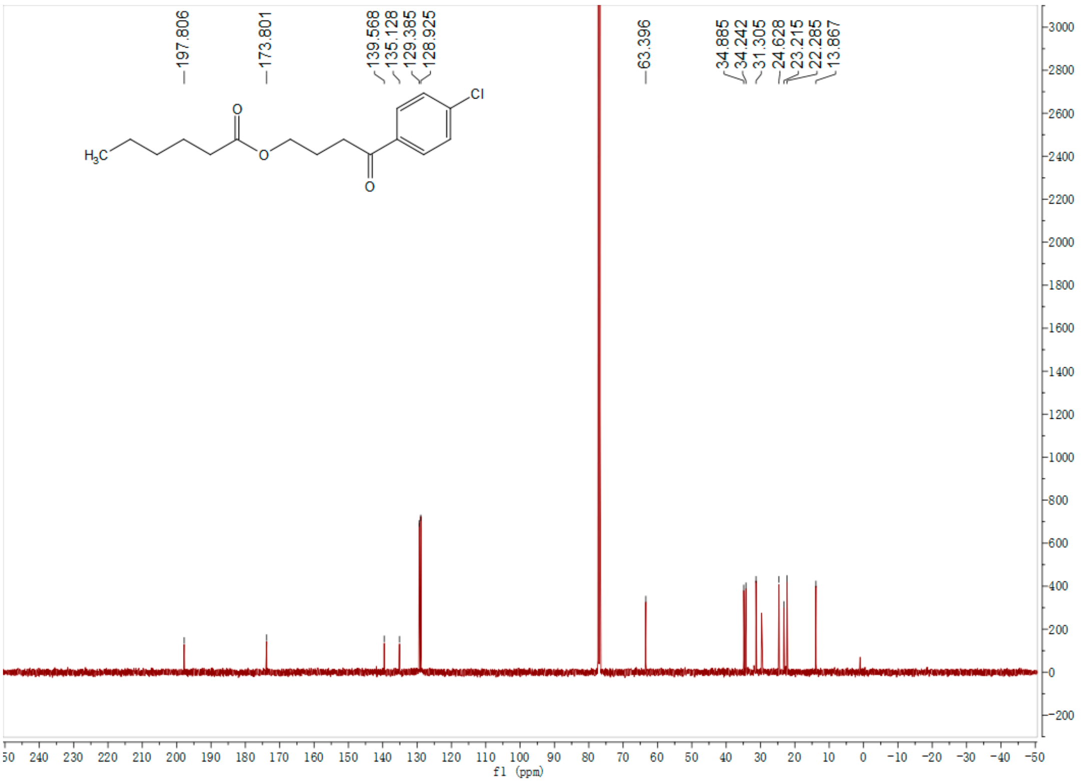

Figure S88 ^{13}C NMR spectra of **4s**

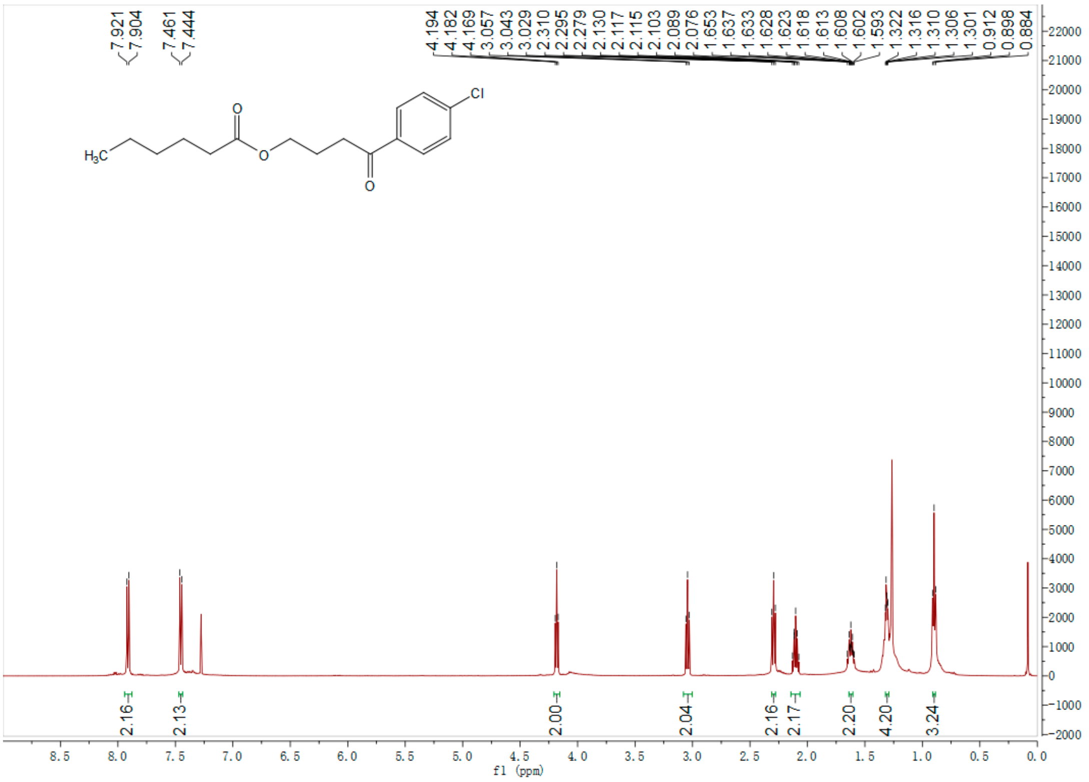

Figure S89 ^{1}H NMR spectra of **4s**

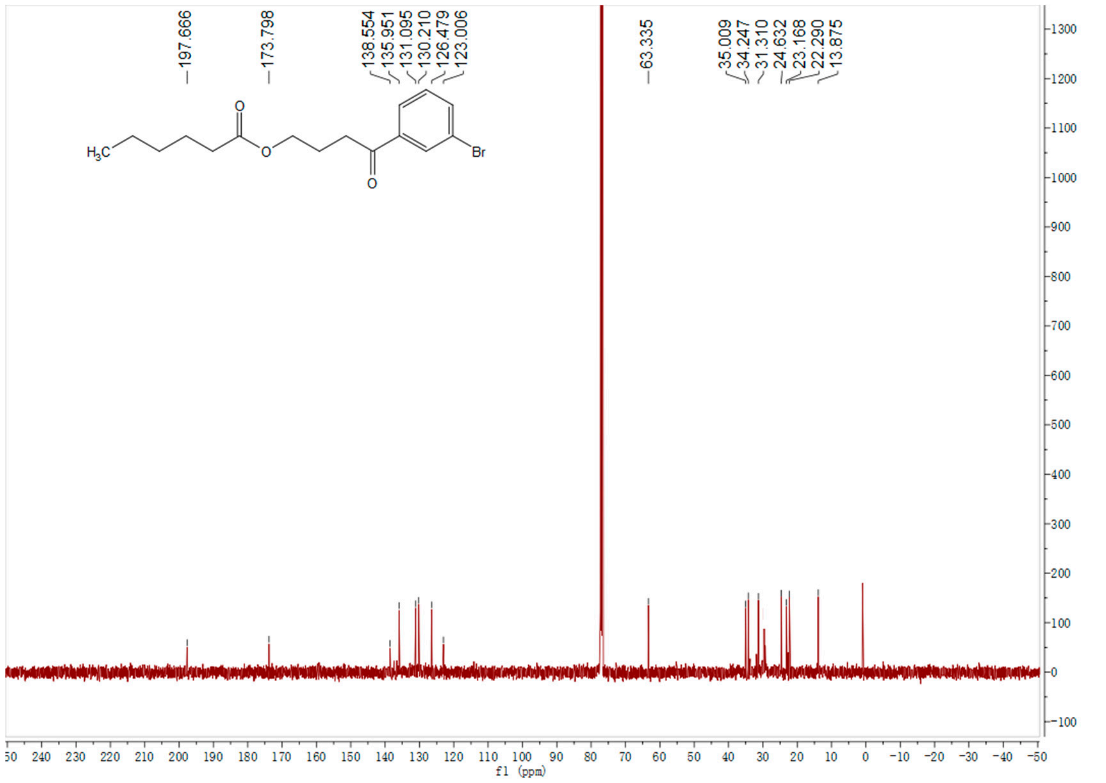

Figure S90 ^{13}C NMR spectra of **4t**

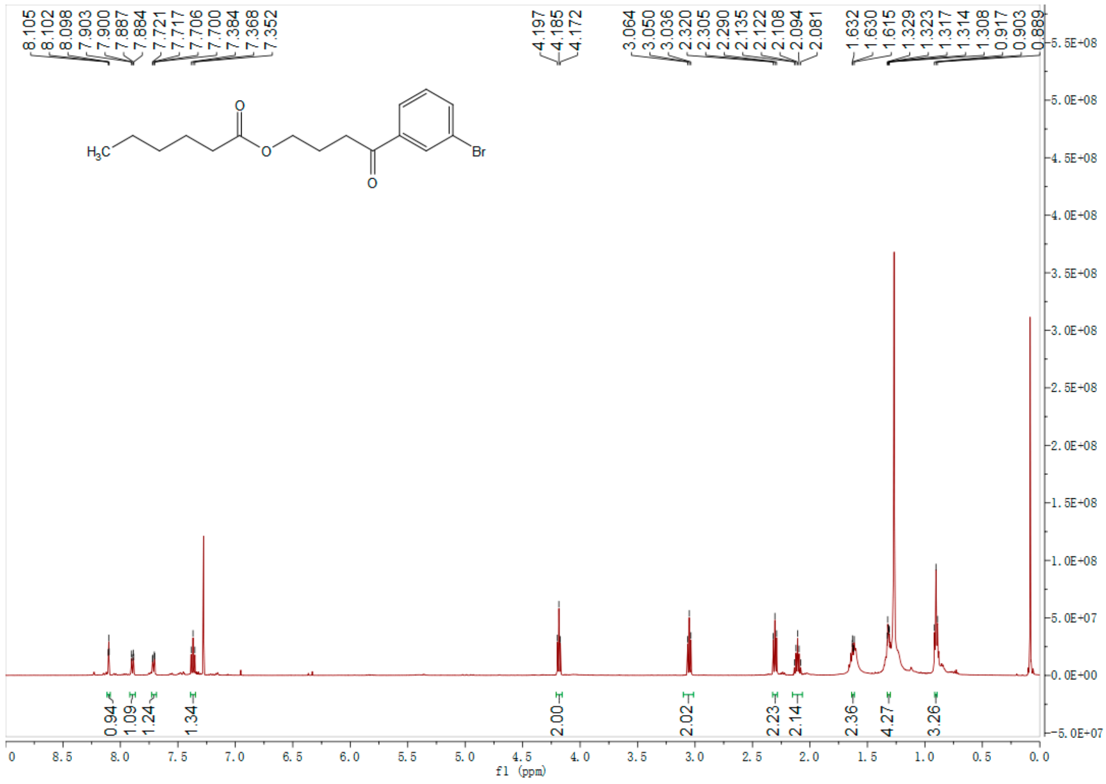

Figure S91 ^{1}H NMR spectra of **4t**

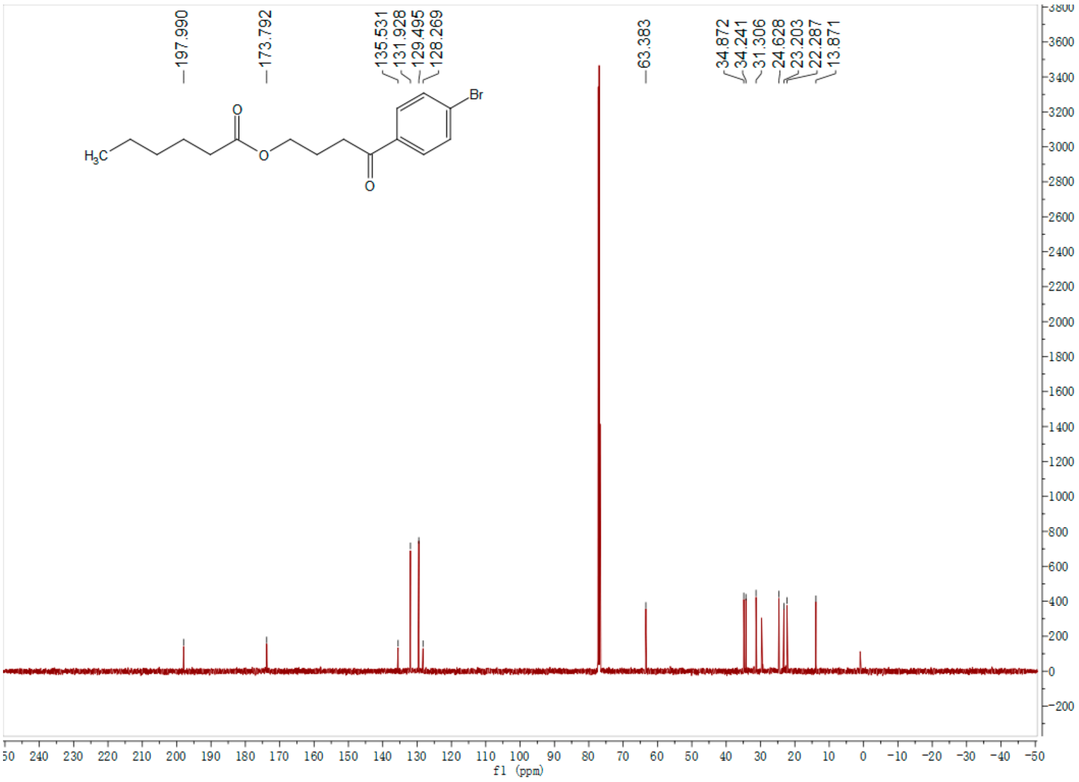

Figure S92 ^{13}C NMR spectra of **4u**

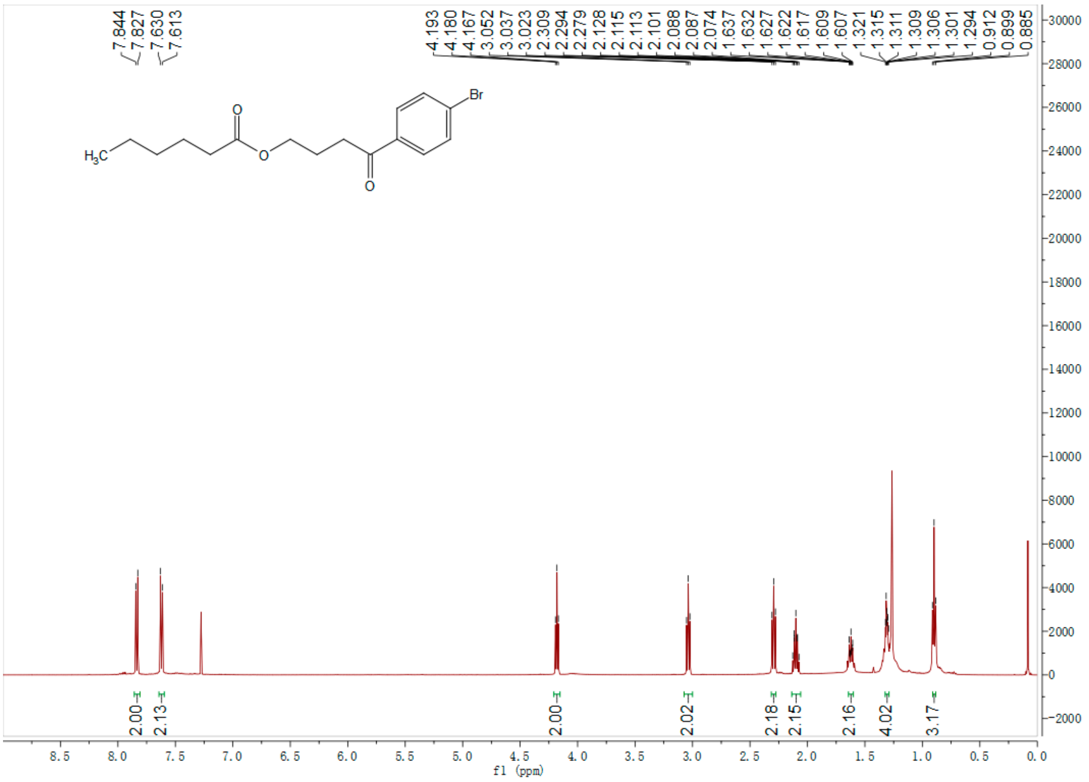

Figure S93 ^{1}H NMR spectra of **4u**

3. ^{1}H and ^{13}C NMR spectra of compounds **5**

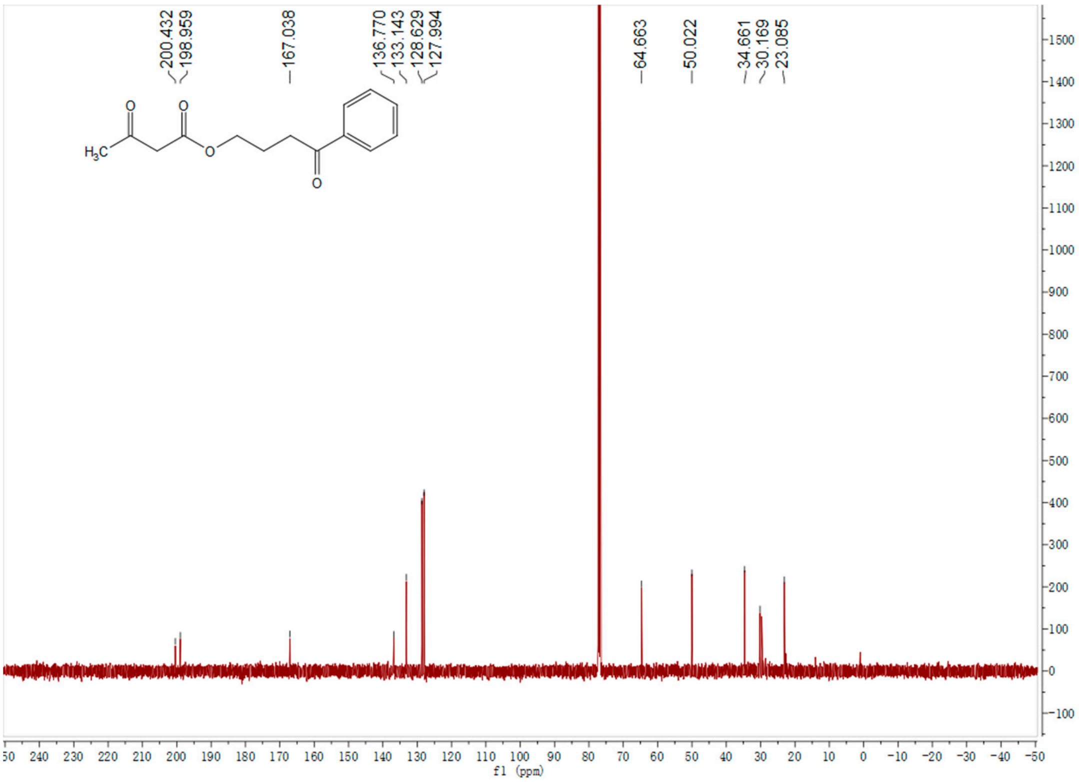

Figure S94 ^{13}C NMR spectra of **5a**

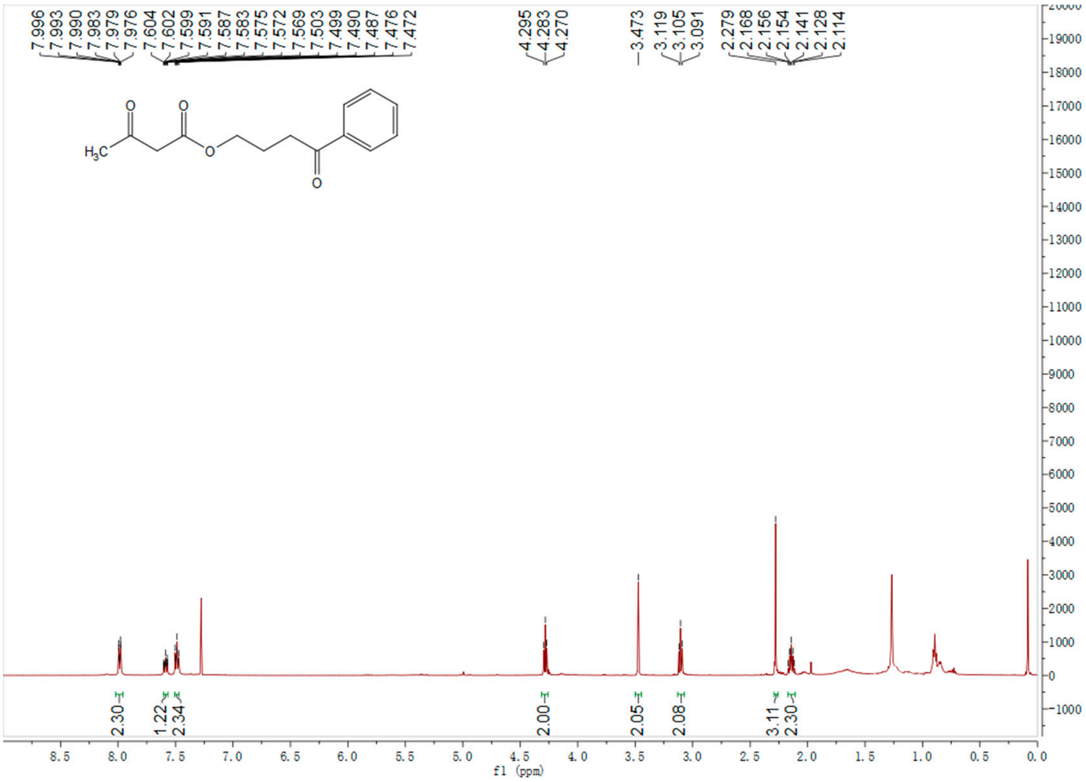

Figure S95 ^{1}H NMR spectra of **5a**

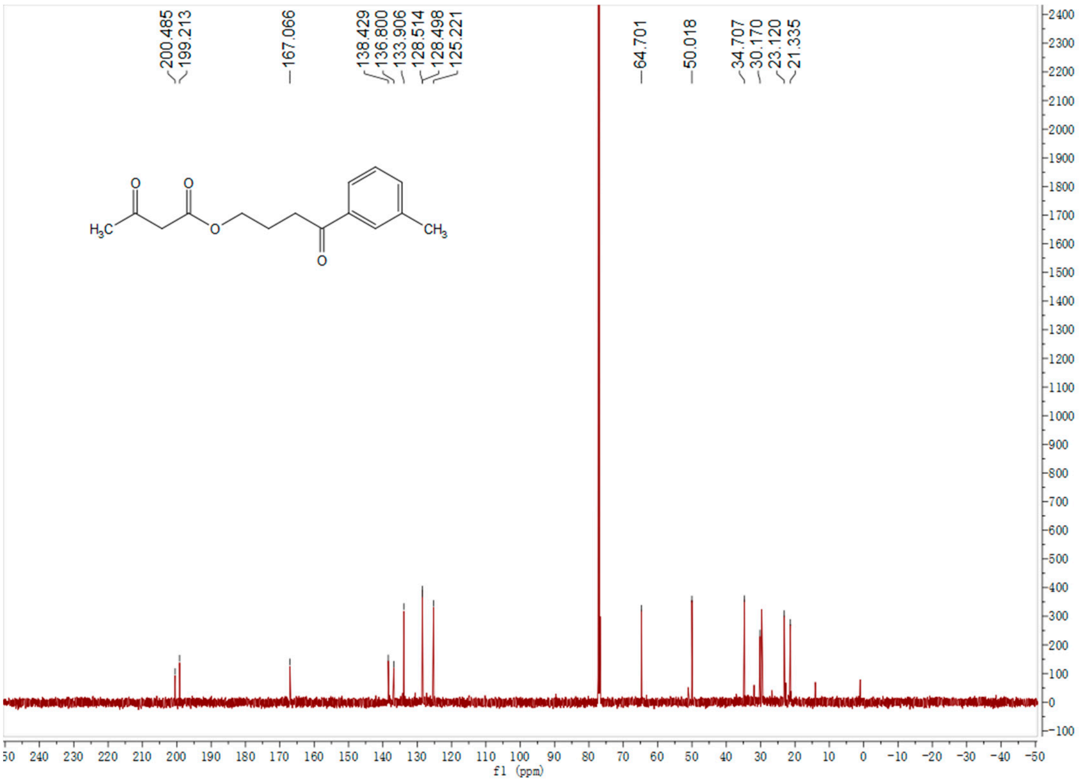

Figure S96 ^{13}C NMR spectra of **5b**

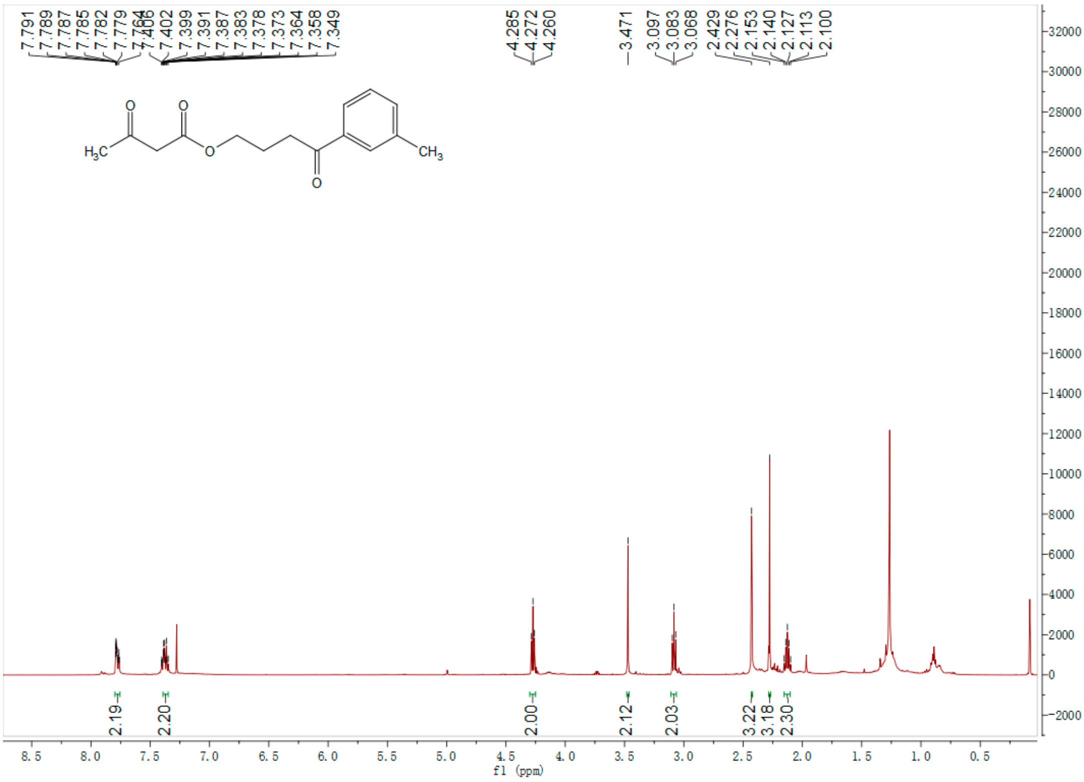

Figure S97 ^{1}H NMR spectra of **5b**

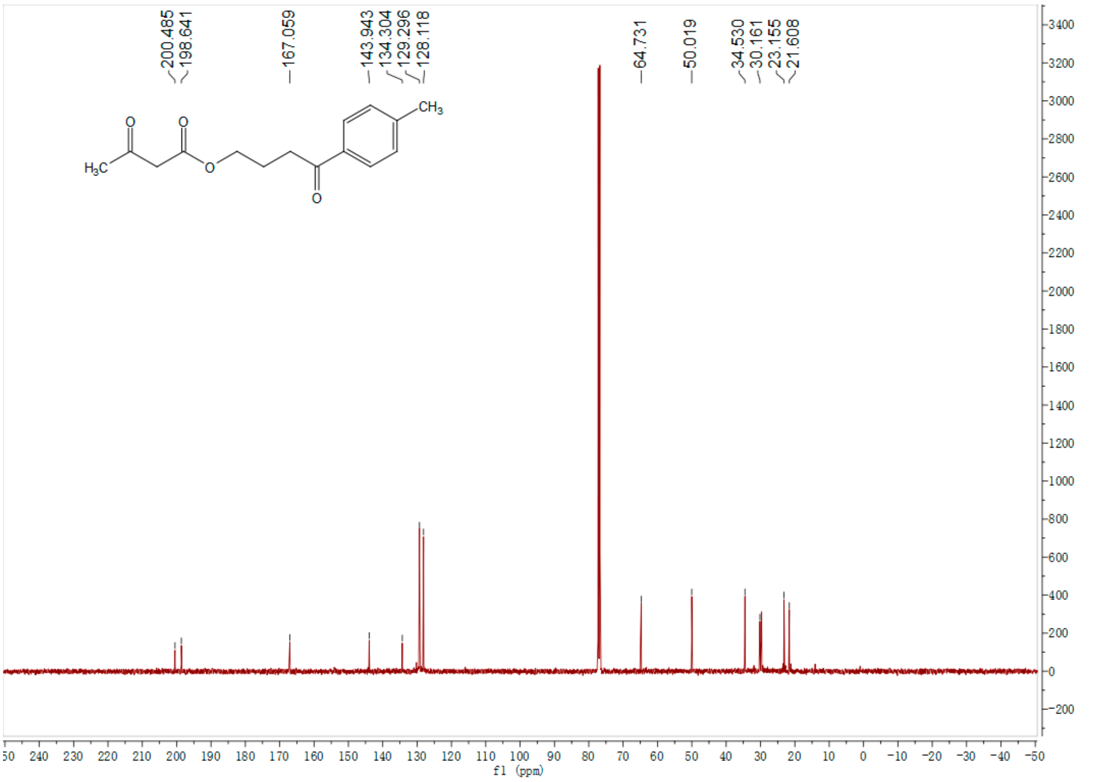

Figure S98 ^{13}C NMR spectra of **5c**

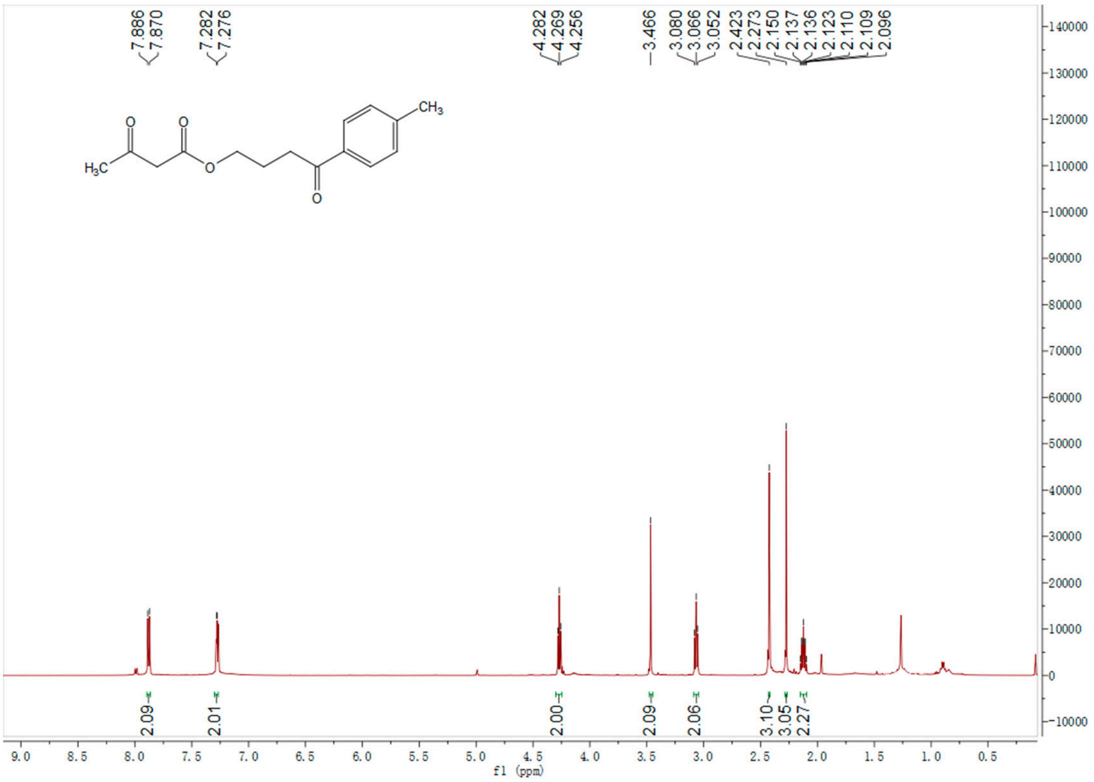

Figure S99 ^{1}H NMR spectra of **5c**

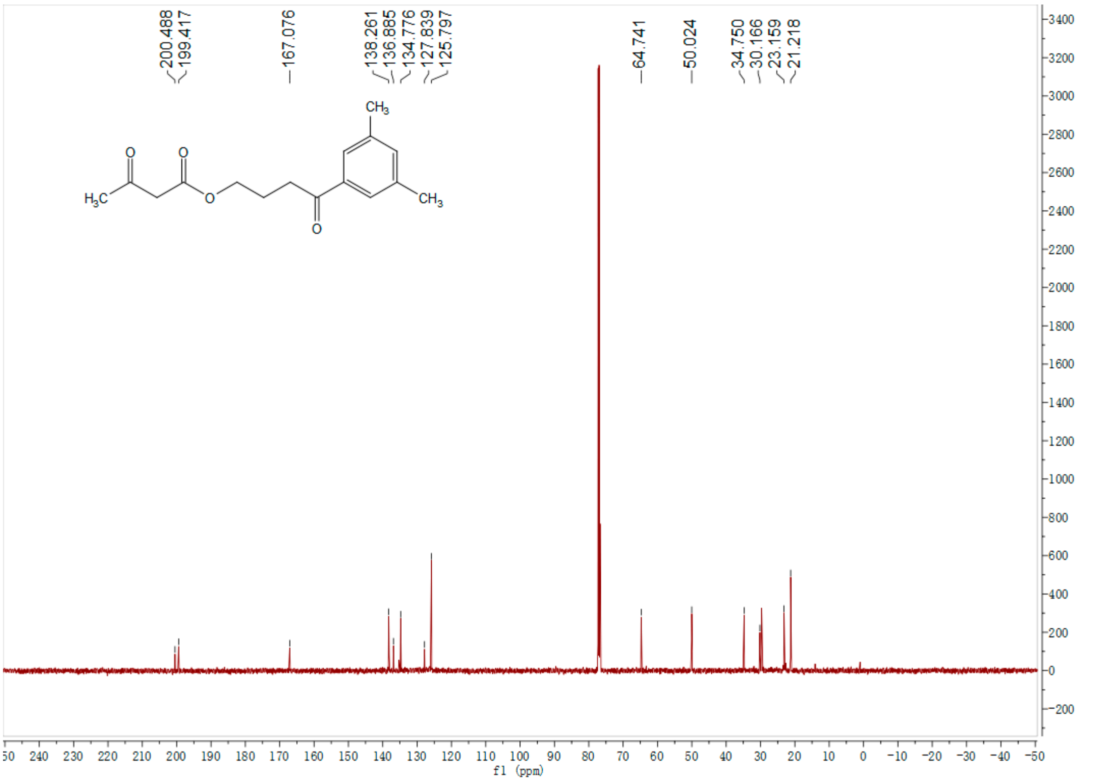

Figure S100 ^{13}C NMR spectra of **5d**

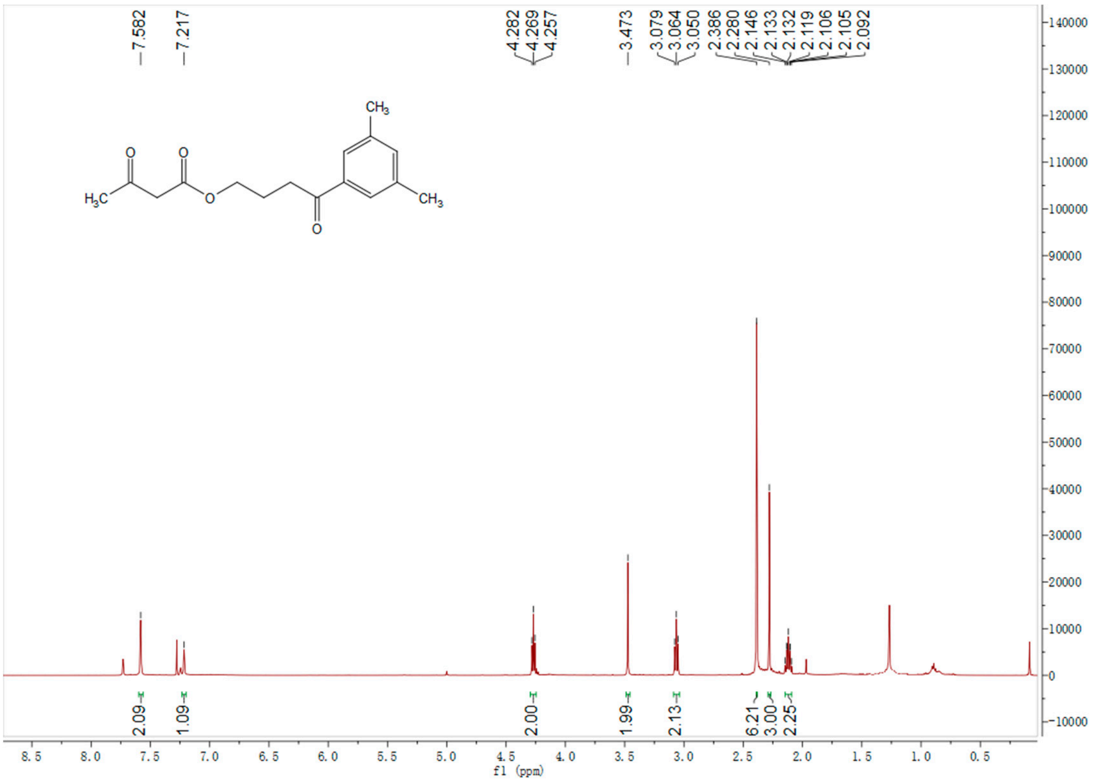

Figure S101 ^{1}H NMR spectra of **5d**

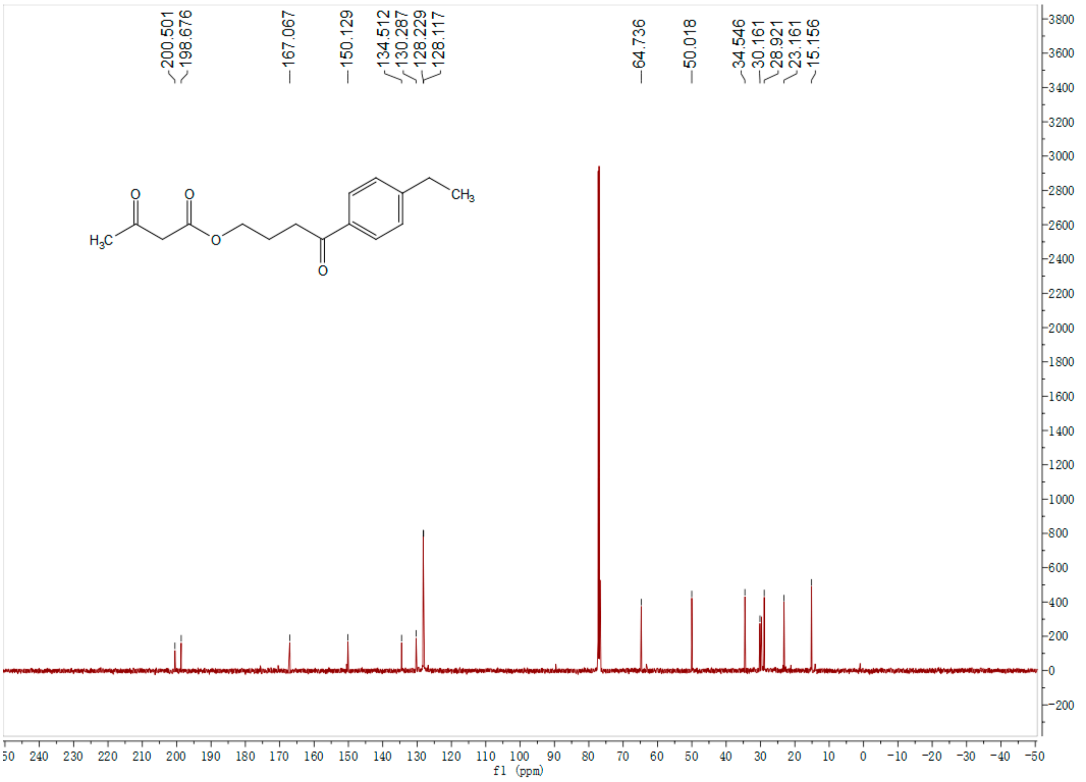

Figure S102 ^{13}C NMR spectra of **5e**

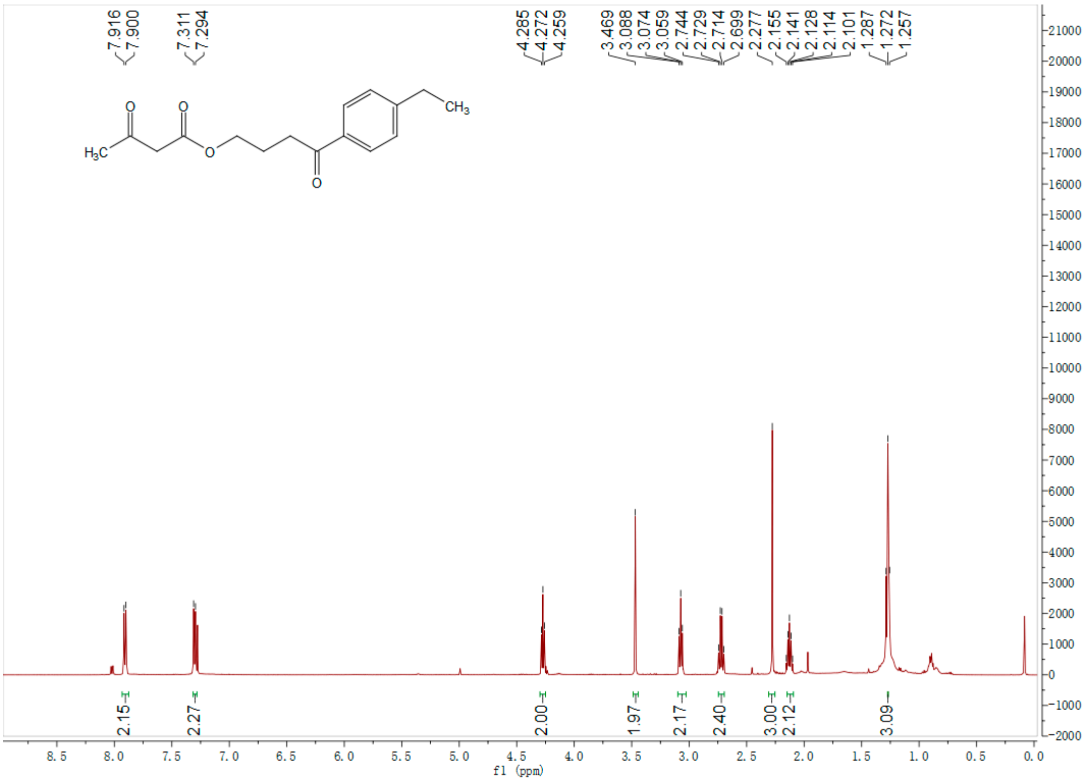

Figure S103 ^{1}H NMR spectra of **5e**

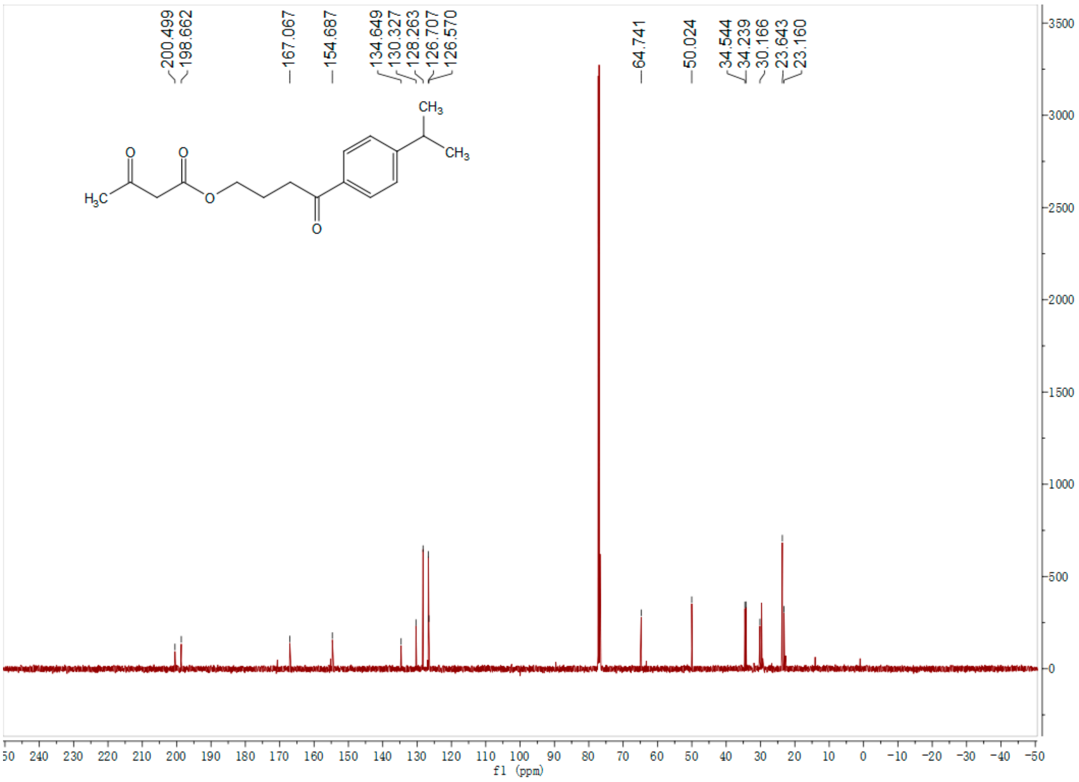

Figure S104 ^{13}C NMR spectra of **5f**

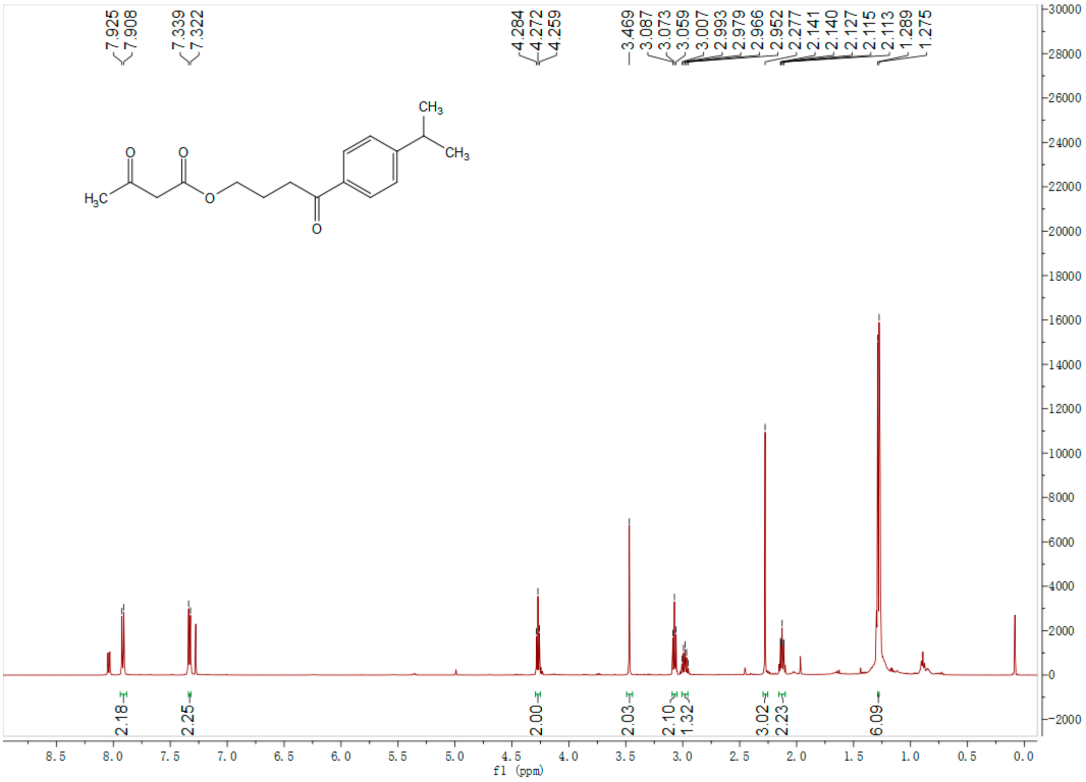

Figure S105 ^{1}H NMR spectra of **5f**

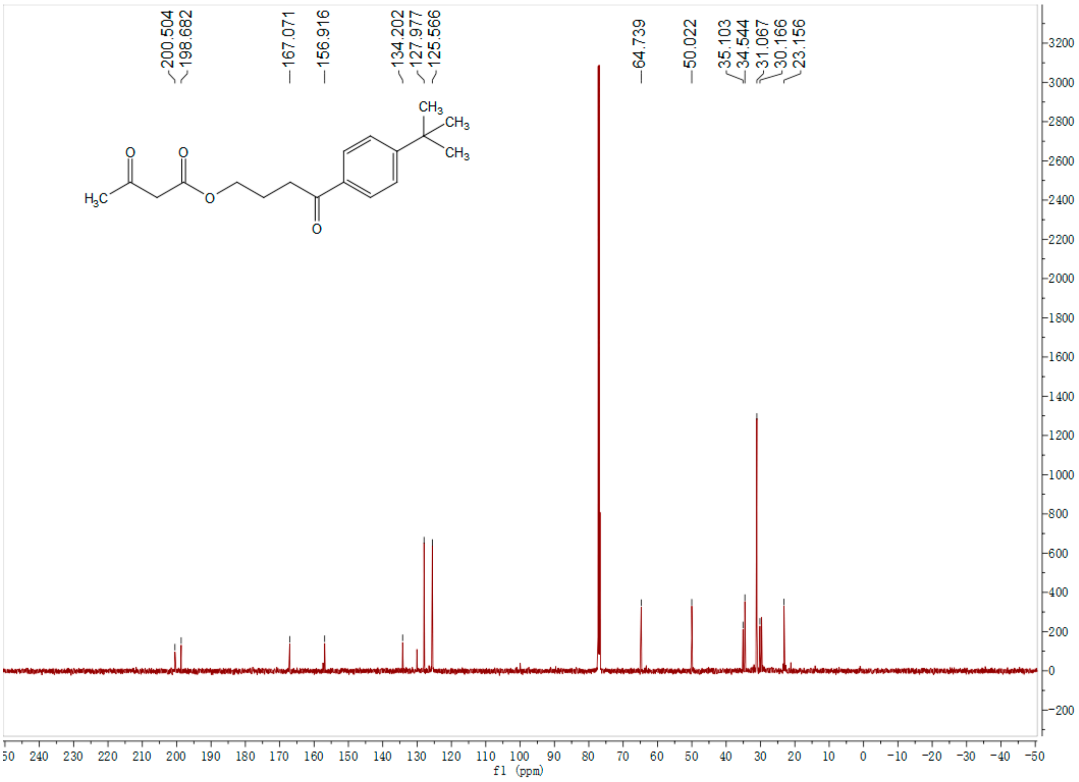

Figure S106 ^{13}C NMR spectra of **5g**

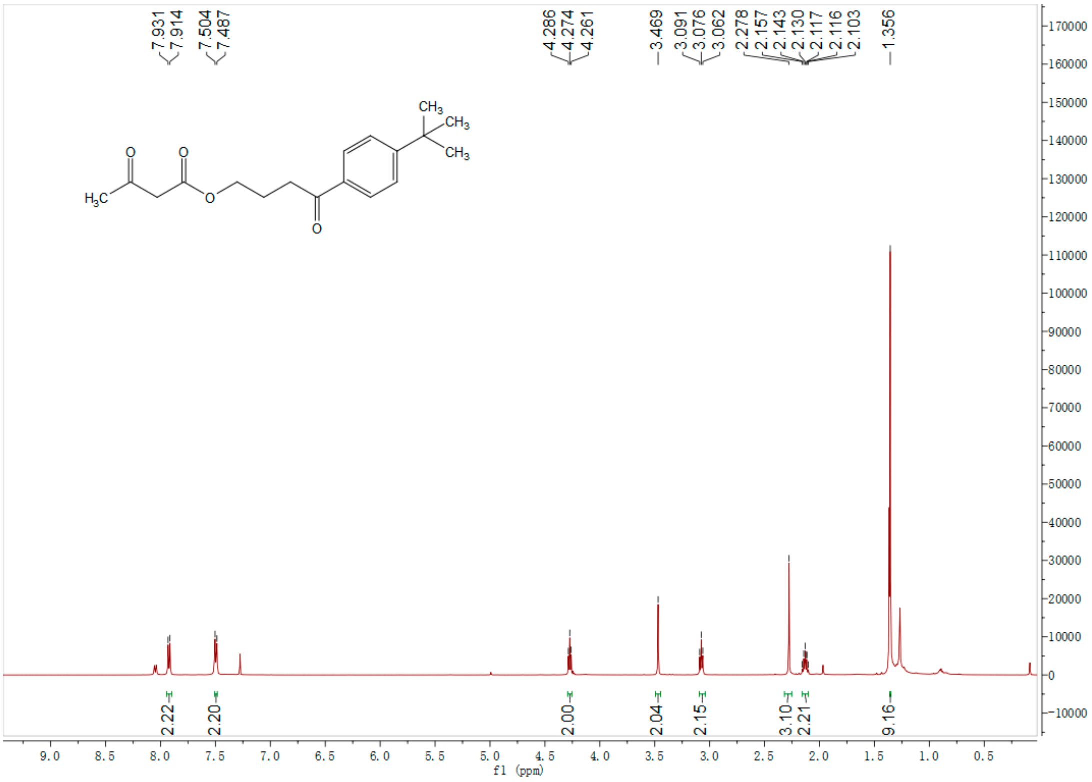

Figure S107 ^{1}H NMR spectra of **5g**

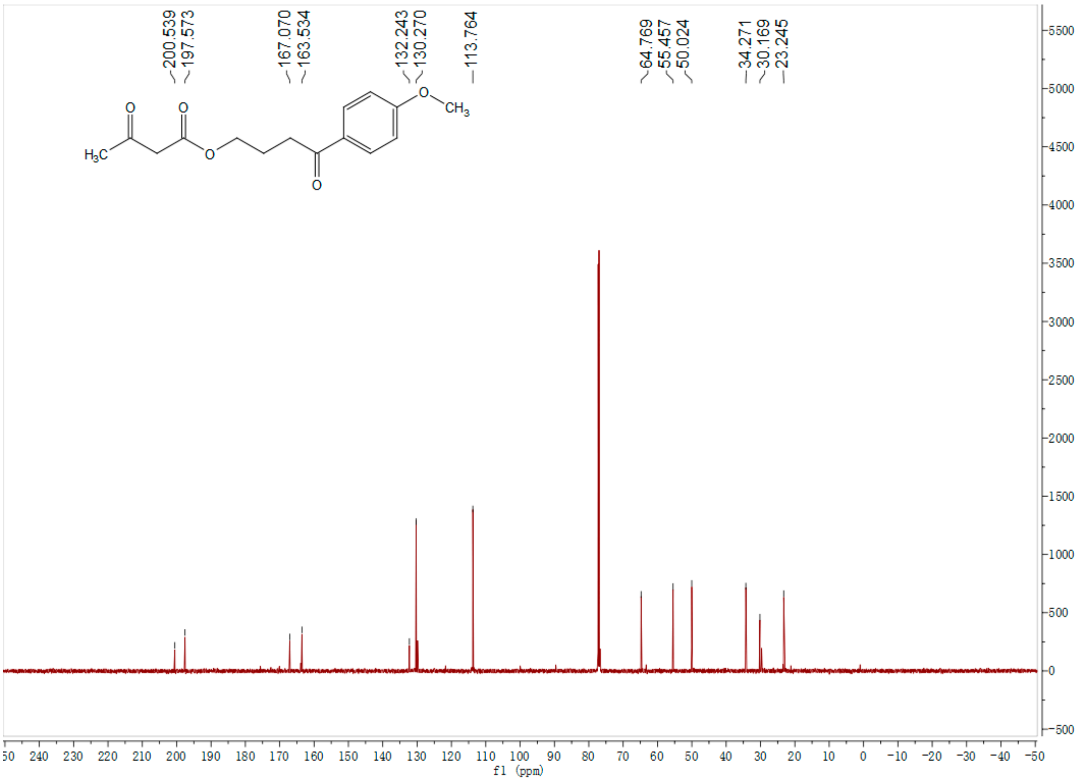

Figure S108 ^{13}C NMR spectra of **5h**

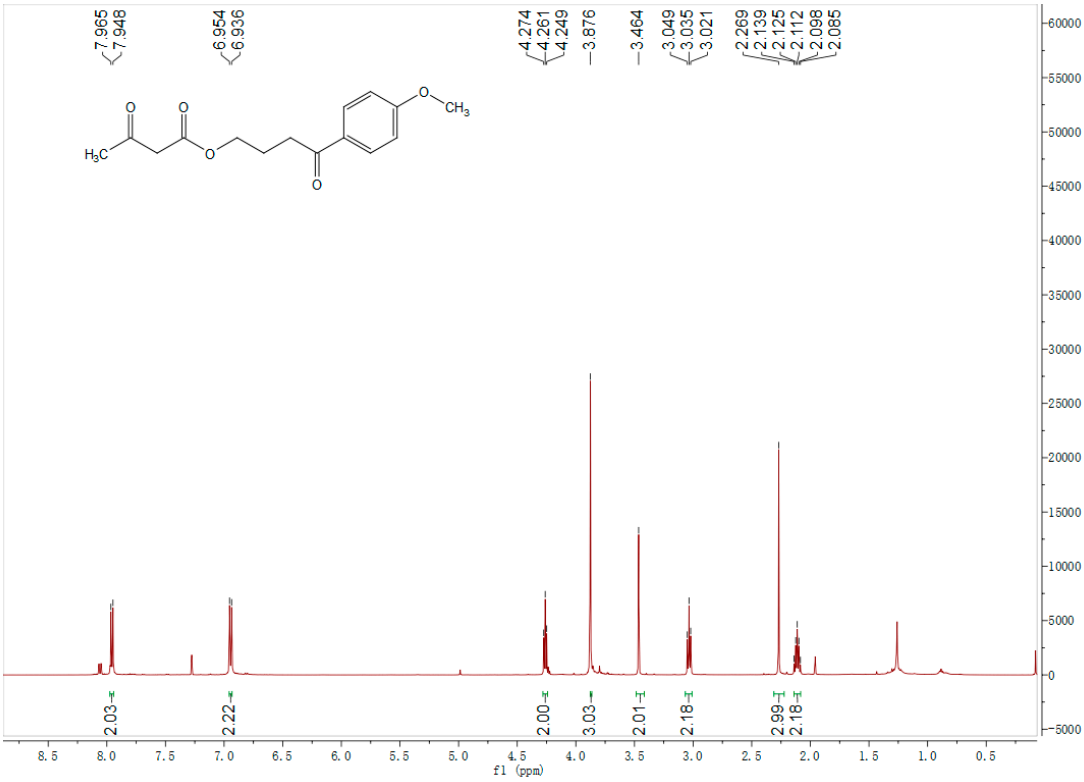

Figure S109 ^{1}H NMR spectra of **5h**

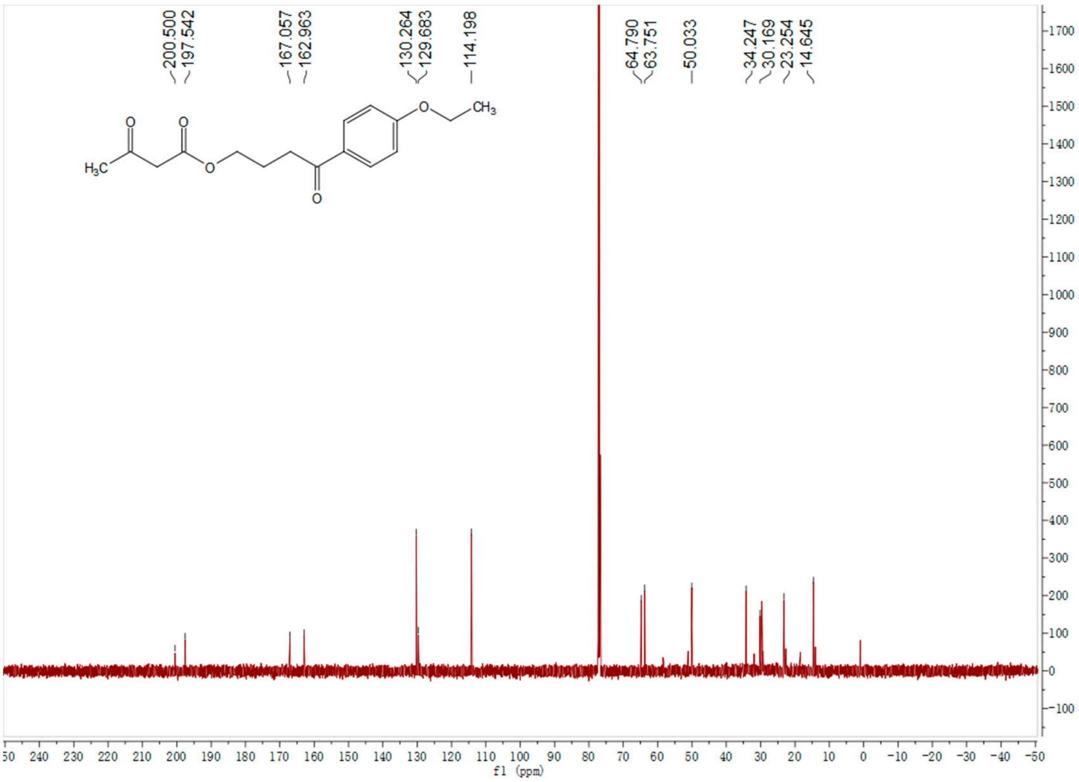

Figure S110 ^{13}C NMR spectra of **5i**

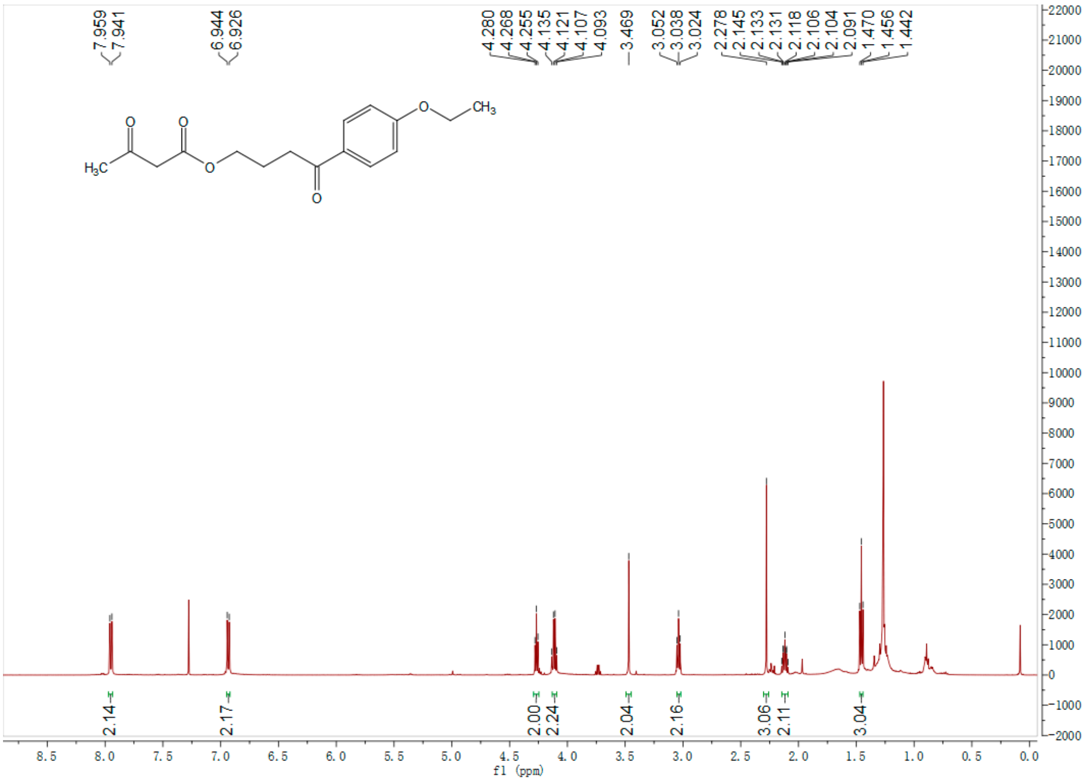

Figure S111 ^{1}H NMR spectra of **5i**

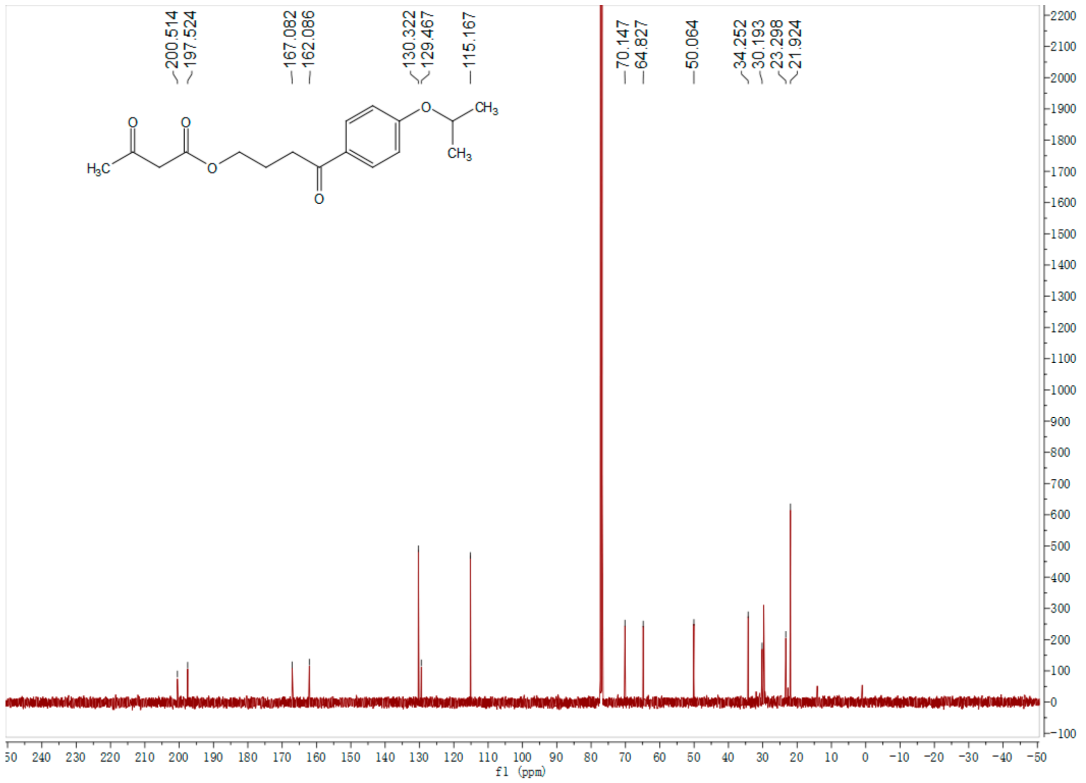

Figure S112 ^{13}C NMR spectra of **5j**

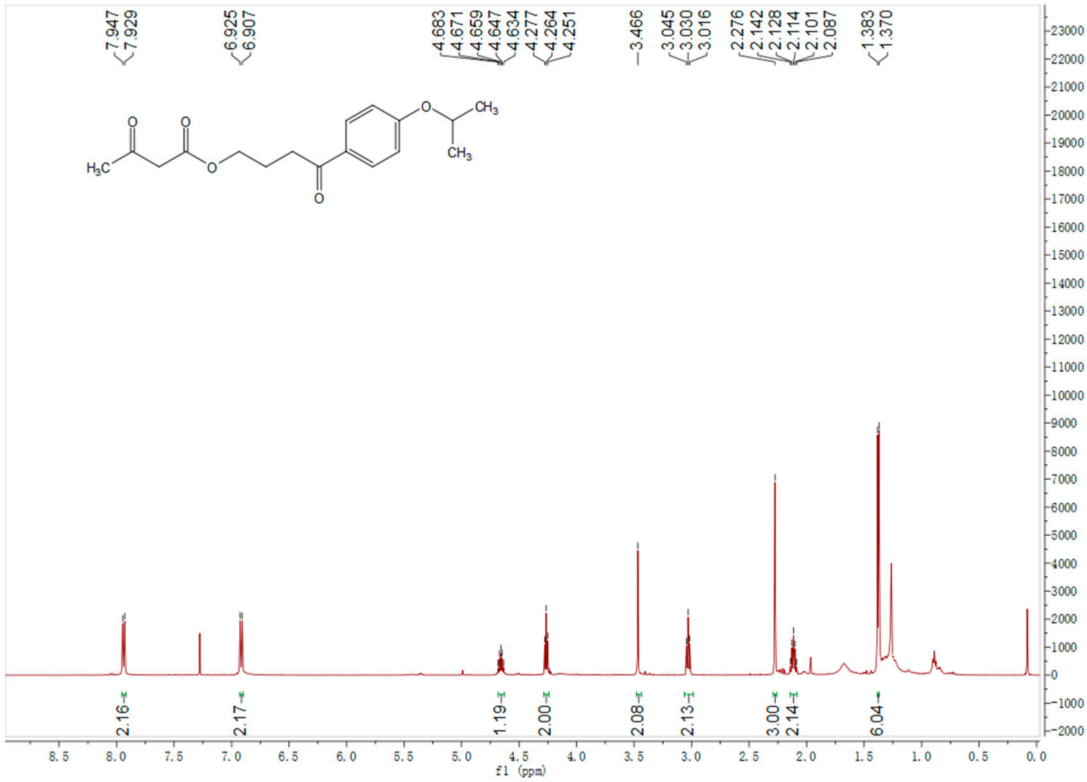

Figure S113 ^{1}H NMR spectra of **5j**

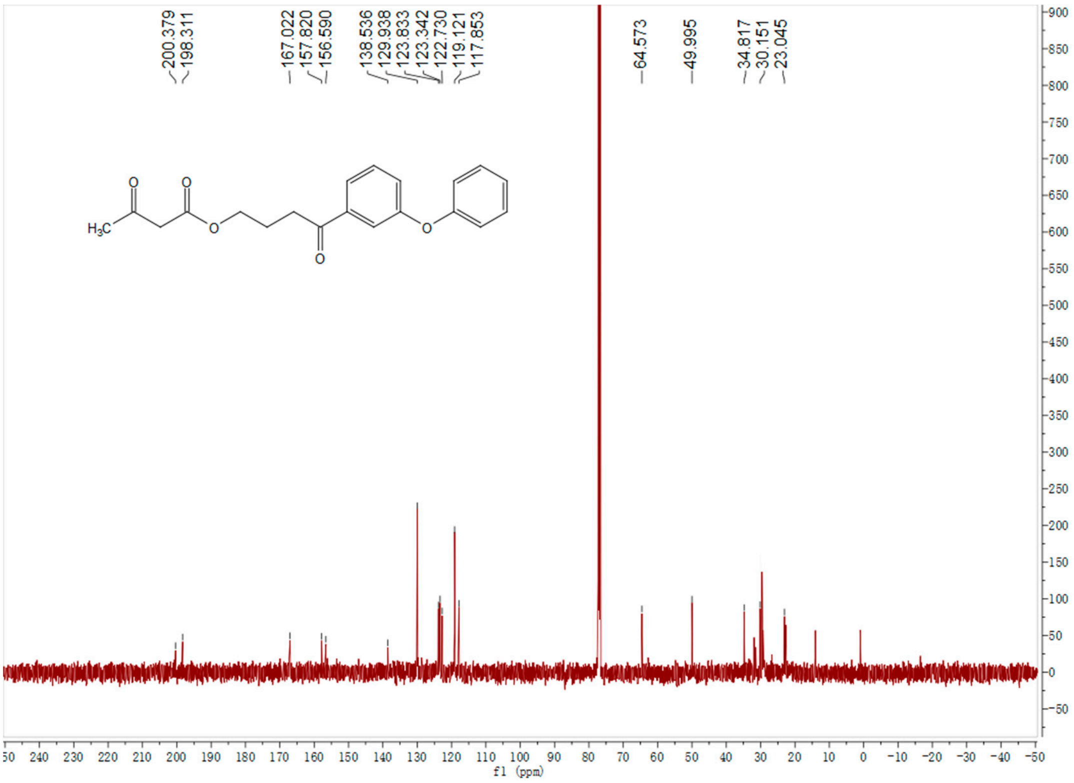

Figure S114 ^{13}C NMR spectra of **5k**

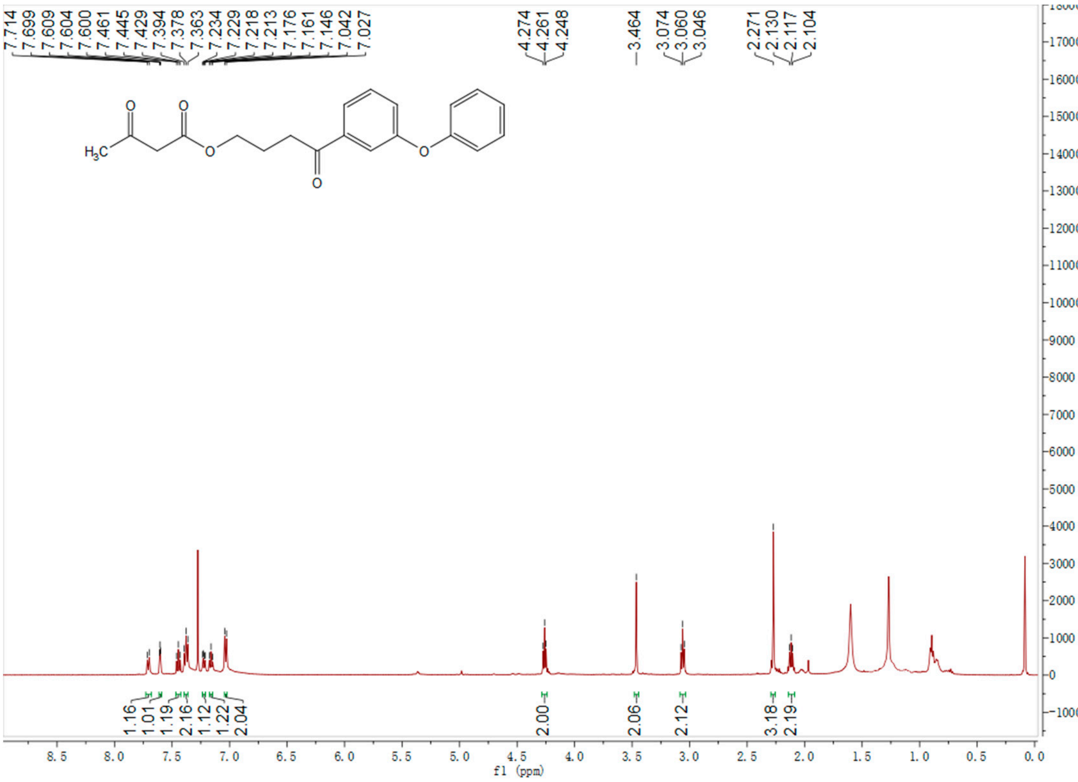

Figure S115 ^{1}H NMR spectra of **5k**

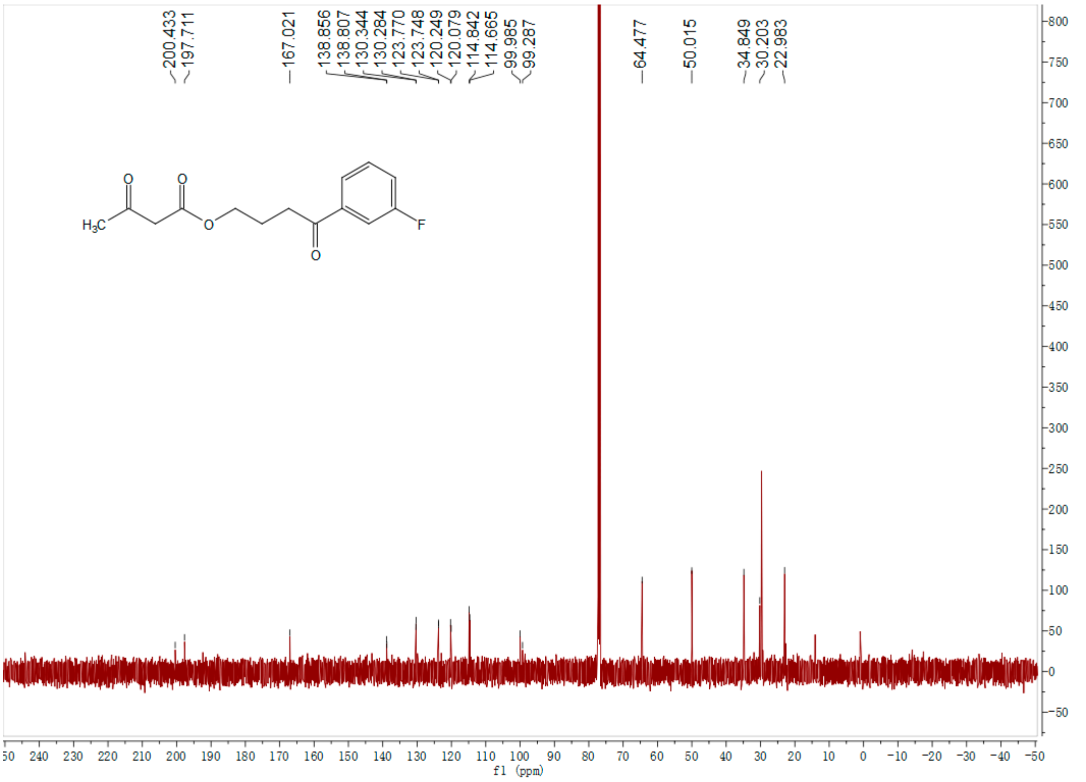

Figure S116 ^{13}C NMR spectra of **5l**

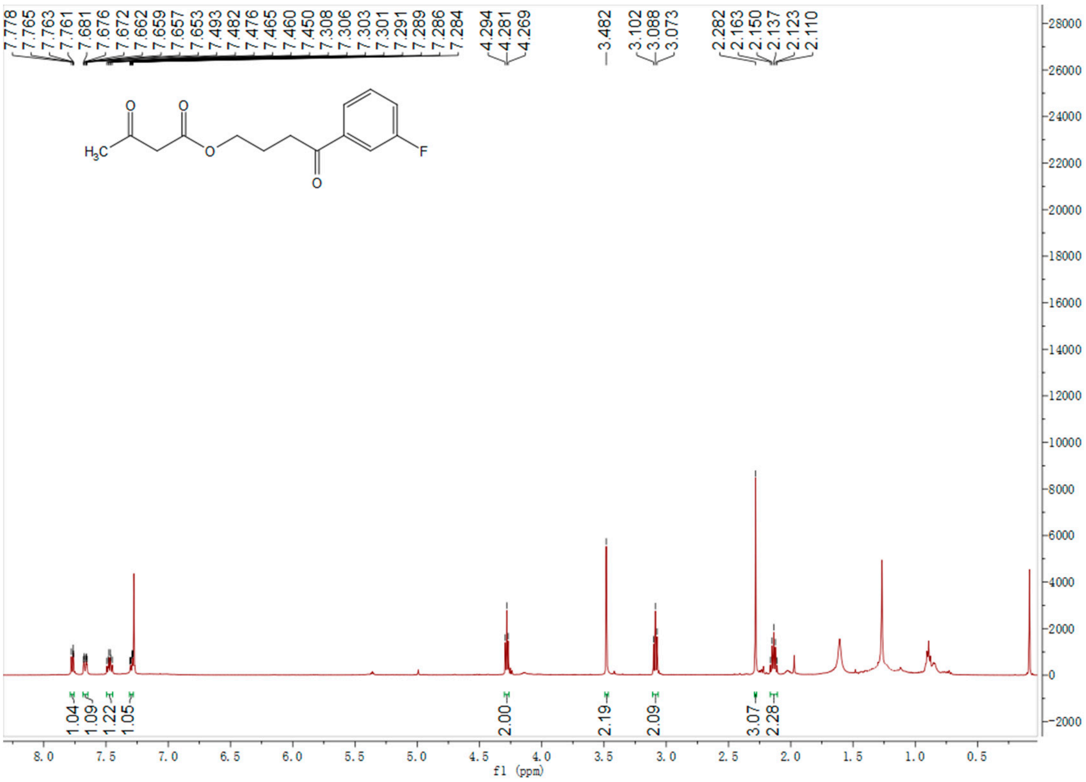

Figure S117 ^{1}H NMR spectra of **5l**

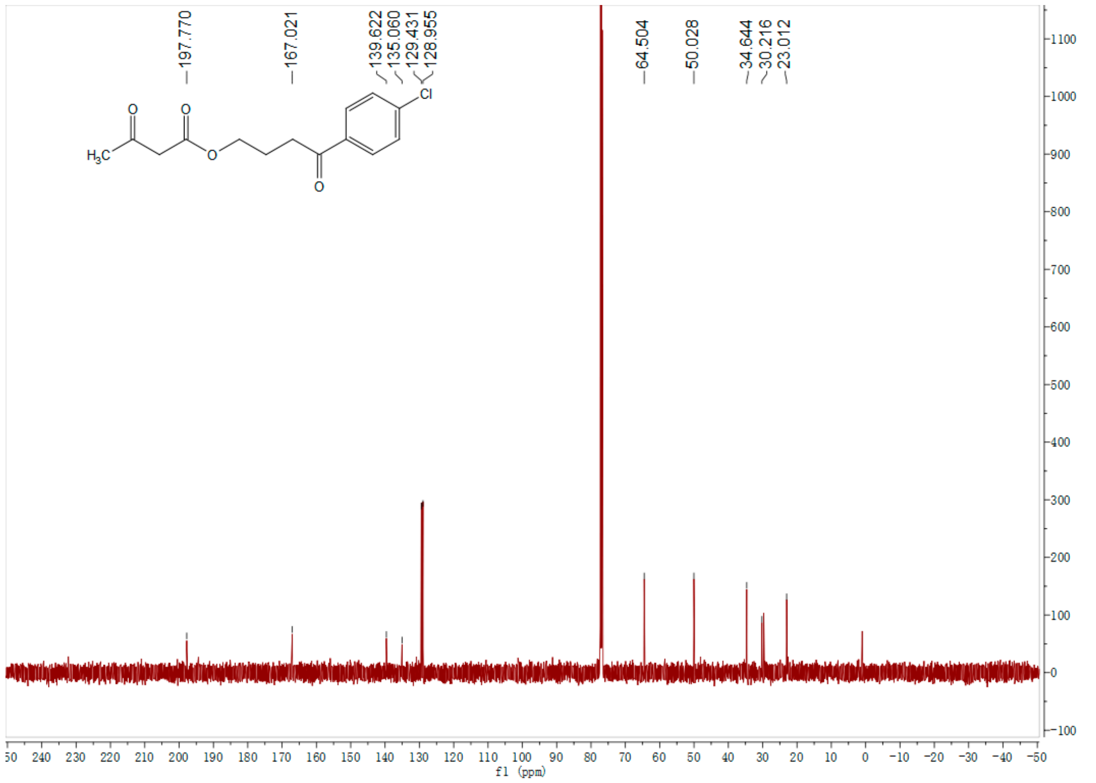

Figure S118 ^{13}C NMR spectra of **5m**

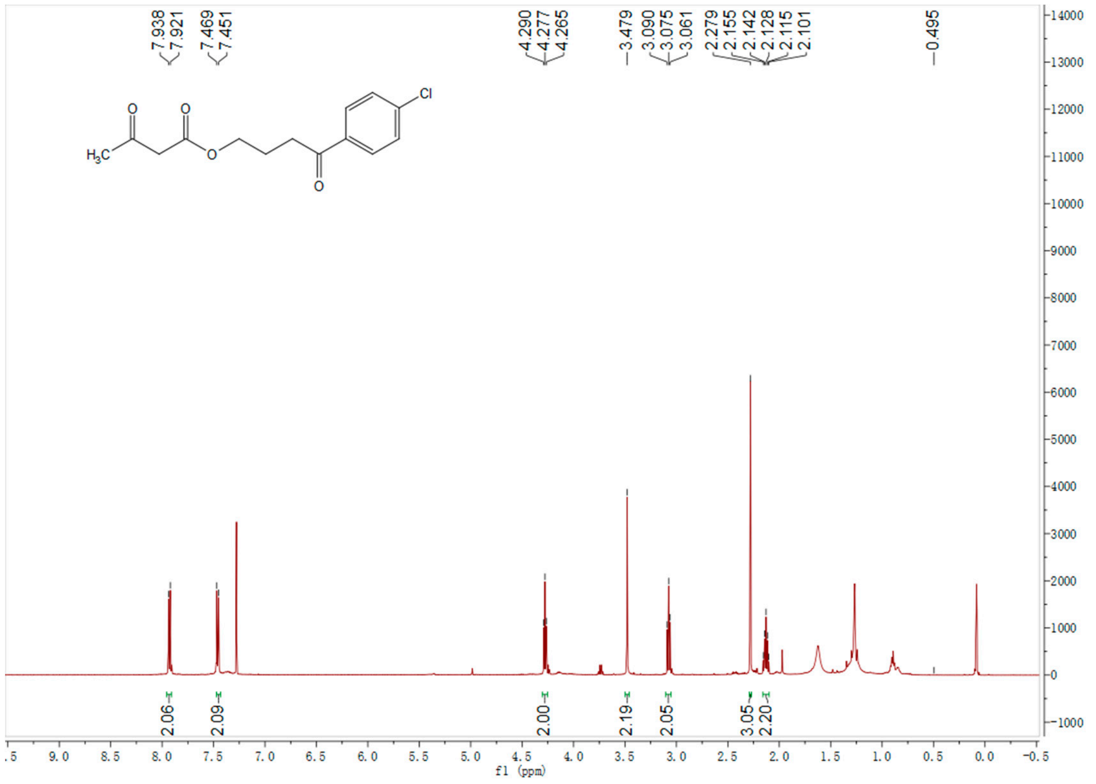

Figure 119 ^{1}H NMR spectra of **5m**

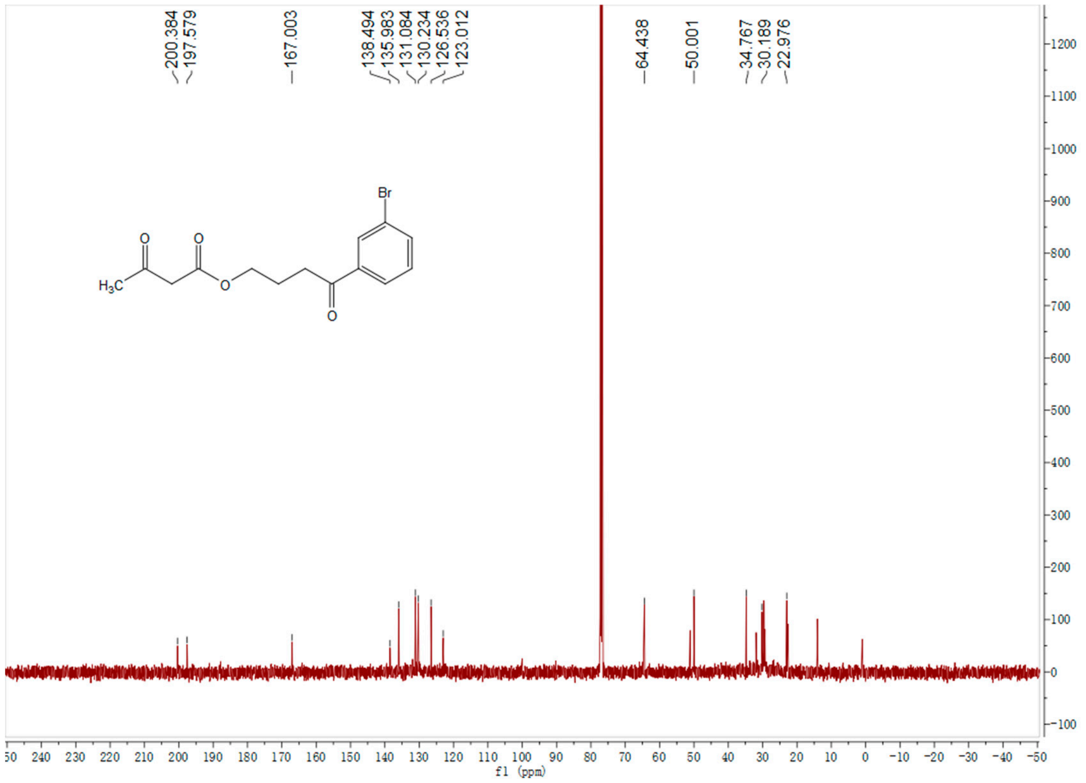

Figure S120 ^{13}C NMR spectra of **5n**

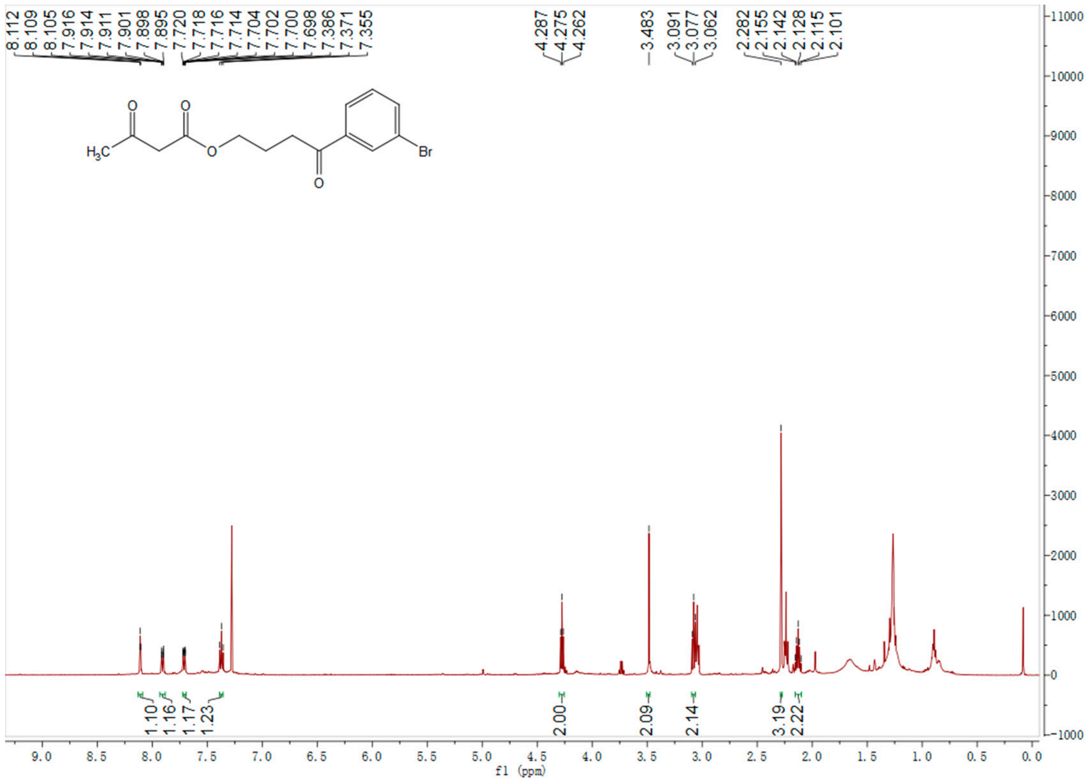

Figure S121 ^{1}H NMR spectra of **5n**

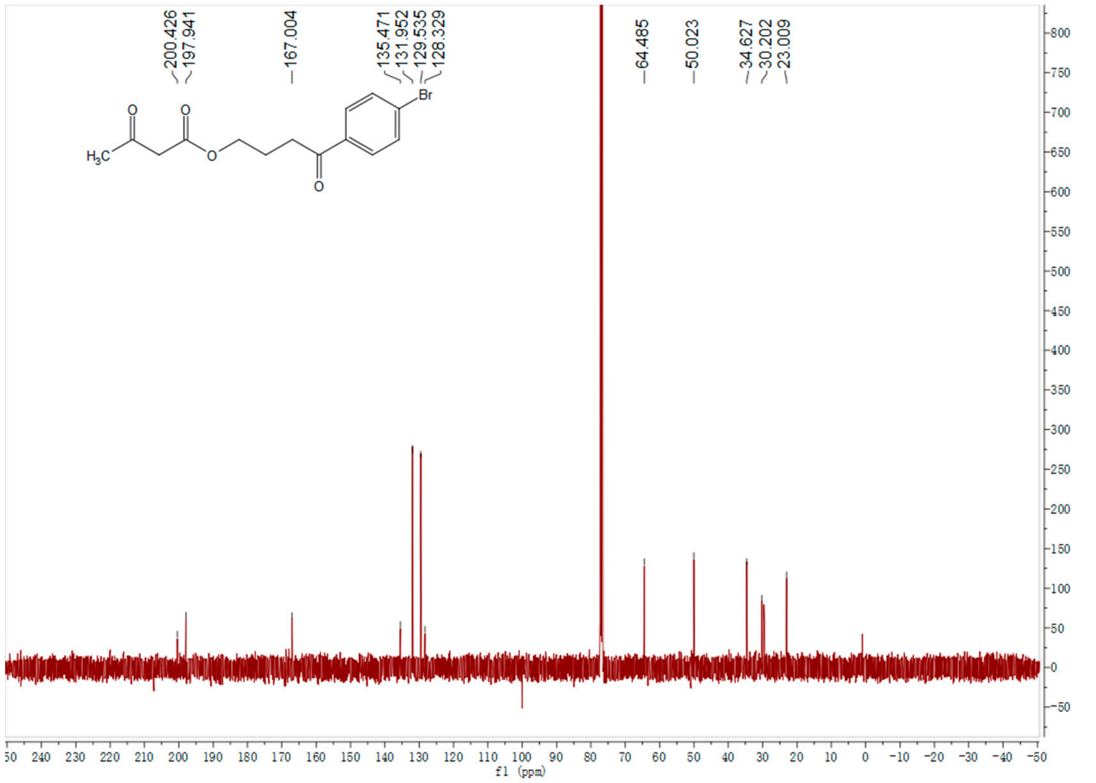

Figure S122 ^{13}C NMR spectra of **5o**

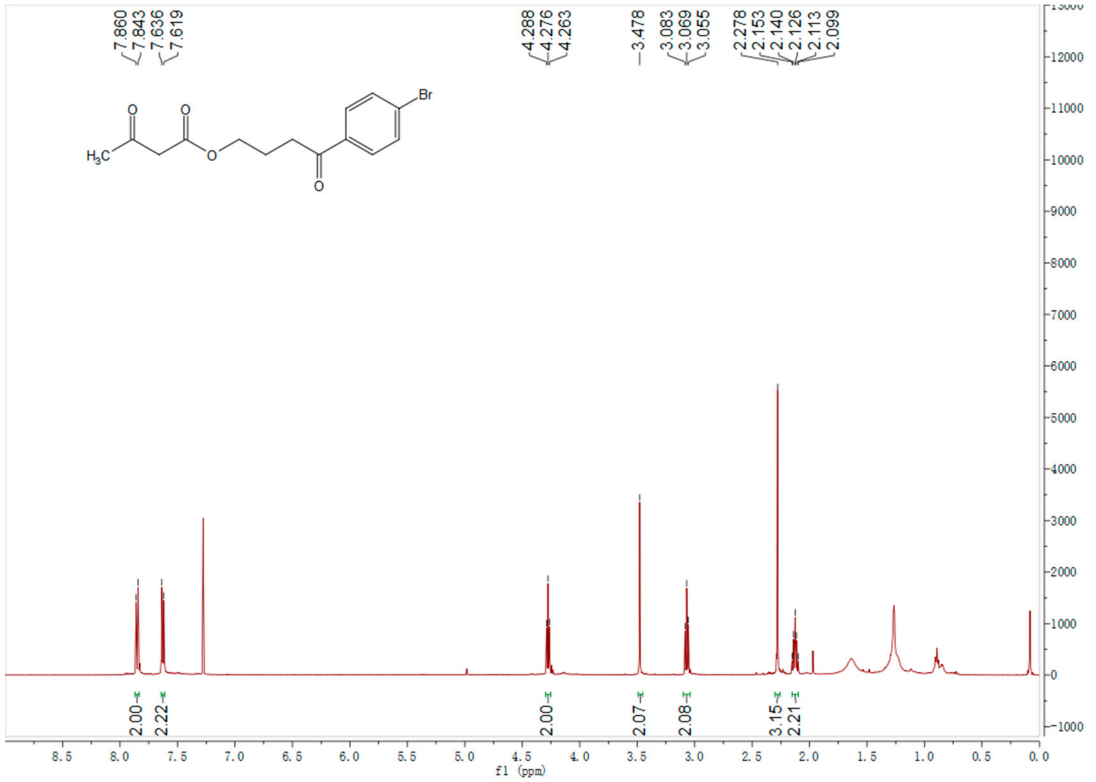

Figure S123 ^{1}H NMR spectra of **5o**

4. ESI-HRMS spectra of compounds **3**.

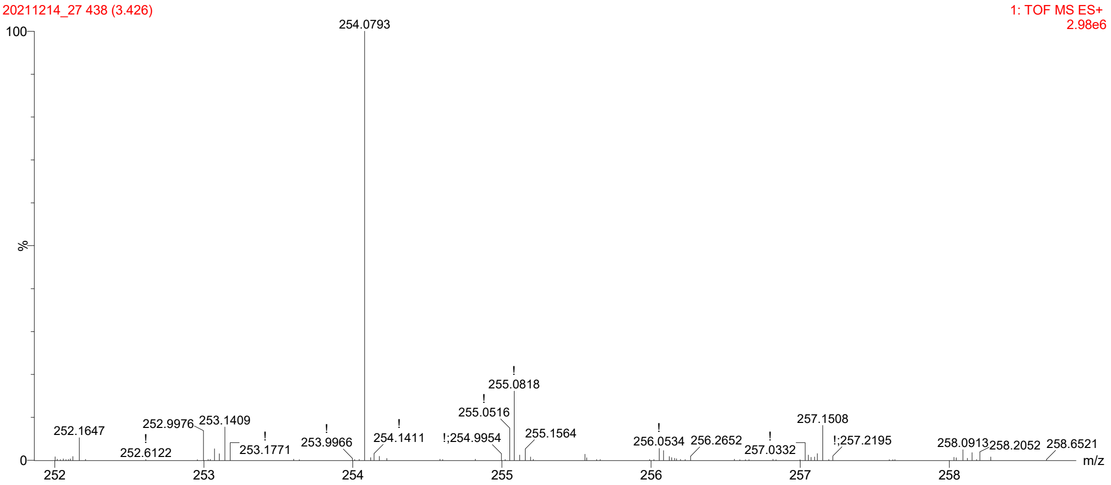

Figure S124 ESI-HRMS spectra of **3a**

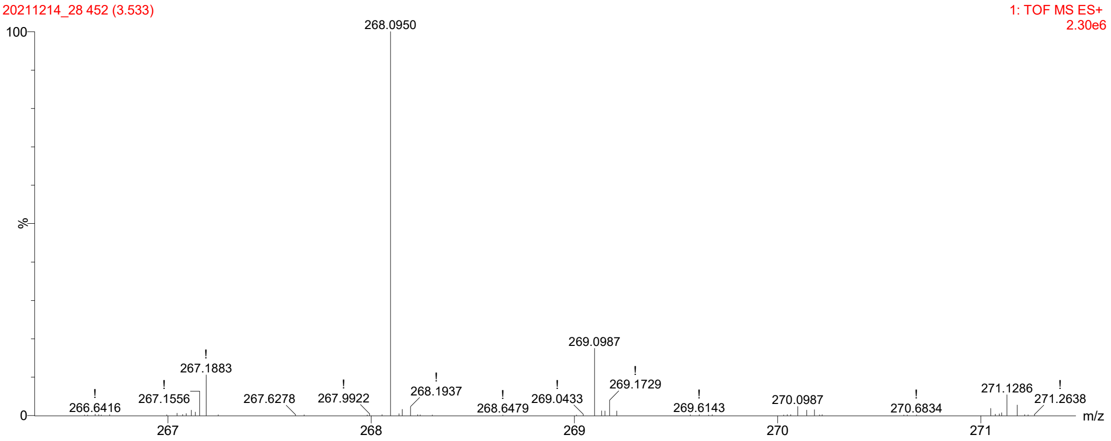

Figure S125 ESI-HRMS spectra of **3b**

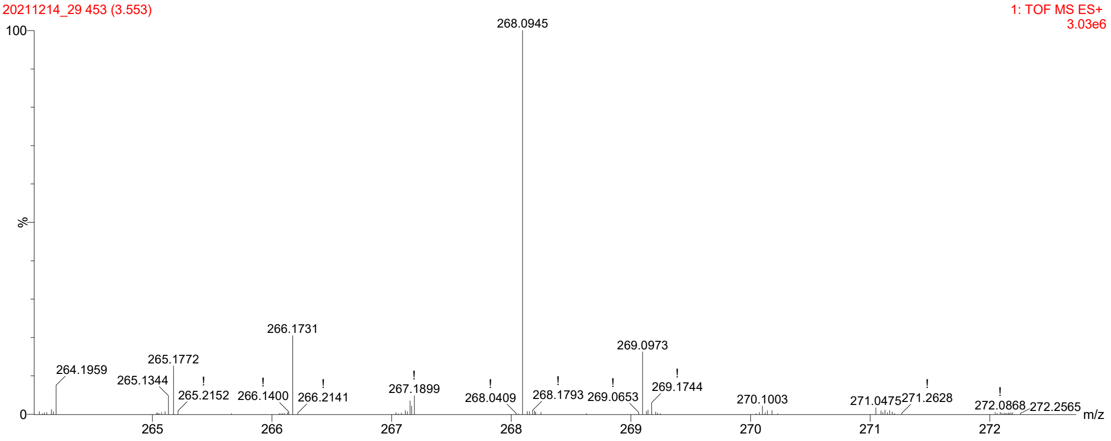

Figure S126 ESI-HRMS spectra of **3c**

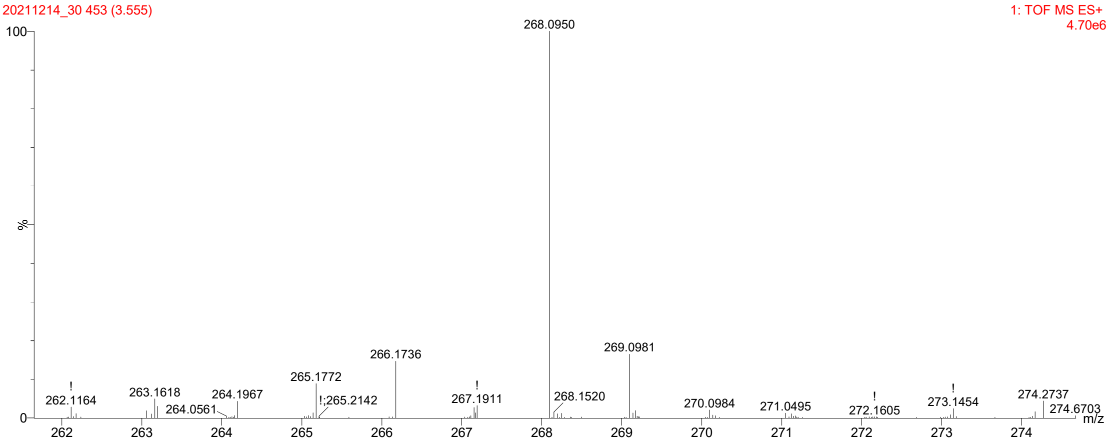

Figure S127 ESI-HRMS spectra of **3d**

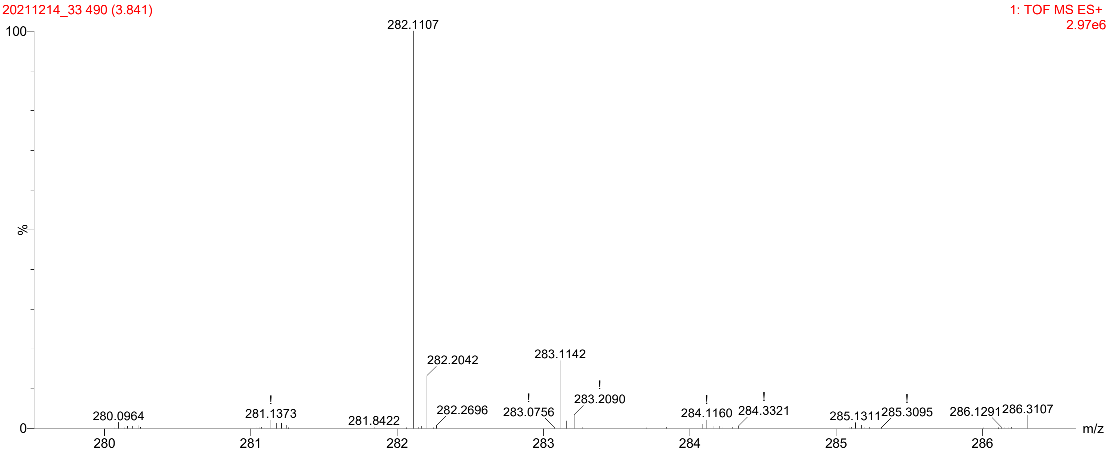

Figure S128 ESI-HRMS spectra of **3e**

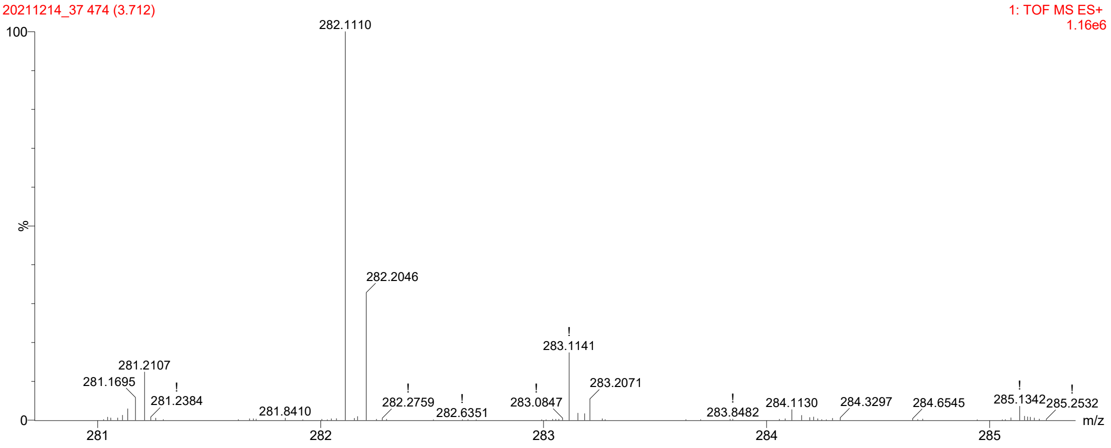

Figure S129 ESI-HRMS spectra of **3f**

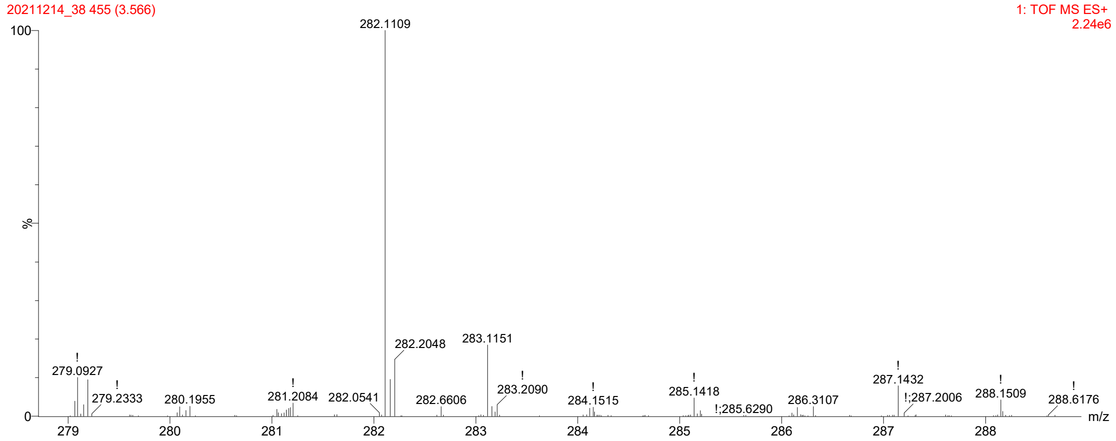

Figure S130 ESI-HRMS spectra of **3g**

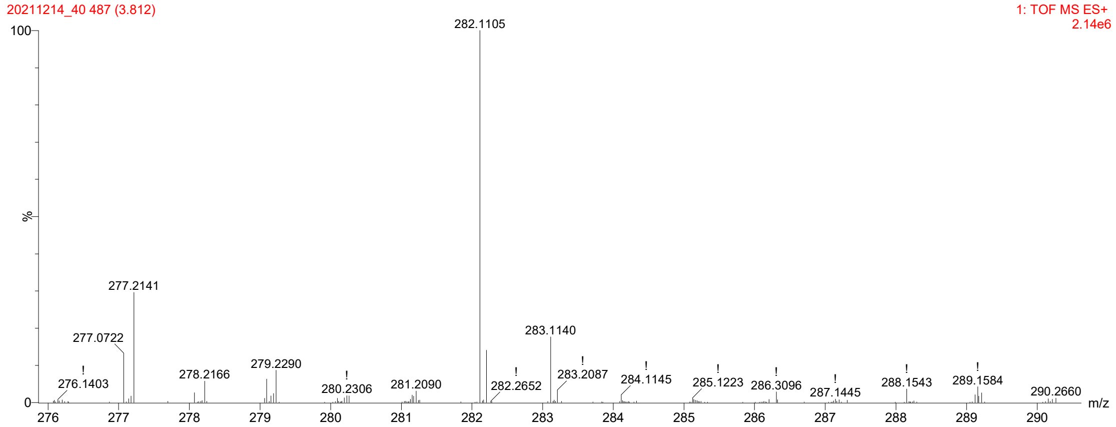

Figure S131 ESI-HRMS spectra of **3h**

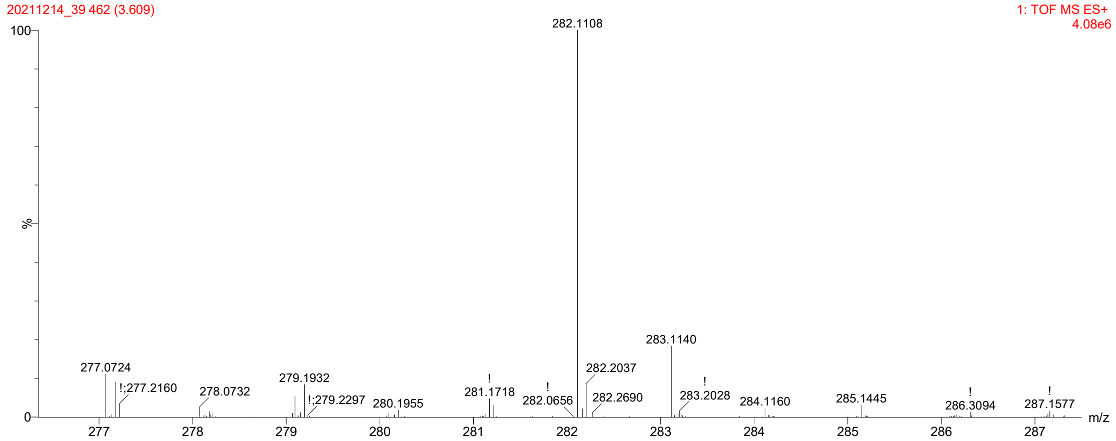

Figure S132 ESI-HRMS spectra of **3i**

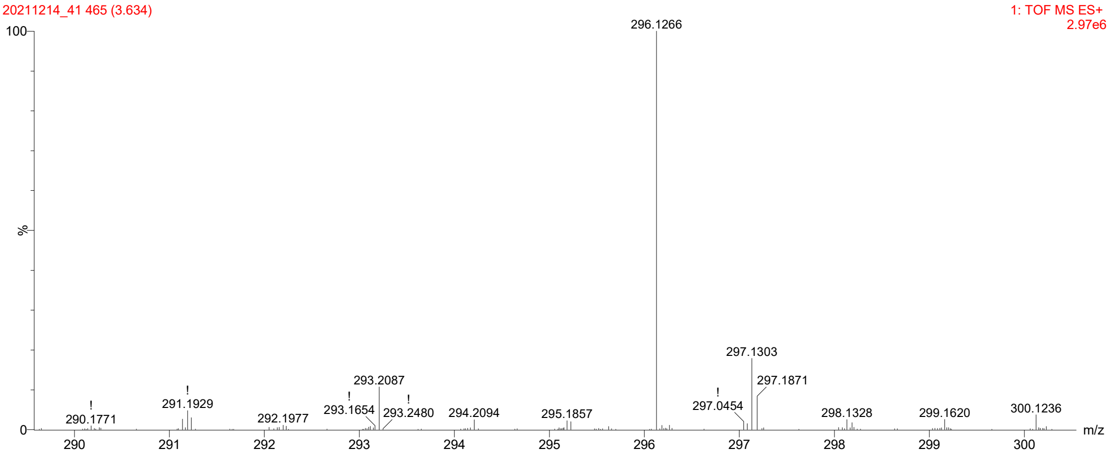

Figure S133 ESI-HRMS spectra of **3j**

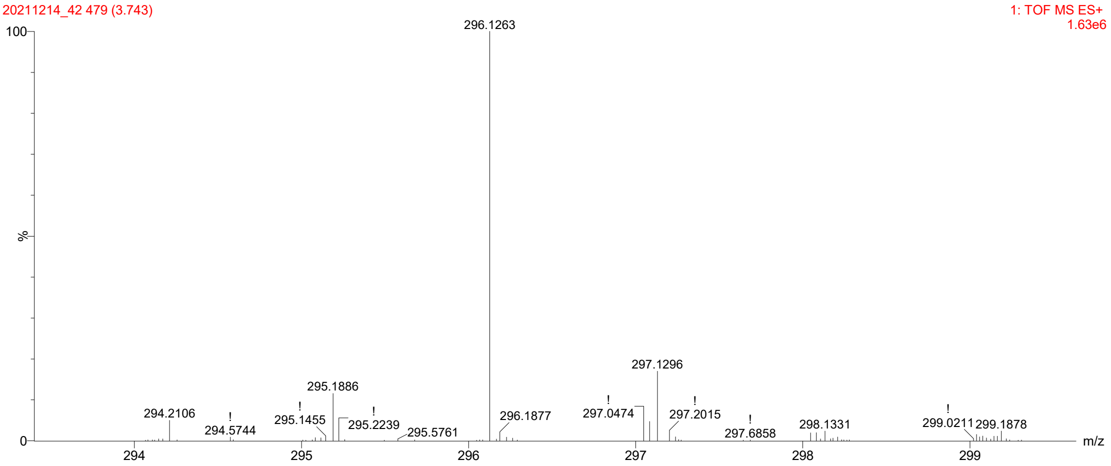

Figure S134 ESI-HRMS spectra of **3k**

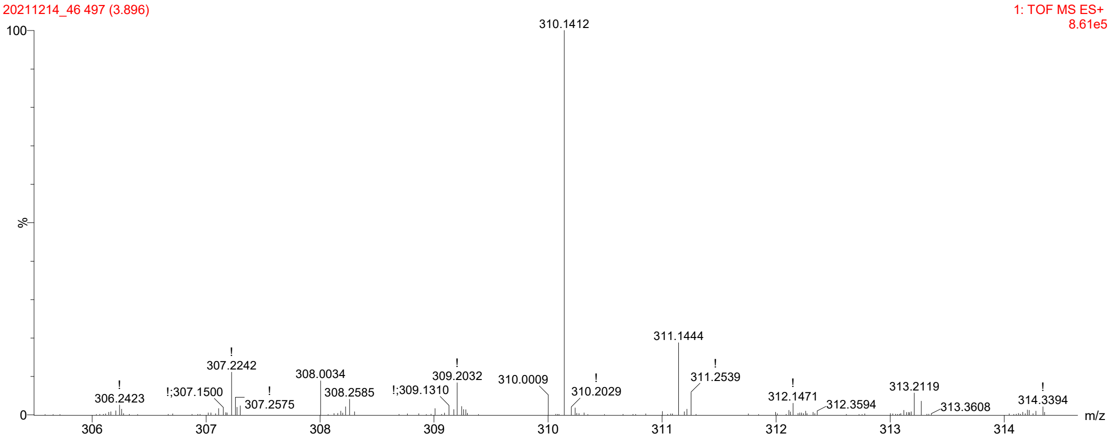

Figure S135 ESI-HRMS spectra of **3l**

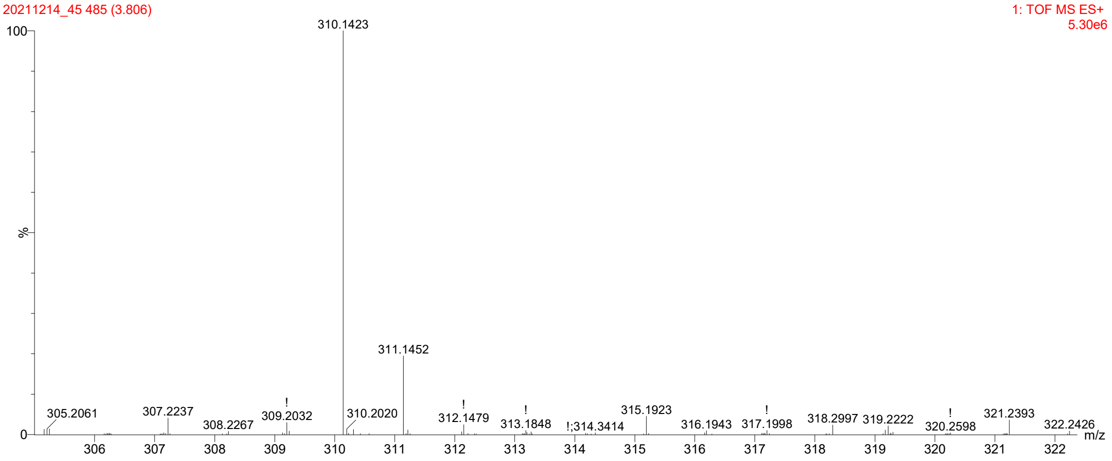

Figure S136 ESI-HRMS spectra of **3m**

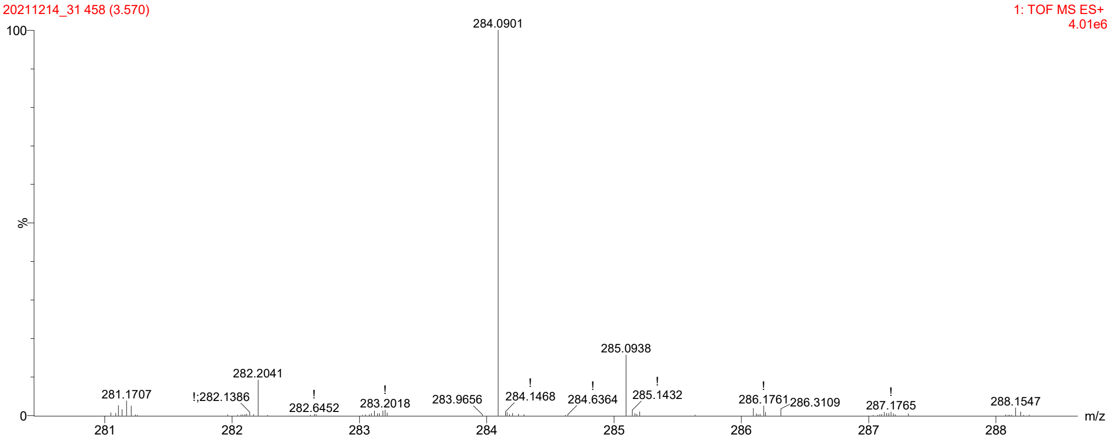

Figure S137 ESI-HRMS spectra of **3n**

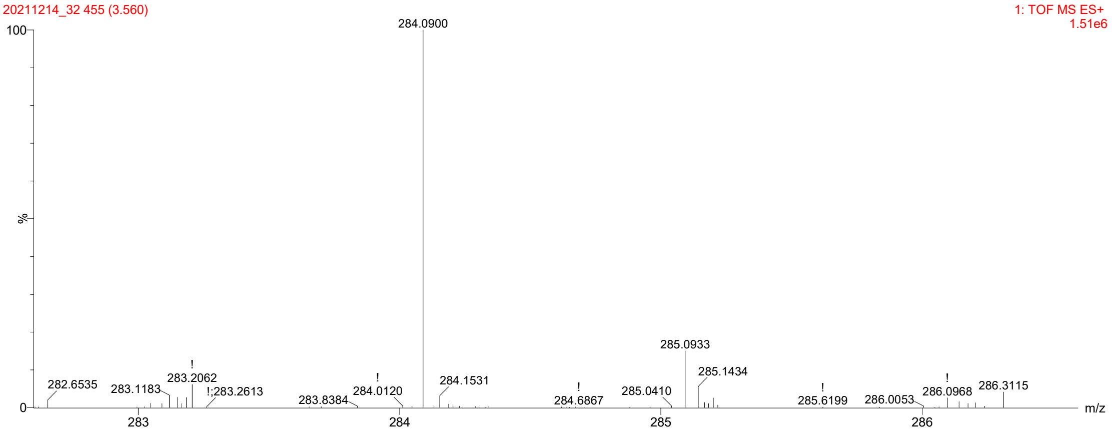

Figure S138 ESI-HRMS spectra of **3o**

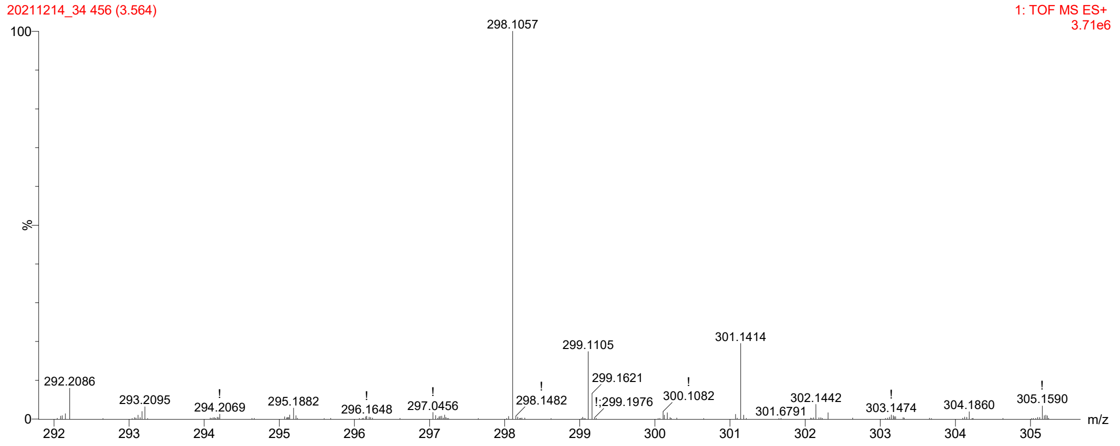

Figure S139 ESI-HRMS spectra of **3p**

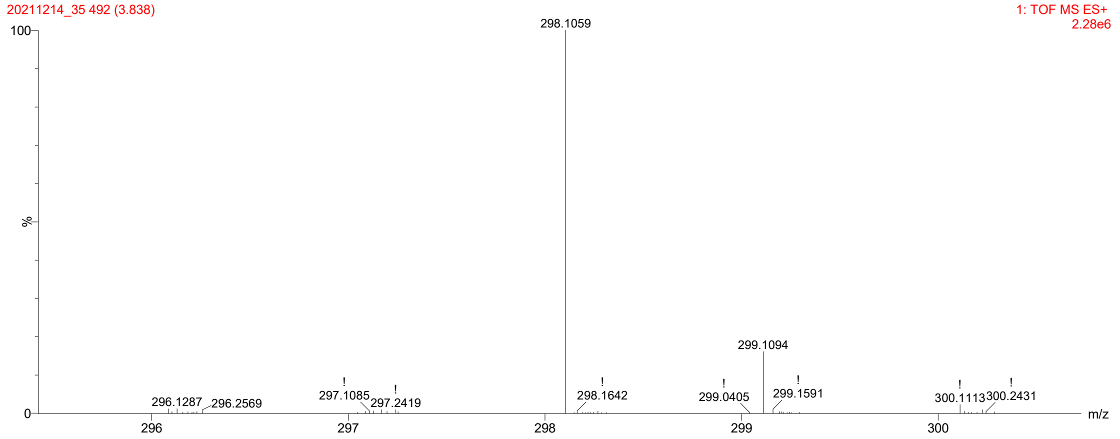

Figure S140 ESI-HRMS spectra of **3q**

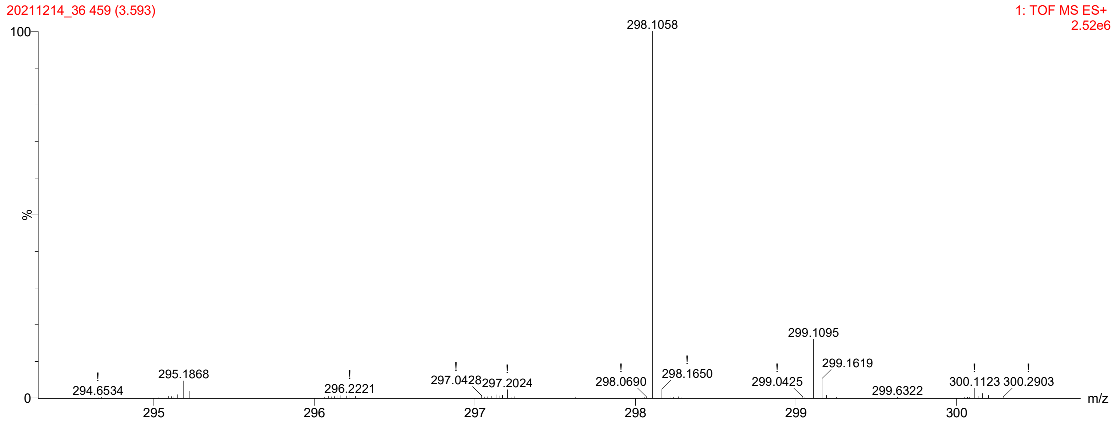

Figure S141 ESI-HRMS spectra of **3r**

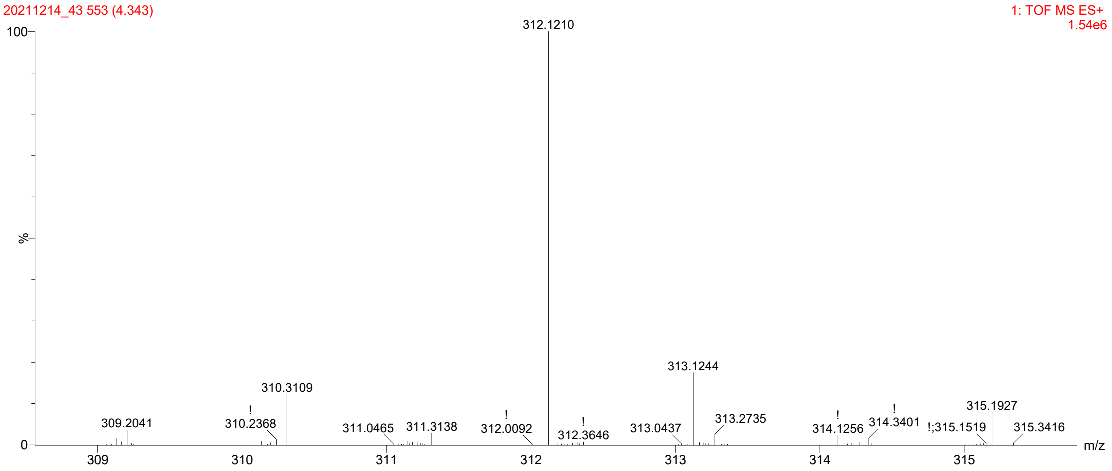

Figure S142 ESI-HRMS spectra of **3s**

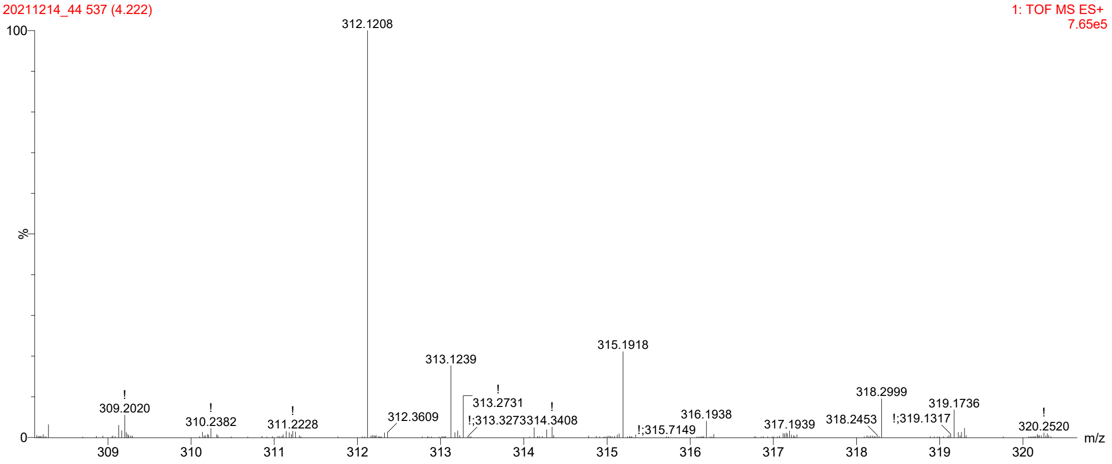

Figure S143 ESI-HRMS spectra of **3t**

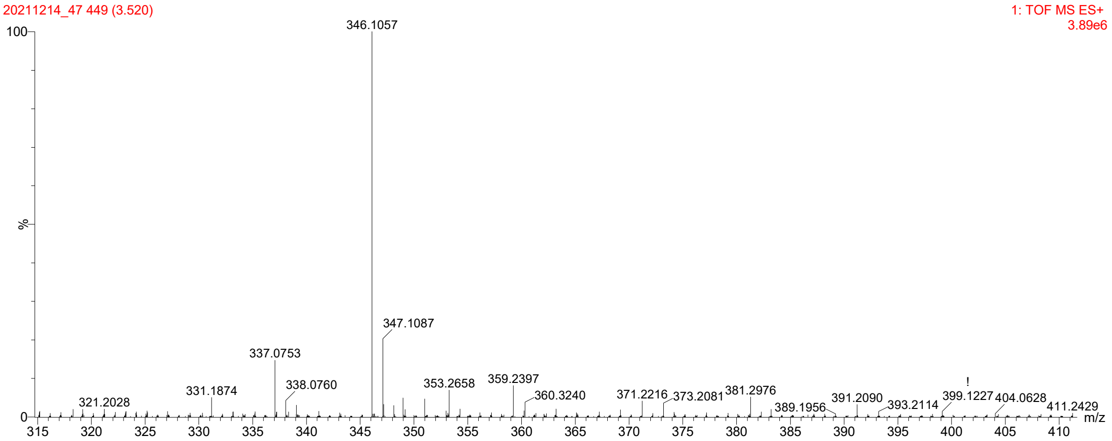

Figure S144 ESI-HRMS spectra of **3u**

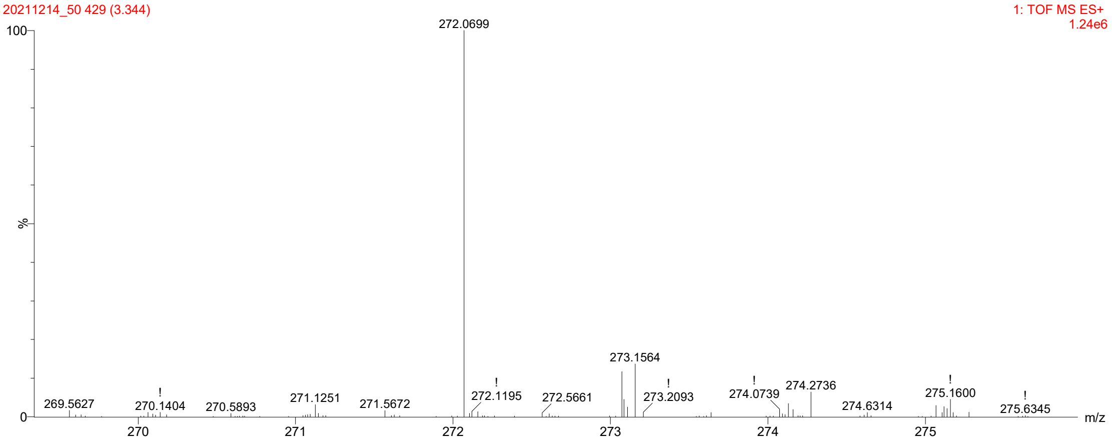

Figure S145 ESI-HRMS spectra of **3v**

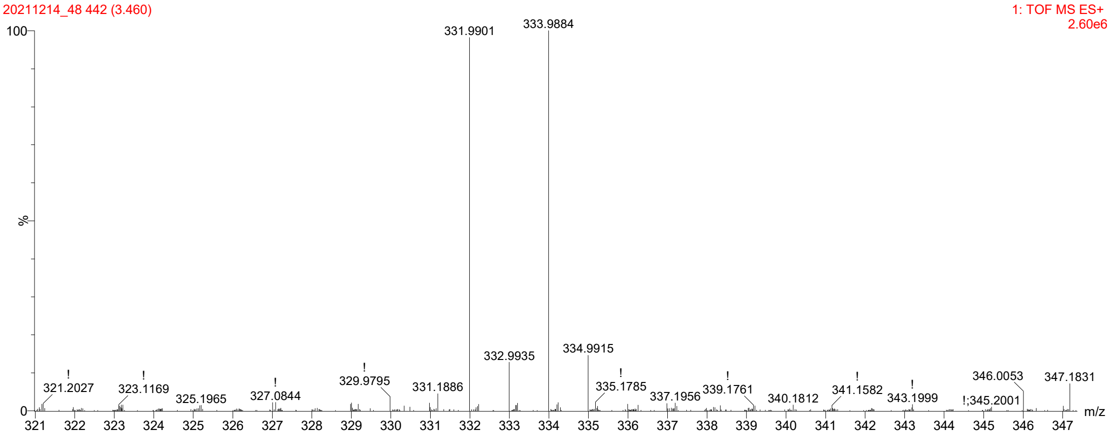

Figure S146 ESI-HRMS spectra of **3w**

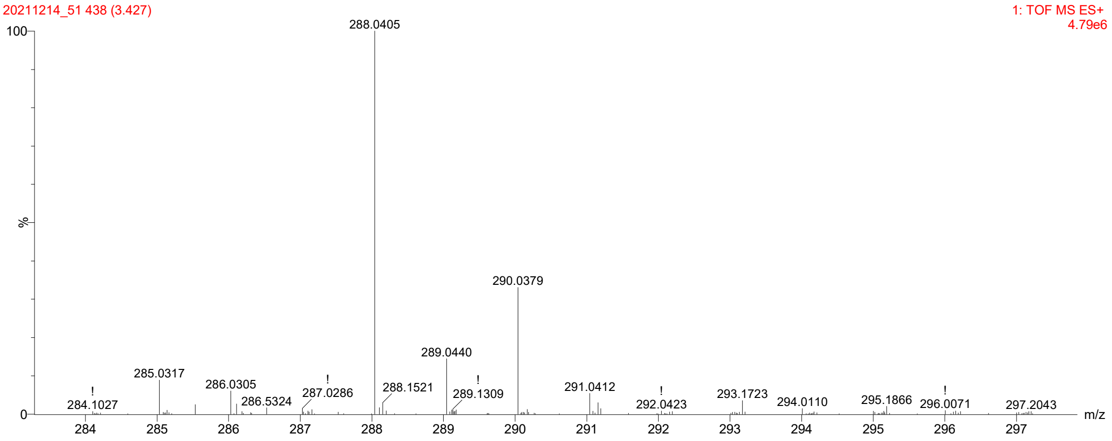

Figure S147 ESI-HRMS spectra of **3x**

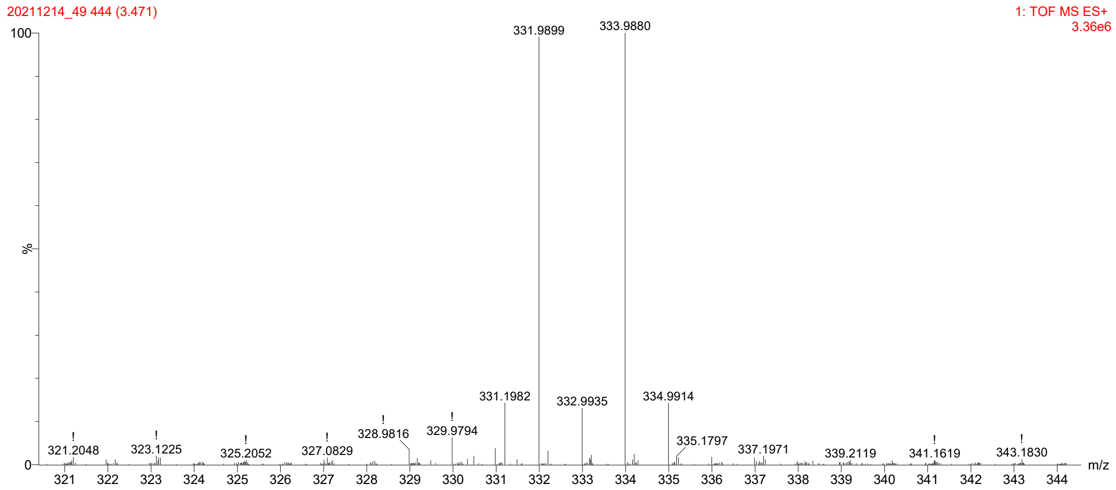

Figure S148 ESI-HRMS spectra of **3y**

5. ESI-HRMS spectra of compounds **4**.

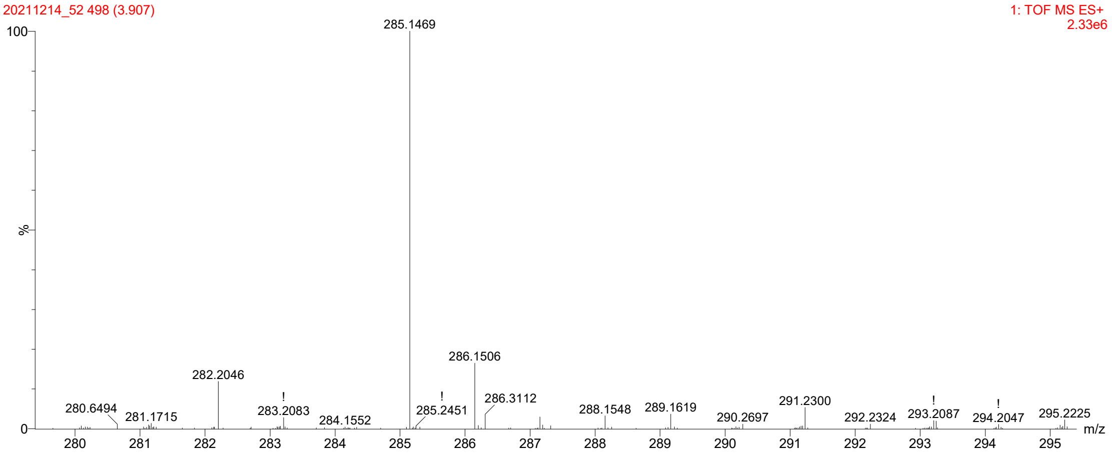

Figure S149 ESI-HRMS spectra of **4a**

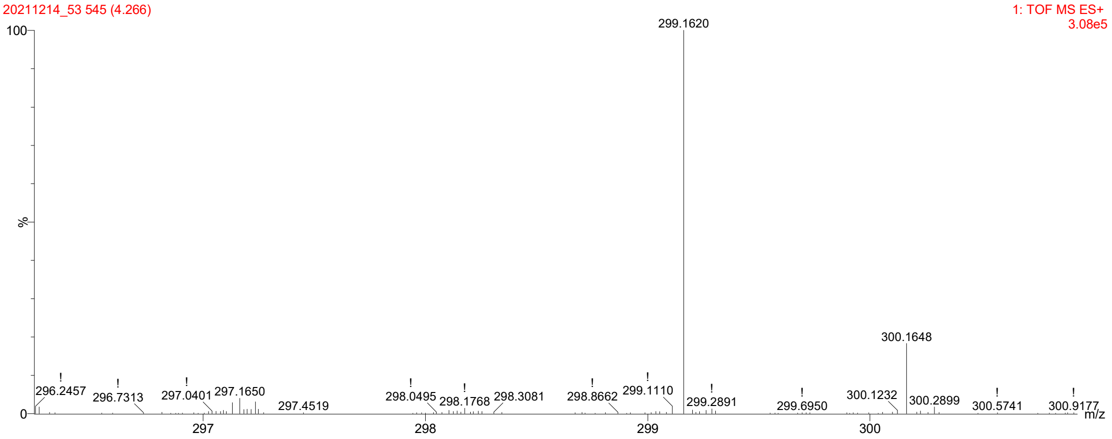

Figure S150 ESI-HRMS spectra of **4b**

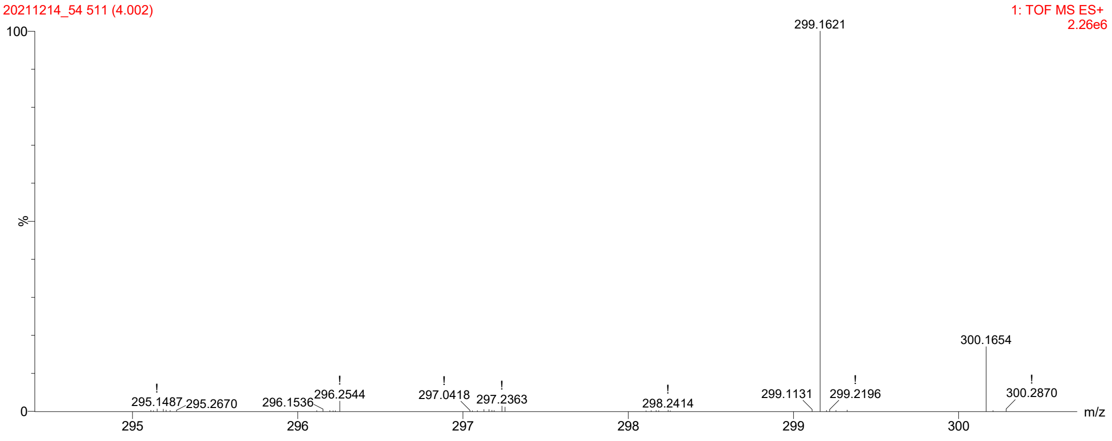

Figure S151 ESI-HRMS spectra of **4c**

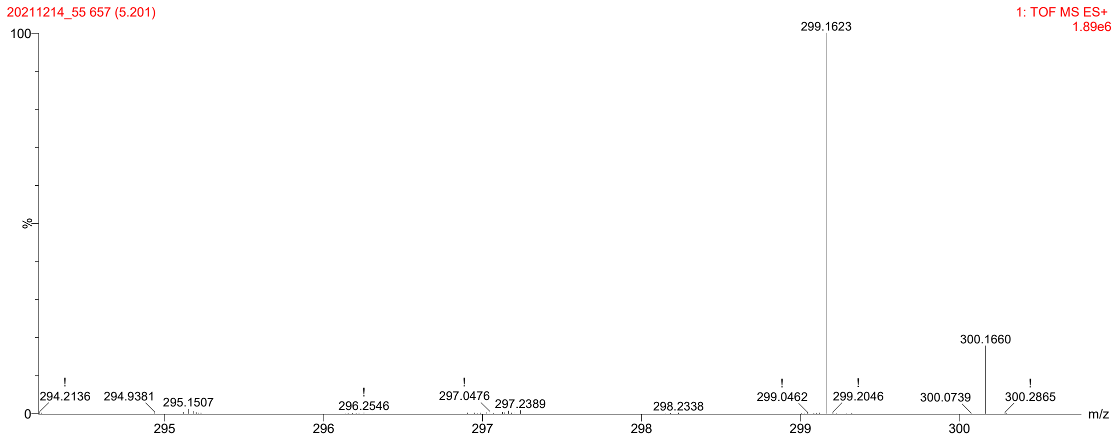

Figure S152 ESI-HRMS spectra of **4d**

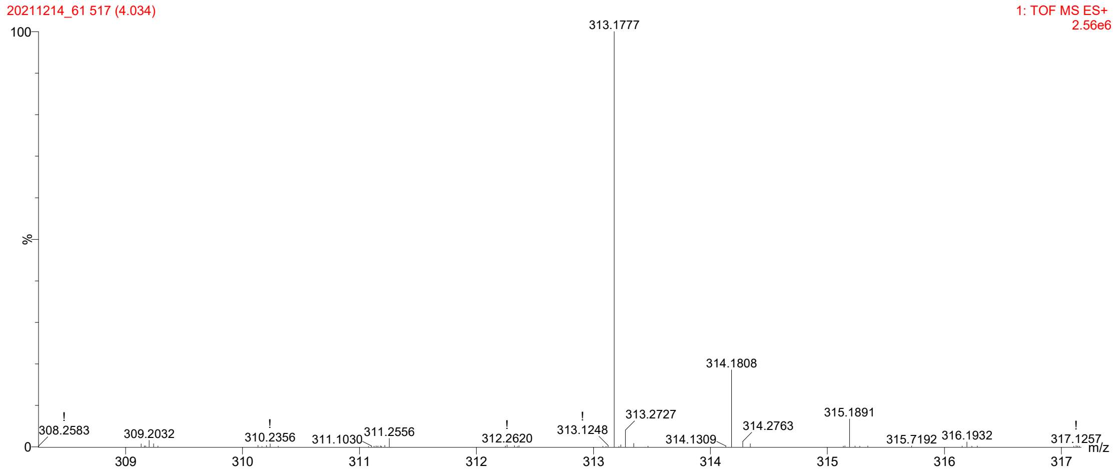

Figure S153 ESI-HRMS spectra of **4e**

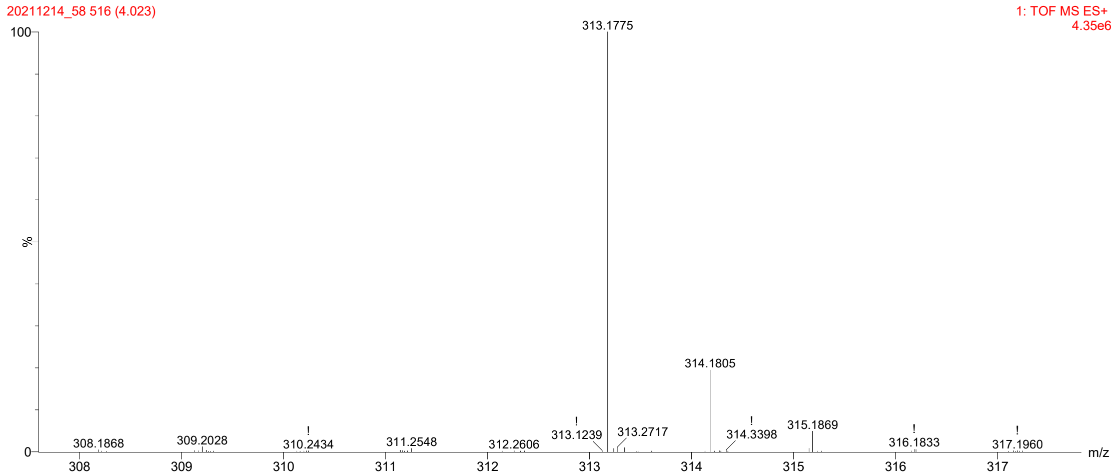

Figure S154 ESI-HRMS spectra of **4f**

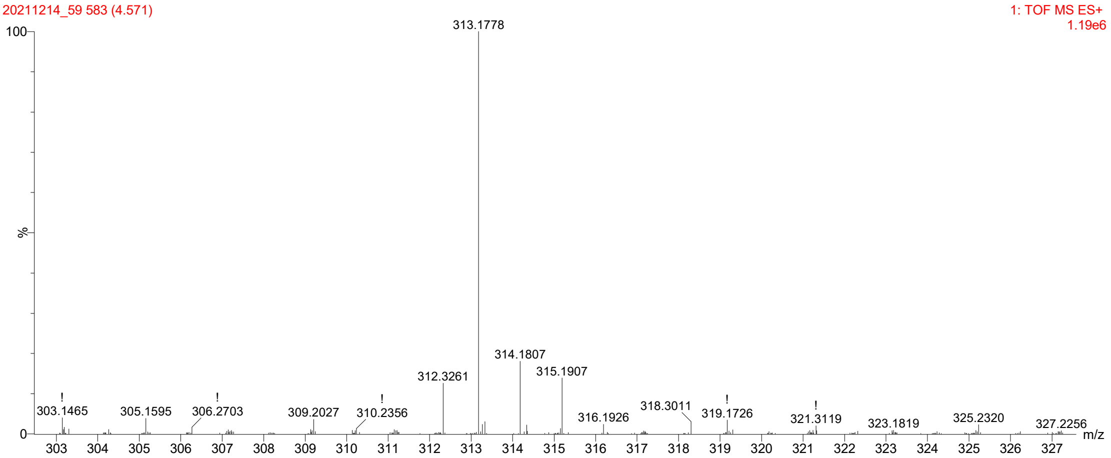

Figure S155 ESI-HRMS spectra of **4g**

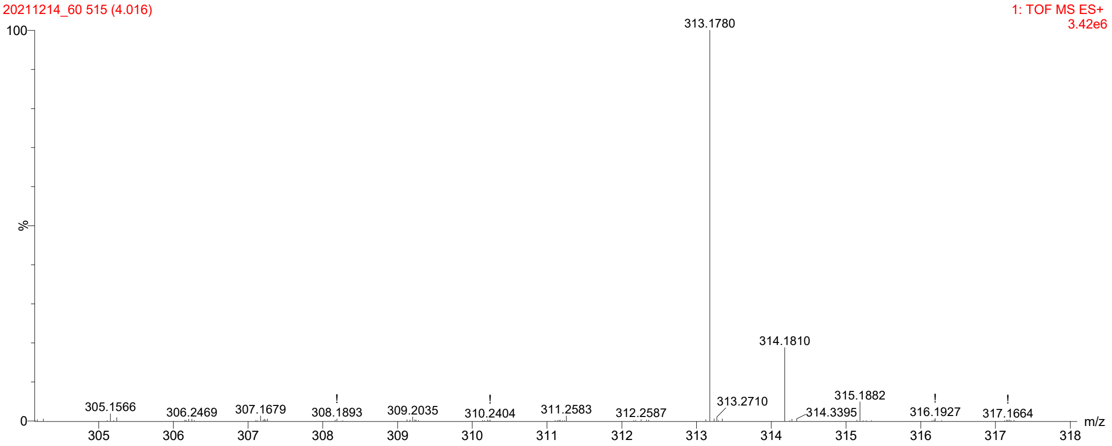

Figure S156 ESI-HRMS spectra of **4h**

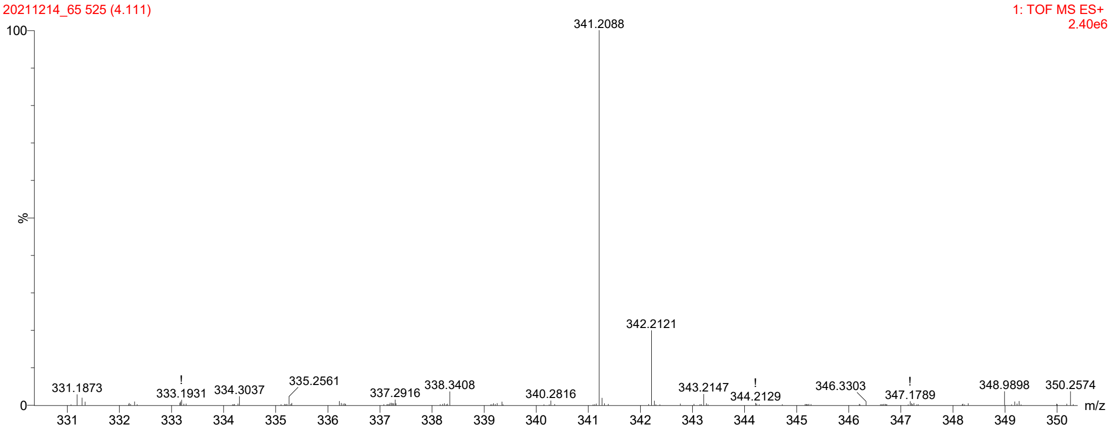

Figure S157 ESI-HRMS spectra of **4i**

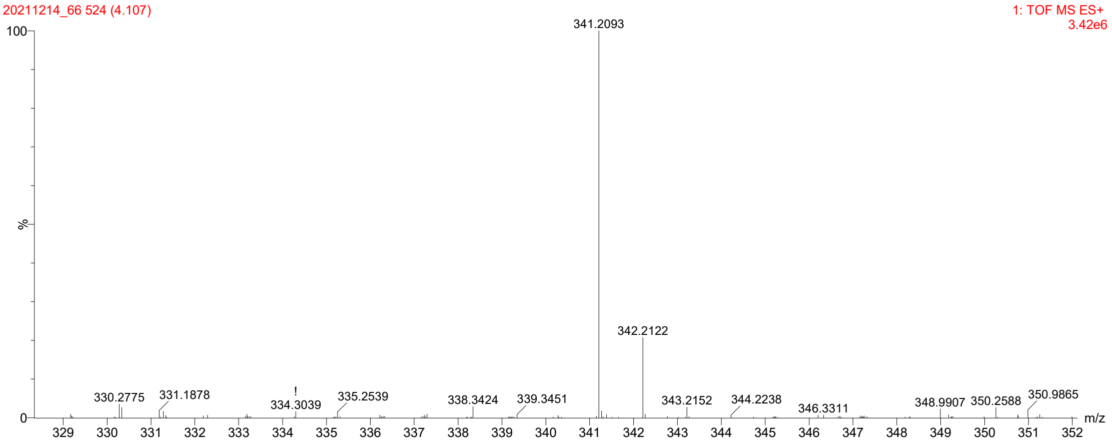

Figure S158 ESI-HRMS spectra of **4j**

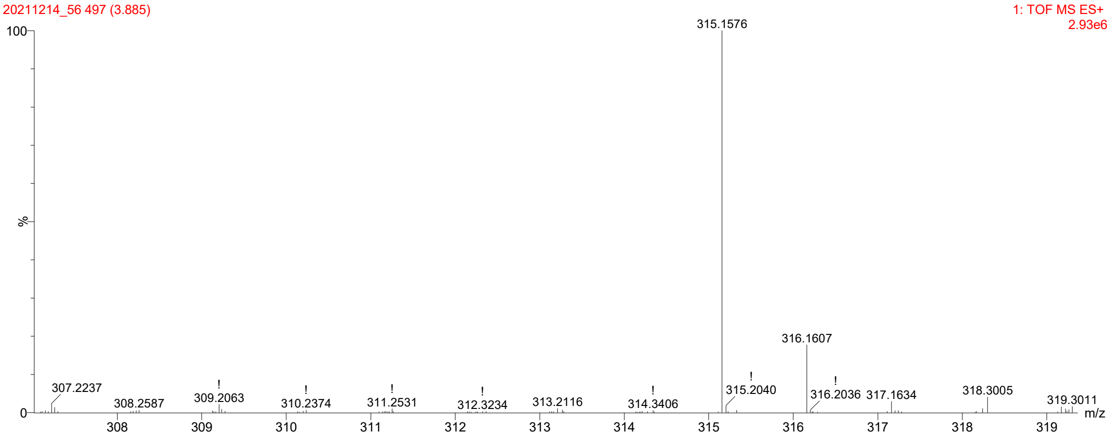

Figure S159 ESI-HRMS spectra of **4k**

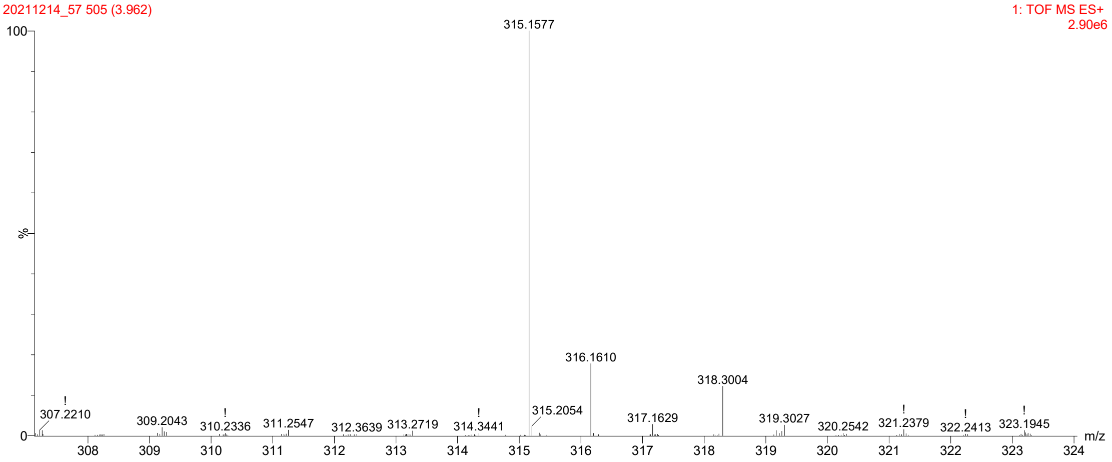

Figure S160 ESI-HRMS spectra of **4l**

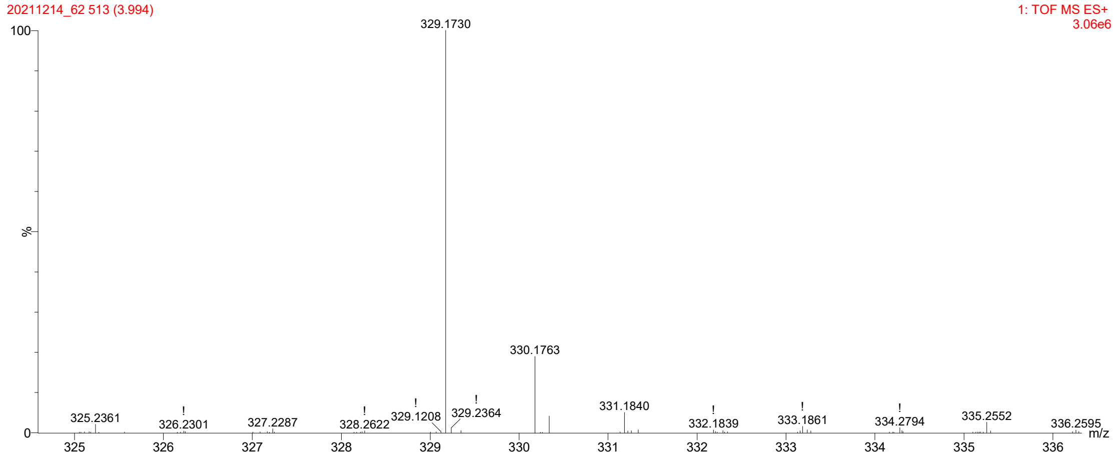

Figure S161 ESI-HRMS spectra of **4m**

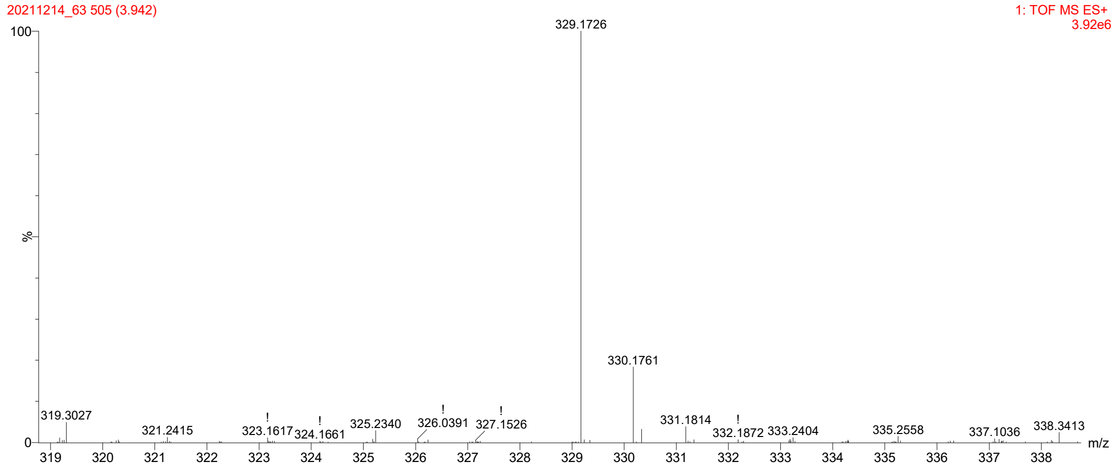

Figure S162 ESI-HRMS spectra of **4n**

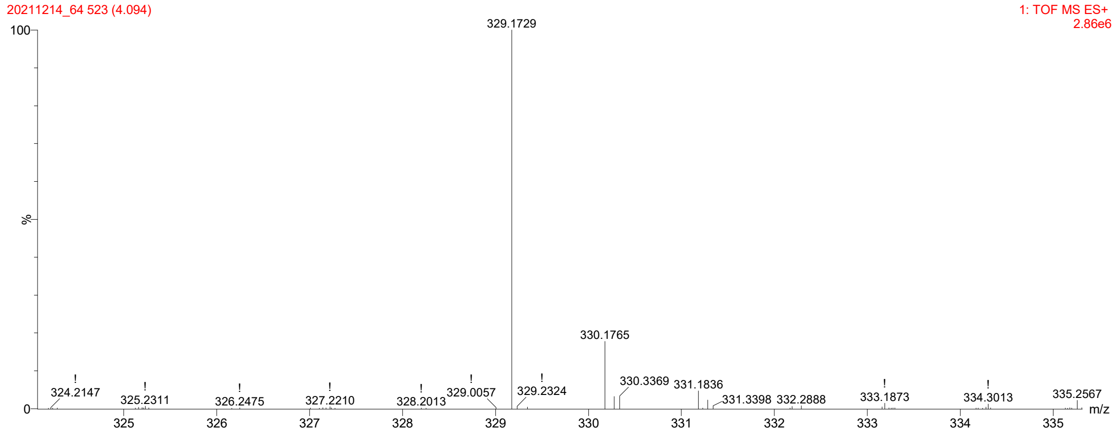

Figure S163 ESI-HRMS spectra of **4o**

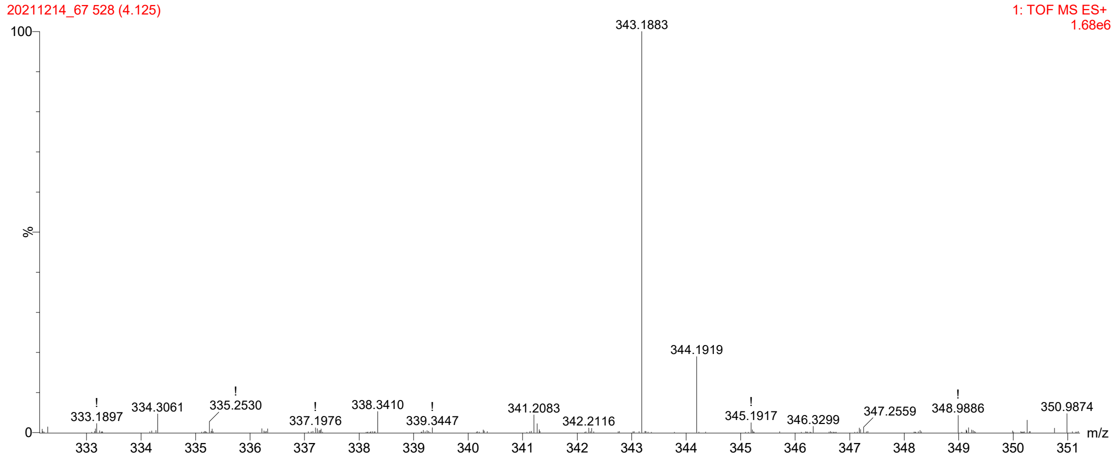

Figure S164 ESI-HRMS spectra of **4p**

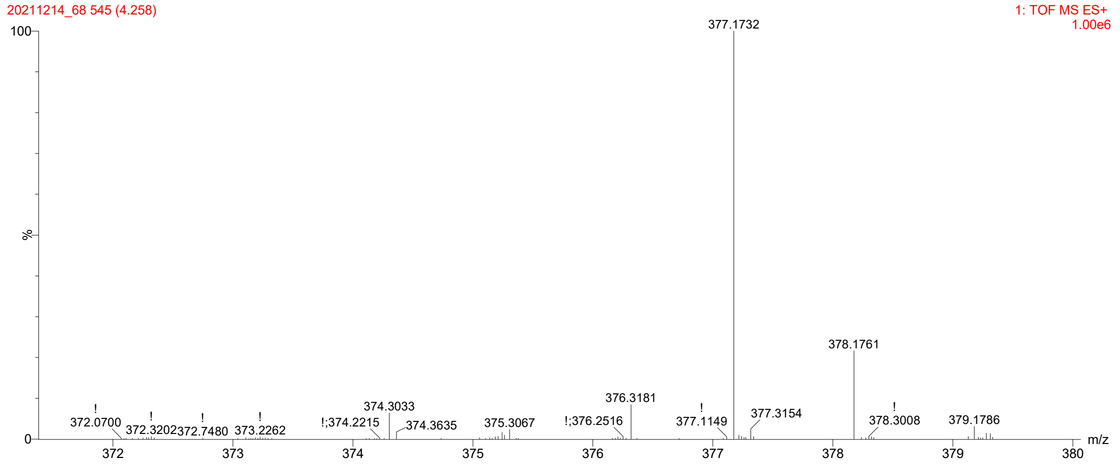

Figure S165 ESI-HRMS spectra of **4q**

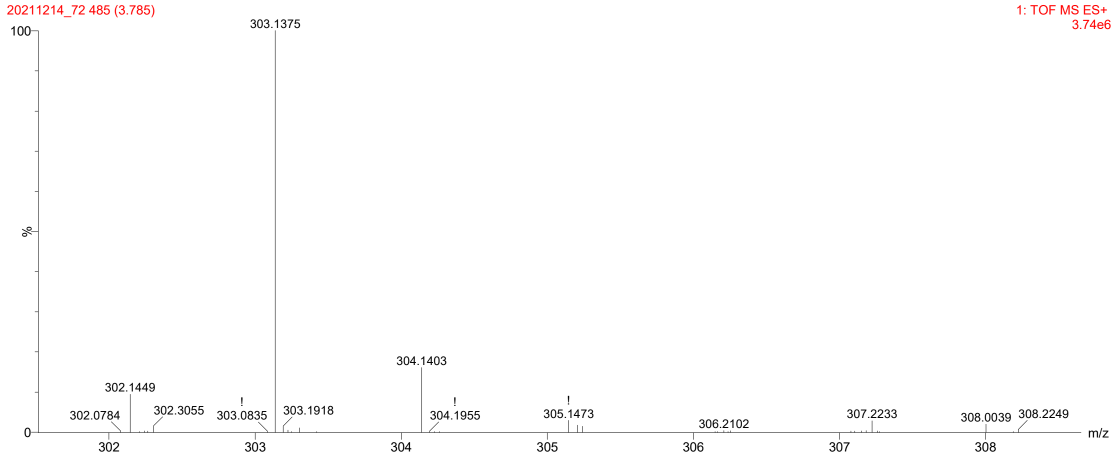

Figure S166 ESI-HRMS spectra of **4r**

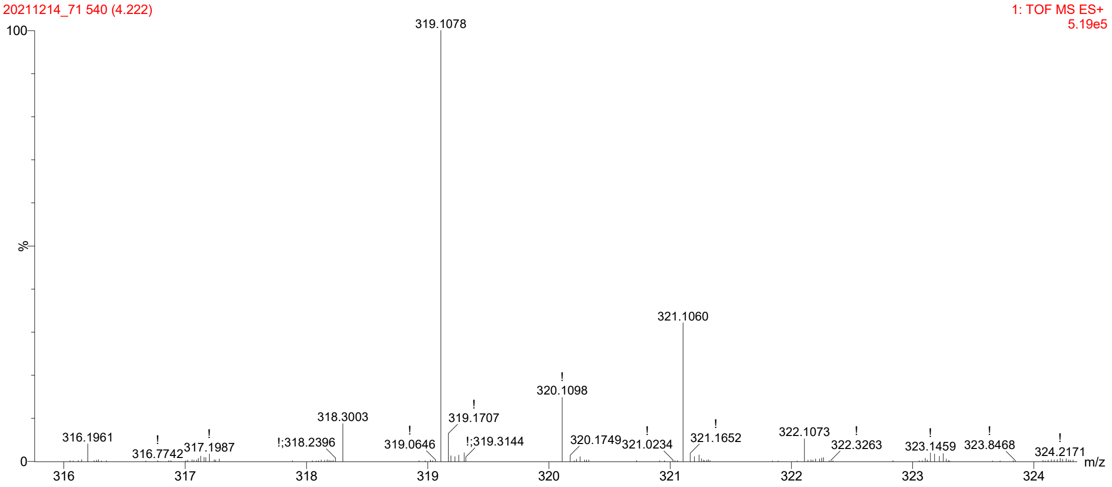

Figure S167 ESI-HRMS spectra of **4s**

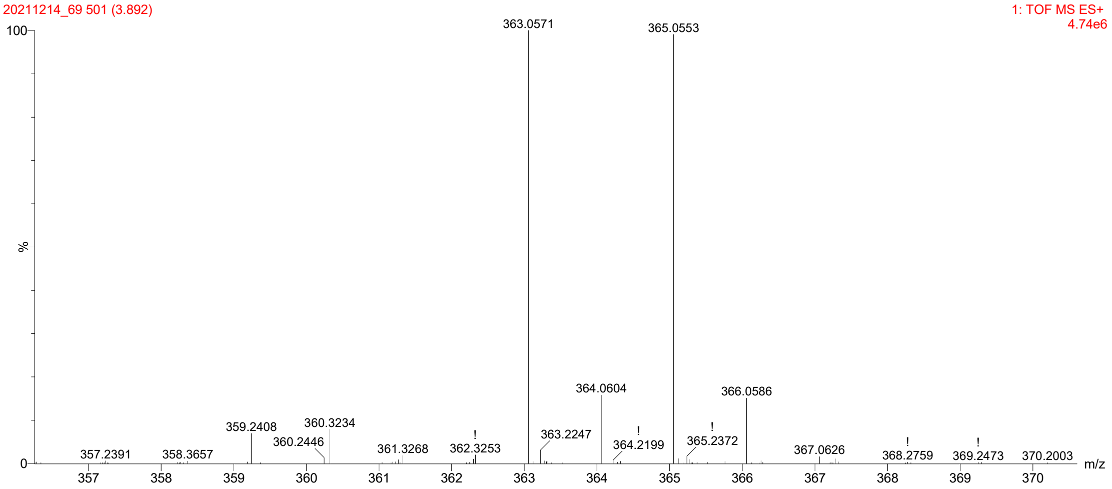

Figure S168 ESI-HRMS spectra of **4t**

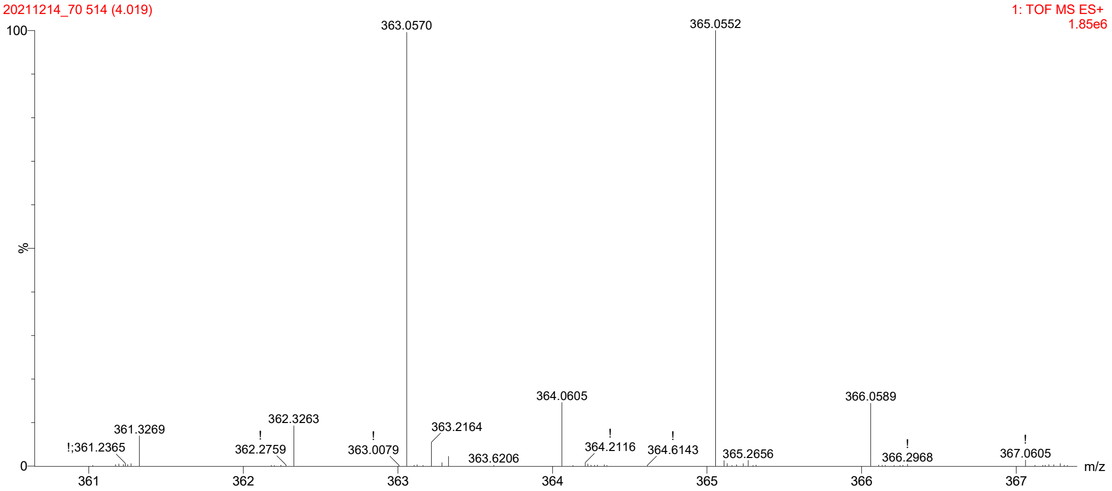

Figure S169 ESI-HRMS spectra of **4u**

6. ESI-HRMS spectra of compounds **5**.

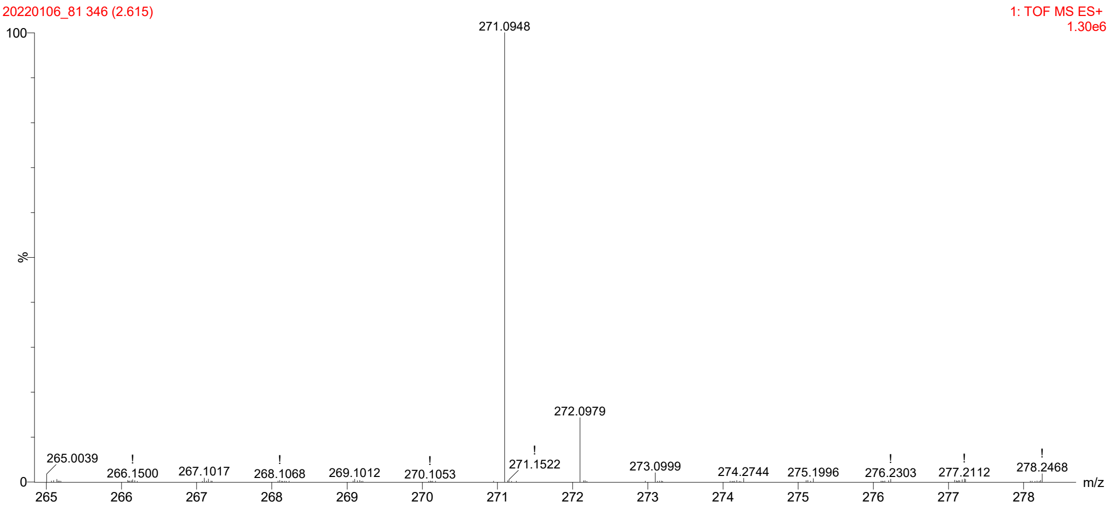

Figure S170 ESI-HRMS spectra of **5a**

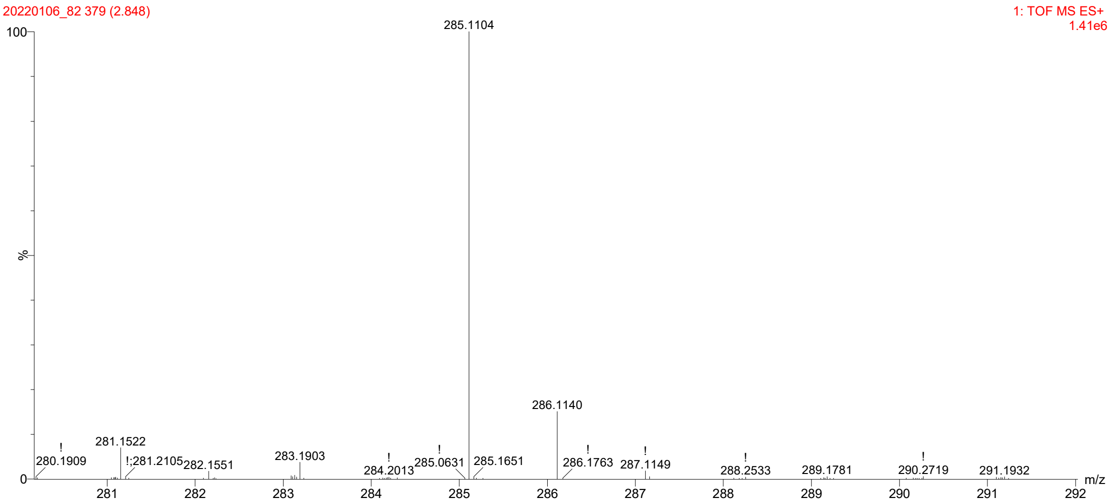

Figure S171 ESI-HRMS spectra of **5b**

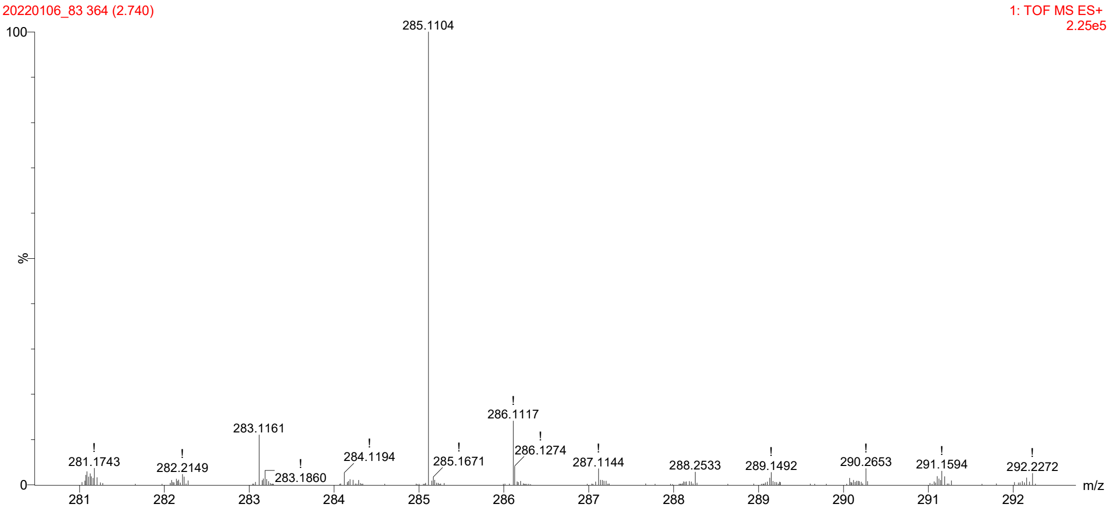

Figure S172 ESI-HRMS spectra of **5c**

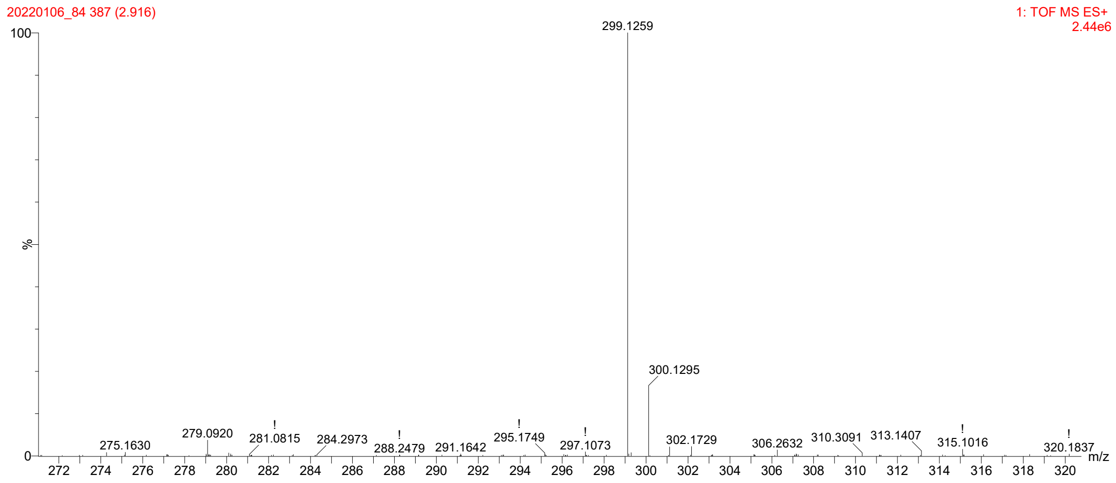

Figure S173 ESI-HRMS spectra of **5d**

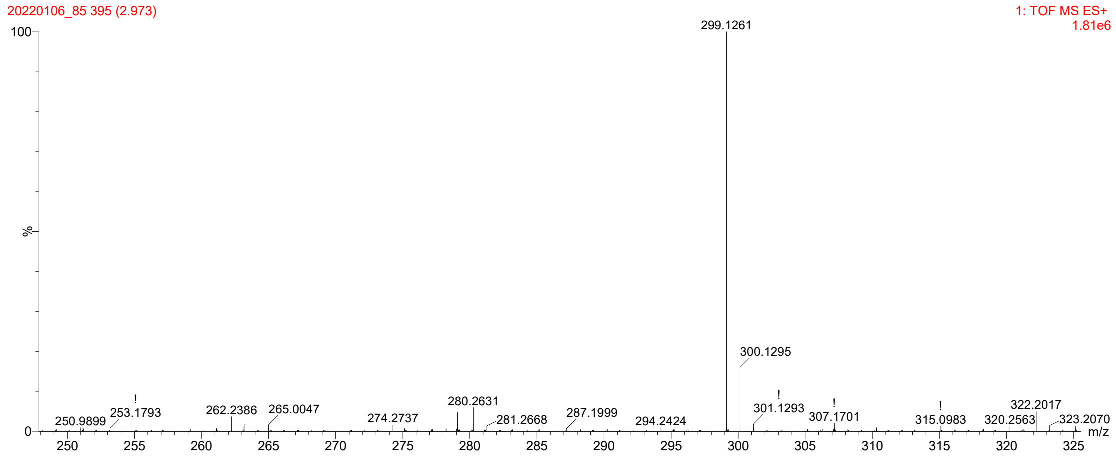

Figure S174 ESI-HRMS spectra of **5e**

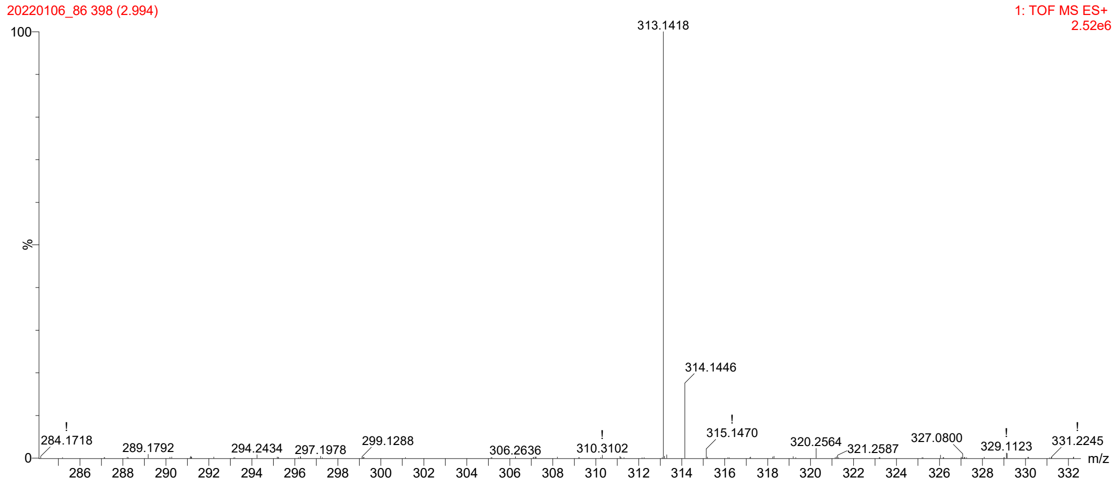

Figure S175 ESI-HRMS spectra of **5f**

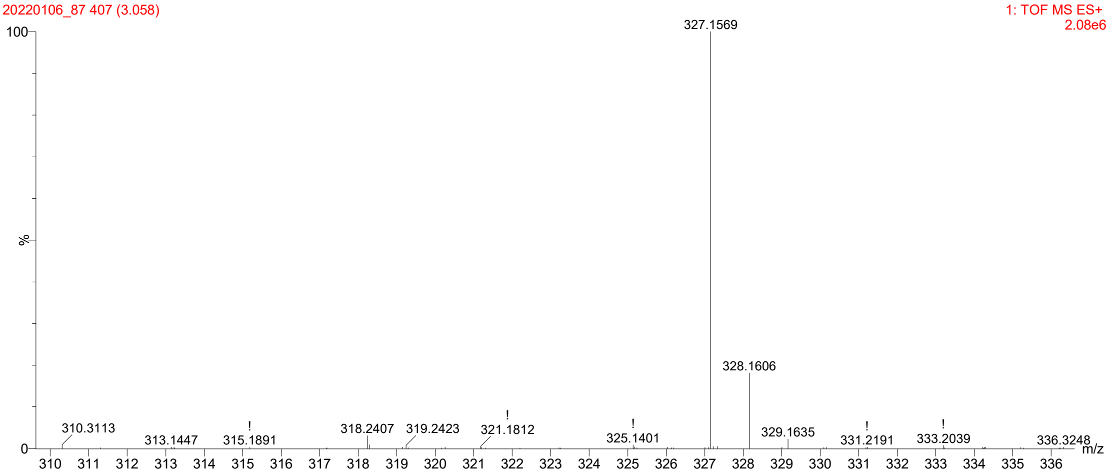

Figure S176 ESI-HRMS spectra of **5g**

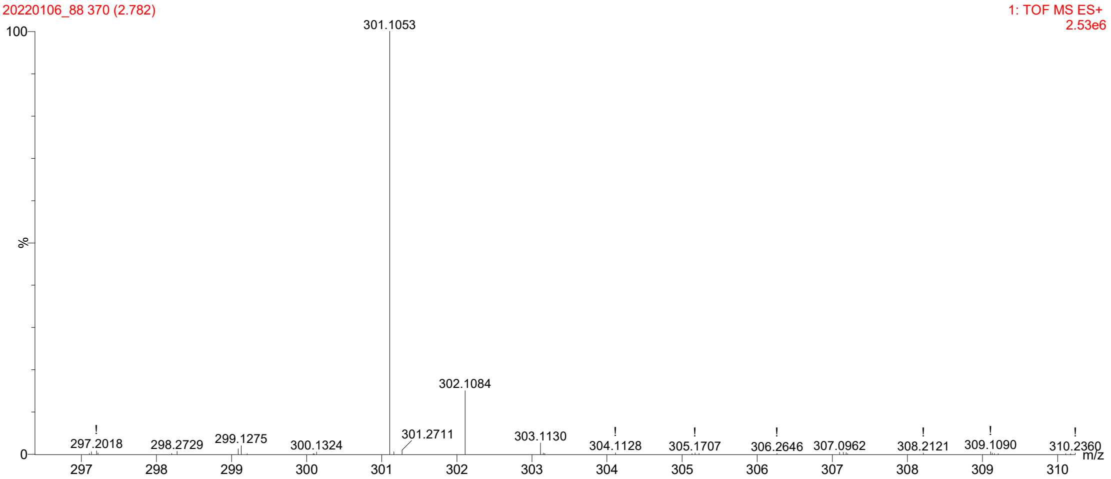

Figure S177 ESI-HRMS spectra of **5h**

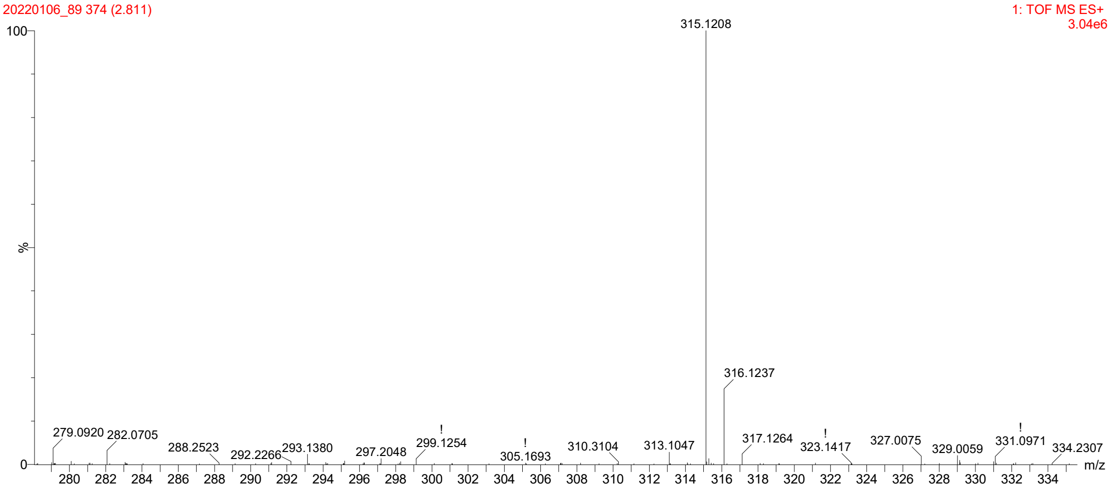

Figure S178 ESI-HRMS spectra of **5i**

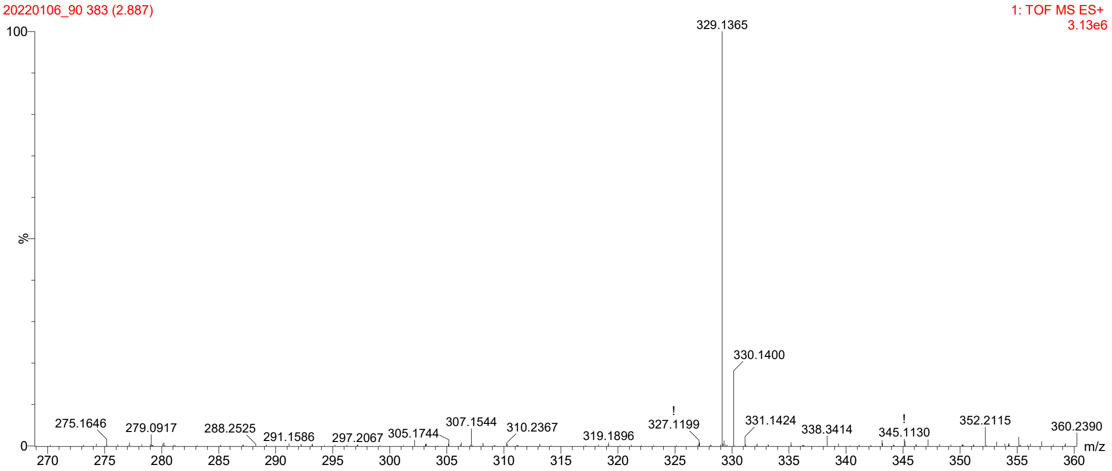

Figure S179 ESI-HRMS spectra of **5j**

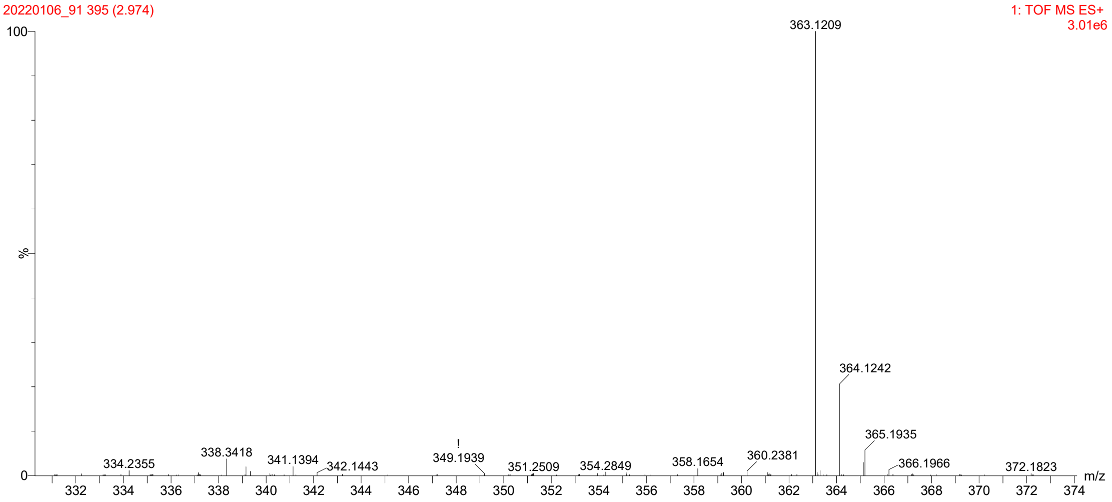

Figure S180 ESI-HRMS spectra of **5k**

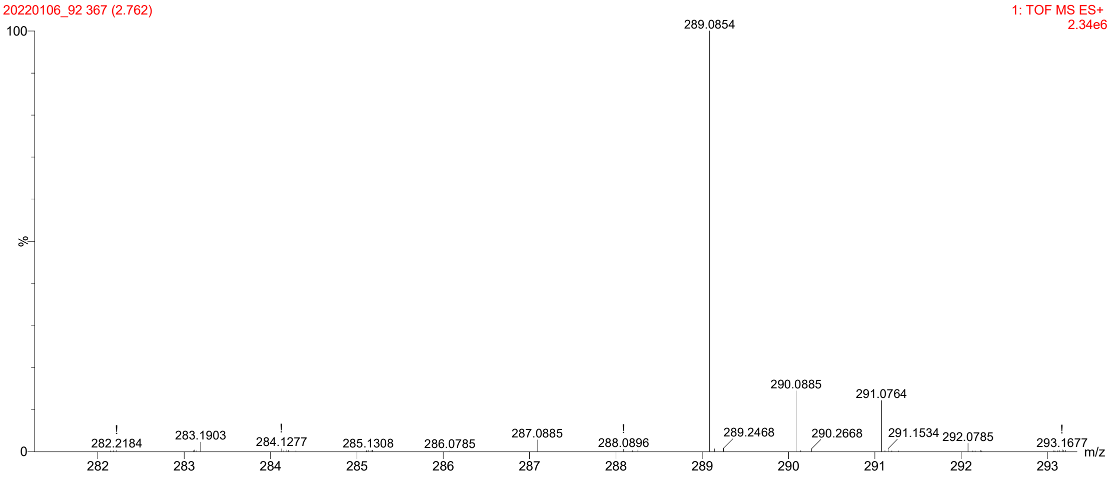

Figure S181 ESI-HRMS spectra of **5l**

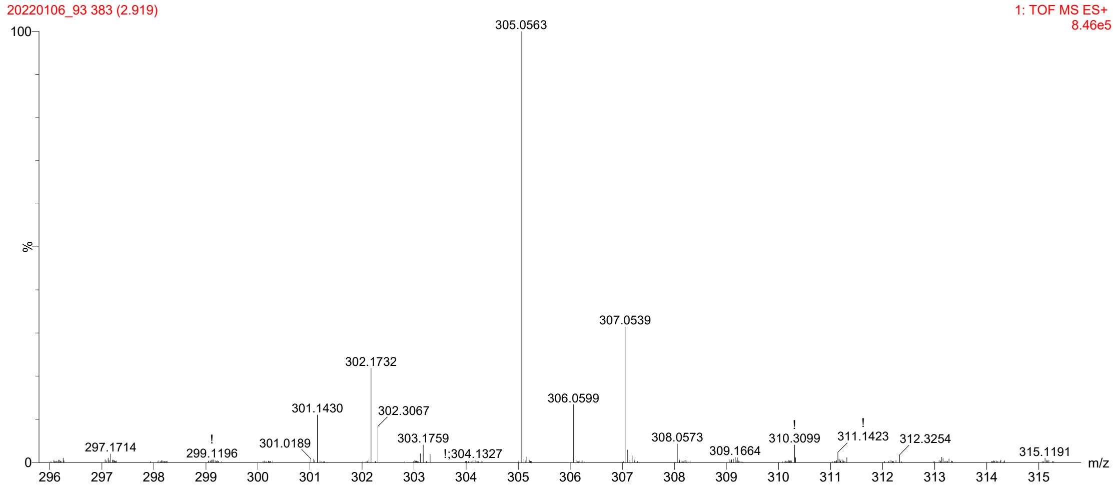

Figure S182 ESI-HRMS spectra of **5m**

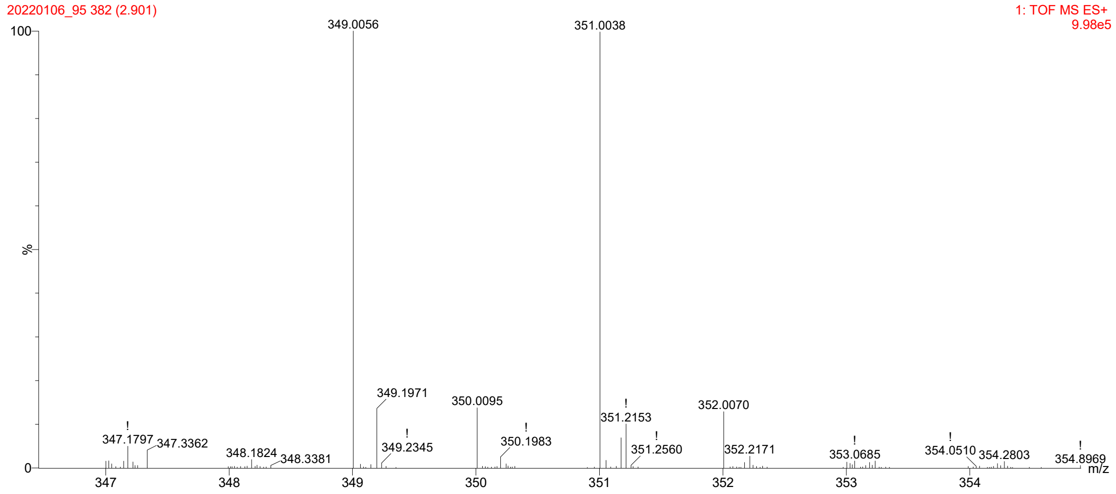

Figure S183 ESI-HRMS spectra of **5n**

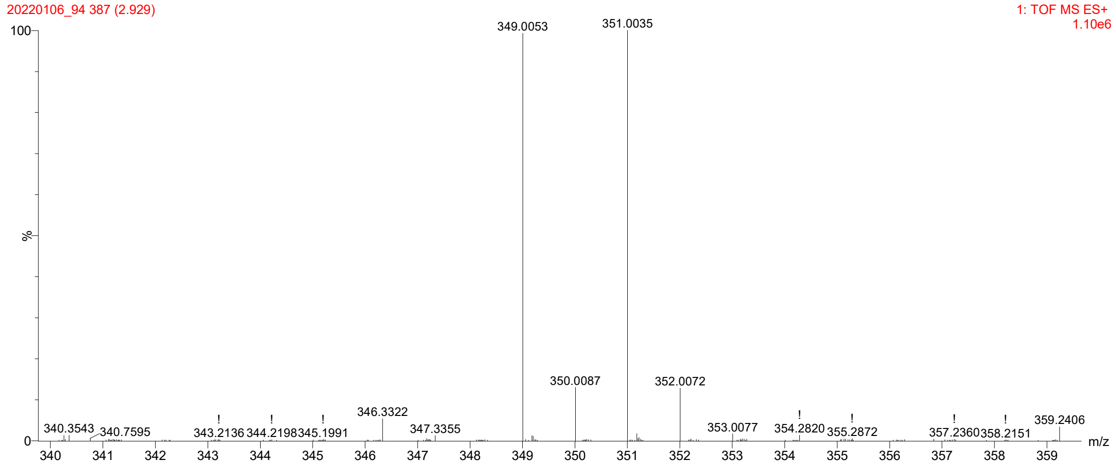

Figure S184 ESI-HRMS spectra of **5o**
